# Antimicrobial Activity of a Polymyxin A-like Compound and Characterisation of the Cognate Biosynthetic Gene Cluster Within the Genome of the Producing *Paenibacillus polymyxa*

**DOI:** 10.3390/antibiotics15080816

**Published:** 2026-08-21

**Authors:** Amy McLeman, Alexander D. H. Kingdon, Robin Hoeven, George Taylor, Ellie Allman, Issra Bulgasim, Claudia McKeown, Richard N. Goodman, Sabrina Moyo, Adam P. Roberts

**Affiliations:** 1 Centre for Drugs and Diagnostics, Department of Tropical Disease Biology, Liverpool School of Tropical Medicine, Liverpool L3 5QA, UK; amy.mcleman@lstmed.ac.uk (A.M.); alexander.kingdon@lsmted.ac.uk (A.D.H.K.); ellie.allman@lstmed.ac.uk (E.A.); issra.bulgasim@nhs.net (I.B.); claudia.mckeown@lstmed.ac.uk (C.M.); richard.goodman@lstmed.ac.uk (R.N.G.); sabrina.moyo@lstmed.ac.uk (S.M.); 2 Faculty of Biology, Medicine and Health, The University of Manchester, Manchester M13 9PL, UK; robin.hoeven@manchester.ac.uk (R.H.); george.taylor-2@manchester.ac.uk (G.T.); 3 Department of Microbiology, Leeds Teaching Hospital NHS Trust, Leeds LS9 7TF, UK; 4 Department of Clinical Science, University of Bergen, 5020 Bergen, Norway

**Keywords:** colistin, polymyxin, mass spectrometry, biosynthetic gene cluster, antibiotic resistance, mcr

## Abstract

**Background**: Here, we report the isolation and identification of a *Paenibacillus polymyxa* strain from the citizen science project, Swab and Send. *P. polymyxa* is well known for its production of polymyxin E (colistin), and polymyxin B. Polymyxins are ranked in the highest-priority critically important antimicrobial classification by the WHO and are of particular importance for treating Gram-negative multi-drug-resistant pathogens. Due to their clinical use, most of the literature focusses on these polymyxin variants, and there is sparse genetic research on other polymyxin variants. **Methods**: The *Paenibacillus polymyxa* 1G strain was isolated and tested for antimicrobial activity using a combination of on-agar and liquid-based inhibition assays to detect antimicrobial activity against *Escherichia coli*. The isolate was also tested for activity in liquid media against a panel of isolates showing various resistances to test if our strain could overcome current clinically important resistance mechanisms. Our isolate was whole-genome-sequenced, and bioinformatics was carried out on the resulting sequence to analyse the relevant biosynthetic gene cluster. The cell-free supernatant from the *P. polymyxa* 1G isolate was also analysed using mass spectrometry to confirm the production of the polymyxin. **Results**: *P. polymyxa* 1G was active on agar and resulted in antimicrobial activity, with a 99% reduction in area under the curve when tested in liquid media against *E. coli*. *P. polymyxa* 1G did not inhibit the growth of *E. coli* containing *mcr-1* or *mcr-4* colistin-resistance genes. Using whole genome sequencing, we are able to describe the biosynthetic gene cluster of the putative polymyxin A, compare the *pmxA*, *pmxB*, and *pmxE* genes to five other polymyxin genes that encode known polymyxin variants, and provide mass spectrometry data that supports the production of polymyxin A_1_ (1157 *m*/*z*) and A_2_ (1143 *m*/*z*). **Conclusions**: Here, we report the isolation and identification of a *P. polymyxa* strain producing a polymyxin A-like compound that was discovered through the citizen science project, Swab and Send. We add to the genetic and mass spectrometry data for polymyxin A, and demonstrate that this putative polymyxin A, produced naturally by our strain, is unable to overcome the current clinically relevant resistance mechanisms to colistin.

## 1. Introduction

Antimicrobial resistance (AMR) is one of the largest threats to global health, with approximately 4.95 million associated deaths in 2019, and modelling forecasts estimate up to 8.22 million deaths associated with AMR by 2050 [1,2]. One approach for tackling the threat of AMR is the discovery of novel antimicrobials [3,4,5]. Most antibiotics are derived from, or inspired by, natural products originating from bacteria or fungi, often found in soil [5,6,7]. Multi-drug-resistant (MDR) Gram-negative bacteria are of particular concern, with ever-rising numbers of infections reported; antibiotic-resistant *Klebsiella pneumoniae*, *Acinetobacter baumannii*, *Pseudomonas aeruginosa*, and *Escherichia coli* are some of the noted pathogens posing an important challenge in global healthcare [2,8,9,10,11]. There is potential to substantially alleviate future morbidity caused by MDR Gram-negative bacteria by 11.1 million between 2025 and 2050 through the development of new antimicrobials [2].

One of the early discoveries made in the “Golden Age” of natural product discovery in 1947, by Y. Koyama, was polymyxin E, more commonly referred to as colistin [12]. *Paenibacillus polymyxa*, previously referred to as *Bacillus polymyxa*, has been extensively studied due its production of many active metabolites, including multiple antibiotics such as fusaricidin and polymyxin [13,14]. The discovery of polymyxin from *B. polymyxa* by Benedict and Langlykke (1947), and subsequent research around the time of discovery, showed proof of antimicrobial discovery by analysing cell-free supernatant (CFS) [14,15]. Despite how long the polymyxins have been known, there are knowledge gaps around the production of these antimicrobials, particularly genome sequence data for polymyxin biosynthesis [16]. To the best of our knowledge, only five polymyxin biosynthetic gene clusters (BGCs) with the cognate polymyxin variant have been reported [17,18,19,20].

Initially thought of as a promising new antibiotic due to its low levels of resistance and good activity against Gram-negative bacteria, colistin (polymyxin E) was abandoned for clinical use by the 1980s due to its toxicity [12,17,21,22]. Polymyxins are associated with nephrotoxicity, neurotoxicity, and allergic and topical reactions, as well as pain, fever, and even mortality [17,21,23]. The exact risk of incidence of nephrotoxic effects on patients receiving polymyxin treatments are highly variable across studies from 20 to 76% of patients, while a meta-analysis of two large studies suggests that colistin does not show significantly more cases of nephrotoxicity than other antimicrobials [21]. The older literature indicates severe events of neurotoxicity, usually associated with patients with cystic fibrosis, while newer reports suggest zero or low cases of neurotoxic effects [21].

While the safety concerns surrounding polymyxins led to their discontinued clinical use in favour of less toxic antimicrobials in the 1970s, they have made a return to clinical use for treating MDR, particularly carbapenem-resistant, Gram-negative infections [16,24,25,26,27]. Polymyxin B and E (colistin) are the polymyxin analogues used in clinical practice, and are used as a ‘last-line’ of defence for MDR Gram-negative infections; they are present in the WHO’s list of medically important antimicrobials under a “sole, or one of limited available therapies” for certain infections in people [11,16,25,27,28,29]. As such, research into polymyxin analogues with reduced toxicity is underway [11]. While polymyxin B and E have been used clinically, at least 15 naturally occurring unique polymyxins have been identified [17]. There are limited studies focussing on polymyxin A. The first record of a BGC encoding this analogue was reported in 2009 and highlights antimicrobial activity, showing that polymyxin A_2_ was slightly more active than polymyxin E or B against MDR clinical isolates and less toxic to immune cells (THP-1) [17,30]. Polymyxin A_2_ was also less toxic to kidney cells (HEK293) than polymyxin B, but showed similar toxicity to polymyxin E [30]. The most important factor to note here is the significant differences in the effects of polymyxin A_2_ versus polymyxin A_1_, as polymyxin A_1_ shows the highest toxicity to HEK293 cells [30]. The difference between A_1_ and A_2_ is the fatty acid chain shortening from a 6-methyloctanoic acid to a 6-methylheptanoic acid [30].

Despite toxicity concerns, increased cases of MDR infections have required the use of colistin as a last line of defence [16,22,28,31,32]. Due to their heavy usage in agriculture since the 1950s, antimicrobial resistance to polymyxins is widespread and of serious concern [16,22,28,31,33]. Resistance to colistin is most common through chromosomal mutations modifying the bacterial outer membrane, the lipopolysaccharide (LPS) layer, and in particular modifications of lipid A [28,31,34,35,36]. Mutations of *prmAB*, *prmC*, *pmrE*, *mgrB*, *pmrHFIJKLM*, and *phoPQ* are reported to cause resistance to polymyxins by modifying lipid A or by reducing the negative charge of the LPS, which alters the way colistin interacts with the bacterial outer membrane [28,34]. Efflux pumps are also recognised for the role they play in colistin resistance; for example, the *acrAB* and *kpnE* activates efflux pump systems, leading to colistin resistance [28,34].

Of particular concern for colistin resistance is mobile resistance genes: *mcr* genes are carried on plasmids and are spread through bacterial populations via horizontal gene transfer [28,33,36,37,38]. This means that these resistance genes can be spread quickly over wide geographical areas and across species, making them a serious concern for eliminating the effectiveness of colistin as a last-resort treatment [28,33,36,37,38]. *mcr* genes work by reducing the negative charge of the LPS through the modification of lipid A [33,36]. The first *mcr* gene (*mcr-1*) was reported in 2016 from *E. coli* isolated from a pig in China; since this report, a total of ten variants of *mcr* genes have been identified in animals, humans, and the environment across widespread geographical locations [28,33,34,35,36,39].

In this work, we report the isolation, genome sequencing, and characterisation of the biosynthetic gene cluster and cognate natural product (a polymyxin A-like compound) from a strain of *P. polymyxa* and determine its antimicrobial activity against a range of Enterobacterales containing a diverse array of resistance genes.

## 2. Results

**Figure 1:**
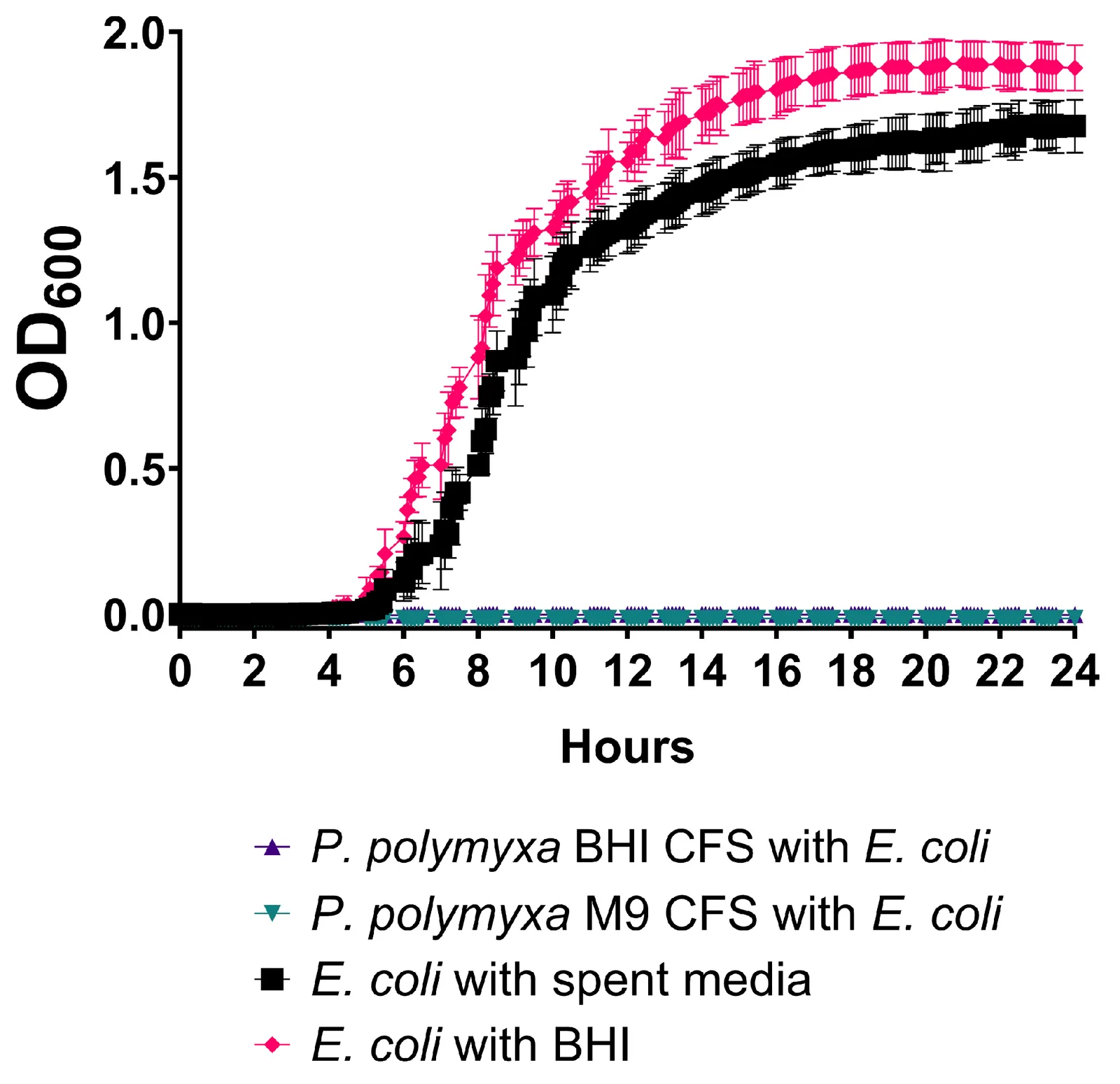
*E. coli* growth curves showing the inhibitory effects of *P. polymyxa* 1G CFS. The growth of *E. coli* when grown in the presence of the CFS from our isolate 1G versus with BHI broth only (control 1) or with CFS from the *E. coli* overnight spent media (control 2). Control 1 shows the normal growth of *E. coli* in nutrient rich broth; control 2 accounts for the loss of nutrients when an isolate is grown in media overnight. Our isolate 1G was grown in BHI and M9 media. OD_600_ = absorbance at 600 nm. N = 3 biological replicates (each three technical replicates). Error bars represent standard deviation.

The *P. polymyxa* 1G strain was isolated from a swab of a coal scuttle in 2019 from the Swab and Send project [40]. The isolate showed a zone of inhibition against the susceptible *E. coli* strain NCTC86 on agar screening, and the follow-up CFS assay showed a 99% reduction in area under the curve when compared to the growth of *E. coli* in spent media, as shown in Figure 1. The almost total inhibition of growth of *E. coli* led to species identification of the *P. polymyxa* 1G isolate, with the closest match of the 16S rRNA sequence being to *Paenibacillus polymyxa*. The isolate was whole-genome sequenced using Illumina and Oxford Nanopore sequencing. This resulted in a complete closed genome of 6,088,001 bp as one chromosome and a coverage of 33.8x. CheckM used the Paenibacillaceae marker set to derive a completeness of 99.85% and contamination of 0.02%. Using the whole-genome sequence, we identified the closest match as *P. polymyxa* B (GCF_001719045.1), with an ANI of 96.0% across 89.0% of the aligned genome, using GTDB-Tk. Using JSpeciesWS, the three *P. polymyxa*-type strains, DSM36, ATCC 842, and NCTC 10343, had ANIb values of 89.2% across aligned values of 79.04–79.20%. The closest matches were *P. polymyxa* E681 with an ANIb of 95.80% across 78.54% of the aligned genome and *P. polymyxa* J with an ANIb of 95.47% across 82.03% of the genome.

**Figure 2:**
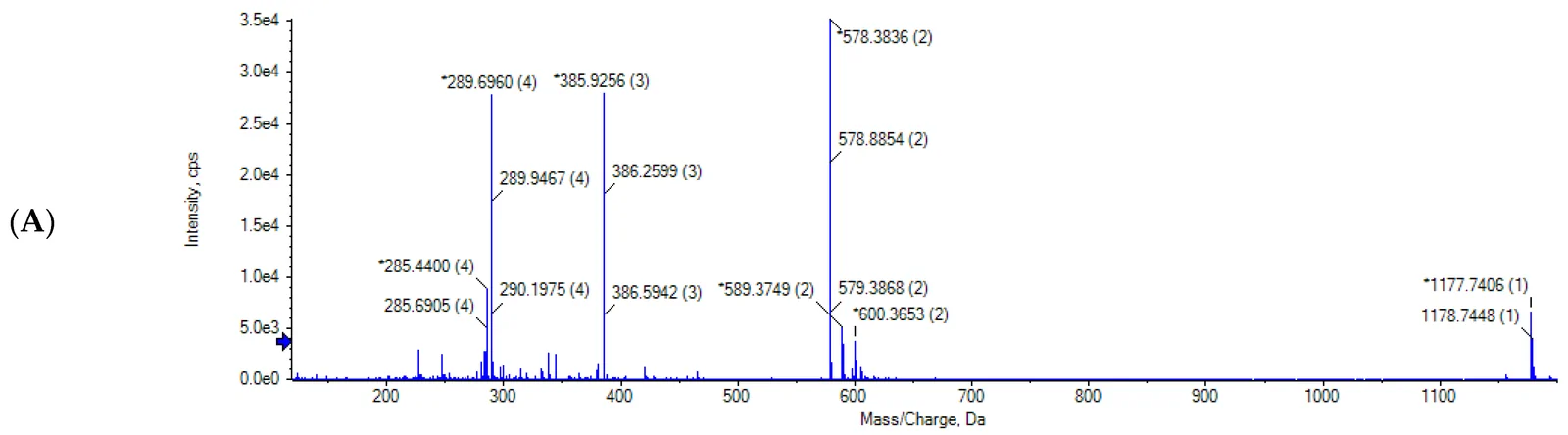

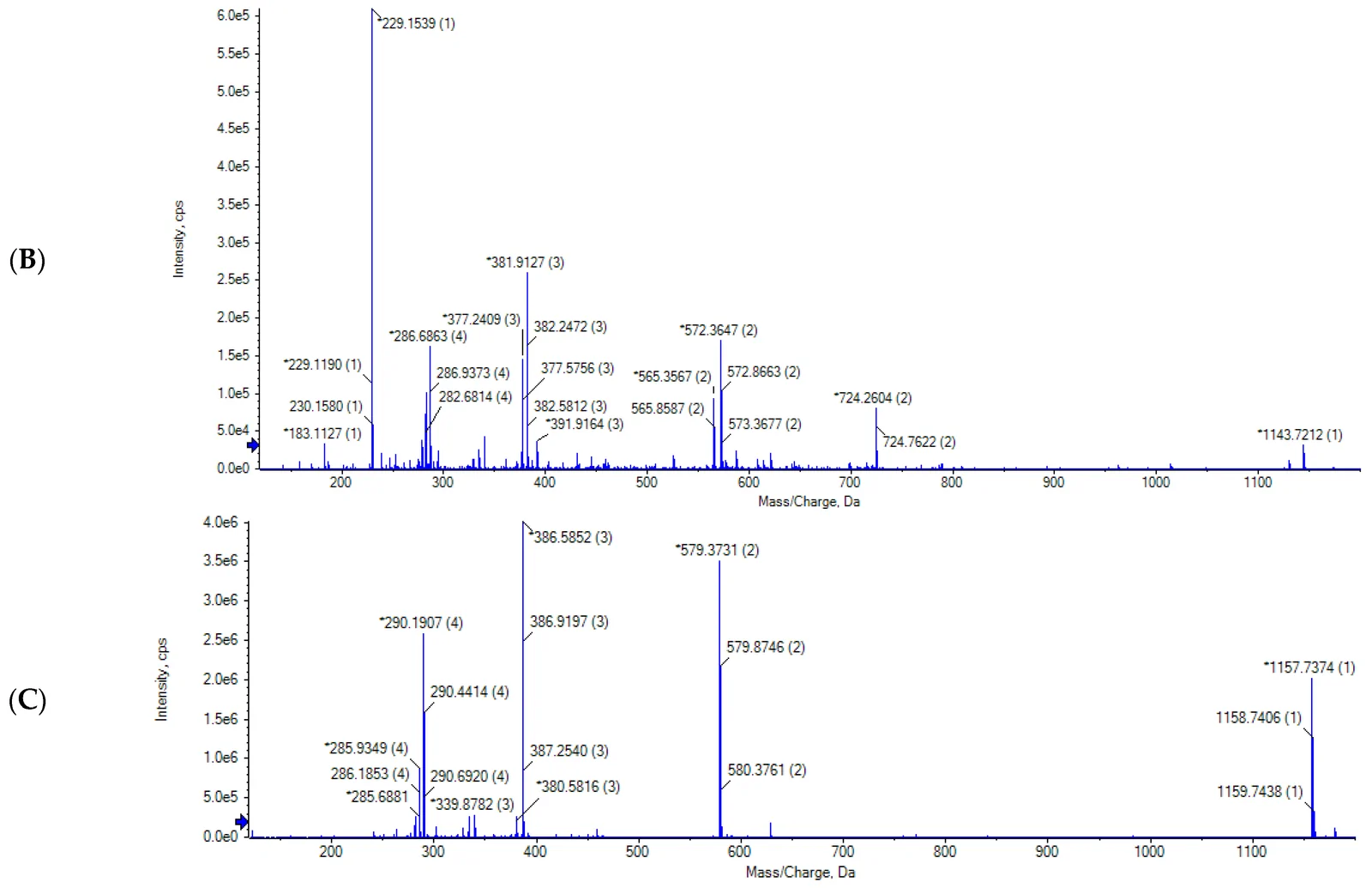
Mass spectrometry spectra summarising masses detected at specific peaks of LC-MS elution, where the asterisk (*) denotes if the *m*/*z* is monoisotopic. Panel (**A**) is the colistin sulphate peak eluting at 3.576 min. Panel (**B**) is the *P. polymyxa* 1G CFS peak eluting at 3.324 min. Panel (**C**) is the *P. polymyxa* 1G CFS peak eluting at 3.483 min.

A simpler growth media background was required for downstream data processing using mass spectrometry, so the isolate was grown in M9 using glucose as the carbon source. The *P. polymyxa* 1G isolate was grown in M9 media for 2 weeks, and retention of activity was confirmed by comparing the activity of BHI broth CFS to M9 broth CFS causing a 99% and 98% reduction in area under the curve, respectively (Figure 1). The compounds produced by the *P. polymyxa* 1G strain showed a similar pattern of mass ion peaks to colistin. Colistin sulphate bought from Apollo Scientific was used as a positive polymyxin control for mass spectrometry. The comparison between mass spectrometry spectra for the two peaks within our sample and the commercial colistin sulphate sample can be seen in Figure 2.

Colistin sulphate eluted at 3.576 min, while our isolate CFS had a small elution peak at 3.324 min, followed by a large peak eluting at 3.483 min (Supplementary Figure S1), both of which appear to have similar ion-charge M^1+^, M^2+^, M^3+^, and M^4+^ patterns to colistin in the mass spectrum (Figure 2). The molecular ions from the peaks eluting at 3.483 and 3.324 min were 1157.74 [M+H]^+^ and 1143.72 [M+H]^+^, respectively. In our experiment, the polymyxin E (colistin) molecular ion showed an *m*/*z* value of 1177.74 [M+Na]^+^, but has also been reported as the molecular ion of 1155.74 [M+H]^+^ [41,42]. While the ion charge patterns were similar to known polymyxin E, the difference in mass suggests that our isolate did not produce polymyxin E. The literature shows a close match to our two peaks with two compounds *m*/*z* values of 1143.69 [M+H]^+^ and 1157.69 [M+H]^+^ [30]. These two compounds were identified as polymyxin A variants A_1_ and A_2_, with a difference of 14 Daltons corresponding to a methylene (CH_2_) group. Our analogues have the same difference between our two peaks *m*/*z* values, equating to a longer N-terminal fatty acid chain [30]. We therefore predict that our isolate is likely producing these two variants of polymyxin A.

**Figure 3:**
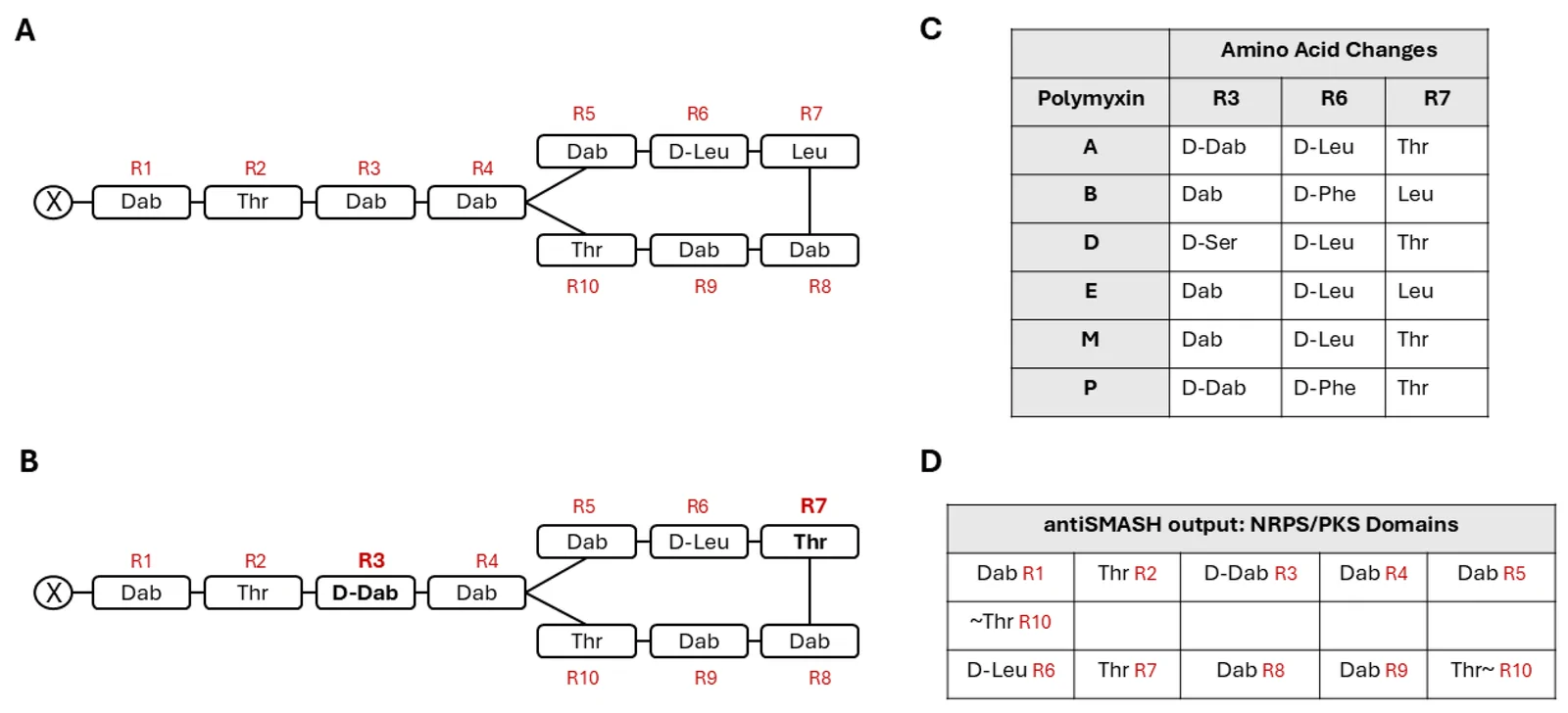
The amino acid structures and regions (R) of (**A**) colistin/polymyxin E [43], (**B**) the polymyxin amino acid structure of our isolate, (**C**) a table of the amino acid changes at the respective regions for the different polymyxin analogues (adapted from [11]), and (**D**) the antiSMASH output for the NRPS/PKS domains used to show the amino acid structure of our predicted compound.

To qualify the prediction of the polymyxin produced by our isolate being polymyxin A, the WGS was obtained and run through antiSMASH to investigate the predicted BGCs encoded by our isolate. One BGC showed a high confidence match to polymyxin, among several other high-confidence matches (Supplementary Table S1) within the genome, including clusters related to other bioactive metabolites. Therefore, the inhibitory activity of the crude CFS cannot be assigned exclusively to polymyxin A, and this is a limitation of the study. One output from antiSMASH is a prediction of the amino acids which are incorporated into a final compound produced by non-ribosomal peptide synthetase (NRPS). Each NRPS domain is responsible for addition of a single amino acid into the final compound. The predicted amino acids forming the encoded polymyxin were extracted from the antiSMASH output and compared to the expected amino acids for each of the other previously characterised polymyxin analogues (Figure 3). This figure shows that the predicted polymyxin-like compound produced by our isolate matches polymyxin A. Both our encoded polymyxin A – like compound and polymyxin A had the same three amino acid differences from polymyxin E in region 3 (R3), 6 (R6), and 7 (R7), as well as the same two amino acid differences from the other polymyxin variants in R6 and R7 (Supplementary Table S2).

**Figure 4:**
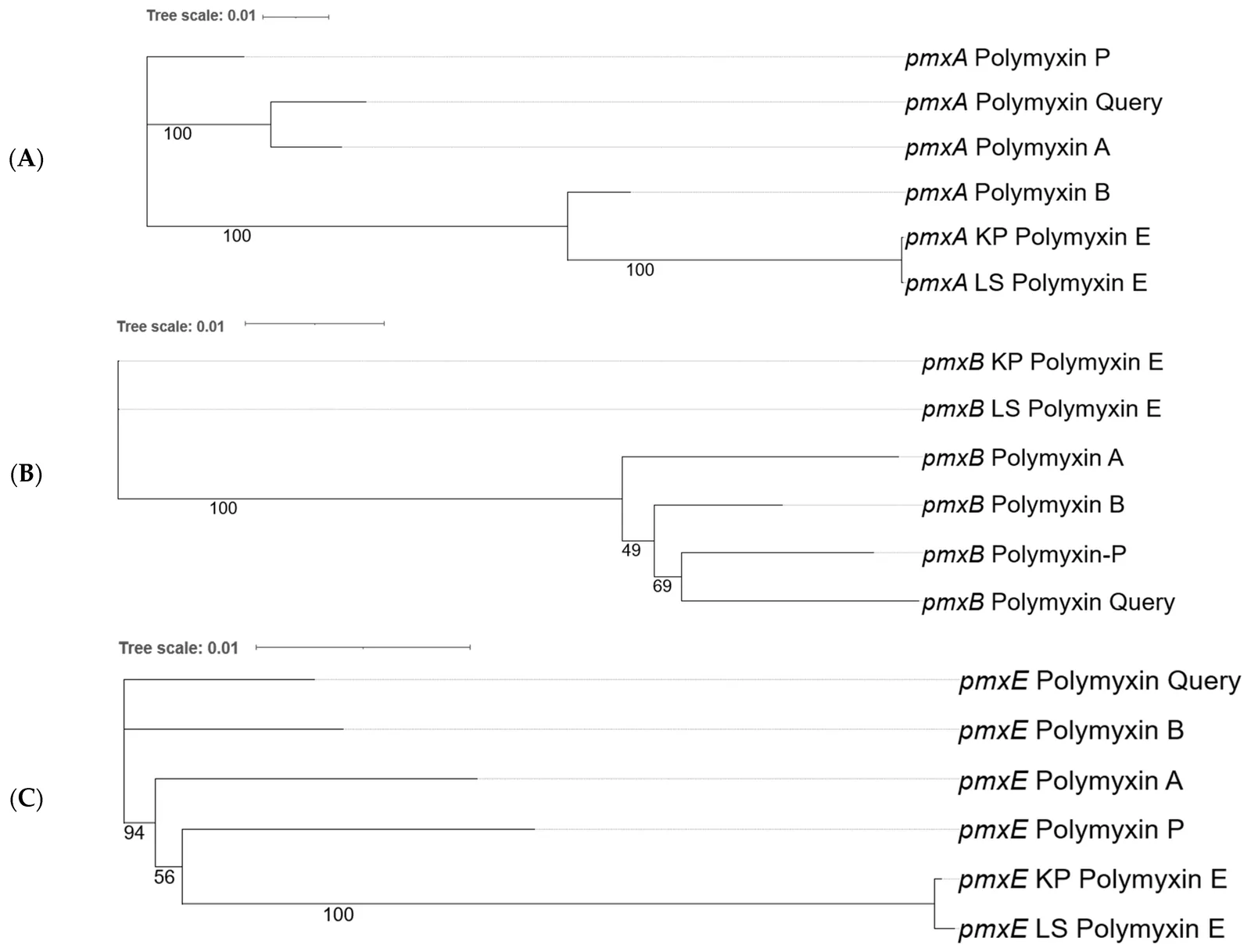
The phylogenetic trees compare the three *pmx* genes (**A**) *pmxA*, (**B**) *pmxB*, and (**C**) *pmxE* for all polymyxin analogue protein sequences when the polymyxin analogue is also known. Important note: the polymyxin B aligned here is for a variant of polymyxin B that has a D-Dab at region 3 instead of the expected L-Dab. IQTree was used to generate the phylogenetic trees using 1000 bootstraps.

Polymyxin biosynthesis is encoded for by three NRPSs (*pmxA*, *pmxB*, and *pmxE*) and two ABC-type transporters. Each *pmx* NRPS gene from our isolate was aligned with the other polymyxin analogue *pmx* NRPS genes (Supplementary Figure S2), using ClustalOMEGA to determine the alignment. IQTree was used to calculate the phylogenetic trees of the *pmxA* genes for all polymyxin analogues, as shown in Figure 4, alongside the same procedure for *pmxB* and *pmxE*.

The three *pmx* genes encode different regions of the polymyxin; *pmxE* encodes regions 1–5, *pmxA* encodes regions 6–9, and *pmxB* encodes region 10 (Figure 3). As *pmxB* only encodes for a threonine at region 10, for all known polymyxins with associated gene sequences, significant differences in the sequence would not be expected, although the grouping of both polymyxin E analogues would be expected as both sequences are from *P. alvei* instead of *P. polymyxa.* While *pmxE* encodes the first five regions, for the four characterised analogues, only polymyxin E differs in containing an L-Dab (2,4-diaminobutyric acid) instead of a D-Dab in region 3; thus, we would expect clustering of the polymyxin E encoding *pmxE* genes separate from the other polymyxin analogue *pmxE* genes. An important note is that, while normally we would also expect this difference for polymyxin B, the characterised variant encodes a D-Dab at region 3, removing this expected difference between polymyxin B and polymyxins A and P.

The most important alignment for distinguishing the polymyxin analogues is the *pmxA* gene due to it encoding both region 6 and 7, which are the variable regions for each polymyxin analogue (see Figure 3 and Figure 4). Both *pmxA* genes encoding polymyxin E cluster together, potentially due to them being from a different *Paenibacillus* species, but also due to them encoding D-leucine and L-leucine at regions 6 and 7, respectively. Closely clustering to the polymyxin E *pmxA* is polymyxin B’s *pmxA*, encoding the same amino acid at region 7 but D-phenylalanine at region 6 instead. While the polymyxin P *pmxA* gene clusters more closely with the polymyxin A *pmxA* gene, this is likely due to region 7 encoding leucine in polymyxin B and E versus threonine in polymyxin P and A. The higher weighting in relatedness for changes at region 7 versus region 6 is further explained by the increased variation in the active site residues of region 7 (Supplementary Table S2). The clustering of our query polymyxin sequence with polymyxin A and the conserved active site residues at every region between these two sequences supports the identification of *P. polymyxa* 1G’s polymyxin as a polymyxin A-like compound.

**Figure 5:**
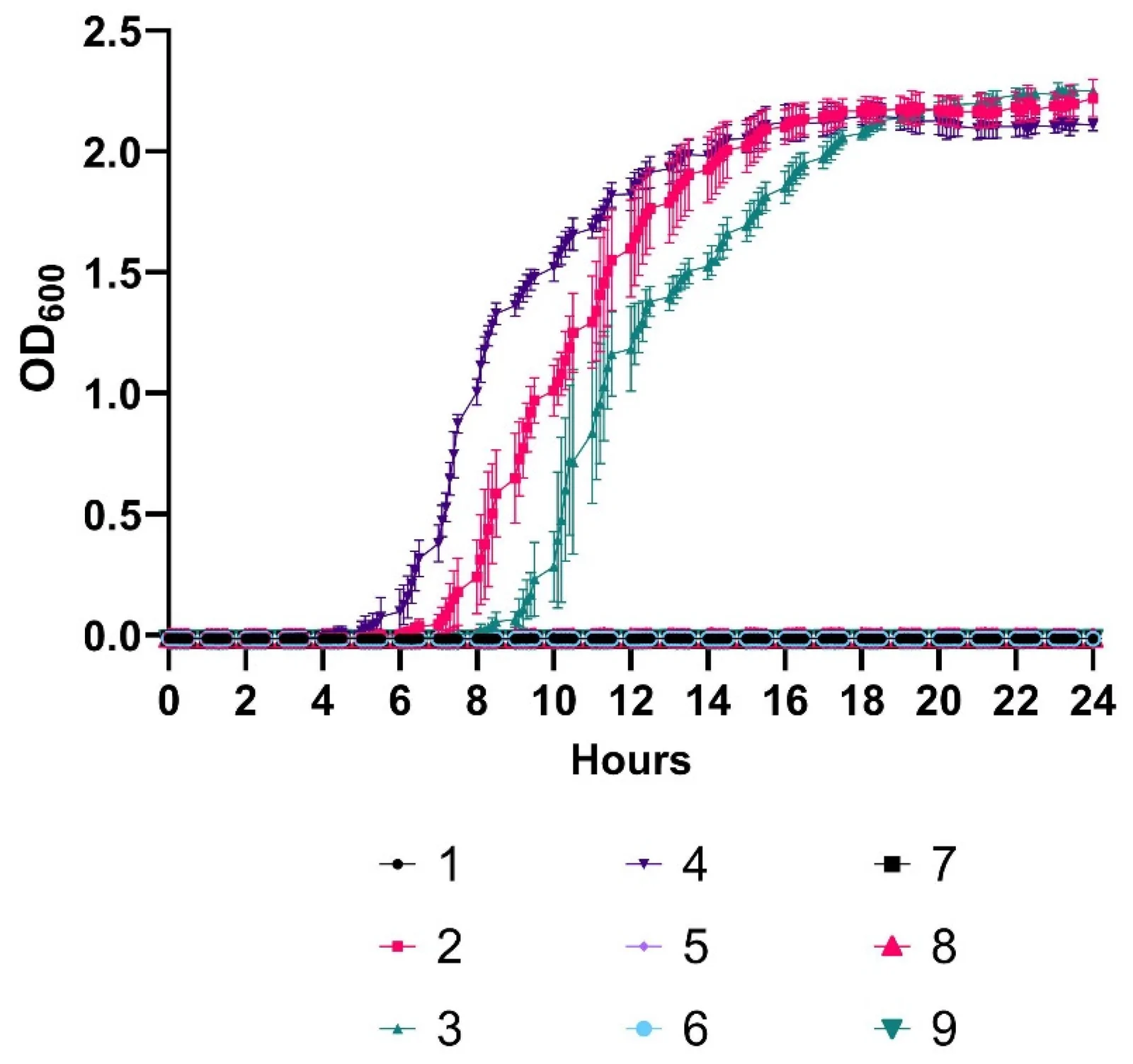
The growth of all nine RP isolates in the presence of CFS from *P. polymyxa* 1G grown in M9, which has mass spectrum data to support the presence of polymyxin. Here, we see resistance to our CFS containing the putative polymyxin A from isolates 2 and 3 containing *mcr*-1 and *mcr*-4, respectively. Resistance from RP isolate 4 has supporting biological data to show low-level resistance to colistin (Supplementary Figure S3). N = 3 biological replicates (each three technical replicates). Error bars represent standard deviation.

We tested eight resistant isolates against the CFS of *P. polymyxa* 1G strain and found that the isolates with *mcr*-resistance genes (RP 2 and RP 3) were resistant to polymyxin A, as expected, and isolate RP 4 was also resistant (Figure 5). Resistance from isolate RP 4 was unexpected, as there are no polymyxin-resistance genes or chromosomal mutations. A follow-up experiment was run to test resistance to colistin sulphate salt using *E. coli* NCTC 86 (RP 1) as a negative control and RP 2 and RP 3 as positive controls (Supplementary Figure S3). Both *E. coli* isolates containing *mcr*-resistance genes are resistant to the highest tested concentration of colistin 4 mg/L surpassing the clinical breakpoint for colistin resistance at 2 mg/L. *E. coli* NCTC 86 was able to grow normally in the presence of colistin up to 0.5 mg/L, while isolate RP 4 was able to grow relatively well in the presence of colistin at 1 mg/L. The susceptibility of *E. coli* strains is shown to be the same across polymyxin E and A [30], and so this data suggests that the growth of RP 4 in the presence of *P. polymyxa* 1G CFS versus the lack of growth of *E. coli* NCTC 86 may be because this isolate produces our putative polymyxin A at similar levels.

## 3. Discussion

In this study, we present mass spectrum data for our isolate’s antimicrobial CFS containing two compounds with *m*/*z* values of 1143.7212 and 1157.7374, which show very similar *m*/*z* values to two polymyxin A variants characterised by Jangra et al., 2018, being 1143.69 and 1157.69 [30]. Jangra et al., 2018, showed that the 14 Da difference between the compounds was a difference in the N-terminal fatty acid chain length, from 6-methyloctanoic acid in polymyxin A_1_ (1157 *m*/*z*) to 6-methylheptanoic acid in polymyxin A_2_ (1143 *m*/*z*) [30]. So, rather than two distinct antimicrobial compounds being produced by *P. polymyxa* 1G, we are likely seeing the two variants of polymyxin A, as Jangra et al., 2018, described [30]. This is supported by sequence data from our isolate *P. polymyxa* 1G showing that the predicted amino acid structure (Figure 3) and active site residues match (Supplementary Table S2) between our sequence and the sequence that encodes polymyxin A. We also show the alignment of our query sequence against all five polymyxin BGCs. As the differences for polymyxin A compared to polymyxin B, E, and P are most significant at regions 6 and 7, encoded for by *pmxA*, the clustering of our sequence with polymyxin A encoding *pmxA* for this NRPS also indicates that our isolate produces polymyxin A compounds.

Clinically used polymyxin variants are restricted to polymyxin E and polymyxin B due to them having the lowest nephrotoxicity amongst tested analogues in in vivo models in the 1960s [22]. However, more recent research suggests that polymyxin A_2_ has lower toxicity to an immune cell line, similar toxicity to a kidney cell line, and better efficacy than polymyxin B and E against MDR clinical bacterial isolates [30]. Jangra et al., 2018, suggested that further research was need into polymyxin A’s potential clinical use, prompting us to test the putative polymyxin A compounds from this *P. polymyxa* 1G strain against eight clinical isolates with various resistance gene profiles and a pan-susceptible *E. coli* NCTC 86 strain [30]. As polymyxin is a last line of defence antibiotic important for treating Gram-negative infections, and colistin resistance is particularly widespread in *E. coli* and *Klebsiella* spp. infections, a selection of clinical isolates were chosen to test against [37,38,39]. Two of the resistance panel isolates, RP 2 and RP 3, contained *mcr* genes that confer resistance to polymyxins by modifying lipid A in the outer membrane lipopolysaccharides [33]. *mcr-1*, which is present in RP 2, is globally widespread [33], and we have descriptive data to show that it confers resistance to our putative polymyxin A. *mcr-4*, present in the RP 3, is a less common *mcr* gene that we show can also confer resistance to our putative polymyxin A [33].

Lastly, RP 4 showed reduced susceptibility to the CFS of *P. polymyxa* 1G, but had no known resistance genotype to polymyxins. The biological data collected in this study showed over 50% of expected growth in 1 mg/L colistin compared to only 5% growth of the *E. coli* NCTC 86 indicator strain (Supplementary Figure S3). Jangra et al., 2018, show that there are negligible, if any, differences between the MICs of the different polymyxin variants A, B, and E against *E. coli* and *Klebsiella* spp. Strains [30]. Combined with our data, this suggests that our strain could be producing polymyxin-like compounds at a relatively low concentration, allowing us to see growth at just below the colistin EUCAST breakpoint of 2 mg/L in RP 4 but not in the pan-susceptible *E. coli* NCTC 86 strain. An alternative and not mutually exclusive explanation is that there is a novel polymyxin resistance mechanism, and further work is ongoing to investigate this possibility.

## 4. Materials and Methods

### 4.1. Bacterial Growth Conditions

All bacterial isolates were grown on brain–heart infusion (BHI) agar at 37 °C for 18 to 24 h before growth in liquid media. Our *Paenibacillus polymyxa* 1G strain (NCBI accession number: JBVPZV000000000) was grown in brain–heart infusion (BHI) or M9 glucose media at room temperature. For 500 mL M9, glucose it contained 1× of the M9 salts in 0.5 L of water plus 500 µL of MgSO4 (1 M), 500 µL CaCl2 (0.1 M), and 8 mL of 40% (*w*/*v*) glucose. *P. polymyxa* 1G was grown at room temperature, while the resistance panel strains were incubated at 37 °C. All growth curves were performed in a CLARIOstar plate reader (BMG Labtech, Aylesbury, UK) at 37 °C with orbital shaking at 200 rpm between readings, for 24 h, reading at OD600 using 100 flash every 10 min.

### 4.2. Resistance Panel for Antimicrobial Susceptibility Testing

Eight clinical isolates were selected due to their differing resistance mechanisms for testing, alongside a pan-susceptible *E. coli* NCTC 86 (RP1). Two of the resistance panel isolates were an *E. coli* containing *mcr-1* (RP 2) and another *E. coli* isolate containing *mcr-4* (RP 3). Isolates are referred to by their strain number, as listed in Table 1, with the associated resistance mechanisms described. Bioinformatic analysis was run on the whole genome sequences (WGS’s) to establish their resistance mechanisms. WGSs were run through ResFinder and PointFinder with the following settings: Chromosomal point mutations: threshold for ID: 90.0%; minimum length: 60.0%; show unknown mutations: False; ignore premature stop codons: True; ignore frameshift indels: True. Acquired antimicrobial resistance genes: threshold for ID: 90.0%; minimum length: 60.0%. Specified species and input data type as either *E. coli* or *Klebsiella*. Database versions: ResFinder-2.5., PointFinder-4.1.1.

### 4.3. On-Agar Inhibition Assay

*P. polymyxa* 1G was tested for antimicrobial activity against *Escherichia coli* NCTC 86. Colonies of *E. coli* were added to BHI broth and the OD_600_ corrected to 0.15. A sterile swab was used to spread the diluted *E. coli* culture across a BHI agar plate (4% (*w*/*v*) agar) to make a background lawn of microbial growth. A spot of *P. polymyxa* 1G was added onto the lawn, and the agar plate was incubated (2 days, room temperature) and the zone of inhibition was assessed.

### 4.4. Cell-Free Supernatant Inhibition Assay

After *P. polymyxa* 1G showed a zone of inhibition on agar, it was screened in a CFS assay against either *E. coli* or the resistance panel strains. *P. polymyxa* 1G was grown for 18 h (10 mL BHI) for 2 weeks (10 mL, M9 glucose) at room temperature). *P. polymyxa* did not grow as well in M9 medium, which is why it was incubated for two weeks to accumulate as much of our putative polymyxin as possible. In total, 1 mL aliquots of the cultures were centrifuged at 13,000× *g* for 1 min, and the supernatant was filtered (0.22 µm) to produce the CFS. Each well of a 96-well plate (Costar, New York, NY, USA) had either CFS added (100 µL) or was a control well containing CFS from the *E. coli* or the resistance panel strains (100 µL). This was followed by the addition of diluted cultures of the test strains (1:5000 dilution of an OD_600_ = 0.1 culture of an *E. coli*, or one of the resistance panel strains, overnight cultures, all into fresh BHI). Positive control wells had 100 µL BHI and 100 µL diluted *E. coli* or resistance panel strain, and negative control wells had 200 µL BHI. Growth was measured for 24 h using a CLARIOstar plate reader (BMG Labtech, Aylesbury, UK), as described above. Three biological replicates (each three technical replicates) were performed.

The resulting data was analysed using GraphPad Prism (version 10.4.0 for Windows, GraphPad Software, www.graphpad.com, accessed on 10 August 2026) software to calculate the area under the curve. GraphPad Prism uses the trapezoid rule to calculate the area under the curve, using ΔX × ([(Y1 + Y2)/2] − Baseline) to calculate the area between each pair of adjacent points on the growth curve before summing to obtain the total area under the curve. The reduction in area under the curve is calculated by comparing the CFS from *E. coli* or resistance panel strain, respectively, with *E. coli* growth in each environmental isolates’ CFS. Growth in the presence of colistin sulphate (Apollo Scientific, Manchester, UK) was assayed using the same protocol for the resistance panel (strains 1–4), except for colistin diluted in molecular water replaced CFS in each well of the 96-well plate.

### 4.5. Isolate Identification and Sequencing

Strains were grown for two days (room temperature on BHI agar). Colony PCR used an individual colony of each isolate, MyTaq 2x Master Mix, and the following primers (27F—AGAGTTTGATCCTGGCGCAG and 1492R—GGTTACCTTGTTACGACTT) with PCR conditions: 95 °C, 10 min; 35 cycles (95 °C, 1 min; 50 °C, 30 s; 72 °C, 30 s) and a final 72 °C, 5 min. PCR cleanup was performed using the Monarch^®^ Spin PCR & DNA Cleanup Kit (New England Biolabs, Ipswich, MA, USA). The purified DNA was then sent to GeneWiz (Takeley, UK) for Sanger sequencing. The resulting sequence was matched to other 16S rRNA sequences using BLASTn against the core nucleotide database to identify the closest species matches [44].

*P. polymyxa* 1G was also sent for whole-genome sequencing. MicrobesNG (MicrobesNG, Birmingham, UK) performed short-read sequencing using HiSeq X10 (Illumina, San Diego, CA, USA). Genomic DNA was extracted using a Fire Monkey High Molecular Weight DNA Extraction Kit (RevoluGen, Berkshire, UK), and long-read sequencing was carried out using Oxford Nanopore Technologies (Oxford, UK) R9.4.1 flow cell on a MinION sequencer. The reads were basecalled with MinKNOW software using the Guppy algorithm (version 4.0.9). Long-reads were trimmed using Porechop (https://github.com/rrwick/Porechop, accessed on 10 August 2026) and filtered using Filtlong (https://github.com/rrwick/Filtlong, accessed on 10 August 2026), and the genome assembly was performed using a Unicycler (version 0.4.8.0) with default parameters [45]. The assembly yielded a genome sequence of 6,088,001 bp with a G+C content of 45.7%. The genome was annotated using NCBI’s Prokaryotic Genome Annotation Pipeline (PGAP) (version 6.10) [46]. CheckM (version 1.1.2) was used with the lineage_wf workflow and the Paenibacillaceae marker set [47].

Following whole-genome sequencing, for additional evidence of species identification, the genome was searched for the closest type-strain using GTDB-Tk (version 2.1.1), using the classify_wf workflow for species assignment [48]. The genome was also assessed using the JSpeciesWS webserver (version 5.0.3), using ANIb to compare the *P. polymyxa* 1G genome to other *Paenibacillus* spp. genomes [49].

### 4.6. Bioinformatics Analysis of P. polymyxa 1G Whole Genome Sequence

The assembled *P. polymyxa* 1G genome was run through antiSMASH (version 8.0) to look for known BGCs [50]. The polymyxin BGC was focussed upon, and the *pmxA*, *pmxB*, and *pmxE* genes encoding the biosynthetic proteins were analysed in more detail. Available *pmx* genes associated with characterised polymyxin variants were aligned with our isolate’s genes using ClustalOMEGA multiple sequence alignment [43] on the EMBL-EBI job dispatcher [51]. The phylogenetic trees were created using IQTree (version 1.6.1) using 1000 bootstraps, with ModelFinder selecting model TN+F+I for *pmxA* and *pmxB* and TIM2+F+I for *pmxE*, and the consensus trees were visualised using iTOL (version 7.2) [52,53,54]. Alignment sequence Genbank accession numbers were as follows: polymyxin A from *P. polymyxa* (EU371992.1) [17], a polymyxin B variant with D-dab at region 3 from *P. polymyxa* (JN660148.1) [19], polymyxin E from *P. alvei* (LS992241.1) [20], polymyxin E from *P. alvei* (KP262070.1) [20], and polymyxin P from *P. polymyxa* (HE577054.1) [18]. The two polymyxin Es are differentiated using the first two letters of their accession number in the following text.

Supplementary Table S2 shows the active site residues for the polymyxin variants and was created by extracting active site residues from antiSMASH (v8.0) using the Stachelhaus sequence. The stereochemical configuration of each amino acid was predicted from the antiSMASH NRPS/PKS module view, with the presence of an epimerisation domain indicating the D-stereoisomer of an amino acid.

### 4.7. Mass Spectrometry

The *P. polymyxa* 1G isolate was cultured in M9 glucose for two weeks (room temperature). The samples were mixed with acetonitrile (100 µL + 100 µL) to remove protein and were centrifuged at 20,000× *g* for 3 min at 4 °C. The supernatant was then dried under nitrogen gas flow at 40 °C and resuspended in 100 µL 5% acetonitrile in water.

Liquid chromatography–mass spectrometry (LC-MS) analysis was performed using a SCIEX Exion LC system consisting of two AD high-pressure gradient pumps, vacuum degasser, solvent valve, AC column oven, and AC Autosampler, coupled to a SCIEX 7600 ZenoTOF Q-TOF mass spectrometer (AB Sciex LLC, Marlborough, MA, USA) with a TurboV Optiflow ion source running a 50 µm ESI probe. The system was controlled using SCIEX OS (v3.0).

A sample volume of 10 μL was injected onto a 50 μL sample loop. The injection cycle time was 1 min per sample. Separations were performed using a Thermo Accucore C18 column (Thermo Fisher Scientific Inc., Waltham, MA, USA) of 150 mm length, 2.1 mm diameter, and 2.6 μm particle size equipped with a guard column of the same phase. Mobile phase A was water with 0.1% formic acid; mobile phase B was 98% acetonitrile and 2% water with 0.1% formic acid. Separation was performed by gradient chromatography at a flow rate of 0.3 mL/min, starting at 5% B for 1 min, ramping to 100% B over 7 min, holding at 100% B for 2 min, then returning to 5% B. Re-equilibration time was 4 min. Total run time including injection cycle was 15 min.

The mass spectrometer was run in positive mode under the following source conditions: curtain gas pressure, 50 psi; ion spray voltage, 5500 V; temperature, 400 °C; ESI nebulizer gas pressure, 50 psi; heater gas pressure, 70 psi; declustering potential, 30 V. Mass spectrum data was acquired in the mass range of 120–1200 *m*/*z* with an accumulation time of 0.25 s. Declustering potential was 50 V and collision energy was 10 V. Colistin was analysed by measuring the peak areas of the [M+2H]^2+^ and [M+3H]^3+^ ions at *m*/*z* 578.383 and 385.924 ± 0.05, respectively, with the [M+3H]^3+^ ion acting as the quantifier and the [M+2H]^2+^ ion acting as the qualifier. The retention time of colistin was determined to be 3.48 min. Polymyxin A_1_ and A_2_ were identified as the [M+H]^+^ ions at *m*/*z* 1157.74 and 1143.72, eluting at 3.483 and 3.324 min from the *P. polymyxa* 1G CFS.

The metabolomics data have been deposited in the MetaboLights repository with the study identifier MTBLS15006 [55].

## 5. Conclusions

*P. polymyxa* 1G identified from the Swab and Send project has been shown here to produce polymyxin A-like compounds with mass spectrum data and genetic data matching the predicted amino acid structure of polymyxin A. Sequence alignments of the *pmx* genes encoding the amino acids differences between polymyxin variants also align with polymyxin A. While previous toxicity data suggest that polymyxin A may be a good alternative to polymyxin E and B, we found that clinical colistin-resistance mechanisms in the strains we tested also confer resistance to our putative polymyxin A. Although it may have similar or less toxicity to clinically used polymyxins, it is unlikely to be an alternative for use against pathogens resistant to the last line-of-defence clinical polymyxins, at least in the strains tested within this study.

## Figures and Tables

**Table 1:** Strains used in the study and their resistance genes, including *E. coli* NCTC 86 strain and eight clinical isolates. Strains 1–7 are *E. coli* and strains 8 and 9 are *Klebsiella* spp. Strains in green grew in the presence of CFS from *P. polymyxa* 1G, while all of the other strains did not.

Strain Identifier		Resistance Genes					
	Colistin Resistance	Beta Lactam	Aminoglycosides	Quinolones	Cotrimoxazole	Others	Resistance Conferred by Chromosomal Mutations
RP 1 (*E. coli* NCTC 86)		-	-	-	-	-	-
RP 2	*mcr-1.1*	*blaCMY-2*	*ant(3* *″* *)-la*	-	*dfrA1*	*inu(F)*	-
		*blaTEM-1A*			*sul2*		
RP 3	*mcr-4.6*	*blaTEM-1A,*	*ant(3* *″* *)-la*	-	-		-
		*tet(M)*					
		*tet(A)*					
RP 4		*blaTEM-1B*	*aph(3* *″* *)-Ib*	-	*sul2*	*mph(A)*	-
			*aph(6)-Id*		*dfrA8*		
RP 5		*blaTEM-1B*	*aadA5*	-	*sul2*	*mph(A)*	Nalidixic Acid
		*blaCTX-M-15*	*aph(3* *″* *)-Ib*		*dfrA17*		Ciprofloxacin
			*aph(6)-Id*		*sul1*		
RP 6		*blaTEM-1B*	*aph(3* *″* *)-Ib*	-	*sul2*	-	-
			*aph(6)-Id*		*dfrA8*		
RP 7		-	-	-	*sul1*	-	-
					*dfrA7*		
RP 8		*blaSHV-164*	*aac(3)-lld*	*oqxA*	*sul2*	*fosA*	Fluoroquinolone
		*blaSHV-59*		*oqxB*	*dfrA30*		Cephalosporins
		*blaCTX-M-15*					Carbapenem
		*blaTEM-1B*					
RP 9		*blaSHV-110*	*aac(3)-lld*	*oqxA*	*sul2*	*fosA6*	Fluoroquinolone
		*blaSHV-81*		*oqxB*	*dfrA30*		Cephalosporins
		*blaCTX-M-15*					Carbapenem
		*blaTEM-1B*					Tigecycline

## Data Availability

The *Paenibacillus polymyxa* 1G strain sequence has been deposited in NCBI, accession number JBVPZV000000000. The raw data supporting the conclusions of this article will be made available by the authors on request.

## Supplementary Materials

The following supporting information can be downloaded at https://www.mdpi.com/article/10.3390/antibiotics15080816/s1. Table S1: antiSMASH output from *P. polymyxa* 1G; Table S2: active residue sites for polymyxin variants aligned with *P. polymyxa* 1G; Figure S1: chromatogram showing elution of commercial colistin and *P. polymyxa* 1G; Figure S2: Alignment of *pmxA*, *pmxB* and *pmxE*; Figure S3: comparative growth of resistance panel strains in the presence of commercial colistin.

## Abbreviations

The following abbreviations are used in this manuscript:

BGC	Biosynthetic gene cluster
BHI	Brain–heart infusion
CFS	Cell-free supernatant
MDR	Multi-drug resistant
MIC	Minimum inhibitory concentration
NRPS	Non-ribosomal peptide synthetase
