# Supplementary material for: Antimicrobial Activity of a Polymyxin A-like Compound and Characterisation of the Cognate Biosynthetic Gene Cluster Within the Genome of the Producing Paenibacillus polymyxa
Source: Antibiotics (Basel). 2026 Aug 21;15(8):816. doi: 10.3390/antibiotics15080816 (PMC13510159; doi:10.3390/antibiotics15080816)
Supplement: Supplementary file 1 [file antibiotics-15-00816-s001.zip › antibiotics-4384609-supplementary.pdf]

Supplementary Material for Antimicrobial activity of polymyxin A, and characterization of the cognate biosynthetic gene cluster within the genome of the producing *Paenibacillus polymyxa*.

Amy McLeman[1], Alex D. H. Kingdon[1], Robin Hoeven[2], George Taylor[2], Ellie Allman[1], Issra Bulgasim[1,3], Claudia McKeown[1], Richard N. Goodman[1], Sabrina Moyo[1,4], and Adam P. Roberts[1,#].

Supplementary Table S1 showing the output of antiSMASH 8.0 from running the whole genome sequence of *Paenibacilus polymyxa* 1G using relaxed detection strictness, and the extra features: Known Cluster Blast, TFBS analysis, Active Site Finder, Sub Cluster Blast and RREFinder.

Table S1: antiSMASH output from *P. polymyxa* 1G.

**Region**	**Type**	**From**	**To**	**Similarity confidence**	**Most similar known cluster**
**Region 1**	NRPS	67,893	131,619	**High**	fusaricidin B
**Region 2**	RRE-containing	379,655	401,109		
**Region 3**	NI-siderophore	1,031,093	1,066,494		
**Region 4**	Proteusin	1,216,382	1,236,618		
**Region 5**	NRPS,transAT-PKS	1,272,973	1,372,957		
**Region 6**	lassopeptide	1,408,291	1,432,389	**Low**	paeninodin
**Region 7**	lanthipeptide-class-i	1,776,601	1,803,608	**High**	paenilan
**Region 8**	NRPS-like	2,128,959	2,172,654		
**Region 9**	cyclic-lactone-autoinducer	2,183,714	2,204,357		
**Region 10**	NRPS	2,549,080	2,642,697	**High**	tridecaptin
**Region 11**	transAT-PKS,NRPS	2,731,709	2,801,719	**Low**	Pelgipeptin A/ B/ C/ D
**Region 12**	transAT-PKS,NRPS	2,816,149	2,898,822	**Medium**	paenilipoheptin
**Region 13**	cyclic-lactone-autoinducer	3,248,592	3,269,140		
**Region 14**	terpene-precursor	3,601,295	3,622,218		
**Region 15**	transAT-PKS,NRPS,T3PKS,PKS-like	3,788,534	3,890,613	**Low**	aurantinin B/ C/ D
**Region 16**	terpene-precursor	4,243,400	4,265,778		
**Region 17**	NRPS	5,216,214	5,297,097	**High**	polymyxin
**Region 18**	phosphonate	5,895,571	5,946,724		

Supplementary Table S2 showing the active site residues for the five polymyxin analogues and the polymyxin query sequence from *P. polymyxa* 1G. Highlights show the differences in active site residues relative to our isolate's Pmx proteins predicted active site residues.

Table S2: Active residue sites for polymyxin variants aligned with *P. polymyxa* 1G

Region	Accession number	Genbank accession	Polymyxin Analogue	Important active site residues in adenylation-domain										Specified amino acid
Region 1														
**pmxE1**	Polymyxin Query			D	V	G	E	I	S	S	I	D	K	L-Dab
	E681	EU371992.1	Polymyxin A	D	V	G	E	I	S	S	I	D	K	L-Dab
	M-1	HE577054.1	Polymyxin P	D	V	G	E	I	S	S	I	V	K	L-Dab
		KP262070.1	Polymyxin E	D	V	G	E	I	S	S	I	D	K	L-Dab
	B-LR	LS992241.1	Polymyxin E	D	V	G	E	I	S	S	I	D	K	L-Dab
	PKB1	JN660148.1	D-Dab3-Polymyxin B	D	V	W	E	I	S	S	I	D	K	L-Dab
Region 2														
**pmxE2**	Polymyxin Query			D	F	W	N	I	G	M	V	H	K	L-Thr
	E681	EU371992.1	Polymyxin A	D	F	W	N	I	G	M	V	H	K	L-Thr
	M-1	HE577054.1	Polymyxin P	D	F	W	N	I	G	M	V	H	K	L-Thr
		KP262070.1	Polymyxin E	D	F	W	N	I	G	M	V	H	K	L-Thr
	B-LR	LS992241.1	Polymyxin E	D	F	W	N	I	G	M	V	H	K	L-Thr
	PKB1	JN660148.1	D-Dab3-Polymyxin B	D	F	W	N	I	G	M	V	H	K	L-Thr
Region 3														
**pmxE3**	Polymyxin Query			D	V	G	E	I	S	S	I	D	K	D-Dab
	E681	EU371992.1	Polymyxin A	D	V	G	E	I	S	S	I	D	K	D-Dab
	M-1	HE577054.1	Polymyxin P	D	V	G	E	I	S	S	I	D	K	D-Dab
		KP262070.1	Polymyxin E	D	V	G	E	L	S	S	I	D	K	L-Dab
	B-LR	LS992241.1	Polymyxin E	D	V	G	E	L	S	S	I	D	K	L-Dab
	PKB1	JN660148.1	D-Dab3-Polymyxin B	D	V	G	E	I	S	S	I	D	K	D-Dab
Region 4														
**pmxE4**	Polymyxin Query			D	V	G	E	I	S	A	I	D	K	L-Dab
	E681	EU371992.1	Polymyxin A	D	V	G	E	I	S	A	I	D	K	L-Dab
	M-1	HE577054.1	Polymyxin P	D	V	G	E	I	S	A	I	D	K	L-Dab
		KP262070.1	Polymyxin E	D	V	G	E	I	S	A	I	D	K	L-Dab
	B-LR	LS992241.1	Polymyxin E	D	V	G	E	I	S	A	I	D	K	L-Dab
	PKB1	JN660148.1	D-Dab3-Polymyxin B	D	V	G	E	I	S	A	I	D	K	L-Dab
Region 5														
**pmxE5**	Polymyxin Query			D	V	G	E	I	S	A	I	D	K	L-Dab

	E681	EU371992.1	Polymyxin A	D	V	G	E	I	S	A	I	D	K	L-Dab
	M-1	HE577054.1	Polymyxin P	D	V	G	E	I	S	A	I	D	K	L-Dab
		KP262070.1	Polymyxin E	D	V	G	E	I	S	A	I	D	K	L-Dab
	B-LR	LS992241.1	Polymyxin E	D	V	G	E	I	S	A	I	D	K	L-Dab
	PKB1	JN660148.1	D-Dab3-Polymyxin B	D	V	G	E	I	S	A	I	D	K	L-Dab
Region 6														
**pmxA1**	Polymyxin Query			D	A	W	I	V	G	A	I	V	K	D-Leu
	E681	EU371992.1	Polymyxin A	D	A	W	I	V	G	A	I	V	K	D-Leu
	M-1	HE577054.1	Polymyxin P	D	A	W	T	I	A	A	I	A	K	D-Phe
		KP262070.1	Polymyxin E	D	A	W	I	V	G	A	I	V	K	D-Leu
	B-LR	LS992241.1	Polymyxin E	D	A	W	I	V	G	A	I	V	K	D-Leu
	PKB1	JN660148.1	D-Dab3-Polymyxin B	D	A	W	T	I	A	A	I	A	K	D-Phe
Region 7														
**pmxA2**	Polymyxin Query			D	F	W	N	I	G	M	V	H	K	L-Thr
	E681	EU371992.1	Polymyxin A	D	F	W	N	I	G	M	V	H	K	L-Thr
	M-1	HE577054.1	Polymyxin P	D	F	W	N	I	G	M	V	H	K	L-Thr
		KP262070.1	Polymyxin E	D	G	F	F	I	G	V	V	Y	K	L-Leu/Ile
	B-LR	LS992241.1	Polymyxin E	D	G	F	F	L	G	V	V	Y	K	L-Leu/Ile
	PKB1	JN660148.1	D-Dab3-Polymyxin B	D	G	F	L	L	G	L	V	Y	K	L-Leu
Region 8														
**pmxA3**	Polymyxin Query			D	V	G	E	I	S	A	I	D	K	L-Dab
	E681	EU371992.1	Polymyxin A	D	V	G	E	I	S	A	I	D	K	L-Dab
	M-1	HE577054.1	Polymyxin P	D	V	G	E	I	S	A	I	D	K	L-Dab
		KP262070.1	Polymyxin E	D	V	G	E	I	S	A	I	D	K	L-Dab
	B-LR	LS992241.1	Polymyxin E	D	V	G	E	I	S	A	I	D	K	L-Dab
	PKB1	JN660148.1	D-Dab3-Polymyxin B	D	V	G	E	I	S	A	I	D	K	L-Dab
Region 9														
**pmxA4**	Polymyxin Query			D	V	G	E	I	S	A	I	D	K	L-Dab
	E681	EU371992.1	Polymyxin A	D	V	G	E	I	S	A	I	D	K	L-Dab
	M-1	HE577054.1	Polymyxin P	D	V	G	E	I	S	A	I	D	K	L-Dab
		KP262070.1	Polymyxin E	D	V	G	E	I	S	A	I	D	K	L-Dab
	B-LR	LS992241.1	Polymyxin E	D	V	G	E	I	S	A	I	D	K	L-Dab
	PKB1	JN660148.1	D-Dab3-Polymyxin B	D	V	G	E	I	S	A	I	D	K	L-Dab
Region 10														
**pmxB1**	Polymyxin Query			D	F	W	N	I	G	M	V	H	K	L-Thr
	E681	EU371992.1	Polymyxin A	D	F	W	N	I	G	M	V	H	K	L-Thr
	M-1	HE577054.1	Polymyxin P	D	F	W	N	I	G	M	V	H	K	L-Thr
		KP262070.1	Polymyxin E	D	F	W	N	I	G	M	V	H	K	L-Thr
	B-LR	LS992241.1	Polymyxin E	D	F	W	N	I	G	M	V	H	K	L-Thr
	PKB1	JN660148.1	D-Dab3-Polymyxin B	D	F	W	N	I	G	M	V	H	K	L-Thr

Supplementary Figure S1 showing the chromatogram of the *P. polymyxa* 1G CFS (top) and the equivalent chromatogram of a colistin sulphate commercial sample (bottom). This highlights that they both elute under the same conditions.

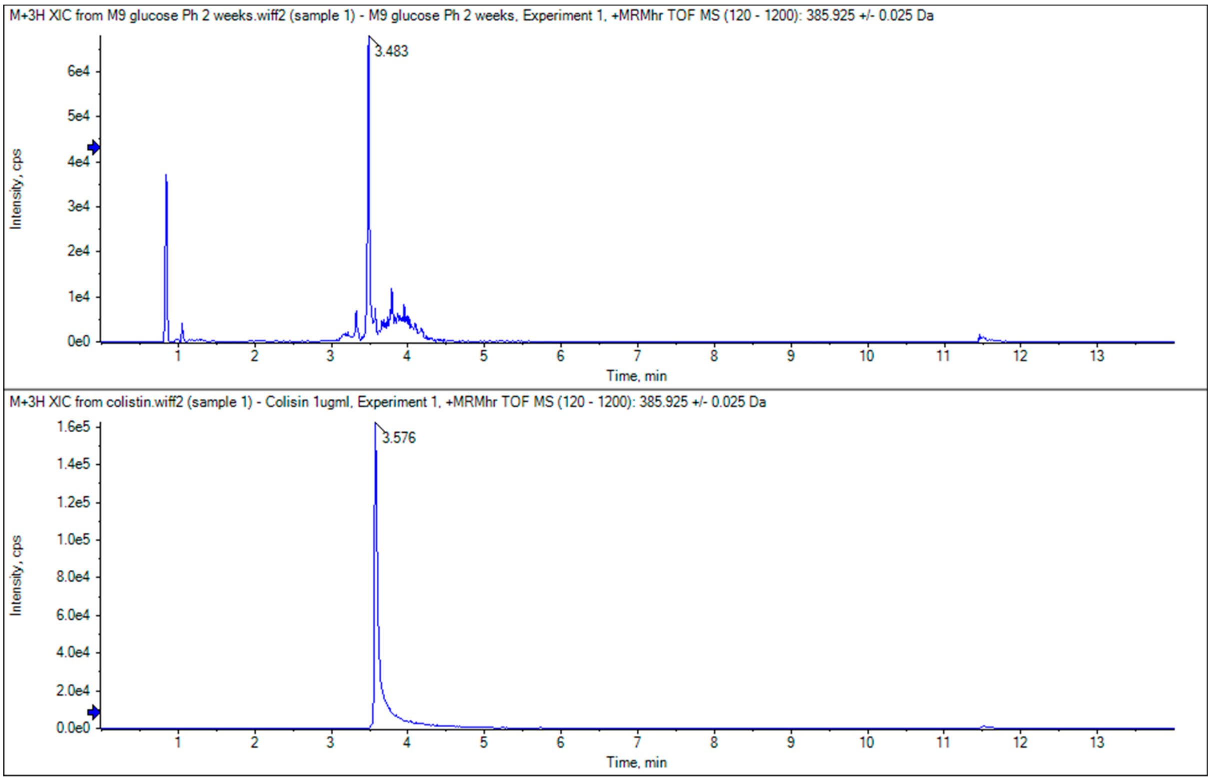

Figure S1: chromatogram showing elution of commercial colistin and *P. polymyxa* 1G

CLUSTAL O(1.2.4) multiple sequence alignment of pmxA

```
pmxA_Polymyxin-P             ATGTTTGAAAAGGCGGGGAGCGAGGCTGAAGCGTTGCTGCGGCATATGCTCCGCCGGATT 60
pmxA_Polymyxin-A             ------------------------------------------------------------ 0
pmxA_1G_Polymyxin            ---------------------------------ttgctgcggcatatgctccgccggatt 27
pmxA-D-DaB3-Polymyxin-B      ------------------------------------------------------------ 0
pmxA_B-LR_Polymyxin-E        ATGTTTGAAAAGGCGGGGAGCATGGCTGTAGCGTTGCTGCGGCATATGCTCCGCCGGATT 60
pmxA_KP26_Polymyxin-E        ATGTTTGAAAAGGCGGGGAGCATGGCTGTAGCGTTGCTGCGGCATATGCTCCGCCGGATT 60


pmxA_Polymyxin-P             GCTTTTACCGATGAAGAGAGGGGAACGATCCAGATGGCTTTTGAAAAAGAAACGTTGTTT 120
pmxA_Polymyxin-A             ---------------------------------ATGGCTTTTGAAAAAGAAACGTTGTTT 27
pmxA_1G_Polymyxin            gcttttaccgatgaagagaggggaacgatccagatggcttttgaaaaagaaacgttgttt 87
pmxA-D-DaB3-Polymyxin-B      ---------------------------------ATGGCTTTTGAAAAAGAAACGTTGTTT 27
pmxA_B-LR_Polymyxin-E        GCTTTTACCGATGTAGAGAGGGGGACGATCCAGGTGGCTTTTGAAAAAGAAACGTTGTTT 120
pmxA_KP26_Polymyxin-E        GCTTTTACCGATGTAGAGAGGGGGACGATCCAGGTGGCTTTTGAAAAAGAAACGTTGTTT 120
                                                               **************************


pmxA_Polymyxin-P             TGGAACGAAAAATTCGGTAGCGACGATTATACCTTGACGCGGCTGCCTTACAGCAAAGCT 180
pmxA_Polymyxin-A             TGGAACGAAAAATTTGGTAACGACGATTATACCTTGACGCGGCTGCCTTACAGCAAAGCT 87
pmxA_1G_Polymyxin            tggaacgaaaaattcggtagcgacgattataccttgacgccgctgccttacagcaaagct 147
pmxA-D-DaB3-Polymyxin-B      TGGAACGAAAAATTCGGTAGCGACGATTATACCTTGACGCGGCTGCCTTACAGCAAAGCT 87
pmxA_B-LR_Polymyxin-E        TGGAACGAAAAATTCGGTAGTGACGATTATACCTTGACGCGGCTGCCTTACAGCAAAGCT 180
pmxA_KP26_Polymyxin-E        TGGAACGAAAAATTCGGTAGTGACGATTATACCTTGACGCGGCTGCCTTACAGCAAAGCT 180
                             ************** ****  ******************* *******************


pmxA_Polymyxin-P             CCAAGCTCCCTGGAGCCCATTATGACAACCGTCGGCGGTTCGCTTTCGGAGGAAGTGGCG 240
pmxA_Polymyxin-A             CCAAGCTCCCTGGCGCCCATTATGACAACCGTCGGCGGTTCGCTTTCGGAGGAAGCGGCG 147
pmxA_1G_Polymyxin            ccaagctccctggcgcccattatgaaaaccgtaggcggttcgctttcggaaaagcggcg 207
pmxA-D-DaB3-Polymyxin-B      CCAAGCTCCCTGGCGCCCATTATGACAACCGTCGGCGGGACGCTTTCGGAGGAAGTGGCG 147
pmxA_B-LR_Polymyxin-E        CCAAGCTCCCAGGCGCCCATTATGACAACCGTCGGCGGTTCGCTTTCGGAGAAAGCGGCG 240
pmxA_KP26_Polymyxin-E        CCAAGCTCCCAGGCGCCCATTATGACAACCGTCGGCGGTTCGCTTTCGGAGAAAGCGGCG 240
                             ********** ** *********** ****** *****  **********  *** ****


pmxA_Polymyxin-P             CAGCGCGTCCTTCAAATGAGCAAGGGCGCTCCGCTGGCCACTTTTATGATTTTGCTCGCA 300
pmxA_Polymyxin-A             CAGCGCGTCCTTCAAATGAGCAAGGGCGCTCCGCTGGCCGCTTTTATGATTTTGCTCGCC 207
pmxA_1G_Polymyxin            cagcgcgtccttcaaatgagcaagggcgctccgctggccgcttttatgattttgctcgcc 267
pmxA-D-DaB3-Polymyxin-B      CAGCGCGTCCTTCAAATGAGCAAGGGCGCTCCGCTGGCCACTTTTATGATTTTGCTCGCA 207
pmxA_B-LR_Polymyxin-E        CAGCGCGTCCTTCAAATGAGCAAGGGCGCTCCGCTGGCCGCTTTTATGATTTTGCTCGCC 300
pmxA_KP26_Polymyxin-E        CAGCGCGTCCTTCAAATGAGCAAGGGCGCTCCGCTGGCCGCTTTTATGATTTTGCTCGCC 300
                             *************************************** *******************
```

```
pmxA_Polymyxin-P             GGTGTTCAGTCGCTCTTGCATAAATATACGGGCGCTTCTGACATTCTGGTCGGCATGCCG 360
pmxA_Polymyxin-A             GGTGTTCAGTCGCTCTTGCATAAATATACAGGCGCTTCTGACATTCTGGTCGGCATGCCG 267
pmxA_1G_Polymyxin            ggtgttcagtcactcttgcataaatacacaggcgcttctgacattctggtcggcatgccg 327
pmxA-D-DaB3-Polymyxin-B      GGTGTTCAGTCGCTCTTGCATAAATATACAGGCGCTTCTGACATTCTGGTCGGCATGCCG 267
pmxA_B-LR_Polymyxin-E        GGTGTTCAGTCACTCTTGCATAAATATACAGGCGCTTCTGACATTCTGGTCGGCATGCCG 360
pmxA_KP26_Polymyxin-E        GGTGTTCAGTCACTCTTGCATAAATATACAGGCGCTTCTGACATTCTGGTCGGCATGCCG 360
                             *********** ************** ** ******************************

pmxA_Polymyxin-P             GTTGTACGGAAACCGACGGAGACGCGCCGATCCGTTAATCATACGGCCATTTTGAAAAAC 420
pmxA_Polymyxin-A             GTTGTACGGAAACCGACGGAGACGCGCCGATCCGTTAATCATACGGTCATTTTGAAAAAC 327
pmxA_1G_Polymyxin            gttgtacggaaaccgacggagacgcgtcgatccgttaatcatacggtcattttgaaaaac 387
pmxA-D-DaB3-Polymyxin-B      GTTGTACGGAAACCGACGGAGACGCGCCGATCCGTTAATCATACGGTCATTTTGAAAAAC 327
pmxA_B-LR_Polymyxin-E        GTTATACGGAAACCGACGGAGACGCGCCGATCCGTTAATCATACGGTCATTTTGAAAAGC 420
pmxA_KP26_Polymyxin-E        GTTATACGGAAACCGACGGAGACGCGCCGATCCGTTAATCATACGGTCATTTTGAAAAGC 420
                             *** ********************** ******************* *********** *

pmxA_Polymyxin-P             TCGCTTTCGGCGGGTGCGACTTTTAAAACGCTTCTGAGCGAGCTGAGGACTTCCTTGCCG 480
pmxA_Polymyxin-A             TCGCTTTCGGCGGGTGCGACTTTTAAAACGCTTCTGAACGAGCTGAGGACTTCCTTGCCG 387
pmxA_1G_Polymyxin            tcgctttcggcgggtgcgacttttaaaacgcttctgagcgagctgaggacttccttgccg 447
pmxA-D-DaB3-Polymyxin-B      TCGCTTTCGGCGGGTGCGACTTTTAAAACGCTTCTGAGCGAGCTGAGGACTTCCTTGCCG 387
pmxA_B-LR_Polymyxin-E        TTGCTTTCGGCGGGATCGACTTTTAAAACGCTTCTGAGCGAGCTGAGAACTTCGTTGCCG 480
pmxA_KP26_Polymyxin-E        TTGCTTTCGGCGGGATCGACTTTTAAAACGCTTCTGAGCGAGCTGAGAACTTCGTTGCCG 480
                             * ************  ********************* ********* ***** ******

pmxA_Polymyxin-P             GAAGCGATTCAGCATCAACATATTCCATTCTTAAAAATGACAGAGAAGCTGGACCTGCAA 540
pmxA_Polymyxin-A             AGAGCGATTCAGCATCAACATATTCCGTTCTTGAAAATGACGGAGAAGCTGGACCTGCAA 447
pmxA_1G_Polymyxin            gaagcgattcagcatcaacatattccgttcttgaaaatgacggagaagctggacctgcaa 507
pmxA-D-DaB3-Polymyxin-B      GAAGCGATTCAGCATCAACATATTCCGTTCTTGAAAATGACGGAGAAGCTGGACCTGCAA 447
pmxA_B-LR_Polymyxin-E        GAAACGATTCAGCATCAACATATTCCGTTCTTGAAAATGACGGAGAAGCTGGATCTGCAA 540
pmxA_KP26_Polymyxin-E        GAAACGATTCAGCATCAACATATTCCGTTCTTGAAAATGACGGAGAAGCTGGATCTGCAA 540
                               * ********************** ***** ******** *********** ******

pmxA_Polymyxin-P             TATGCAGACGGGATACCCGTCGTCCATACGCTAGTATCCCTTAAGGAGCTGCATCTGGAC 600
pmxA_Polymyxin-A             TATGCAGACGGGATACCCGTCGTCCATACGCTAGTATCCCTTAAGGAGCTGCATCTGGAC 507
pmxA_1G_Polymyxin            tatgcagacggtatacccgtcatccatacgctagtatcccttaaggagctgcatctggac 567
pmxA-D-DaB3-Polymyxin-B      TATGCAGGCGGGATACCCGTCGTCCATACGCTAGTATCCCTTAAGGAGCTGAATCTGGAC 507
pmxA_B-LR_Polymyxin-E        TATGCAGACGGGATACCCATCATCCATACGCTAGTATCCCTTAAGGAGCTGCATCTGGAT 600
pmxA_KP26_Polymyxin-E        TATGCAGACGGGATACCCATCATCCATACGCTAGTATCCCTTAAGGAGCTGCATCTGGAT 600
                             ******* *** ****** ** ***************************** *******

pmxA_Polymyxin-P             GAAATCGGGCAAAACGTGGTCACGGATTGTTCCTTTGAATTTAGCTTAACCGGCGGGACG 660
pmxA_Polymyxin-A             GAAATCGGGCAAAACGTGGTCACGGATTGTTCCTTTGAATTCAGCTTAACCGGCGGGACG 567
pmxA_1G_Polymyxin            gaaatcgggcaaaatgtggtcacggattgttcctttgaattcagcttaaccgacgggacg 627
```

```
pmxA-D-DaB3-Polymyxin-B      GAAATCGGGCAAAACGTGGTCACAGATTGTTCCTTTGAATTCAGCTTAACCGGCGGGACG 567
pmxA_B-LR_Polymyxin-E        GAAATTGGGCAAAACGTGGTCACGGATTGTTCCTTTGAATTCAGCTTAACCGGCGGGACG 660
pmxA_KP26_Polymyxin-E        GAAATTGGGCAAAACGTGGTCACGGATTGTTCCTTTGAATTCAGCTTAACCGGCGGGACG 660
                             ***** ******** ******** **************** ********** *******

pmxA_Polymyxin-P             ATACAGCTAGCGCTGTCATATAACGAGCACTTATATGACTCCGAGTTTATGACGCGGGTC 720
pmxA_Polymyxin-A             ATACAGCTAGCGCTGTCATATAACGAGCACTTATATGACTCCGAGTTCATGACTCGGGTC 627
pmxA_1G_Polymyxin            atacagctagcgctgtcatataacgagcacttatatgactccgagtttatgactcgggtc 687
pmxA-D-DaB3-Polymyxin-B      ATACAGCTAGCGCTGTCATATAACGAGCACTTACATGACTCCGAGTTCATGACTCGGGTC 627
pmxA_B-LR_Polymyxin-E        ATACAGCTAGCGCTCTCATATAACGAGCACTTATATGACTCCAAGTTCATGACTCGGATC 720
pmxA_KP26_Polymyxin-E        ATACAGCTAGCGCTCTCATATAACGAGCACTTATATGACTCCAAGTTCATGACTCGGATC 720
                             ************** ***************** ******** **** ***** *** **

pmxA_Polymyxin-P             GTCGGCCATCTGAATCGCCTGCTGGCCGTGGGGCTTTACGAGTTGGAGCTGGACATTGTG 780
pmxA_Polymyxin-A             GTCGGCCATCTGAATCGCCTGCTGGTCGTGGGGCTTCACGAGTTGGAGCTGGACATCGTG 687
pmxA_1G_Polymyxin            gtcggccatctgaatcgcctgctggccgtggggcttcacgagttggagctggacatcgtg 747
pmxA-D-DaB3-Polymyxin-B      GTCGGCCATCTGAATCGCCTGCTGGCCGTGGGGCTTCACGAGTTGGAGCTGGACATCGTG 687
pmxA_B-LR_Polymyxin-E        GTCGGCCATCTGAATCGCCTGCTGGCCGTGGGGCTTCACGAGTTGGAGCTGGACATCGTG 780
pmxA_KP26_Polymyxin-E        GTCGGCCATCTGAATCGCCTGCTGGCCGTGGGGCTTCACGAGTTGGAGCTGGACATCGTG 780
                             ************************* ********** ****************** ***

pmxA_Polymyxin-P             CGGGCGGACATGCTGTCGGAGGACGAGAAATTTCAATTGCTGCAAAGCTTTAACGATACC 840
pmxA_Polymyxin-A             CGGGCGGACATGCTGTCGGAAGACGAGAAATTTCAATTGCTGCAAAGCTTTAACGATACC 747
pmxA_1G_Polymyxin            cgggcggacatgctgtcggaggacgagaaatttcaattgctgcaaagctttaacgatacc 807
pmxA-D-DaB3-Polymyxin-B      CGGGCGGACATGCTGTCGGAGGACGAGAAATTTCAATTGCTGCAAAGCTTTAACGATACC 747
pmxA_B-LR_Polymyxin-E        CGGGTGGACATGCTGTCGGAGGACGAGAAATTTCAATTGCTGCAAAGCTTTAACGATACC 840
pmxA_KP26_Polymyxin-E        CGGGTGGACATGCTGTCGGAGGACGAGAAATTTCAATTGCTGCAAAGCTTTAACGATACC 840
                             **** *************** ***************************************

pmxA_Polymyxin-P             GAGAAGGACTATCCCCGTGATCGGACGATTCATCAGCTCGTGGAGGAGCAGGCGAAGCGG 900
pmxA_Polymyxin-A             GAGAAGGACTATCCCCGTGATCGGACAATTCATCAGCTCGTGGAGGAGCAGGCGAAGCGG 807
pmxA_1G_Polymyxin            gagaaggactatccccgtgatcggacgattcatcagctcgtggaggagcaggcgaagcgg 867
pmxA-D-DaB3-Polymyxin-B      GAGAAGGACTATCCCCGTGATCGGACGATTCATCAGCTCGTGGAGGAGCAGGCGAAGCGG 807
pmxA_B-LR_Polymyxin-E        GAGAAGGACTATCCTCGAGATCGGACGATTCATCAGCTCGTGGAGGAGCAGGTGAAGAGG 900
pmxA_KP26_Polymyxin-E        GAGAAGGACTATCCTCGAGATCGGACGATTCATCAGCTCGTGGAGGAGCAGGTGAAGAGG 900
                             ************** ** ******** ************************* **** **

pmxA_Polymyxin-P             GTGCCCGAAGCGACGGCGGTTGTCTTTGAGGGGCGTCGTCTTTCGTACGCGGAGCTGAAC 960
pmxA_Polymyxin-A             GTGCCCGAAGCGACGGCGGTTGTCTTTGAGGGGCGGCGGCTTTCGTACGCGGAGCTGAAC 867
pmxA_1G_Polymyxin            gtgcccgaagcgacggcggttgtctttgaggggcggcggctttcgtacgcggagctgaac 927
pmxA-D-DaB3-Polymyxin-B      GTGCCCGAAGCGACGGCGGTTGTCTTTGAGGGGCGGCGGCTTTCGTACGCGGAGCTGAAC 867
pmxA_B-LR_Polymyxin-E        GTGCCCGAAGCAACGGCGATTGTCTTTGAGGGGCGGCGGCTTTCGTACGCTGAGCTGAAC 960
pmxA_KP26_Polymyxin-E        GTGCCCGAAGCAACGGCGATTGTCTTTGAGGGGCGGCGGCTTTCGTACGCTGAGCTGAAC 960
                             *********** ****** **************** ** *********** *********
```

```
pmxA_Polymyxin-P            GAACGGGCAAACCGGCTGGCGCGGACGCTGCGATCGGTCGGCGTGCTGCCCAATCAGCTG 1020
pmxA_Polymyxin-A            GAACGGGCGAACCGGCTGGCGCGGACGTTGCGATCGATCGGCGTGCTGCCCAATCAGCTG 927
pmxA_1G_Polymyxin           gaacgggcgaaccggctggcgcggacgttgcgatcggtcggcgtgctgcccaatcagctg 987
pmxA-D-DaB3-Polymyxin-B     GAACGGGCGAACCGGCTGGCGCGGACGCTGCGATCGGTCGGCGTGCTGCCCAATCAGCTG 927
pmxA_B-LR_Polymyxin-E       GAACGGGCGAACCGGCTGGCGCGGACGCTGCGATCGGTCGGCGTGCTGCCCAATCAGCTG 1020
pmxA_KP26_Polymyxin-E       GAACGGGCGAACCGGCTGGCGCGGACGCTGCGATCGGTCGGCGTGCTGCCCAATCAGCTG 1020
                            ******** ****************** ******** ***********************

pmxA_Polymyxin-P            GTAGGCTTGATGGCCAGGAGATCGCTGGAGACGGTCGTTGGCATTCTGGCGGTTCTGAAA 1080
pmxA_Polymyxin-A            GTAGGCTTGATGGTCAGAAGATCGTTGGAGACGGTCGTTGGCATTCTGGCGGTTCTGAAA 987
pmxA_1G_Polymyxin           gtaggcttgatggtcaggagatcgctggagacggtcgttggcattctggcggttctgaaa 1047
pmxA-D-DaB3-Polymyxin-B     GTAGGCTTGATGGCCAGGAGATCGCTGGAGACGGTCGTTGGCATTCTGGCGGTTCTGAAA 987
pmxA_B-LR_Polymyxin-E       GTAGGCTTGATGGCCAGGAGATCGCTGGAGACGGTCGTTGGCATTCTGGCGGTTCTGAAA 1080
pmxA_KP26_Polymyxin-E       GTAGGCTTGATGGCCAGGAGATCGCTGGAGACGGTCGTTGGCATTCTGGCGGTTCTGAAA 1080
                            ************* *** ****** ***********************************

pmxA_Polymyxin-P            GCTGGCGGTGCCTACGTGCCGATCGACCCGGAATACCCGGAAGAACGCATCCGCTACATT 1140
pmxA_Polymyxin-A            GCTGGCGGTGCCTACGTGCCGATCGACCCGGAATACCCGGAAGAACGCATCCGCTACATT 1047
pmxA_1G_Polymyxin           gctggcggtgcctacgtgccgatcgacccggaatacccggaagaacgcatccgctacatt 1107
pmxA-D-DaB3-Polymyxin-B     GCTGGCGGTGCCTACGTGCCGATCGACCCGGAATACCCGGAAGAACGCATCCGCTACATT 1047
pmxA_B-LR_Polymyxin-E       GCTGGCGGTGCCTACGTGCCGATCGACCCGGAATACCCGGAAGAACGCATCCGCTACATT 1140
pmxA_KP26_Polymyxin-E       GCTGGCGGTGCCTACGTGCCGATCGACCCGGAATACCCGGAAGAACGCATCCGCTACATT 1140
                            ************************************************************

pmxA_Polymyxin-P            CTGGAGAACTCGAACGCGCAGCTGTTGCTGACGCAAAGGGAGCTGCAGCA---GCAGGTG 1197
pmxA_Polymyxin-A            TTGGAGAACTCGAACGCGCAGCTGCTGCTGACTCAAAGGGAGCTGCTGCAGCTGCAGGTG 1107
pmxA_1G_Polymyxin           ctggagaactcgaacgcgcagctgctgctgactcaaaggcacctgctgca---gcaggtg 1164
pmxA-D-DaB3-Polymyxin-B     CTGGAGAACTCGAACGCGCAGCTGCTGCTGACTCAAAGGGAGCTGCTGCA---GCAGGTG 1104
pmxA_B-LR_Polymyxin-E       CTGGAGAACTCGAACGCGCAGCTGCTGCTGACTCAAAGAAAGCTGCAACA---GCAGGTG 1197
pmxA_KP26_Polymyxin-E       CTGGAGAACTCGAACGCGCAGCTGCTGCTGACTCAAAGAAAGCTGCAACA---GCAGGTG 1197
                             *********************** ******* *****  * ****  **   *******

pmxA_Polymyxin-P            CCGTTCGAAGGGACCGTGGTGGCGCTGGATGACGAGCAGGCCTACAGCGACGATGGCTCG 1257
pmxA_Polymyxin-A            CCGTTCGAAGGGACCGTGGTGGCGCTGGATGACGAGCAGGCCTACAGCGACGATGGCTCG 1167
pmxA_1G_Polymyxin           ccgttcgaagggaccgtggtggcgctggatgacgagcaggcctatagcgccgatggctcg 1224
pmxA-D-DaB3-Polymyxin-B     CCATTCGAAGGGACCGTGGTGGCGCTGGATGACGAGCAGGCCTACAGCGACGATGGCTCG 1164
pmxA_B-LR_Polymyxin-E       CCGTTCGAAGGGACTGTGTTGGCGCTGGATGACGAGCAGGCCTACAGCGATGATGGAACG 1257
pmxA_KP26_Polymyxin-E       CCGTTCGAAGGGACTGTGTTGGCGCTGGATGACGAGCAGGCCTACAGCGATGATGGAACG 1257
                            ** *********** *** ************************* ****  *****  **

pmxA_Polymyxin-P            AACCTGGATCCGGCCAGCGGTCCGAATGATCTGGCTTATGTCATCTATACATCAGGTACG 1317
pmxA_Polymyxin-A            AACCTGGAGCCGGCCAGCGGTCCGAACGATCTGGCTTATGTCATCTATACGTCAGGTACG 1227
pmxA_1G_Polymyxin           aacctggagccggccagcggtccgaacgatctggcttatgtcatctatacgtcaggtacg 1284
```

```
pmxA-D-DaB3-Polymyxin-B      AACCTGGAGCCGGCCAGCGGTCCGAACGATCTGGCTTATGTCATCTATACATCAGGTACG 1224
pmxA_B-LR_Polymyxin-E        AATCTGGAGCCGGCCAGCGGTTCGAATGATCTGGCTTATGTCATCTATACGTCAGGTACG 1317
pmxA_KP26_Polymyxin-E        AATCTGGAGCCGGCCAGCGGTTCGAATGATCTGGCTTATGTCATCTATACGTCAGGTACG 1317
                             ** ***** ************ **** *********************** *********

pmxA_Polymyxin-P             ACGGGCAAACCCAAAGGGGTCATGCTGGAGCATCGCGGGCTGGTCAGCCTGAAACTGACG 1377
pmxA_Polymyxin-A             ACGGGCAATCCCAAAGGGGTCATGCTGGAGCATCACGGTTTGGTCAGCTTGAAGCTGATG 1287
pmxA_1G_Polymyxin            acgggcaatcccaaaggggtcatgctagagcatcgcggtttggtcagcttgaagctgatg 1344
pmxA-D-DaB3-Polymyxin-B      ACGGGCAAACCCAAAGGGGTCATGCTGGAGCATCGCGGGCTGGTCAGCCTGAAACTGACG 1284
pmxA_B-LR_Polymyxin-E        ACGGGCAAACCCAAAGGGGTCATGCTGGAGCATCGCGGTTTGGTCAGCTTGAAACTGATG 1377
pmxA_KP26_Polymyxin-E        ACGGGCAAACCCAAAGGGGTCATGCTGGAGCATCGCGGTTTGGTCAGCTTGAAACTGATG 1377
                             ******** ***************** ******* ***  ******** **** **** *

pmxA_Polymyxin-P             TTTGCCCATACGCTTCACACGACCGAACAGGATCGAGTGCTCCAATTCGCCAGCCTATCG 1437
pmxA_Polymyxin-A             TTCGCGGACAGGCTGGGCATCACGGAGCATGACCGGATCGTTCAATTCGCCAGCCTGTCG 1347
pmxA_1G_Polymyxin            ttcgcggacaggctgggcatcacggagcatgaccggatcgttcaattcgccagcctgtcg 1404
pmxA-D-DaB3-Polymyxin-B      TTTGCCCATACGCTTCACACGACCGAACAGGATCGAGTGCTGCAATTCGCCAGCCTATCG 1344
pmxA_B-LR_Polymyxin-E        TTCGCGGACAGGCTCGGCATCACGGAGCATGACCGGATCGTTCAATTCGCCAGCCTGTCG 1437
pmxA_KP26_Polymyxin-E        TTCGCGGACAGGCTCGGCATCACGGAGCATGACCGGATCGTTCAATTCGCCAGCCTGTCG 1437
                             ** **  * * ***   **  ** ** ** ** **  *  * ************* ***

pmxA_Polymyxin-P             TTCGATGCGTCGTGCTGGGAGATGTTCAACGCTCTATATTTCGGGGCGACCTTATATATA 1497
pmxA_Polymyxin-A             TTCGACGCGTCCTGCTGGGAAGTGTTCAAAGCGCTCTATTTTGGCGCGGCTTTGTACATC 1407
pmxA_1G_Polymyxin            ttcgacgcgtcctgctgggaagtgttcaaagcgctctattttggcgcggctttgtacatc 1464
pmxA-D-DaB3-Polymyxin-B      TTCGATGCGTCGTGCTGGGAGATGTTCAACGCTCTGTATTTCGGGGCGACCTTATATATA 1404
pmxA_B-LR_Polymyxin-E        TTCGACGCGTCCTGCTGGGAAGTGTTCAAAGCGCTCTATTTTGGCGCGACTTTGTACATA 1497
pmxA_KP26_Polymyxin-E        TTCGACGCGTCCTGCTGGGAAGTGTTCAAAGCGCTCTATTTTGGCGCGACTTTGTACATA 1497
                             ***** ***** ********  ******* ** ** ***** ** *** * ** ** **

pmxA_Polymyxin-P             CCGTCAACCGAGACGATTTTGGATGATCAGCTGTTCGAACGTTTCATGAACGAGCATGCA 1557
pmxA_Polymyxin-A             CCGACGGCCGAGACGATTCTCGACAACCGCCTGTTCGAGAGTTATATGAACGAGCATGCG 1467
pmxA_1G_Polymyxin            ccgacggccgagacgattctcgacaaccgcctgttcgagagttatatgaacgagcatgcg 1524
pmxA-D-DaB3-Polymyxin-B      CCGTCAACCGAGACGATTTTGGATGATCAGCTGTTCGAACGTTTCATGAACGAGCATGCA 1464
pmxA_B-LR_Polymyxin-E        CCGACGGCCGAGACGATTCTCGACAACCGCCTGTTTGAGAGTTATATGAACGAGCATGCG 1557
pmxA_KP26_Polymyxin-E        CCGACGGCCGAGACGATTCTCGACAACCGCCTGTTTGAGAGTTATATGAACGAGCATGCG 1557
                             *** *  *********** * **  * *  ***** **  ***  **************

pmxA_Polymyxin-P             ATTACGATCGCCACTTTGCCGCCAACTTATGCGGCTTATTTGAATTCAGACCGCCTGCCG 1617
pmxA_Polymyxin-A             ATTACGGCGGCGATTTTGCCTCCGACGTACAGTGCTTATTTGAACCCGGACCGCCTTCCC 1527
pmxA_1G_Polymyxin            attacggcggcgattttgcctccgacgtacagtgcttatttgaacccggaccgccttccc 1584
pmxA-D-DaB3-Polymyxin-B      ATTACGATCGCCACTTTGCCGCCAACTTATGCGGCTTATTTGAATTCAGACCGCCTGCCG 1524
pmxA_B-LR_Polymyxin-E        ATTACGGCGGCGATTTTGCCTCCGACGTACAGCGCTTATTTGAACCCGGACCGCCTTCCC 1617
pmxA_KP26_Polymyxin-E        ATTACGGCGGCGATTTTGCCTCCGACGTACAGCGCTTATTTGAACCCGGACCGCCTTCCC 1617
                             ******   ** * ****** ** ** **    ***********  * ******** **
```

```
pmxA_Polymyxin-P              AGTCTGAGCAGGCTCATCACGGCGGGCTCCGCCGTTTCAGCCGAATTCGTGCAGCAGTGG 1677
pmxA_Polymyxin-A              AGCTTAACGAAGCTCGTAACGGGAGGCTCGGCGGTATCGGCCGAATTCGTGCAGCAGTGG 1587
pmxA_1G_Polymyxin             agcttaacgaagctcgtaacgggaggctcggcggtatcggccgaattcgtgcagcagtgg 1644
pmxA-D-DaB3-Polymyxin-B       AGTCTGAGCAGGCTCATCACGGCGGGCTCCGCCGTTTCAGCCGAATTCGTGCAGCAGTGG 1584
pmxA_B-LR_Polymyxin-E         AGCTTAACGAAGCTCGTAACGGGAGGCTCGGCGGTATCGGCCGAATTCGTGCAGCAGTGG 1677
pmxA_KP26_Polymyxin-E         AGCTTAACGAAGCTCGTAACGGGAGGCTCGGCGGTATCGGCCGAATTCGTGCAGCAGTGG 1677
                              **  * *  * **** * ****  ***** ** ** ** *********************

pmxA_Polymyxin-P              AAAGACAAGGTACAATACTATAACGCGTACGGTCCGACGGAAGCCTCGATCGCCACATCC 1737
pmxA_Polymyxin-A              AAACGCAAGGTCCACTATTTCAATGCTTACGGCCCTACCGAAGCTTCGATTGTTACGACG 1647
pmxA_1G_Polymyxin             aaacgcaaggtccactatttcaatgcttacggccctaccgaagcttcgattgttacgaca 1704
pmxA-D-DaB3-Polymyxin-B       AAAGACAAGGTACAGTACTATAACGCGTACGGTCCGACGGAAGCCTCGATCGCCACATCC 1644
pmxA_B-LR_Polymyxin-E         AAACCGAAGGTCCACTATTTCAATGCTTACGGCCCTACCGAAGCTTCGATTGTTACGACG 1737
pmxA_KP26_Polymyxin-E         AAACCGAAGGTCCACTATTTCAATGCTTACGGCCCTACCGAAGCTTCGATTGTTACGACG 1737
                              ***   ***** ** ** *  ** ** ***** ** ** ***** ***** *  **  *

pmxA_Polymyxin-P              GTGTGGGCGGCGTCTACGTATGACACCGAACGCAGAGCGATACCGATCGGACGTCCGATC 1797
pmxA_Polymyxin-A              CTTTGGGATGCGGATGAGGAGCAGCCGGAGGGCAGAGTCATTCCGATCGGGCGCCCGCTG 1707
pmxA_1G_Polymyxin             ctttgggatgcggatgaggagcagccggagggcagagtcattccgatcgggcgcccgctg 1764
pmxA-D-DaB3-Polymyxin-B       GTGTGGGCGGCGTCTACGTATGACACCGAACGCAGAGCGATACCGATCGGACGTCCGATC 1704
pmxA_B-LR_Polymyxin-E         CTTTGGGATGCGAATGAGGAGCAGCCAGAGCGCAGAGTCATTCCGATTGGGCGCCCGCTG 1797
pmxA_KP26_Polymyxin-E         CTTTGGGATGCGAATGAGGAGCAGCCAGAGCGCAGAGTCATTCCGATTGGGCGCCCGCTG 1797
                               * ****  ***  *  * *  *  * **  ******  ** ***** ** ** *** *

pmxA_Polymyxin-P              TGGAACCATCGCTTGTATATACTGGGCGCACAGAATCAACTGGCGCCTATCGGAGTGGAA 1857
pmxA_Polymyxin-A              GCCAATCACCGGATTTTTATTTTGGATGCCCACCTGCAGCTTGTGCCTCCGGGAGTGGAC 1767
pmxA_1G_Polymyxin             gccaatcaccggatttttattttggatgcccacctgcagcttgtgcctccgggagtggac 1824
pmxA-D-DaB3-Polymyxin-B       TGGAACCATCGCTTGTATATACTGGGCGCACAGAATCAGCTGGCGCCTATCGGAGTGGAG 1764
pmxA_B-LR_Polymyxin-E         GCCAATCACCGGATTTTTATTTTGGATGCCCACCTGCAGCTTGTGCCTCCGGGAGTGGAC 1857
pmxA_KP26_Polymyxin-E         GCCAATCACCGGATTTTTATTTTGGATGCCCACCTGCAGCTTGTGCCTCCGGGAGTGGAC 1857
                                 ** ** **  * * ***  ***  ** **    ** ** * ****   ********

pmxA_Polymyxin-P              GGGGAATTGTGCATCGCGGGCGTCGGCCTCGCCCGGGGATATTGGAACCGGCCGGATCTG 1917
pmxA_Polymyxin-A              GGCGAGCTGTGCGTGGCAGGCGTGGGGCTTGCGAGAGGTTACCTGAACCATCCGGAGCTG 1827
pmxA_1G_Polymyxin             ggcgagctgtgcgtggcaggcgtggggcttgcgagaggttacctgaaccatccggagctg 1884
pmxA-D-DaB3-Polymyxin-B       GGGGAATTGTGCATCGCGGGCGTCGGCCTCGCCCGGGGATATTGGAACCGGCCGGATCTG 1824
pmxA_B-LR_Polymyxin-E         GGCGAGCTGTGCGTGGCAGGCGTGGGGCTTGCGAGAGGTTACCTGAACCATCCGGAGCTG 1917
pmxA_KP26_Polymyxin-E         GGCGAGCTGTGCGTGGCAGGCGTGGGGCTTGCGAGAGGTTACCTGAACCATCCGGAGCTG 1917
                              ** **  ***** * ** ***** ** ** **  * ** **   *****  ***** ***

pmxA_Polymyxin-P              ACGGCGGAGAAATTCATCGAACACCCGTTTGAGCCCGGAGAGAGACTGTACCGGACGGGC 1977
pmxA_Polymyxin-A              ACGGCAGAGAAGTTCGTGGAACATCCGTTTGCGCCGGGAGAACGCCTTTACCGGACGGGA 1887
pmxA_1G_Polymyxin             acggcagagaagttcgtggaacatccgtttgcgccgggagaacgcctttaccggacggga 1944
```

```
pmxA-D-DaB3-Polymyxin-B       ACGGCGGAGAAATTCATCGAACACCCGTTTGAGCCCGGAGAGAGAATGTACCGGACGGGC 1884
pmxA_B-LR_Polymyxin-E         ACGGCAGAAAAGTTCGTGGAACATCCGTTCGCGCCGGGAGAACGCCTTTATCGGACGGGA 1977
pmxA_KP26_Polymyxin-E         ACGGCAGAAAAGTTCGTGGAACATCCGTTCGCGCCGGGAGAACGCCTTTATCGGACGGGA 1977
                              ***** ** ** *** * ***** ***** * *** *****  *  * ** ******** 

pmxA_Polymyxin-P              GACTTGGCCCGCTGGCTGCCGGACGGAAATATTGAGTACTTGGGCCGGATTGACCATCAG 2037
pmxA_Polymyxin-A              GATCTCGCCCGCTGGCTGCCGGACGGAAATGTTGAGTACTTGGGCCGGATCGACCATCAG 1947
pmxA_1G_Polymyxin             gatctcgcccgctggctgccggacggaaatattgagtacttgggtcggatcgaccatcag 2004
pmxA-D-DaB3-Polymyxin-B       GACTTGGCCCGCTGGCTGCCGGACGGAAATATTGAGTACTTGGGCCGGATTGACCATCAG 1944
pmxA_B-LR_Polymyxin-E         GATCTCGCCCGATGGCTGCCGGACGGAAATATTGAGTACTTGGGCCGGATCGACCATCAG 2037
pmxA_KP26_Polymyxin-E         GATCTCGCCCGATGGCTGCCGGACGGAAATATTGAGTACTTGGGCCGGATCGACCATCAG 2037
                              **  * ***** ****************** ************* ***** *********

pmxA_Polymyxin-P              GTGAAAATCCGTGGATTCCGGATCGAGATCGGCGAGATTGAAGAGCAGCTTCTGAAGATC 2097
pmxA_Polymyxin-A              GTGAAAATCCGTGGATTCCGGATCGAGATCGGCGAGATTGAAGAGCAGCTTCTGAAGATC 2007
pmxA_1G_Polymyxin             gtgaaaatccgtggattccggatcgagatcggcgagattgaagagcagcttctgaagatc 2064
pmxA-D-DaB3-Polymyxin-B       GTGAAAATCCGTGGATTCCGGATCGAGATCGGCGAGATTGAAGAGCAGCTTCTGAAGATC 2004
pmxA_B-LR_Polymyxin-E         GTGAAAATCCGTGGATTCCGGATCGAGATCGGCGAGATTGAAGAGCAGCTTCTGAAGATC 2097
pmxA_KP26_Polymyxin-E         GTGAAAATCCGTGGATTCCGGATCGAGATCGGCGAGATTGAAGAGCAGCTTCTGAAGATC 2097
                              ************************************************************

pmxA_Polymyxin-P              GACTCCGTGCAGGAGACGATCGTAATCGCGCGGGAAGGCAAAAACGGGCAAGAACTGTGC 2157
pmxA_Polymyxin-A              GACTCCGTGCAGGAGACGATCGTAATCGCGCGGGAAGGCAAAAGCGGGCAAGAACTGTGC 2067
pmxA_1G_Polymyxin             gactccgtgcaggagacgatcgtaatcgcgcgggaaggcaaaagcgggcaagaactgtgc 2124
pmxA-D-DaB3-Polymyxin-B       GACTCCGTGCAGGAGACGATCGTAATCGCGCGGGAAGGCAAAAGCGGGCAAGAACTGTGC 2064
pmxA_B-LR_Polymyxin-E         GACTCCGTGCAGGAGACGATGGTAATCGCGCGGGAAGGCAAAAGCGGGCAAGAATTGTGC 2157
pmxA_KP26_Polymyxin-E         GACTCCGTGCAGGAGACGATGGTAATCGCGCGGGAAGGCAAAAGCGGGCAAGAATTGTGC 2157
                              ******************** ********************** ********** *****

pmxA_Polymyxin-P              GCTTACCTGGTCGCGGGCCGCCCGCTTACGCTCGGCGAGCTGAGAAGCGCGCTGGCGCAA 2217
pmxA_Polymyxin-A              GCTTACCTGGTCGCGGGCCGCCCGCTTACGCTCGGCGAGCTAAGAAGCGCGCTGGCGCAA 2127
pmxA_1G_Polymyxin             gcttacctggtcgcggggcgcccgcttacgctcggcgagctaagaagcgcgctggcgcaa 2184
pmxA-D-DaB3-Polymyxin-B       GCTTATCTGGTCGCGGGCCGCCCGCTTACGCTCGGCGAGCTGAGAAGCGCGCTGGCGCAA 2124
pmxA_B-LR_Polymyxin-E         GCTTATCTGGTCGCGGACCGCCCGCTTACGCTCGGCGAGCTGAGAAGCGCGCTGGCGCAA 2217
pmxA_KP26_Polymyxin-E         GCTTATCTGGTCGCGGACCGCCCGCTTACGCTCGGCGAGCTGAGAAGCGCGCTGGCGCAA 2217
                              ***** **********  *********************** ******************

pmxA_Polymyxin-P              AAATTGCCGAATTACATGATTCCGGCGCATTTTGTTCAGCTTCCGCGGATGCCGCTCACG 2277
pmxA_Polymyxin-A              AAATTGCCGAATTACATGATTCCGGCGCATTTTGTTCAGCTTCCGCAGATGCCGCTCACG 2187
pmxA_1G_Polymyxin             aaattgccgaattacatgattccggcgcattttgttcagcttccgcagatgccgctcacg 2244
pmxA-D-DaB3-Polymyxin-B       AAATTGCCGAATTACATGATTCCGGCGCATTTTGTTCAGCTTCCGCGGATGCCGCTCACG 2184
pmxA_B-LR_Polymyxin-E         AAATTGCCGAATTACATGATTCCGGCGCATTTTGTTCAGCTTCCGCGAATGCCGCTCACG 2277
pmxA_KP26_Polymyxin-E         AAATTGCCGAATTACATGATTCCGGCGCATTTTGTTCAGCTTCCGCGAATGCCGCTCACG 2277
                              **********************************************  ************
```

```
pmxA_Polymyxin-P            CCGAACGACAAAATCGACCGCAAGGCTTTGCCTGCTCCGGAAGGAAATGCGCTGACCGGC 2337
pmxA_Polymyxin-A            CCGAACGACAAAATCGACCGCAAGGCTTTGCCCGCTCCGGAAGGAAACGCGCTGACCGGC 2247
pmxA_1G_Polymyxin           ccgaacgacaaaatcgaccgcaaggctttgcccgctccggaaggaaacgcgctgaccggc 2304
pmxA-D-DaB3-Polymyxin-B     CCGAACGACAAAATCGACCGCAAGGCTTTGCCCGCTCCGGAAGGAAACGCGCTGACCGGC 2244
pmxA_B-LR_Polymyxin-E       CCGAACGACAAAATCGACCGCAAGGCTTTGCCCGCCCCGGAAGGAAACGCGCTGACCGGC 2337
pmxA_KP26_Polymyxin-E       CCGAACGACAAAATCGACCGCAAGGCTTTGCCCGCCCCGGAAGGAAACGCGCTGACCGGC 2337
                            ******************************** ** *********** ************

pmxA_Polymyxin-P            GGCGCGTACGTAGCTCCTCGCAATGAAGCCGAGCGGACGCTTGCCGATGTGTGGCAGGCG 2397
pmxA_Polymyxin-A            GGCGCGTACGTAGCTCCCCGCAATGAAGCCGAGCGGACGCTTGCCGATGTGTGGCAGGCG 2307
pmxA_1G_Polymyxin           ggcgcgtacgtagctccccgcaatgaagccgagcggacgcttgccgatgtgtggcaggcg 2364
pmxA-D-DaB3-Polymyxin-B     GGCGGGTACGTAGCTCCCCGCAATGAAGCCGAGCGGACGCTTGCCGATGTGTGGCAGGCG 2304
pmxA_B-LR_Polymyxin-E       GGCTTGTACGTAGCTCCCCGCAATGAAGCCGAGCGGACACTTGTCGATGTGTGGCAGGCG 2397
pmxA_KP26_Polymyxin-E       GGCTTGTACGTAGCTCCCCGCAATGAAGCCGAGCGGACACTTGTCGATGTGTGGCAGGCG 2397
                            ***  ************ ******************** **** ****************

pmxA_Polymyxin-P            GTATTGAACGCCGATCGCGTTGGGGTAACGGATCACTTCTTCGAGCTGGGCGGAGACTCG 2457
pmxA_Polymyxin-A            GTATTGAACGCCGATCGCGTTGGGGTAACGGATCATTTCTTCGAGCTGGGCGGAGACTCG 2367
pmxA_1G_Polymyxin           gtattgaacgccgatcgcgttggggtaacggatcatttcttcgagctgggcggagactcg 2424
pmxA-D-DaB3-Polymyxin-B     GTATTGAACGCCGATCGCGTTGGGGTAACGGATCATTTCTTCGAGCTGGGCGGAGACTCA 2364
pmxA_B-LR_Polymyxin-E       GTATTGAACGCCGATCGTGTTGGGGTAACGGATCATTTCTTCGAGCTGGGTGGAGACTCG 2457
pmxA_KP26_Polymyxin-E       GTATTGAACGCCGATCGTGTTGGGGTAACGGATCATTTCTTCGAGCTGGGTGGAGACTCG 2457
                            ***************** ***************** ************** ******** 

pmxA_Polymyxin-P            ATCAAGTCCATTCAAGTATCTTCGCGGCTTCATCAAGCCGGGTACAAGCTGGAAATCCGG 2517
pmxA_Polymyxin-A            ATCAAGTCCATTCAAGTATCTTCGCGGCTTCATCAAGCCGGGTACAAGCTGGAAATCCGG 2427
pmxA_1G_Polymyxin           atcaagtccattcaagtatcttcgcggcttcatcaagccggctacaagctggaaatccgg 2484
pmxA-D-DaB3-Polymyxin-B     ATCAAGTCCATTCAAGTATCTTCGAGGCTTCATCAAGCCGGGTACAAGCTGGAAATACGG 2424
pmxA_B-LR_Polymyxin-E       ATCAAGTCCATTCAAGTATCTTCGCGGCTTCATCAAGCCGGGTACAAGCTGGATATTCGG 2517
pmxA_KP26_Polymyxin-E       ATCAAGTCCATTCAAGTATCTTCGCGGCTTCATCAAGCCGGGTACAAGCTGGATATTCGG 2517
                            ************************ **************** *********** ** ***

pmxA_Polymyxin-P            GATTTGTTCAAATATCCGACTATCTCACAGCTCAGCCTGCATGTGAAACCGATCGGACGT 2577
pmxA_Polymyxin-A            GATTTGTTCAAATATCCGACTATTTCACAGCTCAGCCTGCATGTGAAACCGATCGGACGT 2487
pmxA_1G_Polymyxin           gatttgttcaaatatccgactatttcacagctcagcctgcatgtgaaaccgatcggacgt 2544
pmxA-D-DaB3-Polymyxin-B     GATTTGTTCAAATATCCGACTATCTCACAGCTCAGCCTGCATGTGAAACCGATCGGACGT 2484
pmxA_B-LR_Polymyxin-E       GATTTGTTCAAATATCCGACTATCTCACAGCTCAGCCTGCGTGTGAAACCGATCGGACGT 2577
pmxA_KP26_Polymyxin-E       GATTTGTTCAAATATCCGACTATCTCACAGCTCAGCCTGCGTGTGAAACCGATCGGACGT 2577
                            *********************** **************** *******************

pmxA_Polymyxin-P            ACCATCGATCAAGGCGAAATAACGGGTGAAACGGCGCTGACGCCGATTCAGCATTGGTTT 2637
pmxA_Polymyxin-A            ACCATTGATCAAGGCGAAATAACGGGTGAAACGGCGCTGACGCCGATTCAGCATTGGTTT 2547
pmxA_1G_Polymyxin           accattgatcagggcgaaataacgggtgaaacggcgctgacgccgattcagcattggttt 2604
```

```
pmxA-D-DaB3-Polymyxin-B      ACCATCGATCAAGGTGAAATAACGGGTGAAACGGCGCTGACGCCGATTCAGCATTGGTTT 2544
pmxA_B-LR_Polymyxin-E        ACCATCGATCAAGGCGAAATAACGGGCGAAACGGCGCTGACGCCGATTCAGCATTGGTTT 2637
pmxA_KP26_Polymyxin-E        ACCATCGATCAAGGCGAAATAACGGGCGAAACGGCGCTGACGCCGATTCAGCATTGGTTT 2637
                             ***** ***** ** *********** *********************************

pmxA_Polymyxin-P             TTCGAGAGCTCCTTTGCGGACCCGCATCATTTCAACCAGTCGGTGATGCTGTACCGGAAG 2697
pmxA_Polymyxin-A             TTCGAGAGCTCCTTTGCGGACCCGCATCATTTCAACCAGTCGGTGATGCTGTACCGGAAG 2607
pmxA_1G_Polymyxin            ttcgagagctcctttgcggacccgcatcatttcaaccagtcggtgatgctgtaccggaag 2664
pmxA-D-DaB3-Polymyxin-B      TTCGAGAGCTCCTTTGCGGACCCGCATCATTTCGACCAGTCGGTGATGCTGTACCGGAAG 2604
pmxA_B-LR_Polymyxin-E        TTCGAGAGCTCCTTTGCGGATCCGCATCATTTCAACCAGTCGGTGATGCTGTACCGGAAG 2697
pmxA_KP26_Polymyxin-E        TTCGAGAGCTCCTTTGCGGATCCGCATCATTTCAACCAGTCGGTGATGCTGTACCGGAAG 2697
                             ******************** ************ **************************

pmxA_Polymyxin-P             GAACGCTTTGACGAAGAGACGGTGCGTCAGGTGCTGCAAAAGCTGGCCGAGCATCATGAC 2757
pmxA_Polymyxin-A             GAGCGCTTCGACGAAGAGACGGTGCGTCAGGTGCTGCAAAAGCTGGCCGAGCATCATGAC 2667
pmxA_1G_Polymyxin            gaacgcttcgacgaagagacggtgcgtcaggtgctgcaaaagctggccgagcatcatgac 2724
pmxA-D-DaB3-Polymyxin-B      GAACGCTTCGACGAAGAGACGGTACGTCAGGTGCTGCAAAAGCTGGCCGAGCATCATGAC 2664
pmxA_B-LR_Polymyxin-E        GAACGCTTCGACGAAGAGACGGTGCGTCAGGTACTGCAAAAGCTGGCCGAGCATCATGAC 2757
pmxA_KP26_Polymyxin-E        GAACGCTTCGACGAAGAGACGGTGCGTCAGGTACTGCAAAAGCTGGCCGAGCATCATGAC 2757
                             ** ***** ************** ******** ***************************

pmxA_Polymyxin-P             GCCTTGCGGATGGTGTTCCGCAAGATGGAACAAGGATTTAGTGCGCGGACCCGCGCGATT 2817
pmxA_Polymyxin-A             GCCTTGCGGATGGTGTTCCGCAAGACGGAACAAGGGTTTAGCGCGCGGAACCGCGCGATT 2727
pmxA_1G_Polymyxin            gccttgcggatggtgttccgcaagacggaacaagggtttagcgcgcggacccgcgcgatt 2784
pmxA-D-DaB3-Polymyxin-B      GCCTTGCGGATGGTGTTCCGCAAGACGGAACAAGGGTTTAGCGCGCGGAACCGCGCGATT 2724
pmxA_B-LR_Polymyxin-E        GCCTTGCGGATGGTGTTCCGCAAAACGGAACAAGGGTTTAGCGAAAGGAACCGCGCGATT 2817
pmxA_KP26_Polymyxin-E        GCCTTGCGGATGGTGTTCCGCAAAACGGAACAAGGGTTTAGCGAAAGGAACCGCGCGATT 2817
                             *********************** * ********* ***** *   *** **********

pmxA_Polymyxin-P             CAGGAAGGCGGGCTGTTCACGCTGGACGTGTTCGACTTCAAGGATGCGGAGAATACCGCG 2877
pmxA_Polymyxin-A             CAGGACGGCGGGTTGTTCACGCTGGACGTGTTCGACTTCAAGGATGCGGAGAATACCGAG 2787
pmxA_1G_Polymyxin            caggacggcgggctgttcacgctggacgtgttcgacttcaaggatgcggagaataccgcg 2844
pmxA-D-DaB3-Polymyxin-B      CAGGAAGGCGGGCTGTTCACGCTGGACGTGTTCGACTTCAAGGATGCGGAGAATACCGCG 2784
pmxA_B-LR_Polymyxin-E        CAGGAAGGCGGGCTGTTCACGCTGGACGTGTTCGACTTCAAGGATGCGGAGGATACCGCA 2877
pmxA_KP26_Polymyxin-E        CAGGAAGGCGGGCTGTTCACGCTGGACGTGTTCGACTTCAAGGATGCGGAGGATACCGCA 2877
                             ***** ****** ************************************** ******

pmxA_Polymyxin-P             CAGGCTGTGGAAGCGAAGGCAACGGACATTCAAGCGGGCATCGATCTGGAGAACAGGGCC 2937
pmxA_Polymyxin-A             CAGGCTGTGGAAGCGAAGGCAACGGACATTCAAGCGGGCATCGATCTGGAGAACGGGCTC 2847
pmxA_1G_Polymyxin            caggctgtggaagcgaaggcaacggacattcaagcgggcatcgatctggagaacgggccc 2904
pmxA-D-DaB3-Polymyxin-B      CAGGCTGTGGAAGCGAAGGGAACGGACATTCAAGCGGGCATCGATCTGGAGAACGGGCCC 2844
pmxA_B-LR_Polymyxin-E        CAGGCTCTGGAAGCGAAGGCAACGGATATTCAAGCGGGCATCGATCTGGAGAAAGGGCCC 2937
pmxA_KP26_Polymyxin-E        CAGGCTCTGGAAGCGAAGGCAACGGATATTCAAGCGGGCATCGATCTGGAGAAAGGGCCC 2937
                             ****** ************ ****** **************************  **  *
```

```
pmxA_Polymyxin-P            CTCGTGAAGGCGGGACTGTTCCGGTGCGCGGACGGCGATCATTTGCTGCTCGCGGTTCAT 2997
pmxA_Polymyxin-A            CTCTTGAAGGCGGGACTGTTCCGGTGCGCGGACGGAGATCATTTGCTGCTCGCGGTTCAT 2907
pmxA_1G_Polymyxin           ctcgtgaaggcgggactgttccggtgcgcggacggcgatcatttgctgctcgcggttcat 2964
pmxA-D-DaB3-Polymyxin-B     CTCGTGAAGGCGGGACTGTTCCGGTGCGCGGACGGCGATCATTTGCTGCTCGCGGTTCAT 2904
pmxA_B-LR_Polymyxin-E       CTCGTGAAGGCGGGACTGTTCCGATGCGCGGACGGCGATCATTTACTGCTCGCGGTTCAT 2997
pmxA_KP26_Polymyxin-E       CTCGTGAAGGCGGGACTGTTCCGATGCGCGGACGGCGATCATTTACTGCTCGCGGTTCAT 2997
                            *** ******************* *********** ******** ***************

pmxA_Polymyxin-P            CATGCCGTGGTGGACGGCGTGTCTTGGCGTATTTTGATGGAGGATTTCGCTCTGGGTTAC 3057
pmxA_Polymyxin-A            CATGCCGTGGTGGATGGCGTGTCTTGGCGCATTTTGATGGAGGATTTCGCTCTGGGTTAC 2967
pmxA_1G_Polymyxin           catgccgtggtggacggcgtgtcttggcgcattttgatggaggatttcgctctgggttac 3024
pmxA-D-DaB3-Polymyxin-B     CATGCCGTGGTGGACGGCGTGTCTTGGCGCATTTTGATGGAGGATTTCGCTCTGGGTTAC 2964
pmxA_B-LR_Polymyxin-E       CATGCCGTGGTGGACGGCGTGTCTTGGCGCATTTTGATGGAGGATTTCGCTCTGGGTTAC 3057
pmxA_KP26_Polymyxin-E       CATGCCGTGGTGGACGGCGTGTCTTGGCGCATTTTGATGGAGGATTTCGCTCTGGGTTAC 3057
                            ************** ************** ******************************

pmxA_Polymyxin-P            GAGCAGGCCGGCAAAAGCGAGGAAATTCGTTTCCCAGCGAAAACGGATGCGTACCGCACT 3117
pmxA_Polymyxin-A            GAGCAGGCCGGCAAAAGCGAGGAAATTCGTTTCCCGGCGAAAACGGATGCGTACCGCACT 3027
pmxA_1G_Polymyxin           gagcaggccggcaaaagcgaggaaattcgtttctcggcgaaaacggatgcgtaccgcact 3084
pmxA-D-DaB3-Polymyxin-B     GAGCAGGCCGGCAAAAGCGAGGAAATTCGTTTCCCGGCGAAAACGGATGCGTACCGCACT 3024
pmxA_B-LR_Polymyxin-E       GAGCAGGCCGGCAAAAGCGAGGAAATTCGTTTCCCGGCGAAAACGGATGCGTACCGCACT 3117
pmxA_KP26_Polymyxin-E       GAGCAGGCCGGCAAAAGCGAGGAAATTCGTTTCCCGGCGAAAACGGATGCGTACCGCACT 3117
                            ********************************* * ************************

pmxA_Polymyxin-P            TGGTCCGAGCAGCTGGCCGCTTACGCGCAAAGCCCGGAGATGGCGAAGGAACGGGCCTAT 3177
pmxA_Polymyxin-A            TGGTCCGAACAGCTGGCCGCTTACGCGCAAAGCCCGGAAATGACGAAGGAACGGGCCTAT 3087
pmxA_1G_Polymyxin           tggtccgagcagttagccgcttacgcgcaaagcccggagatggcgaaggaacgggcctat 3144
pmxA-D-DaB3-Polymyxin-B     TGGTCCGAGCAGCTGGCCGCTTACGCGCAAAGCCCGGAGATGGCGAAGGAACGGGCCTAT 3084
pmxA_B-LR_Polymyxin-E       TGGTCCGAGCAGCTGGCCGCTTACGCGCAAAGCCCGGAGATAGCAAAGGAACGGGCTTAT 3177
pmxA_KP26_Polymyxin-E       TGGTCCGAGCAGCTGGCCGCTTACGCGCAAAGCCCGGAGATAGCAAAGGAACGGGCTTAT 3177
                            ******** *** * *********************** **  * *********** ***

pmxA_Polymyxin-P            TGGCAGGCCGTGGAACAAATTGCGGTTCCGGCCTTGCCGAAGGATCTGGAGGCGGACGTT 3237
pmxA_Polymyxin-A            TGGCAGGCCATGGAACAAATTGCGGTTCCGGCGGTGCCGAAGGATCTGGATGTGGACGTT 3147
pmxA_1G_Polymyxin           tggcaggccgtggaacaaattgcggttccggccgtgccgaaggatctggaggcggacgtt 3204
pmxA-D-DaB3-Polymyxin-B     TGGCAGGCCGTGGAACAAATTGCGGTTCCGGCCGTGCCGAAGGATCTGGAGGCGGACGTT 3144
pmxA_B-LR_Polymyxin-E       TGGCAGGCCGTGGAACAAATTGCGGTTCCGGCCTTGCCGAAGGATCTGGAGGCGGACGTT 3237
pmxA_KP26_Polymyxin-E       TGGCAGGCCGTGGAACAAATTGCGGTTCCGGCCTTGCCGAAGGATCTGGAGGCGGACGTT 3237
                            ********* **********************  **************** * *******

pmxA_Polymyxin-P            ACGACGCAGCAGGACAGCGAATCGCTGTTCGTTCGTTTGACTCCCGAAGAAACGGAGCTG 3297
pmxA_Polymyxin-A            ACGACGCAGCAGGACAGCGAATCGCTGTTCGTCCGTTTGACTCCCGAAGAAACGGAGCTG 3207
pmxA_1G_Polymyxin           acgacgcagcaggacagcgaatcgctgttcgtccgtttgactcccgaagaaacggagctg 3264
```

```
pmxA-D-DaB3-Polymyxin-B      ACGACGCAGCAGGACAGCGAATCGCTGTTCGTCCGTTTGACTCCCGAAGAAACGGAGCTG 3204
pmxA_B-LR_Polymyxin-E        ACGACGCAGCAGGACAGCGAATCGCTGTTCGTCCGTTTGACTTCCGAAGAAACGGAGCTG 3297
pmxA_KP26_Polymyxin-E        ACGACGCAGCAGGACAGCGAATCGCTGTTCGTCCGTTTGACTTCCGAAGAAACGGAGCTG 3297
                             ******************************** ********* *****************

pmxA_Polymyxin-P             CTGCTGAAGCGGGTTCACCGGGCCTACAACACCGAAATGAACGATATTTTGGTAACGGCG 3357
pmxA_Polymyxin-A             CTGCTGAAGCGGGTTCACCGGGCCTACAACACCGAAATGAACGATATTTTGGTAACGGCG 3267
pmxA_1G_Polymyxin            ctgctgaagcgggttcaccgggcctacaacaccgaaatgaacgatattttggtaacggcg 3324
pmxA-D-DaB3-Polymyxin-B      CTGCTGAAGCGGGTTCACCGGGCCTACAACACCGAAATGAACGATATTTTGATAACGGCG 3264
pmxA_B-LR_Polymyxin-E        CTGCTGAAGCGGGTACACCGGGCCTACAACACCGAAATGAACGATATTTTGGTAACGGCG 3357
pmxA_KP26_Polymyxin-E        CTGCTGAAGCGGGTACACCGGGCCTACAACACCGAAATGAACGATATTTTGGTAACGGCG 3357
                             ************** ************************************ ********

pmxA_Polymyxin-P             CTCGGCATAGCCGTTCGCAAGTGGACGGGACACGAACGGGTGCGGATCAATCTCGAAGGA 3417
pmxA_Polymyxin-A             CTCGGCATAGCCATTCGCAAGTGGACGGGACACGAACGGGTGCGGATCAATCTCGAAGGA 3327
pmxA_1G_Polymyxin            ctcggcatagccgttcgcaagtggacgggacacgaacgggtgcggatcaatctcgaagga 3384
pmxA-D-DaB3-Polymyxin-B      CTCGGCATAGCCGTTCGCAAGTGGACGGGACACGAAAGGGTGCGGATCAATCTCGAAGGA 3324
pmxA_B-LR_Polymyxin-E        CTCGGCATAGCTGTTCGCAAGTGGACGGGACACGAACGGGTGCGGATCAATCTCGAAGGA 3417
pmxA_KP26_Polymyxin-E        CTCGGCATAGCTGTTCGCAAGTGGACGGGACACGAACGGGTGCGGATCAATCTCGAAGGA 3417
                             ***********  *********************** ***********************

pmxA_Polymyxin-P             CACGGACGCGAATCGATCGGAACGGATATCGACATCACGCGCACAGTCGGCTGGTTTACG 3477
pmxA_Polymyxin-A             CACGGACGCGAATCGATCGGAACGGATATCGACATCACGCGCACAGTCGGCTGGTTTACG 3387
pmxA_1G_Polymyxin            cacggacgcgaatcgatcggaacggatatcgacatcacgcgcacagtcggctggtttacg 3444
pmxA-D-DaB3-Polymyxin-B      CACGGACGCGAATCAATCGGAACGGATATCGACATCACGCGCACAGTCGGCTGGTTTACG 3384
pmxA_B-LR_Polymyxin-E        CACGGACGCGAATCGATCGGAACGGATATCGACATCACGCGCACAGTCGGCTGGTTTACG 3477
pmxA_KP26_Polymyxin-E        CACGGACGCGAATCGATCGGAACGGATATCGACATCACGCGCACAGTCGGCTGGTTTACG 3477
                             ************** *********************************************

pmxA_Polymyxin-P             ACCAAGTTTCCGGTCGTCCTGGAGCCGGAAACCGACCGGGATTTGGCCTATCAGATTAAA 3537
pmxA_Polymyxin-A             ACCAAGTTTCCGGTCGTCCTGGAGCCGGAAACCGACCGGGATTTGGCCTATCAGATTAAA 3447
pmxA_1G_Polymyxin            accaagtttccggtcgtcctggagccagaaaccgaccgggatttggcctatcagattaaa 3504
pmxA-D-DaB3-Polymyxin-B      ACCAAGTTTCCGGTCGTCCTGGAGCCGGAAACCGACCGGGATTTGGCCTATCAGATTAAA 3444
pmxA_B-LR_Polymyxin-E        ACCAAGTTTCCGGTCGTCCTGGAGCCGGAAACCAACCGGGATTTGGCCTATCAGATTAAA 3537
pmxA_KP26_Polymyxin-E        ACCAAGTTTCCGGTCGTCCTGGAGCCGGAAACCAACCGGGATTTGGCCTATCAGATTAAA 3537
                             ************************** ****** **************************

pmxA_Polymyxin-P             CAGGTCAAGGAAAGCTTGCGCCGCATTCCGAACAAGGGGCTTGGGTACGGCGTATGCCGC 3597
pmxA_Polymyxin-A             CAGGTCAAGGAAAGCTTGCGCCGCATTCCGAACAAGGGGCTTGGGTACGGCGTATGCCGC 3507
pmxA_1G_Polymyxin            caggtcaaggaaagcttgcgccgcattccgaacaaggggcttgggtacggcgtatgccgc 3564
pmxA-D-DaB3-Polymyxin-B      CAGGTCAAGGAAAGCTTGCGCCGCATTCCGAACAAGGGGCTTGGGTACGGCGTATGCCGC 3504
pmxA_B-LR_Polymyxin-E        CAGGTCAAGGAAAGCTTGCGTCGCATTCCGAACAAGGGGCTTGGGTACGGTGTATGCCGC 3597
pmxA_KP26_Polymyxin-E        CAGGTCAAGGAAAGCTTGCGTCGCATTCCGAACAAGGGGCTTGGGTACGGTGTATGCCGC 3597
                             ******************** ***************************** *********
```

```
pmxA_Polymyxin-P            TATCTCTCCAAATCGGAGGATGGCTTTGTTTGGGGCTCAGAGCCGGAAATTAATTTTAAC 3657
pmxA_Polymyxin-A            TATCTCTCCAAATCGGAGGATGGCTTTGTTTGGGGCGCAGAGCCGGAAATTAATTTTAAC 3567
pmxA_1G_Polymyxin           tatctctccaaatcggaggatggctttgtttggggcgcagagccggaaattaattttaac 3624
pmxA-D-DaB3-Polymyxin-B     TATCTCTCCAAATCGGAGGATGGCTTTGTTTGGGGCGCAGAGCCGGAAATTAATTTTAAC 3564
pmxA_B-LR_Polymyxin-E       TATCTCTCCAAATCGGAGGATGGCTTTGTTTGGGGCGCAGAGCCGGAAATTAATTTTAAC 3657
pmxA_KP26_Polymyxin-E       TATCTCTCCAAATCGGAGGATGGCTTTGTTTGGGGCGCAGAGCCGGAAATTAATTTTAAC 3657
                            *********************************** ************************

pmxA_Polymyxin-P            TACCTCGGCCAGTTCGACGATGATGTCAATCAGGACGAGATCGGCATATCTTCTTATTCC 3717
pmxA_Polymyxin-A            TACCTCGGCCAGTTCGACGATGATGTCAGTCAGGTCGAGATCGGCATATCTTCTTATTCC 3627
pmxA_1G_Polymyxin           tacctcggccagttcgacgatgatgtcaatcaggacgagatcggcatatcttcttattcc 3684
pmxA-D-DaB3-Polymyxin-B     TACCTCGGCCAGTTCGACGATGATGTCAACCAGGACGAGATCGGCATATCTTCTTATTCC 3624
pmxA_B-LR_Polymyxin-E       TACCTCGGCCAGTTCGACGATGATGTCAACCAGGACGAGATCGGCATATCTTCTTATTCC 3717
pmxA_KP26_Polymyxin-E       TACCTCGGCCAGTTCGACGATGATGTCAACCAGGACGAGATCGGCATATCTTCTTATTCC 3717
                            ****************************  **** *************************

pmxA_Polymyxin-P            AGCGGCAGCCCGGCCAGCGACCGGCAGGCCCGCAGCTTTGTGCTGGATATCAACGGCATG 3777
pmxA_Polymyxin-A            AGCGGCAGCCCGGCCAGCGACCGGCAGGCCCGCAGCTTTGTGCTGGATATCAACGGCATG 3687
pmxA_1G_Polymyxin           agcggcagcccggccagcgaccggcaggcccgcagctttgtgctggatatcaacggcatg 3744
pmxA-D-DaB3-Polymyxin-B     AGCGGCAGCCCGGCCAGCGACCGGCAGGCCCGCAGCTTTGTGCTGGATATCAACGGCATG 3684
pmxA_B-LR_Polymyxin-E       AGCGGCAGCCCGGCCAGCGACCGGCAGGCCCGCAGCTTTGTGCTGGATATCAACGGCATG 3777
pmxA_KP26_Polymyxin-E       AGCGGCAGCCCGGCCAGCGACCGGCAGGCCCGCAGCTTTGTGCTGGATATCAACGGCATG 3777
                            ************************************************************

pmxA_Polymyxin-P            GTGCTGGAAGGCGCTTTATCGCTCGATCTCAGCTACAGCCGGAAGCAGTATCACAAGGAA 3837
pmxA_Polymyxin-A            GTGCTGGACGGCGCTTTATCGCTCGATCTCAGCTACAGCCGGAAGCAGTATCGCAAGGAA 3747
pmxA_1G_Polymyxin           gtgctggacggcactttatcgctcgatctcagctacagccggaagcagtatcgcaaggaa 3804
pmxA-D-DaB3-Polymyxin-B     GTGCTGGACGGCGCTTTATCGCTCGATCTCAGCTACAGCCGGAAGCAGTATCGCAAGGAA 3744
pmxA_B-LR_Polymyxin-E       GTGCTGGACGGCGCTCTATCGCTCGATCTCAGCTACAGCCGGAAGCAGTATCGCAAGGTA 3837
pmxA_KP26_Polymyxin-E       GTGCTGGACGGCGCTCTATCGCTCGATCTCAGCTACAGCCGGAAGCAGTATCGCAAGGTA 3837
                            ******** *** ** ************************************ ***** *

pmxA_Polymyxin-P            ACGATGGAAGCCTTCGCTCAGCGGCTTGAGCAAAGTCTTCGAGAGCTCATTACCCACTGC 3897
pmxA_Polymyxin-A            ACGATGGAAGCCTTCGCTCAGCGGCTTGAGCAAAGTCTGCGAGAGCTCATTACCCACTGC 3807
pmxA_1G_Polymyxin           acgatggaagccttcgctcagcggcttgagcaaagtctccgagagctcattacccactgc 3864
pmxA-D-DaB3-Polymyxin-B     ACGATGGAAGCCTTCGCTCAGCGGCTTGAGCAAAGTCTCCGAGAGCTCATTACCCACTGC 3804
pmxA_B-LR_Polymyxin-E       ACGATGGAAGCCTTCGCTCAGCGGCTTGAGCAAAGTCTCCGAGAGCTCATTACCCACTGC 3897
pmxA_KP26_Polymyxin-E       ACGATGGAAGCCTTCGCTCAGCGGCTTGAGCAAAGTCTCCGAGAGCTCATTACCCACTGC 3897
                            ************************************** *********************

pmxA_Polymyxin-P            GCAGGCAAAGAAAACACCGAATTGACGCCGAGCGACGTGCAATTTAAAGGCTTGACCATC 3957
pmxA_Polymyxin-A            GCAGGCAAAGAAAACACCGAATTGACGCCGAGCGACGTGCAATTTAAAGGCTTGACCATC 3867
pmxA_1G_Polymyxin           gcaggcaaagaaaacaccgaattgacgccgagcgacgtgcaatttaaaggcttgaccatc 3924
```

```
pmxA-D-DaB3-Polymyxin-B      GCAGGCAAAGAAAACACCGAATTGACGCCGAGCGACGTGCAATTTAAAGGCTTGACCATC 3864
pmxA_B-LR_Polymyxin-E        GCAGGCAAAGAAAACACCGAATTGACGCCTAGCGACGTGCAATTTAAAGGCTTGACCATC 3957
pmxA_KP26_Polymyxin-E        GCAGGCAAAGAAAACACCGAATTGACGCCTAGCGACGTGCAATTTAAAGGCTTGACCATC 3957
                             ***************************** ******************************

pmxA_Polymyxin-P             GAGGAATTGGAGCAAATCGCCCAGCGCTCGGGACATCTCGGGGAAATCGAAAATATTTAC 4017
pmxA_Polymyxin-A             GCGGAATTGGAGCAAATCGCCCAGCGCTCGGGCCATCTCGGGGAAATCGAAAATATTTAC 3927
pmxA_1G_Polymyxin            gcggaattggagcaaatcgcccagcgctcgggccatctcggggaaatcgaaaatatttac 3984
pmxA-D-DaB3-Polymyxin-B      GCGGAATTGGAGCAAATCGCCCAGCGCTCGGGCCATCTCGGGGAAATCGAAAATATTTAC 3924
pmxA_B-LR_Polymyxin-E        GCGGAATTGGAGCAAATCGGCCAGCGCTCGGTCCATGTCGGGGAAATCGAGAATATTTAC 4017
pmxA_KP26_Polymyxin-E        GCGGAATTGGAGCAAATCGGCCAGCGCTCGGTCCATGTCGGGGAAATCGAGAATATTTAC 4017
                             * ***************** ***********  *** ************* *********

pmxA_Polymyxin-P             TCGCTTACGCCGATGCAGAAGGGCATGTGGTTCCACAGTGCGCTTGACCAGCAAACGGCA 4077
pmxA_Polymyxin-A             TCGCTTACGCCGATGCAGAAGGGCATGTGGTTCCACAGCGCGCTTGACCGGCAAACTGCC 3987
pmxA_1G_Polymyxin            tcgcttacgccgatgcagaagggcatgtggttccacagcgcgcttgaccggcaaactgcc 4044
pmxA-D-DaB3-Polymyxin-B      TCGCTTACGCCGATGCAGAAGGGCATGTGGTTCCACAGCGCGCTTGACCGACAAACGGCC 3984
pmxA_B-LR_Polymyxin-E        TCGCTTACGCCGATGCAGAAGGGCATGTGGTTCCACAGCGCGCTTGACCGGCAGACGGCC 4077
pmxA_KP26_Polymyxin-E        TCGCTTACGCCGATGCAGAAGGGCATGTGGTTCCACAGCGCGCTTGACCGGCAGACGGCC 4077
                             ************************************** **********  ** ** ** 

pmxA_Polymyxin-P             GCTTACTTCGAGCAGACGCAGTTTACGATGCGGGGAGCGCTCGACGTCCAGCTTTTCGAG 4137
pmxA_Polymyxin-A             GCTTACTTCGAGCAGACGCGGTTTACGATGCGGGGAGCGCTCGACGTCCAGCTTTTCGAG 4047
pmxA_1G_Polymyxin            gcttacttcgagcagacgcggtttacgatgcggggagcgctcgacgtccagcttttcgag 4104
pmxA-D-DaB3-Polymyxin-B      GCTTACTTCGAGCAGACGCGGTTTACGATGCGGGGAGCGCTCGACGTCCAGCTTTTCGAG 4044
pmxA_B-LR_Polymyxin-E        GCTTACTTCGAGCAGACGCGGTTTACGATGCGGGGAGCGCTCGACGTTCAGCTTTTCGAG 4137
pmxA_KP26_Polymyxin-E        GCTTACTTCGAGCAGACGCGGTTTACGATGCGGGGAGCGCTCGACGTTCAGCTTTTCGAG 4137
                             ******************* *************************** ************

pmxA_Polymyxin-P             AGGAGCTGGATGGAGCTTGCAAAACGTCATCTGGTGCTACGGGCGAATTTTGTGAAAGGA 4197
pmxA_Polymyxin-A             AGGAGCTGGATGGAGCTTGCGAAACGTCATCTGGTGCTGCGGGCGAATTTTGTGAAAGGA 4107
pmxA_1G_Polymyxin            aggagctggatggagcttgcgaaacgtcatctggtgctgcgggcgaattttgtgaaagga 4164
pmxA-D-DaB3-Polymyxin-B      AGGAGCTGGATGGAGCTTGCGAAACGTCATCTGGTGCTGCGGGCGAATTTTGTGAAAGGA 4104
pmxA_B-LR_Polymyxin-E        AGGAGCTGGACGGAGCTTGCGAAACGTCATCTGGTGCTGCGGGCGAATTTTGTGAAAGGA 4197
pmxA_KP26_Polymyxin-E        AGGAGCTGGACGGAGCTTGCGAAACGTCATCTGGTGCTGCGGGCGAATTTTGTGAAAGGA 4197
                             ********** ********* ***************** *********************

pmxA_Polymyxin-P             CCAACGGGCGAGCCGCTGCAAATCATATACCGCGACAAGCCGGTCGGCTTTGAATATGAA 4257
pmxA_Polymyxin-A             CCGGAGGGCGAGCCGCTGCAAGTCATATACCGCGACAAGCCGGTCGGCTTTGAATATGAA 4167
pmxA_1G_Polymyxin            ccggcgggcgagccgctgcaaatcatataccgcgacaagccggtcggctttgaatatgaa 4224
pmxA-D-DaB3-Polymyxin-B      CCGGCGGGCGAGCCGCTGCAAATCATATACCGCGACAAGCCGGTCGGCTTTGAATATGAA 4164
pmxA_B-LR_Polymyxin-E        CCAGCGGGCGAGCCGCTGCAGATCATATACCGCGACAAACCAGTCGGCTTTGAATATGAA 4257
pmxA_KP26_Polymyxin-E        CCAGCGGGCGAGCCGCTGCAGATCATATACCGCGACAAACCAGTCGGCTTTGAATATGAA 4257
                             **   ***************  **************** ** ******************
```

```
pmxA_Polymyxin-P             GAGCTGCTTCATTTGCAGTCGGACGAGAAACAAGCGTACTTGGATAAAAAAGCCGAGGAT 4317
pmxA_Polymyxin-A             GAGCTGCTTCATTTGCAGGCGGACGAGAAACAAGCGTATTTGGATAAAAAGGCCGAGGAT 4227
pmxA_1G_Polymyxin            gagctgcttcatttgcaggtggacgagaaacaagcgtatttggataaaaaggccgaggat 4284
pmxA-D-DaB3-Polymyxin-B      GAGCTGCTTCATTTGCAGGCGGACGAGAAACAAGCGTATTTGGATAAAAAGGCCGAGGAT 4224
pmxA_B-LR_Polymyxin-E        GAGCTGCTACATTTGCAGGCGGACGAGAAACAAGCGTATTTGGATAAAAAGGCCGAGGAT 4317
pmxA_KP26_Polymyxin-E        GAGCTGCTACATTTGCAGGCGGACGAGAAACAAGCGTATTTGGATAAAAAGGCCGAGGAT 4317
                             ******** *********  ****************** *********** *********

pmxA_Polymyxin-P             GACAAGCTTCGCGGCTTCGACCTGGAACATGACGCGCTCGTTCGGGTTACGATCCTGCGC 4377
pmxA_Polymyxin-A             GACAAGCTTCGCGGCTTCGACATGGAACATGACGCGCTCGTTCGGGTTACGATCTTGCGC 4287
pmxA_1G_Polymyxin            gacaagcttcgcggcttcgacctggaacatgacgcgctcgttcgggttacgatcctgcgc 4344
pmxA-D-DaB3-Polymyxin-B      GACAAGCTTCGCGGCTTCGACATGGAAAATGACGCGCTCGTTCGGGTTACGATCCTGCGC 4284
pmxA_B-LR_Polymyxin-E        GACAAGCTTCGCGGCTTCGACCTGGAACATGACGCGCTCGTTCGGGTTACGATCCTGCGC 4377
pmxA_KP26_Polymyxin-E        GACAAGCTTCGCGGCTTCGACCTGGAACATGACGCGCTCGTTCGGGTTACGATCCTGCGC 4377
                             ********************* ***** ************************** *****

pmxA_Polymyxin-P             ACCGAAGAGCAAAGCTATCATGTGCTGTGGAGTTTCCAGCATATTTTGATGGACGGCTGG 4437
pmxA_Polymyxin-A             ACCGAAGAGCAAAGCTATCATGTGCTGTGGAGTTTCCAGCATATTTTGATGGACGGCTGG 4347
pmxA_1G_Polymyxin            accgaagagcaaagctatcatgtgctgtggagtttccagcatattttgatggacggctgg 4404
pmxA-D-DaB3-Polymyxin-B      ACCGAAGAGCAAAGCTATCATGTGCTGTGGAGTTTCCAGCATATTTTGATGGACGGCTGG 4344
pmxA_B-LR_Polymyxin-E        ACCGAAGAGCAGAGCTATCATGTGCTGTGGAGTTTCCAGCATATTTTGATGGACGGCTGG 4437
pmxA_KP26_Polymyxin-E        ACCGAAGAGCAGAGCTATCATGTGCTGTGGAGTTTCCAGCATATTTTGATGGACGGCTGG 4437
                             *********** ************************************************

pmxA_Polymyxin-P             TGCCTACCGCAGCTGACGCAGGAGCTGTTTGAGACGTACTCGGCCTTGGCATCCGGCAAG 4497
pmxA_Polymyxin-A             TGCCTGCCGCAGCTGACGCAGGAGCTGTTTGAGATGTACTCGGCCTTGGCATCCGGCAAG 4407
pmxA_1G_Polymyxin            tgcctggcgcagctgacgcaggagctgtttgagacgtactcgaccttggcatccggcaag 4464
pmxA-D-DaB3-Polymyxin-B      TGCCTGCCGCAGCTGACGCAGGAGCTGTTTGAGACGTACTCGGCCTTGGCATCCGGCAAG 4404
pmxA_B-LR_Polymyxin-E        TGCCTGCCGCAACTGACGCAGGAGCTGTTTGAGACATACTCGGCCTTGGCATCCGGCAAG 4497
pmxA_KP26_Polymyxin-E        TGCCTGCCGCAACTGACGCAGGAGCTGTTTGAGACATACTCGGCCTTGGCATCCGGCAAG 4497
                             *****  **** **********************  ****** *****************

pmxA_Polymyxin-P             CAGCCAGCGGGAGATAAGGGATCGGATTATGGCGCCTATATCGAATGGTTGGAGAAACAG 4557
pmxA_Polymyxin-A             CAGCCAGCGGGAGATAAGGGATCGGATTATGGCGCTTATATCGAATGGTTGGAGAAACAG 4467
pmxA_1G_Polymyxin            cagccagcgggagataagggatcggattatggcgcctatatcgaatggttggagaaacag 4524
pmxA-D-DaB3-Polymyxin-B      CAGCCAGCGGGAAATAAGGGATCGGATTATGGCGCTTATATCGAATGGTTGGAGAAACAG 4464
pmxA_B-LR_Polymyxin-E        CAGCCAGCGGGAGATAAGGGGTCGGATTATGGCGCTTATATCGAATGGCTGGAGAAACAG 4557
pmxA_KP26_Polymyxin-E        CAGCCAGCGGGAGATAAGGGGTCGGATTATGGCGCTTATATCGAATGGCTGGAGAAACAG 4557
                             ************ ******* ************** ************ ***********

pmxA_Polymyxin-P             GACGATCAGGCGGCATCCGGCTATTGGACGGCATTCCTGGCAGGTTATGAAGGGCAAACC 4617
pmxA_Polymyxin-A             GACGATCAGGCGGCATCCGGCTATTGGACGGCATTCCTGGCAGATTATGAAGGGCAAACC 4527
pmxA_1G_Polymyxin            gacgatcaggtggcatccggctattggacggcattcctgtcaggttatgaagggcaaact 4584
```

```
pmxA-D-DaB3-Polymyxin-B      GACGATCAGGCGGCATCCGGCTATTGGACGGCATTCCTGGCAGGTTATGAAGGGCAAACC 4524
pmxA_B-LR_Polymyxin-E        GACGATCAGGCGGCATCCGGCTATTGGACGGCGTTCCTGGCAGGTTACGAAGGGCAAACT 4617
pmxA_KP26_Polymyxin-E        GACGATCAGGCGGCATCCGGCTATTGGACGGCGTTCCTGGCAGGTTACGAAGGGCAAACT 4617
                             ********** ******************** ****** *** *** ***********

pmxA_Polymyxin-P             GTACTCCCGGGACAAAAGGAACCGGCGCCGAACGGCAGATTTACGGCTGATCACGTCACC 4677
pmxA_Polymyxin-A             GTACTCCCGGGACAAAAGGAACCGGCGCCAAGCGGCATATTTACGGCTGATCACGTCACC 4587
pmxA_1G_Polymyxin            gtactcccgggacaaaaggaaccggcgctgaacggcagatttacggctgatcacgtcacc 4644
pmxA-D-DaB3-Polymyxin-B      GTACTCCCGGGACAAAAGGAACCGGCGCCAAACGGCAGATTTACGGCTGATCACGTCACC 4584
pmxA_B-LR_Polymyxin-E        GTACTCCCGGGGCAAAAGGAAGCGCAGCCGAACGGTAGATTTACGGCTGATCACGTCACC 4677
pmxA_KP26_Polymyxin-E        GTACTCCCGGGGCAAAAGGAAGCGCAGCCGAACGGTAGATTTACGGCTGATCACGTCACC 4677
                             *********** ********* **  **  * *** * **********************

pmxA_Polymyxin-P             GCCGAGCTAGGCAAAGACTTGAGCGAGCGGATGGACCAGGTGGCGAAACAGCGCCTGGTT 4737
pmxA_Polymyxin-A             GCCGAGCTGGGCAAGGACTTGAGCGAGCGGATGGACCGGGTGGCGAAACAGCGCCTGGTT 4647
pmxA_1G_Polymyxin            gccgagctgggcaaggagttgagcgagcggatggatcgggtggcgaaagagcgcctggtt 4704
pmxA-D-DaB3-Polymyxin-B      GCCGAGCTGGGCAAGGACTTGAGCGAGCGGATGGACCGGGTGGCGAAACAGCGCCTGGTT 4644
pmxA_B-LR_Polymyxin-E        GCCGAGCTGGGCAAGGACTTGAGCGAGCGGATGGATCGGGTGGCGAAACAGCGCCTGGTT 4737
pmxA_KP26_Polymyxin-E        GCCGAGCTGGGCAAGGACTTGAGCGAGCGGATGGATCGGGTGGCGAAACAGCGCCTGGTT 4737
                             ******** ***** ** ***************** * ********** ***********

pmxA_Polymyxin-P             ACGGTCAATACGTTGCTGCAAGCCGCATGGGGCGTGATGCTGCAAAAATATAACGGGACA 4797
pmxA_Polymyxin-A             ACGGTCAATACGCTGCTGCAAGCCGCTTGGGGCGTGATGCTGCAAAAATATAACGGAACA 4707
pmxA_1G_Polymyxin            acggtcaatacgctgttgcaagccgcttggggcgtgatgctgcaaaaatataacggaaca 4764
pmxA-D-DaB3-Polymyxin-B      ACGGTCAATACGCTGCTGCAAGCCGCTTGGGGCGTGATGCTGCAAAAATATAACGGAACA 4704
pmxA_B-LR_Polymyxin-E        ACAGTCAATACGCTGCTGCAAGCCGCTTGGGGCGTGATGCTGCAAAAATATAACGGAACA 4797
pmxA_KP26_Polymyxin-E        ACAGTCAATACGCTGCTGCAAGCCGCTTGGGGCGTGATGCTGCAAAAATATAACGGAACA 4797
                             ** ********* ** ********** ***************************** ***

pmxA_Polymyxin-P             AACGATGCCGTATTCGGCAGCGTCGTGGCCGGAAGACCGGCGGAAATCCCGGGCATCGAG 4857
pmxA_Polymyxin-A             AACGATGCCGTATTCGGCAGCGTCGTGGCCGGAAGACCGGCGGAAATCCCGGGCATTGAG 4767
pmxA_1G_Polymyxin            aacgatgccgtattcggcagcgtcgtggccggaagaccggtggaaatcccgggcatcgag 4824
pmxA-D-DaB3-Polymyxin-B      AACGATGCCGTATTCGGCAGCGTCGTGGCCGGAAGACCGGCGGAAATCCCGGGCATCGAG 4764
pmxA_B-LR_Polymyxin-E        AACGATGCCGTATTCGGCAGCGTCGTGGCCGGAAGACCGGCGGAAATCCCGGGTATAGAG 4857
pmxA_KP26_Polymyxin-E        AACGATGCCGTATTCGGCAGCGTCGTGGCCGGAAGACCGGCGGAAATCCCGGGTATAGAG 4857
                             **************************************** ************ ** ***

pmxA_Polymyxin-P             TCCATGATCGGGCTGTTCATCAATACGGTGCCGGTCCGCGTCACGAGCGAAGCGGACACC 4917
pmxA_Polymyxin-A             TCCATGATCGGGCTGTTCATCAATACGGTGCCGGTCCGCGTCACGAGCGAAGCGGACACC 4827
pmxA_1G_Polymyxin            tccatgatcgggctgttcatcaatacggtgccggtccgcgtcacgagcgaagccgacacc 4884
pmxA-D-DaB3-Polymyxin-B      TCCATGATCGGGCTGTTCATCAATACGGTGCCGGTCCGCGTCACGAGCGAAGCGGACACT 4824
pmxA_B-LR_Polymyxin-E        TCCATGATTGGACTGTTTATCAATACGGTGCCGGTTCGCGTCACGAGCGAAGCGGACACC 4917
pmxA_KP26_Polymyxin-E        TCCATGATTGGACTGTTTATCAATACGGTGCCGGTTCGCGTCACGAGCGAAGCGGACACC 4917
                             ******** ** ***** ***************** ***************** *****
```

```
pmxA_Polymyxin-P             GTGTTCGCCGACCTGATGGCGAAGCTCCAAGAGCGGGCGCTGGAGTCCGGGCGTTATGAT 4977
pmxA_Polymyxin-A             GTGTTCGCCGACCTGATGGCGAAGCTCCAAGAGCGGGCGCTGGAGTCCGGGCGATATGAT 4887
pmxA_1G_Polymyxin            gtgttcgccgatatgatggcgaagctccaagagcgtgcgctggagtccgggcgatatgat 4944
pmxA-D-DaB3-Polymyxin-B      GTCTTCGCCGACCTGATGGCGAAGCTCCAAGAGCGGGCGCTGGAGTCCGGGCGTTATGAT 4884
pmxA_B-LR_Polymyxin-E        GTGTTCGCTGACCTGATGGCAAAGCTCCAAGAGCGGGCGCTGGAGTCCGGGCGTTATGAT 4977
pmxA_KP26_Polymyxin-E        GTGTTCGCTGACCTGATGGCAAAGCTCCAAGAGCGGGCGCTGGAGTCCGGGCGTTATGAT 4977
                             ** ***** **  ******* ************** ***************** ******

pmxA_Polymyxin-P             TACTATCCGCTGTATGAAATTCAAGCCCGCAGCGTGCAAAAGCAGAACCTAATCAACCAT 5037
pmxA_Polymyxin-A             TACTATCCGCTGTATGAAATTCAAGCCCGCAGCGTGCAAAAGCAGAACCTGATCAACCAT 4947
pmxA_1G_Polymyxin            tactatccgctgtatgaaattcaagcccgcagcgtgcaaaagcagaacctgatcaaccat 5004
pmxA-D-DaB3-Polymyxin-B      TACTATCCGCTGTATGAAATTCAAGCCCGCAGCGTGCAAAAGCAGAACCTGATCAACCAT 4944
pmxA_B-LR_Polymyxin-E        TACTATCCGCTGTATGAAATTCAAGCCCGCTGCGTGCAAAAGCAAAACCTGATCAACCAT 5037
pmxA_KP26_Polymyxin-E        TACTATCCGCTGTATGAAATTCAAGCCCGCTGCGTGCAAAAGCAAAACCTGATCAACCAT 5037
                             ****************************** ************* ***** *********

pmxA_Polymyxin-P             ATCATCGCTTTCGAGAACTATCCGGTGGACGAGCAGATGGAGCAGGCGGGCGACCAGCAG 5097
pmxA_Polymyxin-A             ATCATCGCTTTCGAGAACTATCCGGTGGACGAGCAGATGGAGCAGGCGGGCGACCAGCAG 5007
pmxA_1G_Polymyxin            atcatcgctttcgagaactatccggtggacgagcagatggagcaggcgggcgaccagcag 5064
pmxA-D-DaB3-Polymyxin-B      ATCATCGCTTTCGAGAACTATCCAGTGGACGAGCAGATGGAGCAGGCGGGCGACCAGCAG 5004
pmxA_B-LR_Polymyxin-E        ATCATCGCTTTCGAGAACTATCCGGTGGATGAGCAGATGGAGCAGGCGGGCGACCAGCAG 5097
pmxA_KP26_Polymyxin-E        ATCATCGCTTTCGAGAACTATCCGGTGGATGAGCAGATGGAGCAGGCGGGCGACCAGCAG 5097
                             *********************** ***** ******************************

pmxA_Polymyxin-P             CACGGCGACCTGACGATCGCTGACGTTCAGATGGAGGAGCAGACGAACTATAACTTCAAC 5157
pmxA_Polymyxin-A             CACGGCGACCTGACGATCGCTGACGTTCAGATGGAGGAGCAGACGAACTATAACTTCAAC 5067
pmxA_1G_Polymyxin            cacggcgacctgacgatcgctgacgttcagatggaggagcagacgaactataacttcaac 5124
pmxA-D-DaB3-Polymyxin-B      CACGGCGACCTTACGATCGCTGACGTTCAGATGGAGGAGCAGACGAACTATAACTTCAAC 5064
pmxA_B-LR_Polymyxin-E        CACGGCGACCTGACGATCACTGACGTTCAGATGGAGGAGCAGACGAACTATAACTTCAAT 5157
pmxA_KP26_Polymyxin-E        CACGGCGACCTGACGATCACTGACGTTCAGATGGAGGAGCAGACGAACTATAACTTCAAT 5157
                             *********** ****** **************************************** 

pmxA_Polymyxin-P             GTGACCGTGGTGCCGGGAGTCGAGATCGAAATTCGGTTCGACTTTAACGCCGAAGTGTTC 5217
pmxA_Polymyxin-A             GTGACCGTGGTGCCGGGAGTCGAGATCGAAATTCGGTTCGACTTTAACGCTGAAGTGTTC 5127
pmxA_1G_Polymyxin            gtgaccgtggtgccgggagccgagatcgaaattcggttcgactttaacgccgaagtgttc 5184
pmxA-D-DaB3-Polymyxin-B      GTGACCGTGGTGCCGGGAGTCGAGATCGAAATTCGATTCGATTTTAACGCCGAAGTGTTC 5124
pmxA_B-LR_Polymyxin-E        GTGACCGTGGTGCCAGGAGCCGAGATCGAAATTCGGTTCGATTTTAACGCCGAAGTGTTT 5217
pmxA_KP26_Polymyxin-E        GTGACCGTGGTGCCAGGAGCCGAGATCGAAATTCGGTTCGATTTTAACGCCGAAGTGTTT 5217
                             ************** **** *************** ***** ******** ******** 

pmxA_Polymyxin-P             GATAAAGACAGCATCGAGCGGCTCAAGGGGCATCTCGTCCATCTGCTGGAGCAGGTGACG 5277
pmxA_Polymyxin-A             GATAAAGACAGCATCGAGCGGCTCAAGGGACACCTCGTCCATCTGCTGGAGCAGGTGACG 5187
pmxA_1G_Polymyxin            gataaagacagcatcgagcggctcaaggggcatctcgtccatctgctggagcaagtgacg 5244
```

```
pmxA-D-DaB3-Polymyxin-B       GATAAAGACAGCATCGAGTTGCTCAAGGGGCATCTCGTCCATCTGCTGGAGCAGGTGACG 5184
pmxA_B-LR_Polymyxin-E         GATAAAGACAGCATCGAACGGCTCAAGGGGCATCTCGTCCATCTGCTGGAGCAGGTGACG 5277
pmxA_KP26_Polymyxin-E         GATAAAGACAGCATCGAACGGCTCAAGGGGCATCTCGTCCATCTGCTGGAGCAGGTGACG 5277
                              *****************   ********* ** ******************** ******

pmxA_Polymyxin-P              GATAACCCGGAAATTACAGTGGGCGAGCTGGAACTTGTGACGGAGGCGGAAAAAACCGAC 5337
pmxA_Polymyxin-A              GATAACCCGGAAATTACAGTGGGCGAGCTGGAACTTGTGACGGAGGGGGAAAAGGCCGAC 5247
pmxA_1G_Polymyxin             gataacccggaaattaccgtgggcgagctggaactagtgacggaggcggaaaatgccgac 5304
pmxA-D-DaB3-Polymyxin-B       GATAACCCGGAAATTACCGTGGGCGAGCTGGAACTTGTGACGGAGGCGGAAAAGGCCGAC 5244
pmxA_B-LR_Polymyxin-E         GATAACCCGGAAATTACCGTGGGAGAGCTGGAACTTGTGACGGAGGCGGAAAAGGCCGAC 5337
pmxA_KP26_Polymyxin-E         GATAACCCGGAAATTACCGTGGGAGAGCTGGAACTTGTGACGGAGGCGGAAAAGGCCGAC 5337
                              ***************** ***** *********** ********** ******  *****

pmxA_Polymyxin-P              CTTCTCGGACGTTTTAACGACACCATCACGGAATTTCCGCGCGGGAAGACGCTCATTCAA 5397
pmxA_Polymyxin-A              CTTCTCGGACGTTTTAACGACACCACCACGGAATTTCCGCGCGGGAAGACGCTCGTTCAA 5307
pmxA_1G_Polymyxin             cttctcggacgttttaacgacaccaccacggaatttccgcgcgggaagacgctcgttcaa 5364
pmxA-D-DaB3-Polymyxin-B       CTTCTCGGACGTTTTAACGACACCACCACGGAATTTCCGCGCGGGAAGACGCTCATTCAA 5304
pmxA_B-LR_Polymyxin-E         CTTCTCGGACGATTTAACGACACCACCACGGAATTTCCGCGCGGGAAGACGCTCATTCAA 5397
pmxA_KP26_Polymyxin-E         CTTCTCGGACGATTTAACGACACCACCACGGAATTTCCGCGCGGGAAGACGCTCATTCAA 5397
                              *********** ************* **************************** *****

pmxA_Polymyxin-P              TTGTTCGAAGAGCAGGCGGAGCTTTACCCGGACAACGTGGCCGCCGTCATGAACGAGCGG 5457
pmxA_Polymyxin-A              TTGTTCGAAGAACAGGCGGAGCTTTACCCGGACAACGTGGCCGCCGTCATGAACGAGCGG 5367
pmxA_1G_Polymyxin             ttgttcgaagagcaggcggagctttacccagacaacgtggccgccgtcatgaacgagcgg 5424
pmxA-D-DaB3-Polymyxin-B       TTGTTCGAAGAGCAGGCGGAGCGCATCCCGGATGCAGCCGCCATCTCCTTGAATGAGCAA 5364
pmxA_B-LR_Polymyxin-E         TTGTTCGAAGAGCAGGTAGAGCGCATCCCGGATGCAGCCGCCATCACCTTGAATGAGCAA 5457
pmxA_KP26_Polymyxin-E         TTGTTCGAAGAGCAGGTAGAGCGCATCCCGGATGCAGCCGCCATCACCTTGAATGAGCAA 5457
                              *********** ****  ****    *** **    *  ***  *  * **** ****  

pmxA_Polymyxin-P              CAGCTGACATACCGCGAGCTGAACGAGCGATCCAACTGCCTTGCGCGGAAGCTGCGGGAG 5517
pmxA_Polymyxin-A              CAACTGACCTACCGCGAGCTGAACGAGCGATCCAACCGCCTTGCGCGGAAGCTGCGAGAG 5427
pmxA_1G_Polymyxin             cagctgacttaccgcgagctgaacgagcgatccaaccgccttgcgcggaagctgcgggag 5484
pmxA-D-DaB3-Polymyxin-B       GAGCTGACCTACCGCGAGCTGAACGAACGCGTTAACCGCCTTGCCCGTACCTTGCGTAGC 5424
pmxA_B-LR_Polymyxin-E         GAGCTGACCTACCGCGAGCTCAACGAACGTGTCAACCGCCTTGCCCGTACCTTGCGTAGC 5517
pmxA_KP26_Polymyxin-E         GAGCTGACCTACCGCGAGCTCAACGAACGTGTCAACCGCCTTGCCCGTACCTTGCGTAGC 5517
                               * ***** *********** ***** **    *** ******* ** *   ****    

pmxA_Polymyxin-P              ACGGGAGTAGAAGCGGACCAGCTGGTAGCGATTCTGGCCGAACGCTCGCTCGATATGGTC 5577
pmxA_Polymyxin-A              ACGGGAGTAGAAGCGGACCAGCTGGTAGCGATTCTGGCCGAACGCTCGCTCGATATGGTC 5487
pmxA_1G_Polymyxin             acgggagtagaagcggaccagctggtagcgattctggccgaacgctcgctcgatatggtc 5544
pmxA-D-DaB3-Polymyxin-B       CACGGGATATCCAAAGGCCGTCTGGTCGCCATTATGGCTGAGCGTTCCATCGAAATGGTG 5484
pmxA_B-LR_Polymyxin-E         CACGGGATATCCAAAGGTCGTCTGGTCGCCATTTTGGCTGAGCGTTCCATTGAAATGGTG 5577
pmxA_KP26_Polymyxin-E         CACGGGATATCCAAAGGTCGTCTGGTCGCCATTTTGGCTGAGCGTTCCATTGAAATGGTG 5577
                                 **  **      *  *  ***** ** *** **** ** ** **  * ** ***** 
```

```
pmxA_Polymyxin-P              GTCGGCATTCTGGCGATTCTCAAAGCGGGCGGAGCATACGTGCCTGTCGATCCCGACTAC 5637
pmxA_Polymyxin-A              GTCGGCATTCTGGCCATTCTCAAAGCGGGCGGAGCCTACGTACCTGTCGATCCCGACTAC 5547
pmxA_1G_Polymyxin             gtcggcattctggcgattctcaaagcgggcggagcctacgtgcctgtcgatcccgactac 5604
pmxA-D-DaB3-Polymyxin-B       GTGGGCATGCTGGCGGCATACAAAGCCGGAGCGGCTTACGTACCGATTGACCCGGAATAT 5544
pmxA_B-LR_Polymyxin-E         GTGGGCATGCTGGCGGCACACAAAGCCGGAGCGGCTTACGTACCGATTGACCCAGAATAT 5637
pmxA_KP26_Polymyxin-E         GTGGGCATGCTGGCGGCACACAAAGCCGGAGCGGCTTACGTACCGATTGACCCAGAATAT 5637
                              ** ***** *****      ****** ** *  ** ***** **  * ** ** ** **

pmxA_Polymyxin-P              CCGGAGGAGCGCATCCGCTTCATGATCGAGGATTCGGGCGCGCCGTTATTGCTGATTCAA 5697
pmxA_Polymyxin-A              CCGGAGGAGCGCATCCGCTTCATGATCGAGGATTCGGGCGCGCCGTTATTGCTGATTCAA 5607
pmxA_1G_Polymyxin             ccggaggagcgcatccgcttcatgatcgaggattcgggcgcgccgttattgctgattcaa 5664
pmxA-D-DaB3-Polymyxin-B       CCCGAGGAGCGTATCCGTTTCTTGATCGAGGATTCGGGAGCGCAGGTCATGCTGACGCAA 5604
pmxA_B-LR_Polymyxin-E         CCCGAGGAGCGTATCCGTTTCTTGATCGAGGATTCGGGAGGGCAGGTCATGCTGACGCAA 5697
pmxA_KP26_Polymyxin-E         CCCGAGGAGCGTATCCGTTTCTTGATCGAGGATTCGGGAGGGCAGGTCATGCTGACGCAA 5697
                              ** ******** ***** *** **************** * ** * *  ******  ***

pmxA_Polymyxin-P              AAGCATCTGCACGAGAAGACCGACTTCGCAGGAACGCGCCTCGAATTGGACGATTACGTT 5757
pmxA_Polymyxin-A              AAGCATCTGCACGAGAAGACCGACTTCGCAGGAACGCGCCTCGAATTGGACGATTTCGTT 5667
pmxA_1G_Polymyxin             aagcatctgcacgagaagaccgacttcgcaggaacgcgcctcgaattggacgatttcgtt 5724
pmxA-D-DaB3-Polymyxin-B       AGCCGCTTGCGCGAGCGCCTGGCGGGTTCGGACTCCGTGATCTTACTGGATGAC------ 5658
pmxA_B-LR_Polymyxin-E         AGCCGCTTGCGCGAGCGCCTGGCGGGTTCGGACCCCGTGATCTTACTGGATGAC------ 5751
pmxA_KP26_Polymyxin-E         AGCCGCTTGCGCGAGCGCCTGGCGGGTTCGGACCCCGTGATCTTACTGGATGAC------ 5751
                              *  *   *** ****      *      * *   *     **  * **** **

pmxA_Polymyxin-P              TGGGGTGACAGAGGGGCGGACTCCGCCGATGCGCTGGACGCTTCGAACCTGGAGCCGATT 5817
pmxA_Polymyxin-A              TGGGGCGACAGAGGGGCGGACTCCGCCGATGCGTTGGACGCTTCGAACCTGGAGCCGATT 5727
pmxA_1G_Polymyxin             tggggcgacagaggggcggactccgaaggtgcgctggacgcttcgaacctggagccgatt 5784
pmxA-D-DaB3-Polymyxin-B       ------------------GAGTCCTTCTATCACGAGGACGGCACAAATCTAAATCCGGGC 5700
pmxA_B-LR_Polymyxin-E         ------------------GAGTCCTTCTATCACGAGGACGGCACAAATCTAAATACGGGC 5793
pmxA_KP26_Polymyxin-E         ------------------GAGTCCTTCTATCACGAGGACGGCACAAATCTAAATACGGGC 5793
                                                ** ***     *     *****   * ** **  *  **

pmxA_Polymyxin-P              TCCGGGCCGGGCAACCTGGCCTATGTCATCTACACGTCGGGAACGACCGGCAGACCGAAA 5877
pmxA_Polymyxin-A              TCCGGGCCGGGCAACCTGGCATATGTCATCTACACGTCGGGAACGACCGGCAGACCGAAA 5787
pmxA_1G_Polymyxin             tccgggccgggcaacctggcatatgtcatctacacgtcgggaacgaccggcagaccgaaa 5844
pmxA-D-DaB3-Polymyxin-B       ACCGAAGCGACGGATCTGGCCTGCGTCATCTATACGTCAGGCACGACGGGCAAGCCGAAA 5760
pmxA_B-LR_Polymyxin-E         ATCGAAGCGACAGATCTGGCCTGCGTCATCTATACGTCAGGCACGACGGGCAAGCCGAAA 5853
pmxA_KP26_Polymyxin-E         ATCGAAGCGACAGATCTGGCCTGCGTCATCTATACGTCAGGCACGACGGGCAAGCCGAAA 5853
                                **   **    * ***** *  ******** ***** ** ***** ****  ******

pmxA_Polymyxin-P              GGAACGCTGATCGAGCATAAGAACGTCGTGCGCCTCCTGTTTAACGACAAGAACCTGTTC 5937
pmxA_Polymyxin-A              GGAACGCTGATCGAGCATAAGAACGTCGTGCGGCTCCTGTTCAACGACAAGAACCTGTTC 5847
pmxA_1G_Polymyxin             ggaacgctgatcgagcataagaacgtcgtgcggctcctgttcaacgacaagaacctgttc 5904
```

```
pmxA-D-DaB3-Polymyxin-B      GGCAATCTTGTTTCGCACCGCAACATCGTGCGGATCGTGCGAAATACGAATTATATCGAC 5820
pmxA_B-LR_Polymyxin-E        GGCAACCCTGTTTCGCACCGCAACATCGTGCGGGTCGTGCAAAATACGAATTATATCGAC 5913
pmxA_KP26_Polymyxin-E        GGCAACCCTGTTTCGCACCGCAACATCGTGCGGGTCGTGCAAAATACGAATTATATCGAC 5913
                             ** *  *   *   ***    *** *******  ** **   **    **  *  *   *

pmxA_Polymyxin-P             GACTTCGGGCCGTCCGACACGTGGACGCTGTTCCACTCGTTCTGCTTCGATTTCTCCGTC 5997
pmxA_Polymyxin-A             GACTTCGGGCCGTCCGACACGTGGACGCTGTTCCACTCGTTCTGCTTCGATTTCTCCGTC 5907
pmxA_1G_Polymyxin            gacttcgggccgtccgacacgtggacgctgttccactcgttctgcttcgatttctccgtc 5964
pmxA-D-DaB3-Polymyxin-B      ATCACCGAACGGGATCATGTCCTCCAGCTTTCGAGCTA---TTCGTTCGACGGAGCGATT 5877
pmxA_B-LR_Polymyxin-E        ATCACCGAGCGGGATCATGTCCTCCAGCTTTCGAGCTA---TTCGTTCGACGGAGCGACT 5970
pmxA_KP26_Polymyxin-E        ATCACCGAGCGGGATCATGTCCTCCAGCTTTCGAGCTA---TTCGTTCGACGGAGCGACT 5970
                               *  **  * *    *         *** *    **     *  *****     *

pmxA_Polymyxin-P             TGGGAAATGTACGGAGCGTTGCTGTACGGAGGCAAGCTGGTCATCGTACCGCCGCTCACG 6057
pmxA_Polymyxin-A             TGGGAAATGTACGGAGCGCTGCTGTACGGAGGCAAGCTGGTCATCGTACCGTCGCTCACG 5967
pmxA_1G_Polymyxin            tgggaaatgtacggagcgctgctgaacggaggcaagctggtcatcgtaccgccgctcacg 6024
pmxA-D-DaB3-Polymyxin-B      TTCGATATTTTCGGCGCTTTGACCAACGGGGCGCGGCTGGTGCTGGTTCCCCGCAAGACT 5937
pmxA_B-LR_Polymyxin-E        TTCGATATTTTCGGCGCTTTGACCAACGGGGCGCGGCTGGTGCTGGTTCCCTACGAGACT 6030
pmxA_KP26_Polymyxin-E        TTCGATATTTTCGGCGCTTTGACCAACGGGGCGCGGCTGGTGCTGGTTCCCTACGAGACT 6030
                             *  ** ** * *** **  **    **** *    ******  * ** **       **

pmxA_Polymyxin-P             GCGAAAAATCCGGCCGATTTCCTGGCGCTGCTGGGCCGCGAACAGGTCACGATTTTGAAC 6117
pmxA_Polymyxin-A             GCGAAAAATCCGGCCGATTTCCTGGCGTTGCTGGGCCGCGAACAGGTCACGATTTTGAAC 6027
pmxA_1G_Polymyxin            gcgaaaaatccggtcgatttcctggcgctgctgggccgcgaacaggtcacgattttgaac 6084
pmxA-D-DaB3-Polymyxin-B      TTGCTGGAAATCGTCCGGCTGGCGGATCTCATCCAGCGCGAGCGCATCTCGGTCATGCTG 5997
pmxA_B-LR_Polymyxin-E        TTGCTGGAAATCGGCCGGCTGGCGGATCTCATCCAGCGCGAGCGCATCTCGGTCATGTTT 6090
pmxA_KP26_Polymyxin-E        TTGCTGGAAATCGGCCGGCTGGCGGATCTCATCCAGCGCGAGCGCATCTCGGTCATGTTT 6090
                               *    *    * *    *   **   *  *    ***** *   ** ** *  **

pmxA_Polymyxin-P             CAGACACCAACGTACTTCTACCAGCTGCTGCGTGAGGTCTTGGCGGACCATCCGTACGAT 6177
pmxA_Polymyxin-A             CAGACGCCAACATACTTCTACCAGCTGCTGCGTGAGGTCTTGGCGGACCATCCGTACGAT 6087
pmxA_1G_Polymyxin            cagacgccaacgtacttctaccagctgctgcgtaaggtcttggcggaccatccgtacgat 6144
pmxA-D-DaB3-Polymyxin-B      ATTACGACGGCTTTCTTCAACGTCCTTGTAGATGTGAACGTCGATTGCCTGCGGGATGTC 6057
pmxA_B-LR_Polymyxin-E        ATTACGACGGCTTTCTTCAACATCCTTGTAGATGTGAACGTCGACTGCCTGCGGGATGTC 6150
pmxA_KP26_Polymyxin-E        ATTACGACGGCTTTCTTCAACATCCTTGTAGATGTGAACGTCGACTGCCTGCGGGATGTC 6150
                                **  *  * * **** **   **  *   *  *  * * *    **  * * * *

pmxA_Polymyxin-P             CTGCGGATTCGCAACGTCATCTTCGGGGGCGAAGCGCTGAGTCCGCTGCTGCTCAAGGGC 6237
pmxA_Polymyxin-A             CTGCGGATTCGCAACGTCATCTTTGGGGGCGAAGCGCTGAGTCCGCTGCTGCTCAAGGGC 6147
pmxA_1G_Polymyxin            ctgcggattcgcaacgtcatcttcgggggcgaagcgctgagtccgctgctgctcaagggc 6204
pmxA-D-DaB3-Polymyxin-B      CGGGCGATTTTGTTCGGAG-----GAGAGCGAGTGTCGGTCGGACATGTGCGTAAAGCGC 6112
pmxA_B-LR_Polymyxin-E        AGGGCGATTTTGTTCGGAG-----GAGAGCGTGTGTCGGTCGGCCATGTGCGCAAAGCGC 6205
pmxA_KP26_Polymyxin-E        AGGGCGATTTTGTTCGGAG-----GAGAGCGTGTGTCGGTCGGCCATGTGCGCAAAGCGC 6205
                               *  ****     **        * * ***     * *       **      *** **
```

```
pmxA_Polymyxin-P              TTCAAGACGAAGTACCCGGAGACGAAGCTGATCAATATGTATGGCATTACCGAGACGACG 6297
pmxA_Polymyxin-A              TTCAAGACGAAGTACCCGGAGACGAAGCTGATCAATATGTACGGCATTACCGAGACGACG 6207
pmxA_1G_Polymyxin             ttcaagacgaagtacccggagacgaagctgatcaatatgtacggcattaccgagacgacg 6264
pmxA-D-DaB3-Polymyxin-B       TCGCCCATATCGGACCGGGC----AGGCTCAACCACCTGTACGGCCCGTCGGAAAGCACG 6168
pmxA_B-LR_Polymyxin-E         TCGCCCATATCGGACCGGGC----AGGCTCAACCATGTGTACGGCCCGACGGAAAGCACG 6261
pmxA_KP26_Polymyxin-E         TCGCCCATATCGGACCGGGC----AGGCTCAACCATGTGTACGGCCCGACGGAAAGCACG 6261
                              *     *    * *** **     * *** * * *  **** ***    * ** *  ***

pmxA_Polymyxin-P              GTTCACGTGACGTATAAGGAAATCACGTGGGTCGAAATGGAGGCGGCGAAGAGCAATATC 6357
pmxA_Polymyxin-A              GTTCACGTGACGTATAAGGAAATCACGTGGGTCGAAATGGAGGCGGCGAAGAGCAATATC 6267
pmxA_1G_Polymyxin             gttcacgtgacgtataaggaaataacgtgggtcgaaatggaggcggcgaagagcaatatc 6324
pmxA-D-DaB3-Polymyxin-B       GTTTATACCACGTACCTTCCGGTCGACTTCGTCGATGAGTCGGCGGTTACCGTACCCATC 6228
pmxA_B-LR_Polymyxin-E         GTTTATACCACGTACCTTCCGGTCGACTTCGTCGATGAGTTGGCGGTTACCGTACCCATT 6321
pmxA_KP26_Polymyxin-E         GTTTATACCACGTACCTTCCGGTCGACTTCGTCGATGAGTTGGCGGTTACCGTACCCATT 6321
                              *** *    *****        *    *  *****   *  *****  *        **

pmxA_Polymyxin-P              GGCAAGCCGATCCCGACGCTGAGGGTGTACGTCCTTGATGAAAACCGCCGCCCTGTGCCG 6417
pmxA_Polymyxin-A              GGCAAGCCGATCCCGACGTTGAGGGTGTACGTCCTAGATGAAAACCGCCGCCCTGTGCCG 6327
pmxA_1G_Polymyxin             ggcaagccgatcccgacgctgagggtgtacatcctggatgaaaaccgccgccctgtgccg 6384
pmxA-D-DaB3-Polymyxin-B       GGACGGCCGATCAGCAATACGAAGGTGTATATCGTCGACAGCCGGAATAAGCTTCTGCCG 6288
pmxA_B-LR_Polymyxin-E         GGACGGCCGATCAGCAATACGACGGTGTATATCGTCGACAGCCGGAACAAACTTCTGCCG 6381
pmxA_KP26_Polymyxin-E         GGACGGCCGATCAGCAATACGACGGTGTATATCGTCGACAGCCGGAACAAACTTCTGCCG 6381
                              **   *******   *    ** ******  ** * **             * * *****

pmxA_Polymyxin-P              ATTGGAGTAGCGGGCGAAATGTACGTGGCCGGGGAAGGCCTTGCGAGAGGATACCTGAAC 6477
pmxA_Polymyxin-A              ATCGGCGTAGCGGGCGAAATGTACGTGGCCGGGGAAGGCCTTGCGAGAGGATACCTGAAC 6387
pmxA_1G_Polymyxin             atcggcgtagtgggcgaaatgtacgtggccggggaaggccttgcgagaggatacctgaac 6444
pmxA-D-DaB3-Polymyxin-B       ATCGGCGTGGCCGGGGAGCTTTGCGTCGGCGGAGAAGGCTTGGTACGGGGCTACAATAAC 6348
pmxA_B-LR_Polymyxin-E         ATCGGCGTGGCCGGGGAACTTTGCGTCGGCGGAGAAGGCTTGGTAAGGGGCTACAATAAC 6441
pmxA_KP26_Polymyxin-E         ATCGGCGTGGCCGGGGAACTTTGCGTCGGCGGAGAAGGCTTGGTAAGGGGCTACAATAAC 6441
                              ** ** ** *  ** **  * * *** * *** ****** * *   * ** ***   ***

pmxA_Polymyxin-P              CGTCCGGATCTGACGGCGGAGAAGTTCGTCGATTCCCCGTTTGCGGAGGGGGAGAAGCTG 6537
pmxA_Polymyxin-A              CGTCCGGATCTGACGGCGGAGAAGTTCGTTGATTCCCCGTTTGCGGAGGGGGAGAAACTG 6447
pmxA_1G_Polymyxin             cgtccggatctgacggcggagaagttcgtcgattccccgtttgcggagggggagaaactg 6504
pmxA-D-DaB3-Polymyxin-B       CGGCCGGAGCTGACGGCGGAGAAATTTGTGGACAATCCGTTTGTGCTGGGAGAGCGCATG 6408
pmxA_B-LR_Polymyxin-E         CGGCCGGAGCTGACGGCGGAGAAATTTGTGGACAATCCGTTTGTGCCGGGAGAGCGCATG 6501
pmxA_KP26_Polymyxin-E         CGGCCGGAGCTGACGGCGGAGAAATTTGTGGACAATCCGTTTGTGCCGGGAGAGCGCATG 6501
                              ** ***** ************** ** ** **    ******* *  *** ***    **

pmxA_Polymyxin-P              TACCGCTCGGGCGACTTGGCGGCTTGGCTGCCGGACGGCAACATCGAATACCTGGGCCGG 6597
pmxA_Polymyxin-A              TACCGTTCGGGCGACTTGGCGGCTTGGCTGCCGGACGGCAACATCGAATACCTGGGCCGG 6507
pmxA_1G_Polymyxin             taccgctcgggcgacttggcggcttggctgccggacggcaacatcgaatacctgggccgg 6564
```

```
pmxA-D-DaB3-Polymyxin-B      TACCGGACGGGGGATTTGGCGAAATGGCTGCCGGACGGAACGATCGAATACGTGGGACGG 6468
pmxA_B-LR_Polymyxin-E        TACCGGACGGGGGATTTGGCGAAATGGCTGCCGGACGGCACGATCGAATACGTGGGACGG 6561
pmxA_KP26_Polymyxin-E        TACCGGACGGGGGATTTGGCGAAATGGCTGCCGGACGGCACGATCGAATACGTGGGACGG 6561
                             *****  **** ** ******   ************** *  ********* **** ***

pmxA_Polymyxin-P             ATCGACCACCAGGTCAAAATCCGCGGGTACCGAATCGAGCTGGACGAAATCGAGACGCAG 6657
pmxA_Polymyxin-A             ATCGACCACCAGGTCAAAATCCGTGGGTACCGGATCGAGCTGGACGAAATCGAGACGCAG 6567
pmxA_1G_Polymyxin            atcgaccaccaggtcaaaatccgcgggtaccggatcgagctggacgaaatcgagacgcag 6624
pmxA-D-DaB3-Polymyxin-B      ACGGACGACCAAGTGAAAATCCGCGGCTTCCGCATTGAGCTGGGCGAGATCGAAGCTCAG 6528
pmxA_B-LR_Polymyxin-E        ACGGACGACCAAGTGAAAATCCGCGGCTTCCGAATTGAGCTAGGCGAGATCGAAGCTCAG 6621
pmxA_KP26_Polymyxin-E        ACGGACGACCAAGTGAAAATCCGCGGCTTCCGAATTGAGCTAGGCGAGATCGAAGCTCAG 6621
                             *  *** **** ** ******** ** * *** ** ***** * *** *****  * ***

pmxA_Polymyxin-P             CTTCTGAACGTCGAGGGCGTGGAAGAAGCGGTGGTGCTCGCCCGTCAGGACGGCGAGGGC 6717
pmxA_Polymyxin-A             CTTCTGAACGTCCGGGGTGTGGAAGAAGCGGTGGTGCTCGCCCGTCAGGACGGCGGGGGC 6627
pmxA_1G_Polymyxin            cttctgaacgtcgagggcgtggaagaagcggtggtgctcgcccgtcaggacggggggggc 6684
pmxA-D-DaB3-Polymyxin-B      CTTCAGAAAGTGGAGGGAATTCGGAAAGCAACGGTATTCGCGAGGGAAGACGTCTCCGGC 6588
pmxA_B-LR_Polymyxin-E        CTTCAGAAAGTGGAGGGAATTCGGAAAACGACGGTATTCGCGAGGGAAAACGCCTCCGGC 6681
pmxA_KP26_Polymyxin-E        CTTCAGAAAGTGGAGGGAATTCGGAAAACGACGGTATTCGCGAGGGAAAACGCCTCCGGC 6681
                             **** *** **   ***  *     ** *   ***  ****  *  *  ***     ***

pmxA_Polymyxin-P             GAGAAGGCGCTTGTTGCCTACTTTGTGGCGGACCGAACGCTGACGGTCAGCGAAATGAGA 6777
pmxA_Polymyxin-A             GAGAAGGCGCTTGTCGCCTACTTTGTGGCGGACCGGACGCTGACGGTCAGCGAAATGAGA 6687
pmxA_1G_Polymyxin            gagaaggcgcttgtcgcctactttgtggcggaccgaacgctgacggtcagcgaaatgaga 6744
pmxA-D-DaB3-Polymyxin-B      GAGAAGCAGCTTTGCGCCTATTATGAAGCGGACCGCGAGCTTCCGGCGGCCGAGCTGAAG 6648
pmxA_B-LR_Polymyxin-E        GAGAAGCAGCTTTGCGCCTATTATGAAGCGGACTGCGAGCTTCCGGCGGCCGAACTGAAG 6741
pmxA_KP26_Polymyxin-E        GAGAAGCAGCTTTGCGCCTATTATGAAGCGGACTGCGAGCTTCCGGCGGCCGAACTGAAG 6741
                             ******  ****   ***** * **  ****** *   ***  ***    ***  ***

pmxA_Polymyxin-P             ACCTCGCTGGCCCAGGGAATGCCGGGGTACATGATCCCTTCGTACTTCGTGCAGTTGGAG 6837
pmxA_Polymyxin-A             ACCTCGCTGGCCCAGGGAATGCCGGGGTACATGATCCCGTCGTACTTCGTGCAGTTGGAG 6747
pmxA_1G_Polymyxin            acctcgctggcccagggaatgccggggtacatgatcccgtcgtacttcgtgcagttggag 6804
pmxA-D-DaB3-Polymyxin-B      AGCGTGCTTTCCCAGGAACTGCCGGCCTATATGATTCCGGCGTACCTGATCCAGTTGGAG 6708
pmxA_B-LR_Polymyxin-E        AGCGTGCTTTCCAAGGAACTGCCGGCCTATATGATCCCGGCGTACCTGATCCAGTTGGAG 6801
pmxA_KP26_Polymyxin-E        AGCGTGCTTTCCAAGGAACTGCCGGCCTATATGATCCCGGCGTACCTGATCCAGTTGGAG 6801
                             * *  ***  ** *** * ******  ** ***** **  ***** *  * *********

pmxA_Polymyxin-P             CGCATGCCGCTGACGACCAACGGCAAAGTGGACCGCAAAGCGCTGCCGGAGCCGCAAGGC 6897
pmxA_Polymyxin-A             CGCATGCCGCTGACGACCAACGGCAAAGTGGACCGCAAAGCGCTGCCGGAGCCGCAAGGC 6807
pmxA_1G_Polymyxin            cgtatgccgctgacgaccaacggcaaagtggaccgcaaagcgctgccggagccgcaaggc 6864
pmxA-D-DaB3-Polymyxin-B      CGGCTCCCGCTGACGACGAACGGCAAGGTCGACCGCCGATCACTCCCGGCGCCGGAGGAG 6768
pmxA_B-LR_Polymyxin-E        CGGCTCCCGCTGACGACGAACGGCAAGGTCGACCGCCGATCACTCCCGGCGCCGGAGGAG 6861
pmxA_KP26_Polymyxin-E        CGGCTCCCGCTGACGACGAACGGCAAGGTCGACCGCCGATCACTCCCGGCGCCGGAGGAG 6861
                             **  * *********** ******** ** ******  * * ** **** **** * *
```

```
pmxA_Polymyxin-P             GGCATACAAACGGGCGTCGAATACGTAGCGCCGCGTAACCGGACGGAGTCCCAGCTTGTG 6957

pmxA_Polymyxin-A             GGCATACAAACGGGTGTCGAATACGTAGCGCCGCGTAACTGGACGGAGTCCCAGCTTGTG 6867

pmxA_1G_Polymyxin            ggcatacaaacgggcgtcgaatacgtagcgccgcgtaaccggacggagtcccagcttgtg 6924

pmxA-D-DaB3-Polymyxin-B      AGCTTGCAGCCGGGCGAAGAACGTACTCCGCCTCGGACTCCGCTGGAAGCCAGCTTGGCG 6828

pmxA_B-LR_Polymyxin-E        AGCTTGCAGCCGGGCGGAGGAAGTACTCCGCCTCGGACTCCGCTGGAAGCCAGCTTGGCC 6921

pmxA_KP26_Polymyxin-E        AGCTTGCAGCCGGGCGGAGGAAGTACTCCGCCTCGGACTCCGCTGGAAGCCAGCTTGGCC 6921

                              ** * **  **** *  * *       **** ** *    *  ***  **    * *


pmxA_Polymyxin-P             AAGATCTGGGAGGAAGTACTGGGCTACTCCGGCATTGGAGTCCTGGACAATTTCTTCGAC 7017

pmxA_Polymyxin-A             AAGATCTGGGAGGAAGTGCTGGGCTACTCCGGCATTGGAGTCCTGGACAATTTCTTCGAG 6927

pmxA_1G_Polymyxin            aagatctgggaggaagtgctgggctactctggcattggagtcctggacaatttcttcgag 6984

pmxA-D-DaB3-Polymyxin-B      GGAATTTGGAAAAGCGTGCTCGGACTGGAGCACATCGGGGTTCATGACAACTTCTTCGAC 6888

pmxA_B-LR_Polymyxin-E        GGAATTTGGAAAAGCGTGCTCGGACTAGTGCACATTGGCGTTCATGACAACTTCTTCGAC 6981

pmxA_KP26_Polymyxin-E        GGAATTTGGAAAAGCGTGCTCGGACTAGTGCACATTGGCGTTCATGACAACTTCTTCGAC 6981

                                ** *** *    ** ** **         *** ** ** *  ***** ********


pmxA_Polymyxin-P             CTGGGCGGCCATTCCCTGCGGGCGACGACACTGGTGAGCAAGGTGCATCAGGAGCTGAAC 7077

pmxA_Polymyxin-A             CTCGGAGGCCACTCCTTGCGGGCGACGAACCTTGTCAGCAAGATTCAGAAGGAAATGAAC 6987

pmxA_1G_Polymyxin            ctcggaggccactccttgcgggcgacgaaccttgtcagcaagattcagaaggaaatgaac 7044

pmxA-D-DaB3-Polymyxin-B      CTGGGCGGCCATTCCCTGCGGGCGACGACACTGGTGAGCAAGGTGCATCAGGAGCTGAAC 6948

pmxA_B-LR_Polymyxin-E        ATGGGCGGACATTCCCTGCGGGCGACGACACTGGTGAGCAAGGTGCATCAGGAGCTGAAC 7041

pmxA_KP26_Polymyxin-E        ATGGGCGGACATTCCCTGCGGGCGACGACACTGGTGAGCAAGGTGCATCAGGAGCTGAAC 7041

                              * ** ** ** *** ************  ** ** ****** * **  ****  *****


pmxA_Polymyxin-P             GTTGAGCTGCCTCTGCGCGACGTATTCCGCTACTCAACAATCGAAGAGATGGCTCTGGCC 7137

pmxA_Polymyxin-A             GTCGAGCTGCCTCTGCGCGACGTATTCCGCTATTCGACGATCGAAGAGATGGCTCTTGCC 7047

pmxA_1G_Polymyxin            gtcgagctgcctctgcgcgacgtattccgctactcgacgatcgaagagatggctcttgcc 7104

pmxA-D-DaB3-Polymyxin-B      GTCGAGCTGCCTCTGCGCGACGTATTCCGCTACTCGACGATCGAAGAGATGGCTCTCGCC 7008

pmxA_B-LR_Polymyxin-E        GTCGAACTGCCTCTGCGCGACGTATTCCGCTACTCGACGATCGAGGAGATGGCTCTCGCC 7101

pmxA_KP26_Polymyxin-E        GTCGAACTGCCTCTGCGCGACGTATTCCGCTACTCGACGATCGAGGAGATGGCTCTCGCC 7101

                             ** ** ************************** ** ** ***** *********** ***


pmxA_Polymyxin-P             ATCTCCCGGATCGGAGAGCAGTCGTTCTCGTCGATTCCGCTGGCAGGCGCAAGAACATAT 7197

pmxA_Polymyxin-A             ATCTCCCGGATCGGAGAGCAGTCGTTCTCGTCGATTCCGCTGGCAGGCGCAAGAGCATAT 7107

pmxA_1G_Polymyxin            atctcccggatcggagagcagtcgttctcgtcgattccgctggcaggcgcaagagcatat 7164

pmxA-D-DaB3-Polymyxin-B      ATCTCCCGGATCGGAGAGCAGTCGTTCTCGTCGATTCCGCTGGCGGGCGCAAGAGCATAT 7068

pmxA_B-LR_Polymyxin-E        ATCTCCCGGATCGGAGAGCAGTCGTTCTCGTCGATTCCGCTGGCAGGCGCAAGAGCATAT 7161

pmxA_KP26_Polymyxin-E        ATCTCCCGGATCGGAGAGCAGTCGTTCTCGTCGATTCCGCTGGCAGGCGCAAGAGCATAT 7161

                             ******************************************** ********* *****


pmxA_Polymyxin-P             TATCCGCTTTCCTCAGCTCAGAAGCGGCTGTTTATCCTGAATCAGCTGGAAGGGGCCGAT 7257

pmxA_Polymyxin-A             TATCCGCTTTCCTCAGCTCAGAAGCGGCTGTTTATCCTGAATCAGCTGGAAGGGGCCGAT 7167

pmxA_1G_Polymyxin            tatccgctttcctcagctcagaagcggctgtttatcctgaatcagctggaaggggccgat 7224
```

```
pmxA-D-DaB3-Polymyxin-B      TATCCGCTTTCCTCAGCTCAGAAGCGGCTGTTTATCCTGAATCAGCTGGAAGGGGCCGAT 7128
pmxA_B-LR_Polymyxin-E        TATCCGCTTTCCTCAGCTCAGAAGCGGCTGTTTATCCTGAATCAGCTGGAAGGGGCCGAT 7221
pmxA_KP26_Polymyxin-E        TATCCGCTTTCCTCAGCTCAGAAGCGGCTGTTTATCCTGAATCAGCTGGAAGGGGCCGAT 7221
                             ************************************************************

pmxA_Polymyxin-P             CAGAGCTACAACATGCCGGGCGTGCTGTTGCTGGAAGGATCGATTGACCGGAGCCTGCTG 7317
pmxA_Polymyxin-A             CAGAGCTACAACATGCCGGGGGTGCTGCTGCTGGAAGGATCGATTGACCGGAGCCTGCTG 7227
pmxA_1G_Polymyxin            cagagctacaacatgccgagcgtgctgctgctggaaggatcgattgaccggagcctgctg 7284
pmxA-D-DaB3-Polymyxin-B      CAGAGCTACAACATGCCGGGCGTGCTGCTGCTGGAAGGATCGATTGACCGGAGCCTGCTG 7188
pmxA_B-LR_Polymyxin-E        CAGAGCTACAACATGCCGGGCGTGCTGCTGTTGGAAGGATCGATTGACCGGAGCCTGCTG 7281
pmxA_KP26_Polymyxin-E        CAGAGCTACAACATGCCGGGCGTGCTGCTGTTGGAAGGATCGATTGACCGGAGCCTGCTG 7281
                             ***************** * ****** ** ******************************

pmxA_Polymyxin-P             GAGAAGGCTTTCCGCGGACTGATCGCACGGCACGAAACGCTACGAACTGGCTTTGAGATC 7377
pmxA_Polymyxin-A             GAGAAGGCTTTCCGCGGACTGATCGCACGGCACGAAACGCTGCGAACCGGCTTTGAGATT 7287
pmxA_1G_Polymyxin            gagaaggctttccgcggactgatcgcacggcacgaaacactgcgaaccggctttgagatc 7344
pmxA-D-DaB3-Polymyxin-B      GAGAAGGCTTTCCGCGGACTGATCGCACGGCACGAAACGCTGCGAACCGGCTTTGAGATC 7248
pmxA_B-LR_Polymyxin-E        GAGAAGGCTTTCCGCGGACTGATCGCACGGCACGAAACGCTGCGAACCGGCTTTGAGATC 7341
pmxA_KP26_Polymyxin-E        GAGAAGGCTTTCCGCGGACTGATCGCACGGCACGAAACGCTGCGAACCGGCTTTGAGATC 7341
                             ************************************** ** ***** ***********

pmxA_Polymyxin-P             GTACAAGGCGAAGCTGTACAGCGCATTTACGAGAGCGTCGACTTTGCCGTTGAGTACCGT 7437
pmxA_Polymyxin-A             GTACAAGGCGAAGCAATACAGCGCATTTACGAGAGCGTCGACTTTGCCGTCGAGTACCGT 7347
pmxA_1G_Polymyxin            gtacaaggcgaagcaatacagcgcatttacgagagcgtcgactttgccgtcgagtaccgt 7404
pmxA-D-DaB3-Polymyxin-B      GTACAAGGCGAAGCTGTACAGCGCATTTACGAGAGCGTCGACTTTGCCGTCGAGTACCGT 7308
pmxA_B-LR_Polymyxin-E        GTACAAGGCGAAGCAGTACAGCGCATTTACGAGAGCGTCGACTTTGCCGTCGAGTACCGT 7401
pmxA_KP26_Polymyxin-E        GTACAAGGCGAAGCAGTACAGCGCATTTACGAGAGCGTCGACTTTGCCGTCGAGTACCGT 7401
                             **************  ********************************** *********

pmxA_Polymyxin-P             CATGCGAGCGAGGAAGAAGCGCCCGAAGTTGTGCAGGCCTTCATCCGGCCTTTCGACTTG 7497
pmxA_Polymyxin-A             CATGCGAGCGAGGAAGAAGCGCCTGAAGTCGTGCAGGCCTTCATCCGGCCTTTCGACTTG 7407
pmxA_1G_Polymyxin            catgcgagcgaggaagaagcgcctgaagtcgtgcaggacttcatccggcctttcgacttg 7464
pmxA-D-DaB3-Polymyxin-B      CATGTGAGCGAGGAAGAAGCGCCCGAAGTCGTGCAGGCCTTCATCCGGCCTTTCGACTTG 7368
pmxA_B-LR_Polymyxin-E        CATGCGAGCGAGGAAGAAACGCCTGAAGTCGTGCAGGCCTTCATCCGGCCTTTCGACTTG 7461
pmxA_KP26_Polymyxin-E        CATGCGAGCGAGGAAGAAACGCCTGAAGTCGTGCAGGCCTTCATCCGGCCTTTCGACTTG 7461
                             **** ************* **** ***** ******* **********************

pmxA_Polymyxin-P             GCGAAGCCTCCGCTGCTGCGAGCGGAACTCGTAGAGCTGGCAGCCGAACGTTATTTGCTG 7557
pmxA_Polymyxin-A             GCGAAGCCCCCGTTGCTGCGGGCGGAGCTCGTAGAGCTGGCAGCCGAACGTTACTTGCTG 7467
pmxA_1G_Polymyxin            gcgaagcctccgctgctgcgggcggagctcgtagagctggcagccgaacgttatttgctg 7524
pmxA-D-DaB3-Polymyxin-B      GCGAAGCCTCCGCTGCTGCGGGCGGAGCTCGTAGAGCTGGCAGCCGAACGTTATTTGCTG 7428
pmxA_B-LR_Polymyxin-E        GCAAAGCCTCCGCTGCTGCGGGCGGAGCTCGTAGAGCTGGCAATCGAACGTTATTTGCTG 7521
pmxA_KP26_Polymyxin-E        GCAAAGCCTCCGCTGCTGCGGGCGGAGCTCGTAGAGCTGGCAATCGAACGTTATTTGCTG 7521
                             ** ***** *** ******* ***** ***************  ********* ******
```

```
pmxA_Polymyxin-P            ATGTTCGACATGCACCATATCGTCTCCGACGGGGTTTCGATGGACGTGTTAGTCGAGGAA 7617
pmxA_Polymyxin-A            ATGTTCGACATGCACCATATCGTCTCCGACGGGGTTTCGATGGACGTGTTAGTCGAGGAA 7527
pmxA_1G_Polymyxin           atgttcgacatgcaccatatcgtctccgacggggtttcgatggacgtgttagtcgaggaa 7584
pmxA-D-DaB3-Polymyxin-B     ATGTTCGACATGCACCATATCGTCTCCGACGGGGTTTCGATGGACGTGTTAGTCGAGGAA 7488
pmxA_B-LR_Polymyxin-E       ATGTTCGACATGCACCATATCGTCTCCGACGGGGTTTCGATGGACGTGTTAGTCGAGGAA 7581
pmxA_KP26_Polymyxin-E       ATGTTCGACATGCACCATATCGTCTCCGACGGGGTTTCGATGGACGTGTTAGTCGAGGAA 7581
                            ************************************************************

pmxA_Polymyxin-P            CTCGTTCGTCTGTACGGCGGCGAATCATTAGAGCCTTTGCGCATTCAATACAAGGACTAT 7677
pmxA_Polymyxin-A            CTCGTTCGTCTGTACGGCGGCGAGTCATTAGAGCCTTTGCGCATTCAATACAAGGACTAT 7587
pmxA_1G_Polymyxin           ctcgttcgtctgtacggcggcgagtcattagagcctttgcacattcaatacaaggactat 7644
pmxA-D-DaB3-Polymyxin-B     CTCGTTCGTATGTACGGCGGCGAGTCATTAGAGCCTTTGCGCATTCAATACAAGGACTAT 7548
pmxA_B-LR_Polymyxin-E       CTCGTTCGTCTGTACGGCGGCGAGTCATTAGAGCCTTTGCGCATTCAATACAAGGACTAT 7641
pmxA_KP26_Polymyxin-E       CTCGTTCGTCTGTACGGCGGCGAGTCATTAGAGCCTTTGCGCATTCAATACAAGGACTAT 7641
                            ********* ************* **************** *******************

pmxA_Polymyxin-P            GCGGTATGGCAGCAGTCGGACGAGCAAAAAGTGCAGTTGAAACGCGAGGAAGCTTACTGG 7737
pmxA_Polymyxin-A            GCGGTATGGCAGCAGTCGGACGAGCAAAAAGTGCAGTTGAAACGCGAGGAAGCTTACTGG 7647
pmxA_1G_Polymyxin           gcggtatggcagcagtcggacgagcaaaaagtgcagttgaaacgcgaggaagcttactgg 7704
pmxA-D-DaB3-Polymyxin-B     GCGGTATGGCAGCAGTCGGACGAGCAAAAAGTGCAGTTGAAACGCGAGGAAGCTTACTGG 7608
pmxA_B-LR_Polymyxin-E       GCGGTATGGCAGCAGTCGGACGAGCAAAAAGTGCAGTTGAAACGCGAGGAAGCTTACTGG 7701
pmxA_KP26_Polymyxin-E       GCGGTATGGCAGCAGTCGGACGAGCAAAAAGTGCAGTTGAAACGCGAGGAAGCTTACTGG 7701
                            ************************************************************

pmxA_Polymyxin-P            CTGGATCGTTACCGCGGCGAGCTTCCGGTTCTGGAAATGCCGACGGACTATCCGCGTCCT 7797
pmxA_Polymyxin-A            CTGGACCGTTACCGGGGCGAGCTGCCGGTTCTGGAAATGCCGACGGACTATCCGCGTCCT 7707
pmxA_1G_Polymyxin           ctggaccgttaccggggcgagctgccggttctggaaatgccgacggactatccgcgtcct 7764
pmxA-D-DaB3-Polymyxin-B     CTGGACCGTTACCGGGGCGAGCTGCCGGTTCTGGAAATGCCGACGGACTATCCGCGTCCT 7668
pmxA_B-LR_Polymyxin-E       TTGGACCGTTACCGGGGCGAGCTGCCGGTTCTGGAAATGCCGACGGACTATCCGCGTCCT 7761
pmxA_KP26_Polymyxin-E       TTGGACCGTTACCGGGGCGAGCTGCCGGTTCTGGAAATGCCGACGGACTATCCGCGTCCT 7761
                             **** ******** ******** ************************************

pmxA_Polymyxin-P            GCCGTGCAGAGCTTTGAGGGACAAACGCTGACGTCCTTCGTGGACGAGGCAACGAACGAA 7857
pmxA_Polymyxin-A            GCCGTGCAGAGCTTTGAGGGACAAACGCTGACGTCCTTCGTGGACGAGGCAACGAACGAA 7767
pmxA_1G_Polymyxin           gccgtgcagagctttgagggacaaacgctgacgtccttcgtggacgaggcaacgaacgaa 7824
pmxA-D-DaB3-Polymyxin-B     GCCGTGCAGAGCTTTGAGGGACAAACGCTGACGTCCTTCGTGGACGAGGCAACGAACGAA 7728
pmxA_B-LR_Polymyxin-E       GCCGTGCAGAGCTTTGAGGGACAAACGCTGACGTCCTTCGTGGACGAGGCAACGAACGAA 7821
pmxA_KP26_Polymyxin-E       GCCGTGCAGAGCTTTGAGGGACAAACGCTGACGTCCTTCGTGGACGAGGCAACGAACGAA 7821
                            ************************************************************

pmxA_Polymyxin-P            GGCTTGAAGCAGCTGGCCGCTCAAAGAGGAACGACGCTGTATATGGTGCTGCTTGCGGCA 7917
pmxA_Polymyxin-A            GGCTTGAAGCAGCTGGCCGCTCAAAGAGGAACGACGCTGTATATGGTGCTGCTTGCGGCA 7827
pmxA_1G_Polymyxin           ggcttgaagcagctggccgctcaaagaggaacgacgctgtatatggtgctgcttgcggca 7884
```

```
pmxA-D-DaB3-Polymyxin-B      GGCTTGAAGCAGCTGGCCGCTCAAAGAGGAACGACGCTGTATATGGTGCTGCTTGCGGCA 7788
pmxA_B-LR_Polymyxin-E        GGCTTAAAGCAACTGGCCGCTCAAAAAGGAACGACGCTGTATATGGTACTGCTTGCGGCA 7881
pmxA_KP26_Polymyxin-E        GGCTTAAAGCAACTGGCCGCTCAAAAAGGAACGACGCTGTATATGGTACTGCTTGCGGCA 7881
                             ***** ***** ************* ********************* ************

pmxA_Polymyxin-P             TATACCGTGCTTTTGCATAAATACACAGGTCAGGACGATTTGATCGTCGGAACGTCGATT 7977
pmxA_Polymyxin-A             TATACCGTGCTTTTGCATAAATACACAGGTCAGGACGATTTGATCGTCGGAACGTCGATT 7887
pmxA_1G_Polymyxin            tataccgtgcttttgcataaatacacaggtcaggacgatttgatcgtcggaacgtcgatt 7944
pmxA-D-DaB3-Polymyxin-B      TATACCGTGCTTTTGCATAAATACACAGGTCAGGACGATTTGATCGTCGGAACGTCGATT 7848
pmxA_B-LR_Polymyxin-E        TATACCGTGCTTTTGCATAAATACACAGGTCAGGACGATTTGATCGTCGGAACGTCGATT 7941
pmxA_KP26_Polymyxin-E        TATACCGTGCTTTTGCATAAATACACAGGTCAGGACGATTTGATCGTCGGAACGTCGATT 7941
                             ************************************************************

pmxA_Polymyxin-P             GCGGGCAGAACGCACGGAGACACGCAGCCTTTGATCGGAATGTTCGTCAATACGCTGGCG 8037
pmxA_Polymyxin-A             GCGGGCAGAACGCACGGAGACACGCAGCCTTTGATCGGAATGTTCGTCAATACGCTGGCG 7947
pmxA_1G_Polymyxin            gcgggcagaacgcacggagacacgcagcctttgatcggaatgttcgtcaatacgctggcg 8004
pmxA-D-DaB3-Polymyxin-B      GCGGGCAGAACGCACGGAGACACGCAGCCTTTGATCGGAATGTTCGTCAATACGCTGGCG 7908
pmxA_B-LR_Polymyxin-E        GCGGGCAGAACGCACGGAGACACGCAGCCTTTGATCGGAATGTTCGTCAATACGCTGGCA 8001
pmxA_KP26_Polymyxin-E        GCGGGCAGAACGCACGGAGACACGCAGCCTTTGATCGGAATGTTCGTCAATACGCTGGCA 8001
                             ***********************************************************

pmxA_Polymyxin-P             CTCCGCAATTATCCGGCTTCGGAGAAGACCTTCCTGTCGTATCTGGAAGAAGTGAAAGAA 8097
pmxA_Polymyxin-A             CTCCGCAATTATCCGGCTTCGAAGAAGACCTTCCTGTCGTATCTGGAAGAAGTGAAAGAA 8007
pmxA_1G_Polymyxin            ctccgcaattatccggcttcggagaagaccttcctgtcgtatctggaagaagtgaaagaa 8064
pmxA-D-DaB3-Polymyxin-B      CTCCGCAATTATCCGGCTTCGGAGAAGACCTTTCTGTCGTATCTGGAAGAAGTGAAAGAA 7968
pmxA_B-LR_Polymyxin-E        CTCCGCAATTATCCGGCTTCGGAGAAGAGCTTCCTGTCGTATCTTGAAGAAGTGAAAGAA 8061
pmxA_KP26_Polymyxin-E        CTCCGCAATTATCCGGCTTCGGAGAAGAGCTTCCTGTCGTATCTTGAAGAAGTGAAAGAA 8061
                             ********************* ****** *** *********** ***************

pmxA_Polymyxin-P             ACGACCTTAGGCGCTTACGAGCATCAGAATTATCCGTTCGAAGAGCTCGTCGATAAAGTG 8157
pmxA_Polymyxin-A             ACGACTTTAGGCGCTTACGAGCATCAGAATTATCCGTTCGAAGAGCTCGTTGATAAAGTG 8067
pmxA_1G_Polymyxin            acgaccttaggcgcttacgagcatcagaattatccgttcgaagagctcgttgataaagtg 8124
pmxA-D-DaB3-Polymyxin-B      ACGACCTTAGGCGCTTACGAGCATCAGAATTATCCGTTCGAAGAGCTCGTCGATAAAGTG 8028
pmxA_B-LR_Polymyxin-E        ACGACCTTAGGCGCTTACGAGCATCAGAATTATCCGTTCGAAGAGCTCGTTGATAAAGTG 8121
pmxA_KP26_Polymyxin-E        ACGACCTTAGGCGCTTACGAGCATCAGAATTATCCGTTCGAAGAGCTCGTTGATAAAGTG 8121
                             ***** ******************************************** *********

pmxA_Polymyxin-P             CAGGTCAGCCGGGATTTGAGCCGCAACCCGCTGTTTGACACGATGTTCTCCCTGCAAAAC 8217
pmxA_Polymyxin-A             CAGGTCAGCCGGGATTTGAGCCGCAACCCGCTGTTTGACACGATGTTCTCCCTGCAAAAC 8127
pmxA_1G_Polymyxin            caggtcagccgggatttgagccgcaacccgctgtttgacacgatgttctccctgcaaaac 8184
pmxA-D-DaB3-Polymyxin-B      CAGGTCAGCCGGGATTTGAGCCGCAACCCGCTGTTTGACACGATGTTCTCCCTGCAAAAC 8088
pmxA_B-LR_Polymyxin-E        CAGGTCAGCCGGGATTTGAGCCGCAACCCGCTGTTTGACACGATGTTCTCCCTGCAAAAC 8181
pmxA_KP26_Polymyxin-E        CAGGTCAGCCGGGATTTGAGCCGCAACCCGCTGTTTGACACGATGTTCTCCCTGCAAAAC 8181
                             ************************************************************
```

```
pmxA_Polymyxin-P            TTGGAGGATAAAGAGTTTGAGCTGGAAGGGCTGAAATTGTCCCCGTACCCTAGCGAATAC 8277
pmxA_Polymyxin-A            TTGGAGGATAAAGAGTTTGAGCTGGAAGGGCTGAAATTGTCCTCGTACCCTAGCGAATAC 8187
pmxA_1G_Polymyxin           ttggaggataaagagtttgagctggaagggctgaaattgtccccgtaccctagcgaatac 8244
pmxA-D-DaB3-Polymyxin-B     TTGGAGGATAAAGAGTTTGAGTTGGAAGGGCTGAAATTGTCCCCGTACCCTAGCGAATAC 8148
pmxA_B-LR_Polymyxin-E       TTGGAGGATAAAGAGTTTAAGCTGGAAGGGCTGAAATTGTCCCCGTACCCTAGTGAATAC 8241
pmxA_KP26_Polymyxin-E       TTGGAGGATAAAGAGTTTAAGCTGGAAGGGCTGAAATTGTCCCCGTACCCTAGTGAATAC 8241
                            ****************** ** ******************** ********** ******

pmxA_Polymyxin-P            GGCACGGCCAAGTTCGACCTGAGTGTGGATGTTACGGAAGAAAACGGCGGCCTGGAGTGC 8337
pmxA_Polymyxin-A            GGCACGGCCAAGTTCGACCTGAGTGTGGATGTTACGGAAGAAAACGGCGGCCTGGAGTGC 8247
pmxA_1G_Polymyxin           ggcacggccaagttcgacctgagtgtggatgttacggaagaaaacggcggcctggagtgc 8304
pmxA-D-DaB3-Polymyxin-B     GGCACGGCCAAGTTCGACCTGAGTGTGGATGTTACGGAAGAAAATGGCGGCCTGGAGTGC 8208
pmxA_B-LR_Polymyxin-E       GGCACGGCCAAGTTCGATCTGAGTGTGGATGTTACGGAAGAAAACGGCGGCTTGGAGTGC 8301
pmxA_KP26_Polymyxin-E       GGCACGGCCAAGTTCGATCTGAGTGTGGATGTTACGGAAGAAAACGGCGGCTTGGAGTGC 8301
                            ***************** ************************** ****** ********

pmxA_Polymyxin-P            AGCTTTGAATTCGCAACGGCTCTTTATAAAGAAAGCACGATCCGGCGGCTGTCGACTCAT 8397
pmxA_Polymyxin-A            AGCTTTGAATTCGCAACGGCTCTTTATAAAGAAAGCACGATCCGGCGGCTGTCGACTCAT 8307
pmxA_1G_Polymyxin           agctttgaattcgcaacggctctttataaagaaagcacgatccggcggctgtcgactcat 8364
pmxA-D-DaB3-Polymyxin-B     AGCTTTGAATTCGCAACGGCTCTTTATAAAGAAAGCACGATCCGGCGGCTGTCGACTCAT 8268
pmxA_B-LR_Polymyxin-E       ATCTTTGAATTCGCAACGGCTCTTTATAAAGAAAGCACGATCCGGCGGCTGTCGACTCAT 8361
pmxA_KP26_Polymyxin-E       ATCTTTGAATTCGCAACGGCTCTTTATAAAGAAAGCACGATCCGGCGGCTGTCGACTCAT 8361
                            * **********************************************************

pmxA_Polymyxin-P            TTCGGACATTTGCTTGCGGCGATCGTAAGCCGTCCAGATGCGAAGATTGCCGAGCTGAAC 8457
pmxA_Polymyxin-A            TTCGGACATTTGCTTGCGGCGATCGCAAGCCGTCCGGATGCGAAGATTGCCGAGCTGAAC 8367
pmxA_1G_Polymyxin           ttcggacatttgcttgcggcgatcgtaagctgtccggatgcgaagattgccgagctgaac 8424
pmxA-D-DaB3-Polymyxin-B     TTCGGACATTTGCTCGTAACGATCGTAAGCCGTCCGGATGCGAAGATCGCCGAGCTGAAC 8328
pmxA_B-LR_Polymyxin-E       TTTGGACATTTGCTTGCGGCGATCGTAAGTCGTCCGGATGCGAAGATCGCCGAGCTGAAC 8421
pmxA_KP26_Polymyxin-E       TTTGGACATTTGCTTGCGGCGATCGTAAGTCGTCCGGATGCGAAGATCGCCGAGCTGAAC 8421
                            ** *********** *   ****** ***  **** *********** ************

pmxA_Polymyxin-P            TTGCTGACGGCAGAGGAAAAAGAACAAATTCTCGGCGCGTTCAACCCGGCACAGCCGGAA 8517
pmxA_Polymyxin-A            TTGCTGACGGCAGAGGAAAAAGAGCAAATTCTCGGCGCGTTCAACCCGGCGCAGCTGGAA 8427
pmxA_1G_Polymyxin           ttgttgacggcagaggaaaaagagcaaattctcggcgcgtttaacccggcgcagccggaa 8484
pmxA-D-DaB3-Polymyxin-B     TTGCTGACGGCAGAGGAAAAAGAACAAATTCTCGGCGCGTTTAACCCGGCGCAGCCTGAA 8388
pmxA_B-LR_Polymyxin-E       TTGTTGACGGCAGAGGAAAATGAGCAAATTCTCGGCGCGTTCAACCCGGCGCAGCCGGAA 8481
pmxA_KP26_Polymyxin-E       TTGTTGACGGCAGAGGAAAATGAGCAAATTCTCGGCGCGTTCAACCCGGCGCAGCCGGAA 8481
                            *** **************** ** ***************** ******** ****  ***

pmxA_Polymyxin-P            GCGGCTCCTGCGGCCGCGTTCCACCGGCTGTTTGAGGAACAGGCGGAGCGCACGCCGGAA 8577
pmxA_Polymyxin-A            GCGGCTCCTGCGGCCGCGTTCCACCGGCTGTTTGAGGAACAGGTGGAGCGCACGCCGGAA 8487
pmxA_1G_Polymyxin           gcggcttctgtggcagcgttccaccggctgttcgaggaacaggcggagcgcacgccggaa 8544
```

```
pmxA-D-DaB3-Polymyxin-B      GCGGCTCCTGTGGCGGCGTTCCACCGGCTGTTCGAGGAACAGGCGGAGCGCACGCCGGAA 8448
pmxA_B-LR_Polymyxin-E        GCGGCTCCTGCGGCCGCGTTCCACCGGCTGTTCGAAGAACAGGCGGAGCGCACGCCGGAA 8541
pmxA_KP26_Polymyxin-E        GCGGCTCCTGCGGCCGCGTTCCACCGGCTGTTCGAAGAACAGGCGGAGCGCACGCCGGAA 8541
                             ****** *** *** ***************** ** ******* ****************

pmxA_Polymyxin-P             GAGGCGGCCGTCGTGTACGAGAACGACCGGCTGACGTATGCGGAGCTGAACGAGCGGGCG 8637
pmxA_Polymyxin-A             GAGGCGGCCGTCGTGTACGAGAATGAGCGGCTGACGTATGCGGAGCTGAACGAGCGGGCG 8547
pmxA_1G_Polymyxin            gaggcggccgtcgtgtacgagaacgagcggctgacgtatgcggagctgaacgagcgggcg 8604
pmxA-D-DaB3-Polymyxin-B      GCGGAGGCCGTCGTGTACGAGAACGACCGGCTGACGTATGCGGAGCTGAACGAGCGGGCG 8508
pmxA_B-LR_Polymyxin-E        GCGGAGGCCGTCGTGTACGAGAACGACCGGCTGACGTATGCGGAGCTGAACGAGCGGGCG 8601
pmxA_KP26_Polymyxin-E        GCGGAGGCCGTCGTGTACGAGAACGACCGGCTGACGTATGCGGAGCTGAACGAGCGGGCG 8601
                             * ** ****************** ** *********************************

pmxA_Polymyxin-P             AACCGCTTGGCGGCTACGCTGCGCGCAAGCGGCATCGGCCGGGAGACGATCGTCGGCATT 8697
pmxA_Polymyxin-A             AACCGCTTGGCGGCTACGCTGCGCGCAAGCGGCATCGGCCGGGAGACGATCGTCGGCATT 8607
pmxA_1G_Polymyxin            aaccgcttggcgggcacgctgcgcgcaagcggcatcggccgggagacgatcgtcggcatt 8664
pmxA-D-DaB3-Polymyxin-B      AACCGCTTGGCGGCTACGCTGCGCGCAAGCGGCATCGGCCGGGAGACGATCGTCGGCATT 8568
pmxA_B-LR_Polymyxin-E        AACCGCTTGGCGGCTACGCTGCGCGCAAGCGGCATCGGCCGGGAGTCGATCGTCGGCATT 8661
pmxA_KP26_Polymyxin-E        AACCGCTTGGCGGCTACGCTGCGCGCAAGCGGCATCGGCCGGGAGTCGATCGTCGGCATT 8661
                             *************  ****************************** **************

pmxA_Polymyxin-P             CTCGCCGAGCGTTCGGTGGACTTGCTGGTGGCCGTGCTGGCCGTCTGGAAAGCGGGCGGG 8757
pmxA_Polymyxin-A             CTCGCCGAGCGTTCGGTGGACTTGCTGGTGGCCGTGCTGGCCGTCTGGAAAGCGGGCGGG 8667
pmxA_1G_Polymyxin            ctcgccgagcgttcggtggacttgctggtggccgtgctggccgtctggaaagcgggcggt 8724
pmxA-D-DaB3-Polymyxin-B      CTCGCCGAGCGTTCGGTGGACTTGCTGGTGGCCGTGCTGGCCGTCTGGAAAGCGGGCGGG 8628
pmxA_B-LR_Polymyxin-E        CTCTCCGAGCGTTCGGTGGACTTGCTGGTGGCCGTGCTGGCCGTCTGGAAAGCGGGCGGG 8721
pmxA_KP26_Polymyxin-E        CTCTCCGAGCGTTCGGTGGACTTGCTGGTGGCCGTGCTGGCCGTCTGGAAAGCGGGCGGG 8721
                             *** *******************************************************

pmxA_Polymyxin-P             GCGTATGTGCCACTCGACCCGGATTATCCAGCGGAGCGCGTGCGGTTCATGCTTGAAGAC 8817
pmxA_Polymyxin-A             GCGTATGTGCCGCTCGACCCGGATTATCCGGCAGACCGCGTGCGGTTCATGCTTGAAGAC 8727
pmxA_1G_Polymyxin            gcgtatgtgccgctcgacccggattatccggcagaccgcgtgcggttcatgcttgaagac 8784
pmxA-D-DaB3-Polymyxin-B      GCGTATGTGCCGCTCGACCCGGATTATCCGGCGGAGCGCGTGCGGTTCATGCTCGAAGAC 8688
pmxA_B-LR_Polymyxin-E        GCGTATGTGCCGCTCGACCCGGATTATCCGGCGGACCGCGTGCGGTTCATGCTTGAAGAC 8781
pmxA_KP26_Polymyxin-E        GCGTATGTGCCGCTCGACCCGGATTATCCGGCGGACCGCGTGCGGTTCATGCTTGAAGAC 8781
                             *********** ***************** ** ** ***************** ******

pmxA_Polymyxin-P             AGCGGAGCGAAGGTGCTGCTGACGCAAACGGCGCTGCGAGAGCGTGCCGAAGCCTGGCTC 8877
pmxA_Polymyxin-A             AGCGGAGCGAAGGTGCTGCTGACGCAAACGGCGCTGCGAGAGCGTGCCGAAGCCTGGCTC 8787
pmxA_1G_Polymyxin            agcggagcgaaggtgctgctgacgcaaacgctgctgcgagagcgtgccgaagcatggctc 8844
pmxA-D-DaB3-Polymyxin-B      AGCGGAGCGAAGGTACTGCTGACGCAAACGGCGCTGCGAGAGCGTGCTGAAGCCTGGCTC 8748
pmxA_B-LR_Polymyxin-E        AGTGGAGCGAAGGTGCTGCTGACGCAAACGGTGCTGCGAGAGCGCGCCGAAGCCTGGCTC 8841
pmxA_KP26_Polymyxin-E        AGTGGAGCGAAGGTGCTGCTGACGCAAACGGTGCTGCGAGAGCGCGCCGAAGCCTGGCTC 8841
                             ** *********** ***************  ************ ** ***** ******
```

```
pmxA_Polymyxin-P             GGCGAAGAGGAGTCGGCGCTGGCGGCGGTGCTGTACCTGGACGACGAAGCGTCGTACAGC 8937
pmxA_Polymyxin-A             GGCGAAGAGGAGCTGGCGCTGGCGGCGGTGCTGTACCTGGACGACGAAGCGTCGTACAAC 8847
pmxA_1G_Polymyxin            ggcgaagaggagctggcgctggcggcggtgctgtacctcgacgacgaagcgtcgtacagc 8904
pmxA-D-DaB3-Polymyxin-B      GACGAAGAGGAGCTGGCGCTGGCGGCGGTGCTGTACCTCGACGACGAAACTTCGTACAGC 8808
pmxA_B-LR_Polymyxin-E        GGCGAAGAGGAGCTGGCGCTGGCAGCGGTGCTGTACCTGGACGACGAAGCGTCGTACAGC 8901
pmxA_KP26_Polymyxin-E        GGCGAAGAGGAGCTGGCGCTGGCAGCGGTGCTGTACCTGGACGACGAAGCGTCGTACAGC 8901
                             * **********  ********* ************** ********* * ******* *

pmxA_Polymyxin-P             GAGGAGCGGGCGAATGCGCCGATTGGCTCGGGCATGGTCTCCGGCAAGCTGACGGATGCT 8997
pmxA_Polymyxin-A             GAGGAGCGGGCGAATGCGCCGGTTGGCTCCGGCATGGTCTCCGGCAAGCTGACGGATGCT 8907
pmxA_1G_Polymyxin            gacgagcgggcgaatgcgccgctggaagcgg----------------------------- 8935
pmxA-D-DaB3-Polymyxin-B      GAGGAGCGGACGAATGC------------------------------------------- 8825
pmxA_B-LR_Polymyxin-E        GAGGAGCGGGCGAATGCGCCGATTGGTTCCGGCATGGTCTCCGGCAAGCTGACGGATGCT 8961
pmxA_KP26_Polymyxin-E        GAGGAGCGGGCGAATGCGCCGATTGGTTCCGGCATGGTCTCCGGCAAGCTGACGGATGCT 8961
                             ** ****** *******

pmxA_Polymyxin-P             GTGGACGACAGCGATGAGAGCCATCAGCATGTCGGCACCGACAGCTTCCATGAAGCCCGT 9057
pmxA_Polymyxin-A             GTGGACGACGGCGATGAGAGCCATCAGAATGTCGACACCGACGGCTTCCATGAAGCCCGT 8967
pmxA_1G_Polymyxin            -------------------------------------atgatcacttgcacgaatgccgt 8958
pmxA-D-DaB3-Polymyxin-B      --------------------------------------CGACAGCTTCCATGAAGCCCGT 8847
pmxA_B-LR_Polymyxin-E        GTGGACGACGGCGATGTGAGCCATCAGAAGGTCGGCATGGGCAGCTTCCATGAAGCCCGT 9021
pmxA_KP26_Polymyxin-E        GTGGACGACGGCGATGTGAGCCATCAGAAGGTCGGCATGGGCAGCTTCCATGAAGCCCGT 9021
                                                                    *    *** ** ***  ****

pmxA_Polymyxin-P             CCGGAGGATCTGGCGTACGTGATCTATACGTCGGGAACGACGGGCAAGCCGAAGGGCGTG 9117
pmxA_Polymyxin-A             CCAGAGGACCTGGCGTACGTGATCTACACGTCGGGAACGACGGGCAAGCCGAAGGGCGTG 9027
pmxA_1G_Polymyxin            ccggaggacctggcgtacgtgatctacacgtcgggaacgacgggcaagccgaagggcgtg 9018
pmxA-D-DaB3-Polymyxin-B      CCGGAGGATCTGGCGTACGTGATCTATACGTCAGGAACGACGGGCAAGCCGAAGGGCGTG 8907
pmxA_B-LR_Polymyxin-E        CCGGAGGATCTGGCGTACGTGATCTATACGTCGGGAACGACGGGCAAGCCGAAGGGCGTA 9081
pmxA_KP26_Polymyxin-E        CCGGAGGATCTGGCGTACGTGATCTATACGTCGGGAACGACGGGCAAGCCGAAGGGCGTA 9081
                             ** ***** ***************** ***** **************************

pmxA_Polymyxin-P             ATGATCGAGCACCGCAGCCTGGTGAACACGGCGGCGGGCTACCGGCGGGAATACCGGTTA 9177
pmxA_Polymyxin-A             ATGATCGAGCACCGCAGCCTGGTGAACACGGCGGCGGGCTACCGGCGGGAATACCGGTTG 9087
pmxA_1G_Polymyxin            atgatcgagcaccgcagcctggtgaacacggcggcgggctaccggcgggaataccggttg 9078
pmxA-D-DaB3-Polymyxin-B      ATGATCGAGCACCGCAGCCTGGTGAACACGGCGGCGGGCTACCGGCGGGAATACCGGTTG 8967
pmxA_B-LR_Polymyxin-E        ATGATCGAGCACCGCAGCCTGGTGAACACGGCGGCGGGCTATCGGCGGGAATACCGGTTG 9141
pmxA_KP26_Polymyxin-E        ATGATCGAGCACCGCAGCCTGGTGAACACGGCGGCGGGCTATCGGCGGGAATACCGGTTG 9141
                             ***************************************** *****************

pmxA_Polymyxin-P             GATCAGTTCCCGGTGCGGCTGCTGCAGCTCGCAAGCTTCTCGTTCGACGTGTTCGTGGGA 9237
pmxA_Polymyxin-A             GATCAGTTCCCGGTGCGGCTGCTGCAGCTCGCAAGCTTCTCGTTCGACGTGTTCGTGGGA 9147
pmxA_1G_Polymyxin            gatcagttcccggtgcggctgctgcagctcgcaagcttctcgtttgacgtgttcgtggga 9138
```

```
pmxA-D-DaB3-Polymyxin-B      GATGAGTTCCCGGTGCGGCTGCTGCAGCTCGCCAGCTTCTCGTTCGACGTGTTCGTAGGA 9027
pmxA_B-LR_Polymyxin-E        GATCAGTTCCCGGTGCGGCTGCTGCAGCTCGCAAGCTTCTCGTTCGACGTATTCGTGGGC 9201
pmxA_KP26_Polymyxin-E        GATCAGTTCCCGGTGCGGCTGCTGCAGCTCGCAAGCTTCTCGTTCGACGTATTCGTGGGC 9201
                             *** **************************** *********** ***** ***** **

pmxA_Polymyxin-P             GATATCGCGCGGACGTTGTACAACGGAGGCACGATGGTGATCGTGCCGAAGGACGACCGG 9297
pmxA_Polymyxin-A             GATATCGCGCGGACGCTGTACAACGGAGGTACGATGGTGATTGTGCCGAAGGACGATCGG 9207
pmxA_1G_Polymyxin            gatatcgcccggacgctgtataacggaggcacgatggtgattgtgccgaaggacgaccgg 9198
pmxA-D-DaB3-Polymyxin-B      GATATCGCGCGGACGCTGTACAACGGAGGCACGATGGTGATTGTGCCGAAGGACGACCGG 9087
pmxA_B-LR_Polymyxin-E        GATATCGCGCGGACGCTGTATAACGGAGGCACGATGGTGATTGTGCCGAAGGACGACCGG 9261
pmxA_KP26_Polymyxin-E        GATATCGCGCGGACGCTGTATAACGGAGGCACGATGGTGATTGTGCCGAAGGACGACCGG 9261
                             ******** ****** **** ******** *********** ************** ***

pmxA_Polymyxin-P             ATTGATCCGTCTCGTCTGCACCACTGGATGGAGCGGGAACGGGTCACCATCTTCGAATCG 9357
pmxA_Polymyxin-A             ATTGATCCGTCTCGTCTGCACCACTGGATGGAGCGGGAGCGGGTCACCATCTTCGAATCG 9267
pmxA_1G_Polymyxin            atcgatccgtctcgtctgcactactggattgagcgggagcgggtcacaatcttcgaatcg 9258
pmxA-D-DaB3-Polymyxin-B      ATCGATCCGTCTCGTCTGCACCACTGGATGGAGCGGGAGCGGGTCACCATCTTCGAATCG 9147
pmxA_B-LR_Polymyxin-E        ATCGATCCGTCTCGTCTGCACCACTGGATGGAGCGGGAGCGGGTCACCATCTTCGAATCG 9321
pmxA_KP26_Polymyxin-E        ATCGATCCGTCTCGTCTGCACCACTGGATGGAGCGGGAGCGGGTCACCATCTTCGAATCG 9321
                             ** ****************** ******* ******** ******** ************

pmxA_Polymyxin-P             ACGCCGGCGCTGATCGTGCCGTTCCTGGAGTACGTGCACGAGCAGCGGCTGGATATGAGC 9417
pmxA_Polymyxin-A             ACGCCGGCGCTGATCGTGCCATTCCTGGAGTACGTGCACGAGCAGCGGCTGGATATGAGC 9327
pmxA_1G_Polymyxin            acgccggcgctgatcgtgccattcctggagtacgtgcacgagcagcaattggacatgagc 9318
pmxA-D-DaB3-Polymyxin-B      ACGCCGGCGCTGATCGTGCCGTTCCTGGAGTACGTGCACGAGCAGCGGCTGGATATGAGC 9207
pmxA_B-LR_Polymyxin-E        ACGCCGGCGCTGATCGTGCCGTTCCTGGAGTACGTGCACGAGCAGGGGCTGGATATGAGC 9381
pmxA_KP26_Polymyxin-E        ACGCCGGCGCTGATCGTGCCGTTCCTGGAGTACGTGCACGAGCAGGGGCTGGATATGAGC 9381
                             ******************** ************************    **** ******

pmxA_Polymyxin-P             TGGATGGAGCTGTTGATCACGAGCTCGGACAGCTGCAGCGTGGCGGATTACCGGACCTTG 9477
pmxA_Polymyxin-A             TGGATGGAGCTGTTGATCACGAGCTCGGACAGCTGCAGCGTGGCGGATTACCGGACCTTG 9387
pmxA_1G_Polymyxin            cggttggagctgttgatcacgagctcggacagctgcagcgtggcggattaccggaccttg 9378
pmxA-D-DaB3-Polymyxin-B      TGGATGGAGCTGTTGATCACGAGCTCGGACAGCTGCAGCGTGGCGGATTACCGGACCTTG 9267
pmxA_B-LR_Polymyxin-E        TGGATGGAGCTGTTGATCACGAGTTCGGACAGCTGCAGCGTGGCGGATTACCGGATCTTG 9441
pmxA_KP26_Polymyxin-E        TGGATGGAGCTGTTGATCACGAGTTCGGACAGCTGCAGCGTGGCGGATTACCGGATCTTG 9441
                              ** ******************* ******************************* ****

pmxA_Polymyxin-P             CAGGAACGCTTCGGCTCGTTGTTCCGGATCATCAACGCATACGGCGTAACGGAAGCGGCG 9537
pmxA_Polymyxin-A             CAGGAACGCTTCGGCTCGTTGTTCCGGATCATCAACGCCTACGGCGTGACGGAAGCGGCG 9447
pmxA_1G_Polymyxin            caggaacgcttcggctcgttgttccggatcatcaacgcttacggcgtgacggaagcggcg 9438
pmxA-D-DaB3-Polymyxin-B      CAGGAACGCTTCGGCTCGTTGTTCCGGATTATCAACGCATACGGCGTGACGGAAGCGGCG 9327
pmxA_B-LR_Polymyxin-E        CAGGAACGTTTCGGCTCGTTCTTCCGGATCATCAACGCATACGGCGTGACGGAAGCGGCG 9501
pmxA_KP26_Polymyxin-E        CAGGAACGTTTCGGCTCGTTCTTCCGGATCATCAACGCATACGGCGTGACGGAAGCGGCG 9501
                             ******** *********** ******** ******** ******** ************
```

```
pmxA_Polymyxin-P            ATCGACTCCAGCTTCTACGAAGAGGAGCTGGCGAAGCTGCCGCAGACAGGCAATGTGCCG 9597
pmxA_Polymyxin-A            ATCGACTCCAGCTTCTACGACGAGGAGCTGACGAAGCTGCCGCAGACAGGTCATGTGCCG 9507
pmxA_1G_Polymyxin           atcgactcaagcttctacgacgaggagctggcgaagctgccgcagacaggccatgtgccg 9498
pmxA-D-DaB3-Polymyxin-B     ATCGACTCAAGTTTCTACGACGAGCCGCTGACGAAGCTGCCGCAGACAGGCAATGTGCCG 9387
pmxA_B-LR_Polymyxin-E       ATCGACTCCAGCTTCTACGACGAGGAGCTGACGAAGCTGCCGCAGATAGGCCATGTACCG 9561
pmxA_KP26_Polymyxin-E       ATCGACTCCAGCTTCTACGACGAGGAGCTGACGAAGCTGCCGCAGATAGGCCATGTACCG 9561
                            ******** ** ******** ***  **** *************** ***  **** ***

pmxA_Polymyxin-P            ATCGGTAAAGCGTGGCTGAATGCGAAATTCTACATCGTGGACGCGCATCTGAACCCGGTG 9657
pmxA_Polymyxin-A            ATCGGCAAAGCGTGGCTGAATGCGAAATTCTACATCGTGGACGCGCATCTGAACCCGGTG 9567
pmxA_1G_Polymyxin           attggcaaagcgtggctgaatgcgaaattctacatcgtggacgcgcatctgaacccggtg 9558
pmxA-D-DaB3-Polymyxin-B     ATCGGCAAAGCATGGCTGAATGCGAAATTCTACATCGTGGACGCGCATCTGAACCCGGTG 9447
pmxA_B-LR_Polymyxin-E       ATTGGAAAAGCGTGGCTGAATGCGAAATTCTACATCGTGGATGCGCATCTGAACCCGGTG 9621
pmxA_KP26_Polymyxin-E       ATTGGAAAAGCGTGGCTGAATGCGAAATTCTACATCGTGGATGCGCATCTGAACCCGGTG 9621
                            ** ** ***** ***************************** ******************

pmxA_Polymyxin-P            CCGATTGGGGTGCTAGGCGAGCTGGTCATCGGCGGAGTCGGAGTGGCGCGCGGGTACTTG 9717
pmxA_Polymyxin-A            CCGGTCGGGGTGCTGGGCGAGCTGGTCATCGGCGGAGTCGGAGTGGCGCGCGGGTACTTG 9627
pmxA_1G_Polymyxin           ccggtcggggtgctgggcgagctggtcatcggcggagtcggagtggcgcgcgggtacttg 9618
pmxA-D-DaB3-Polymyxin-B     CCGGTCGGGGTGCTGGGCGAGCTGGTTATCGGCGGAGTTGGGGTAGCGCGCGGGTACTTG 9507
pmxA_B-LR_Polymyxin-E       CCGGTCGGGGTGCTGGGCGAGCTGGTCATCGGCGGAGTCGGTGTGGCGCGAGGGTATTTG 9681
pmxA_KP26_Polymyxin-E       CCGGTCGGGGTGCTGGGCGAGCTGGTCATCGGCGGAGTCGGTGTGGCGCGAGGGTATTTG 9681
                            *** * ******** *********** *********** ** ** ***** ***** ***

pmxA_Polymyxin-P            AACCGTCCGGAGCTGACGGAAGAGAAGTTCGTAGACAGCCCGTTCGCCGCGGGCGAGCGG 9777
pmxA_Polymyxin-A            AACCGTCCGGAGCTGACGGAAGAGAAGTTCGTAGACAGTCCGTTCGCCGCGGGCGAGCGG 9687
pmxA_1G_Polymyxin           aaccgtccggagctgacagaagagaagttcgtagacagcccgttcgccgcgggcgagcgg 9678
pmxA-D-DaB3-Polymyxin-B     AACCGTCCGGAGCTGACGGAAGAGAAGTTCGTAGACAGCCCGTTCGCCGCGGGCGAGCGG 9567
pmxA_B-LR_Polymyxin-E       AACCGTCCGGAGCTGACGGAAGAGAAGTTCGTAGACAGTCCGTTCGCCGCGGGCGAGCGG 9741
pmxA_KP26_Polymyxin-E       AACCGTCCGGAGCTGACGGAAGAGAAGTTCGTAGACAGTCCGTTCGCCGCGGGCGAGCGG 9741
                            ***************** ******************** *********************

pmxA_Polymyxin-P            TTGTACCGCACGGGAGACTTGGCGCGGTGGATGGAGGACGGAACGTGGACTTCATCGGC 9837
pmxA_Polymyxin-A            CTGTACCGCACGGGAGACTTGGCGCGGTGGATGGAGGACGGAACGTGGACTTCATCGGC 9747
pmxA_1G_Polymyxin           ctgtaccgcacgggagacttggcgcggtggatggaggacggcaacgtggacttcatcggc 9738
pmxA-D-DaB3-Polymyxin-B     CTGTACCGCACGGGAGACTTGGCGCGGTGGATGGAAGACGGCAACGTGGACTTCATCGGC 9627
pmxA_B-LR_Polymyxin-E       CTGTACCGCACGGGAGACTTGGCGCGGTGGATGGAGGACGGAACGTGGACTTCATCGGC 9801
pmxA_KP26_Polymyxin-E       CTGTACCGCACGGGAGACTTGGCGCGGTGGATGGAGGACGGAACGTGGACTTCATCGGC 9801
                             ********************************** ***** ******************

pmxA_Polymyxin-P            CGGATCGACAACCAGGCGAAAATCAGGGGGTACCGGATCGAGACGGGCGAGATCGAGTCG 9897
pmxA_Polymyxin-A            CGGATCGACAACCAGGCGAAAATCCGGGGGTACCGGATCGAGACGGGCGAGATCGAGTCG 9807
pmxA_1G_Polymyxin           cggatcgacaaccaggcgaaaatccggggctaccggatcgagacgggcgagatcgagtcg 9798
```

```
pmxA-D-DaB3-Polymyxin-B      CGGATCGACAACCAGGCGAAAATCCGGGGCTACCGGATCGAGACGGGCGAGATCGAGTCG 9687

pmxA_B-LR_Polymyxin-E        CGGATCGACAACCAAGCGAAAATCCGGGGCTACCGGATCGAGACGGGTGAGATCGAGTCG 9861

pmxA_KP26_Polymyxin-E        CGGATCGACAACCAAGCGAAAATCCGGGGCTACCGGATCGAGACGGGTGAGATCGAGTCG 9861

                             ************** ********* **** ***************** ************


pmxA_Polymyxin-P             CAGCTGCTGCGGGTGAAAGGCGTGCGCGAAGCGGTGGTGCTGGTTCGAAGTGACGCGAAC 9957

pmxA_Polymyxin-A             CAGCTGCTGCGGGTGGAAGGCGTGCGCGAAGCAGTGGTGCTGGTTCGAAGTGACGCGAAC 9867

pmxA_1G_Polymyxin            cagctgctgcgggtggaaggtgtgcgcgaagcggtggtgctggttcgaagtgacgcgaac 9858

pmxA-D-DaB3-Polymyxin-B      CAGCTGCTGCGGGTGGAAGGCGTGCGCGAAGCGGTGGTGCTGGTTCGAAGTGACGCGAAC 9747

pmxA_B-LR_Polymyxin-E        CAGCTGCTGCGGGTGGAAGGCGTGCGCGAAGCGGTGGTGCTGGTTCGAAGTGACGCAAAC 9921

pmxA_KP26_Polymyxin-E        CAGCTGCTGCGGGTGGAAGGCGTGCGCGAAGCGGTGGTGCTGGTTCGAAGTGACGCAAAC 9921

                             *************** **** *********** *********************** ***


pmxA_Polymyxin-P             GGGCAGAAGGCGCTATGCGCGTATTACACGCCGGATACCGGAGCGGAGTTGGCAGTGAAC 10017

pmxA_Polymyxin-A             GGGCAGAAGGCGCTATGCGCGTATTACACGCCGGATACCGGAGCGGAGCTGGCGGTGAAC 9927

pmxA_1G_Polymyxin            gggcagaaggcgctatgcgcgtattacacgccggataccggagcggagctggcggtgaac 9918

pmxA-D-DaB3-Polymyxin-B      GGGCAGAAGGCGCTATGCGCGTATTGCACGCCGGGTACCGGAGCGGAGCTGGCGGTGAAC 9807

pmxA_B-LR_Polymyxin-E        GGGCAGAAGGCGTTATGCGCGTATTACACGCTGGATACCGGAGCGGAACTGGCAGTGAAC 9981

pmxA_KP26_Polymyxin-E        GGGCAGAAGGCGTTATGCGCGTATTACACGCTGGATACCGGAGCGGAACTGGCAGTGAAC 9981

                             ************ ************ ***** ** ************  **** ******


pmxA_Polymyxin-P             GATCTGCGCAGCGCGCTGGCGCAGGAGCTGCCGGGGTACATGATCCCGTCGTACTTCGTG 10077

pmxA_Polymyxin-A             GATTTGCGCGGCGCGCTGGCGCAGGAGCTGCCGGGCTACATGATCCCGTCGTACTTCGTG 9987

pmxA_1G_Polymyxin            gatttgcgcagcgcgctggcgcaggagctgccgggctacatgatcccgtcgtacttcgtg 9978

pmxA-D-DaB3-Polymyxin-B      GATTTGCGCAGCGCGCTGGCGCAGGAGCTGCCGGGCTACATGATCCCGTCGTACTTCGTG 9867

pmxA_B-LR_Polymyxin-E        GATCTGCGCAGCACGCTGGCGCAGGAGCTGCCGGGCTACATGATCCCGTCGTACTTCGTG 10041

pmxA_KP26_Polymyxin-E        GATCTGCGCAGCACGCTGGCGCAGGAGCTGCCGGGCTACATGATCCCGTCGTACTTCGTG 10041

                             *** ***** ** ********************** ************************


pmxA_Polymyxin-P             GAGCTGGAGCGCCTGCCGCTGACGCCGAACGGAAAGATTGACCGGAAGGCGCTGCCGGCG 10137

pmxA_Polymyxin-A             GAGCTGGAGCGTCTACCTCTGACGCCGAACGGAAAGATTGACCGGAAGGCGCTGCCGGCG 10047

pmxA_1G_Polymyxin            gagctggagcgcctgcctctgacgccgaacggaaagattgaccggaaggcgctgccggcg 10038

pmxA-D-DaB3-Polymyxin-B      GAGCTGGAGCGCCTGCCTCTGACGCCGAACGGAAAGATTGACCGGAAGGCGTTGCCAGCG 9927

pmxA_B-LR_Polymyxin-E        GAGCTGGAGGGCCTGCCTCTGACGCCGAACGGAAAGATTGACCGGAAGGCGCTGCCGGCG 10101

pmxA_KP26_Polymyxin-E        GAGCTGGAGGGCCTGCCTCTGACGCCGAACGGAAAGATTGACCGGAAGGCGCTGCCGGCG 10101

                             ********* * ** ** ********************************* **** ***


pmxA_Polymyxin-P             CCGGAAGGGGAAGCGGGAAGCGGAACGGAGTACGTCGCACCGCGCAATGAGCTGGAAACG 10197

pmxA_Polymyxin-A             CCGGAAAGGGAAGCGGGAAGCGGAACGGAGTACGTCGCACCGCGCAATGAGCTGGAAACG 10107

pmxA_1G_Polymyxin            ccggaaggggaagcgggaagcggaacggagtacgtcgcaccgcgcaatgagctggaaacg 10098

pmxA-D-DaB3-Polymyxin-B      CCGGAAGGGGAAGCGGGAAGCGGAACGGAGTACGTCGCACCGCGCAATGAGCTGGAAACG 9987

pmxA_B-LR_Polymyxin-E        CCGGAAGGAGAAGCGGGAAGCGGAACGGAGTACGTCGCACCGCGCAATGAGCTGGAAACA 10161

pmxA_KP26_Polymyxin-E        CCGGAAGGAGAAGCGGGAAGCGGAACGGAGTACGTCGCACCGCGCAATGAGCTGGAAACA 10161

                             ****** * **************************************************
```

```
pmxA_Polymyxin-P              AAGCTGGCAGTGATTTGGCAGGAGGTGCTGGGGCTTGCGAAGGAGATTGGCGTTCACGAC 10257
pmxA_Polymyxin-A              AAGCTGACGGCGATTTGGCAGGAGGTGCTGGGGCTTGCGAAGGAGATTGGCGTTCACGAC 10167
pmxA_1G_Polymyxin             aagctggcggcgatttggcaggaggtgctggggcttgcgaaggagattggcattcacgac 10158
pmxA-D-DaB3-Polymyxin-B       AAGCTGGCGGCGATTTGGCAGGAGGTGCTGGGGCTTGCGAAGGAGATTGGCGTTCACGAC 10047
pmxA_B-LR_Polymyxin-E         AAGCTGGCGGCGATTTGGCAGGAGGTGCTGGGGCTTGCGAAGGAGATTGGCGTTCACGAC 10221
pmxA_KP26_Polymyxin-E         AAGCTGGCGGCGATTTGGCAGGAGGTGCTGGGGCTTGCGAAGGAGATTGGCGTTCACGAC 10221
                              ****** * * **************************************** ********

pmxA_Polymyxin-P              AACTTCTTCGACATCGGCGGCCACTCCCTGCGGGCGACGACGCTGGTCAGCAAGATTCAT 10317
pmxA_Polymyxin-A              AACTTCTTCGACATCGGCGGCCACTCCCTGCGAGCGACGACGCTGGTCAGCAAGATTCAT 10227
pmxA_1G_Polymyxin             aacttcttcgacatcggcggccactccctgcgggcgacgacgctggtcagcaagattcat 10218
pmxA-D-DaB3-Polymyxin-B       AACTTCTTCGACATCGGCGGCCACTCCCTGCGGGCGACGACGCTGGTCAGCAAGATTCAT 10107
pmxA_B-LR_Polymyxin-E         AACTTCTTCGACATCGGCGGCCACTCCCTGCGGGCGACGACGCTGGTCAGCAAGGTGCAC 10281
pmxA_KP26_Polymyxin-E         AACTTCTTCGACATCGGCGGCCACTCCCTGCGGGCGACGACGCTGGTCAGCAAGGTGCAC 10281
                              ******************************** ********************* * **

pmxA_Polymyxin-P              AAAGAGTTGAACGTGGATCTGCCGCTGCGCGACGTGTTCCGCCATTCCACCATCGAGAGC 10377
pmxA_Polymyxin-A              AAAGAGTTGAACGTGGATCTGCCGCTGCGCGACGTGTTCCGCCATTCCACTATCGAGAGC 10287
pmxA_1G_Polymyxin             aaagagttgaacgtggatctgccgctgcgtgacgtgttccgccattccatgatcgagagc 10278
pmxA-D-DaB3-Polymyxin-B       AAAGAGTTGAACGTGGATCTGCCGCTGCGCGACGTGTTCCGCCATTCCACGATCGAGAGC 10167
pmxA_B-LR_Polymyxin-E         AAGGAACTGAGCGTGGATCTGCCGCTGCGCGACGTGTTCCGCCATTCCACGATCGAGAGC 10341
pmxA_KP26_Polymyxin-E         AAGGAACTGAGCGTGGATCTGCCGCTGCGCGACGTGTTCCGCCATTCCACGATCGAGAGC 10341
                              ** **  *** ****************** *******************  *********

pmxA_Polymyxin-P              ATGGCGGCCGCCATTTCCAGGCTGGATGAGCAGACATTCGTTGCCATTCCGGTGGCGGAT 10437
pmxA_Polymyxin-A              ATGGCGGCCGCCATTTCCCGGCTGGATGAGCAGACATTCGTTGCCATTCCGGTGGCGGAT 10347
pmxA_1G_Polymyxin             atggcatccgccatttcccggctggatgagcagacattcgttgccattccggtggcggat 10338
pmxA-D-DaB3-Polymyxin-B       ATGGCGGCCGCCATTTCCCGGCTGGATGAGCAGACATTCGTTGCCATTCCGGTGGCGGAT 10227
pmxA_B-LR_Polymyxin-E         ATGGCGGCCGCCATTTCCCGGCTGGATGAGCAGACATTCGTTGCCATTCCGGTGGCGGAT 10401
pmxA_KP26_Polymyxin-E         ATGGCGGCCGCCATTTCCCGGCTGGATGAGCAGACATTCGTTGCCATTCCGGTGGCGGAT 10401
                              *****  *********** *****************************************

pmxA_Polymyxin-P              GACCGAGAGATGTACCCGCAATCTTTTGCTCAAAAACGTCTCTTTATCCTGAATCAACTG 10497
pmxA_Polymyxin-A              GACCGAGAGGTGTACCCGCAATCTTTTGCTCAAAAACGTCTCTTTATCCTGAATCAACTG 10407
pmxA_1G_Polymyxin             gaccgggaggtgtacccgcaatcttttgctcaaaaacgtctctttatcctgaatcaactg 10398
pmxA-D-DaB3-Polymyxin-B       GGCCGAGAGGTGTACCCGCAATCTTTTGCTCAAAAACGTCTCTTTATCCTGAATCAACTG 10287
pmxA_B-LR_Polymyxin-E         GACCGGGAGGTGTACCCGCAATCTTTTGCTCAAAAACGTCTCTTTATCCTGAATCAACTG 10461
pmxA_KP26_Polymyxin-E         GACCGGGAGGTGTACCCGCAATCTTTTGCTCAAAAACGTCTCTTTATCCTGAATCAACTG 10461
                              * *** *** **************************************************

pmxA_Polymyxin-P              GAAGGCGCGGAGCTTAGCTACAACATGCCGGAAGCGATGCTGCTGGAGGGTGCTTTGGAC 10557
pmxA_Polymyxin-A              GAAGGCGCAGAGCTTAGCTACAACATGCCGGAAGCGATGCTGCTGGAGGGTGCTTTGGAC 10467
pmxA_1G_Polymyxin             gaaggcgcggagcttagctacaacatgccggaagcgatgctgctggagggtgctttggat 10458
```

```
pmxA-D-DaB3-Polymyxin-B      GAAGGCGCGGAGCTTAGCTACAACATGCCGGAAGCGATGCTGCTGGAGGGTGCTTTAGAC 10347
pmxA_B-LR_Polymyxin-E        GAAGGCGCGGAGCTTAGCTACAACATGCCGGAGGCGATGCTGCTGGAGGGGGCTTTGGAT 10521
pmxA_KP26_Polymyxin-E        GAAGGCGCGGAGCTTAGCTACAACATGCCGGAGGCGATGCTGCTGGAGGGGGCTTTGGAT 10521
                             ******** *********************** ***************** ***** ** 

pmxA_Polymyxin-P             CGGGCAAGGTTCGAAGAAGCGTTCCGTAAGCTCGTGGCGCGGCATGAAACGCTGCGCACC 10617
pmxA_Polymyxin-A             CGGACAAGGTTCGAAGAAGCGTTCCGTAAGCTCGTGGCGCGGCATGAAACGCTGCGCACC 10527
pmxA_1G_Polymyxin            cggacaaggttcgaagaagcgttccgtaagctcgtggcgcggcatgaaatgctgcgcacc 10518
pmxA-D-DaB3-Polymyxin-B      CGGGCAAGGTTCGAAGAAGCGTTCCGTAAGCTCGTGGCGCGGCATGAAATGCTGCGCACC 10407
pmxA_B-LR_Polymyxin-E        CGGGCAAGGTTCGAAGAAGCATTCCGTAAGCTCGTGGCGCGGCATGAAATGCTGCGCACC 10581
pmxA_KP26_Polymyxin-E        CGGGCAAGGTTCGAAGAAGCATTCCGTAAGCTCGTGGCGCGGCATGAAATGCTGCGCACC 10581
                             *** **************** **************************** **********

pmxA_Polymyxin-P             GGGTTCGAAATGGTGGATGGCGAAGCATCGCAGCGGATTTACCAAGACGTGAATTTTGCC 10677
pmxA_Polymyxin-A             GGGTTCGAAATGGTGGATGGCGAAGCATCGCAGCGGATTTACCAGGACGTGAATTTTGCC 10587
pmxA_1G_Polymyxin            gggttcgaaatggtggatggcgaagcatcgcagcggttataccatgacgtgaattttgct 10578
pmxA-D-DaB3-Polymyxin-B      GGGTTCGAAATGGTGGATGGCGAAGCATCGCAGCGGATTTACCAGGACGTGAATTTTGCC 10467
pmxA_B-LR_Polymyxin-E        GGGTTCGAAATGGTGGATGGCGAAGCATCGCAGCGGGTTTACCAGGACTTGAATTTTGCT 10641
pmxA_KP26_Polymyxin-E        GGGTTCGAAATGGTGGATGGCGAAGCATCGCAGCGGGTTTACCAGGACTTGAATTTTGCT 10641
                             ************************************ * ***** *** ********** 

pmxA_Polymyxin-P             GTGGAGTTCTATCGAGTGGATGAGCAAGAGGCCGAAGAGATGGTTCGCCGTTTCGTCCGT 10737
pmxA_Polymyxin-A             GTGGAGTTCTATCGAGTGGATGAGCAAGAGGCCGAAGAGACGGTTCACCGTTTCGTCCGT 10647
pmxA_1G_Polymyxin            gtggagttctatcgagtggatgagcaagaggccgaagagacggttcgccgtttcgtccgt 10638
pmxA-D-DaB3-Polymyxin-B      GTGGAGTTCTATCGAGCGGATGAGCAAGAGGCCGAAGAGACGGTTCGCCGTTTCGTCCGT 10527
pmxA_B-LR_Polymyxin-E        GTGGAGTTCTATCGAGTAGATGAGCAAGAGGCCGAAGAGACGGTTCGCCGTTTTGTCCGT 10701
pmxA_KP26_Polymyxin-E        GTGGAGTTCTATCGAGTAGATGAGCAAGAGGCCGAAGAGACGGTTCGCCGTTTTGTCCGT 10701
                             ****************  ********************** ***** ****** ******

pmxA_Polymyxin-P             CCGTTTGACTTGGCGAAGCCTCCGCTGCTGAGGGTAGGCCTTGTCGAGCTGGCTCCAGAA 10797
pmxA_Polymyxin-A             CCGTTTGACTTGGCGAAGCCTCCGCTGCTGAGGGTAGGCCTTGTCGAGCTGGCTCCGGAA 10707
pmxA_1G_Polymyxin            tcgtttgacttggcgaagcctccgctgctgagggtaggccttatcgagctggctccggaa 10698
pmxA-D-DaB3-Polymyxin-B      CCGTTTGACTTGGCGAAGCCTCCGCTGCTGAGGGTAGGCCTTGTTGAGCTGGCTCCGGAA 10587
pmxA_B-LR_Polymyxin-E        CCGTTTGACTTGGCGAAGCCTCCGCTGCTGAGGGTAGGCCTTGTCGAGCTGGCTTCGGAA 10761
pmxA_KP26_Polymyxin-E        CCGTTTGACTTGGCGAAGCCTCCGCTGCTGAGGGTAGGCCTTGTCGAGCTGGCTTCGGAA 10761
                              ***************************************** * ********* * ***

pmxA_Polymyxin-P             CGTCATATTCTAATGTACGACATGCATCATATTATTTCCGATGGCGTCTCGATGGAAATC 10857
pmxA_Polymyxin-A             CGCCATATTCTAATGTACGACATGCATCATATTATTTCCGACGGCGTCTCGATGGAAATC 10767
pmxA_1G_Polymyxin            cgccatattctaatgtacgacatgcatcatattatttccgacggcgtctcgatggaaatc 10758
pmxA-D-DaB3-Polymyxin-B      CGCCATATTCTAATGTACGACATGCATCATATTATTTCCGACGGCGTCTCGATGGAAATC 10647
pmxA_B-LR_Polymyxin-E        CGCCATATTCTAATGTACGACATGCATCATATTATTTCCGACGGTGTCTCCATGGAAATC 10821
pmxA_KP26_Polymyxin-E        CGCCATATTCTAATGTACGACATGCATCATATTATTTCCGACGGTGTCTCCATGGAAATC 10821
                             ** ************************************** ** ***** *********
```

```
pmxA_Polymyxin-P              TTTGTTGAAGAATTCGTCCGCTTGTACGGCGGCGAGCAATTGGAGCCTCTGCGCATTCAG 10917
pmxA_Polymyxin-A              TTTGTTGAAGAATTCGTCCGCTTGTACGGCGGCGAGCAATTGGAGCCTCTGCGCATTCAG 10827
pmxA_1G_Polymyxin             tttgttgaagaattcgtccgcttgtacggcggcgagcaattggagcctctgcgcgttcag 10818
pmxA-D-DaB3-Polymyxin-B       TTTGTTGAAGAATTCGTCCGCTTGTACGGCGGCGAGCAATTGGAGCCTCTGCGCATTCAG 10707
pmxA_B-LR_Polymyxin-E         TTTGTTGAAGAATTCGTCCGCTTGTACGGCGGTGAGCAATTGGAGCCTCTTCGCATTCAG 10881
pmxA_KP26_Polymyxin-E         TTTGTTGAAGAATTCGTCCGCTTGTACGGCGGTGAGCAATTGGAGCCTCTTCGCATTCAG 10881
                              ******************************** ***************** *** *****

pmxA_Polymyxin-P              TACAAAGACTACACCGTTTGGCAGCATTCGCAGGAGCAGAAGGAACGGCTTCAGCGTCAG 10977
pmxA_Polymyxin-A              TACAAAGACTACACCGTTTGGCAGCATTCACAGGAGCAGAAAGAACGGCTTCAGCGTCAG 10887
pmxA_1G_Polymyxin             tacaaagactacaccgtttggcagcattcgcaggagcagaaggaacggcttcagcgtcag 10878
pmxA-D-DaB3-Polymyxin-B       TACAAAGACTACACCGTTTGGCAGCATTCGCAGGAGCAGAAGGAACGGCTTCAGCGTCAG 10767
pmxA_B-LR_Polymyxin-E         TACAAAGACTACACAGTTTGGCAGCATTCGCAGGAGCAGAAGGAACGGCTTCAGCGTCAG 10941
pmxA_KP26_Polymyxin-E         TACAAAGACTACACAGTTTGGCAGCATTCGCAGGAGCAGAAGGAACGGCTTCAGCGTCAG 10941
                              ************** ************** *********** ******************

pmxA_Polymyxin-P              GAGGCGTACTGGCTGGACATGTTCCAAGGCGAGCTTCCGGTGCTGGAAATGCCGACCGAT 11037
pmxA_Polymyxin-A              GAGGCGTACTGGCTGGACATGTTCCAAGGCGAGCTTCCGGTGCTGGAAATGCCGACCGAC 10947
pmxA_1G_Polymyxin             gaggcgtactggctggacatgttccaaggcgagcttccggtgctggaaatgccgacggac 10938
pmxA-D-DaB3-Polymyxin-B       GAGGCGTACTGGCTGGACATGTTCCAAGGCGAGCTTCCGGTGCTGGAAATGCCGACCGAC 10827
pmxA_B-LR_Polymyxin-E         GAGGCGTACTGGCTGAACATGTTCCAAGGCGAGCTTCCGGTGCTGGAAATGCCAACCGAC 11001
pmxA_KP26_Polymyxin-E         GAGGCGTACTGGCTGAACATGTTCCAAGGCGAGCTTCCGGTGCTGGAAATGCCAACCGAC 11001
                              *************** ************************************* ** ** 

pmxA_Polymyxin-P              TATCCGCGTCCGTCCGTGCAGAGCTACGAAGGCCAAACGCTGGAGTTTTTCTTCGACGCT 11097
pmxA_Polymyxin-A              TATCCGCGTCCGGCCGTGCAGAGCTACGAAGGCCAAACGCTGGAGTTTTTCTTCGACGCT 11007
pmxA_1G_Polymyxin             tatccgcgtccggccgtgcagagctacgaaggccaaacgctggagtttttcttcgacgct 10998
pmxA-D-DaB3-Polymyxin-B       TATCCGCGTCCGGCCGTGCAGAGCTACGAAGGCCAAACGCTGGAGTTTTTCTTTGACGCT 10887
pmxA_B-LR_Polymyxin-E         TATCCGCGTCCGTCCGTGCAGAGCTACGAAGGCCACACGCTGGAGTTTTTCTTCGACGCT 11061
pmxA_KP26_Polymyxin-E         TATCCGCGTCCGTCCGTGCAGAGCTACGAAGGCCACACGCTGGAGTTTTTCTTCGACGCT 11061
                              ************ ********************** ***************** ******

pmxA_Polymyxin-P              TCGAAAACCGACGGCCTAAAGCAGCTGGCCTCGGAAACGGGCACGACGCTGTTTATGGTG 11157
pmxA_Polymyxin-A              TCGAAAACCGACGGCCTGAAGCAGCTGGCCTCGGAAACGGGCACGACGCTGTTTATGGTG 11067
pmxA_1G_Polymyxin             tcgaaaaccgacggcctgaagcagctggcctcggaaacgggcacaacgctgtttatggtg 11058
pmxA-D-DaB3-Polymyxin-B       TCGAAAACCGACGGCCTGAAGCAGCTGGCCTCGGAAACGGGCACGACGCTGTTTATGGTG 10947
pmxA_B-LR_Polymyxin-E         TCGAAAACCGACGGCCTGAAGCAACTGGCCTCGGAAACGGGCACGACGCTGTTTATGGTG 11121
pmxA_KP26_Polymyxin-E         TCGAAAACCGACGGCCTGAAGCAACTGGCCTCGGAAACGGGCACGACGCTGTTTATGGTG 11121
                              ***************** ***** ******************** ***************

pmxA_Polymyxin-P              CTGCTTGCGGCGTATAACGTCCTTCTGCATAAATATTCAGGTCAGGAAGACGTGATCGTC 11217
pmxA_Polymyxin-A              CTGCTTGCGGCGTATAACGTCCTTCTGCATAAATATTCAGGTCAGGAAGACGTGATCGTC 11127
pmxA_1G_Polymyxin             ctgcttgcggcgtataacgtccttctgcataaatattcaggtcaggaagacgtgatcgtc 11118
```

```
pmxA-D-DaB3-Polymyxin-B      CTGCTTGCGGCGTATAACGTCCTTCTGCATAAATATTCAGGTCAGGAAGACGTGATCGTC 11007
pmxA_B-LR_Polymyxin-E        CTGCTTGCGGCGTATAACGTCCTTCTGCATAAATATTCAGGTCAGGAAGATGTGATCGTT 11181
pmxA_KP26_Polymyxin-E        CTGCTTGCGGCGTATAACGTCCTTCTGCATAAATATTCAGGTCAGGAAGATGTGATCGTT 11181
                             ************************************************** ********

pmxA_Polymyxin-P             GGTACGCCGATTGCCGGAAGGAATCATGGAGATGTGCAGCCGTTGATCGGGATTTTCTTA 11277
pmxA_Polymyxin-A             GGTACGCCGATTGCCGGAAGGAATCATGGAGATGTGCAGCCGTTGATCGGGATTTTCTTA 11187
pmxA_1G_Polymyxin            ggtacgccgattgccggaaggaatcatggggatgtgcagccgttgatcgggattttctta 11178
pmxA-D-DaB3-Polymyxin-B      GGTACGCCGATTGCCGGAAGGAATCATGGAGATGTGCAGCAGTTGATCGGGATTTTCTTA 11067
pmxA_B-LR_Polymyxin-E        GGTACGCCGATTGCCGGAAGGAATCATGGAGATGTGCAGCCGTTGATCGGAATGTTCTTA 11241
pmxA_KP26_Polymyxin-E        GGTACGCCGATTGCCGGAAGGAATCATGGAGATGTGCAGCCGTTGATCGGAATGTTCTTA 11241
                             ***************************** ********** ********* ** ******

pmxA_Polymyxin-P             AACACGCTGGCTATCCGCAGTTATCCGGCTTCGGAGAAGACATTCCTGTCATACCTGAAC 11337
pmxA_Polymyxin-A             AACACGCTGGCTATCCGCAGTTATCCGGCTTCGGAGAAGACATTCCTGTCATACCTGAAC 11247
pmxA_1G_Polymyxin            aacacgctggctatccgcagttatccggcttcggagaagacattcctgtcatacctgaac 11238
pmxA-D-DaB3-Polymyxin-B      AACACGCTGGCTATCCGCAGTTATCCGGCTTCGGAGAAGACATTCCTGTCATACCTGAAC 11127
pmxA_B-LR_Polymyxin-E        AACACGCTGGCGATCCGCAGTTATCCGGCTTCGGAGAAGACATTCCTGTCATACCTGAAC 11301
pmxA_KP26_Polymyxin-E        AACACGCTGGCGATCCGCAGTTATCCGGCTTCGGAGAAGACATTCCTGTCATACCTGAAC 11301
                             *********** ************************************************

pmxA_Polymyxin-P             GAAGTCAAAGAAACGACCCTCCACGCCTTCGAGCATCAAAACTATCCGTTCGAAGAATTG 11397
pmxA_Polymyxin-A             GAAGTCAAAGAAACGACCCTCCACGCCTTCGAGCATCAAAACTATCCGTTCGAAGAATTG 11307
pmxA_1G_Polymyxin            gaagtcaaagaaacgaccctccacgccttcgagcatcaaaactatccgttcgaagaattg 11298
pmxA-D-DaB3-Polymyxin-B      GAAGTCAAAGAAACGACCCTCCACGCCTTCGAGCATCAAAACTATCCGTTCGAAGAATTG 11187
pmxA_B-LR_Polymyxin-E        GAAGTCAAAGAAACGACCCTCCATGCCTTCGAGCATCAAAACTATCCGTTCGAAGAATTG 11361
pmxA_KP26_Polymyxin-E        GAAGTCAAAGAAACGACCCTCCATGCCTTCGAGCATCAAAACTATCCGTTCGAAGAATTG 11361
                             *********************** ************************************

pmxA_Polymyxin-P             GTGGACAAGGTGCAAGTCACCCGTGATCTAAGCCGCAATCCGCTCTTCGACACGCTGTTT 11457
pmxA_Polymyxin-A             GTGGACAAGGTGCAAGTCACCCGTGATTTAAGCCGCAATCCACTCTTCGACACGCTGTTT 11367
pmxA_1G_Polymyxin            gtggacaaggtgcaagtcacccgtgatttaagccgcaatccgctcttcgacacgctgttt 11358
pmxA-D-DaB3-Polymyxin-B      GTGGACAAGGTGCAAGTCACCCGTGATTTAAGCCGCAATCCGCTCTTCGACACGCTGTTT 11247
pmxA_B-LR_Polymyxin-E        GTAGACAAGGTGCAAGTCACCCGTGATTTAAGCCGTAATCCGCTTTTCGACACGCTGTTT 11421
pmxA_KP26_Polymyxin-E        GTAGACAAGGTGCAAGTCACCCGTGATTTAAGCCGTAATCCGCTTTTCGACACGCTGTTT 11421
                             ** ************************ ******* ***** ** ***************

pmxA_Polymyxin-P             ACGATGCAGAATACGGAAAACGAGGAATTTGAGCTGGAAGGGCTTCGCCTGATTCCTTAT 11517
pmxA_Polymyxin-A             ACGATGCAGAATACGGAGAACGAGGAATTTGAGCTGGAAGGGCTTCGCCTGATTCCTTAT 11427
pmxA_1G_Polymyxin            acaatgcagaatacggagaacgaggaatttgagctggaagggcttcgcctgattccttat 11418
pmxA-D-DaB3-Polymyxin-B      ACGATGCAGAATACGGAGAACGAGGAATTTGAGCTGGAAGGGCTTCGCCTGATTCCTTAT 11307
pmxA_B-LR_Polymyxin-E        ACGATGCAGAATACGGAGAATGAAGAATTTGAGCTGGAAGGGCTTCGCCTGATTCCTTAT 11481
pmxA_KP26_Polymyxin-E        ACGATGCAGAATACGGAGAATGAAGAATTTGAGCTGGAAGGGCTTCGCCTGATTCCTTAT 11481
                             ** ************** ** ** ************************************
```

```
pmxA_Polymyxin-P            CCGAGCGCATTGGATACCGCGAAGTTTGATATCAGCTTGGATGTGGGCGAGGAGAACGGC 11577
pmxA_Polymyxin-A            CCGAGCGCACTGGATACCGCGAAGTTTGATATCAGCTTGGATGTGGGCGAGGAGAACGGC 11487
pmxA_1G_Polymyxin           ccgagcgcactggataccgcgaagtttgatatcagcttggatgtgggcgaggagaacggc 11478
pmxA-D-DaB3-Polymyxin-B     CCGAGCGCACTGGATACCGCGAAGTTTGATATCAGCTTGGATGTGAGCGAGGAGAACGGC 11367
pmxA_B-LR_Polymyxin-E       CCGAGCGCACTGGATACCGCAAAGTTTGATATCAGCTTGGATGTGGGCGAGGAGAACGGC 11541
pmxA_KP26_Polymyxin-E       CCGAGCGCACTGGATACCGCAAAGTTTGATATCAGCTTGGATGTGGGCGAGGAGAACGGC 11541
                            ********* ********** ************************ **************

pmxA_Polymyxin-P            GGCTTGGATTACAGCTTCGAATATGCGACGGCTCTCTACAAAAGGGAGACGATCGAACGG 11637
pmxA_Polymyxin-A            GGCTTGGATTACAGCTTCGAATATGCGACGGCTCTCTACAAAAGGGAGACGATCGAACGG 11547
pmxA_1G_Polymyxin           ggcttggattacagcttcgaatatgcgacggctctctacaaaagggagacgatcgaacgg 11538
pmxA-D-DaB3-Polymyxin-B     GGCTTGGATTACAGCTTCGAATATGCGACGGCTCTCTACAAAAGGGAGACGATCGAACGG 11427
pmxA_B-LR_Polymyxin-E       GGCTTGGATTACAGCTTCGAATATGCGACGGCTCTCTACAAAAGGGCGACGATTGAACGG 11601
pmxA_KP26_Polymyxin-E       GGCTTGGATTACAGCTTCGAATATGCGACGGCTCTCTACAAAAGGGCGACGATTGAACGG 11601
                            ********************************************** ****** ******

pmxA_Polymyxin-P            CTGGCGAAACATTACGAGCAGCTGCTCGAAACGATCGTAAGCCGTCCAGATGCGAAGATT 11697
pmxA_Polymyxin-A            CTGGCGAAACATTACGAGCAGCTGCTCGTAACGATCGTAAGCCGTCCGGATGCGAAGATC 11607
pmxA_1G_Polymyxin           ctggcgaaacattacgagcagctgctcgtaacgatcgtaagccgtccggatgcgaagatc 11598
pmxA-D-DaB3-Polymyxin-B     CTGGCAAAACATTACGAGCAGCTGCTCGTAACGATCGTAAGCCGTCCGGATGCGAAGATC 11487
pmxA_B-LR_Polymyxin-E       CTGGCGAAGCATTACGAGCAGCTGCTCGTGACGATCATAAGCCGTCCAGATGCGAAGATC 11661
pmxA_KP26_Polymyxin-E       CTGGCGAAGCATTACGAGCAGCTGCTCGTGACGATCATAAGCCGTCCAGATGCGAAGATC 11661
                            ***** ** *******************  ****** ********** *********** 

pmxA_Polymyxin-P            GCCGAGCTGAACTTGCTGACGGCAGAGGAAAAAGAGCAAATTCTCGGCGCGTTCAACCCG 11757
pmxA_Polymyxin-A            GCCGAGCTGAACTTGCTGACGGCAGAGGAAAAAGGACAAATTCTCGGCGCGTTCCACCCG 11667
pmxA_1G_Polymyxin           gccgagctgaacttgctgacggcagaggaaaaagaacaaattctcggcgcgtttaacccg 11658
pmxA-D-DaB3-Polymyxin-B     GCCGAGCTGAACTTGCTGACGGCAGAGGAAAAAGAACAAATTCTCGGCGCGTTCCACCCG 11547
pmxA_B-LR_Polymyxin-E       GCCGAGCTGAACTTGCTGACGGCAGAGGAAAAAGAACAAATTCTTGGCACATTCAACCCC 11721
pmxA_KP26_Polymyxin-E       GCCGAGCTGAACTTGCTGACGGCAGAGGAAAAAGAACAAATTCTTGGCACATTCAACCCC 11721
                            **********************************  ******** *** * **  **** 

pmxA_Polymyxin-P            GCGCAGCCGGAAGCGGCTCCTGCGGCCGCGTTCCATCGGCTGTTTGAGGAACAGGTGGAG 11817
pmxA_Polymyxin-A            GCGCAGCTGGAAGCGGCTCCTGCGGCCGCGTTCCACCGGCTGTTCGAGGAACAGGCGGAG 11727
pmxA_1G_Polymyxin           gcgcagccggaagcggcttctgcggccgcgttccactggctgttcgaggaacaggcggag 11718
pmxA-D-DaB3-Polymyxin-B     GCGCAGCCGGAAGCGGCTCCTGCGGCCGCGTTCCACTGGCTGTTCGAGGAACAGGCGGAG 11607
pmxA_B-LR_Polymyxin-E       GCGCAGCCGGAAGCGGCTCCTGCGGCCGCGTTCCACCGGCTGTTCGAGGAACAGGCGGAA 11781
pmxA_KP26_Polymyxin-E       GCGCAGCCGGAAGCGGCTCCTGCGGCCGCGTTCCACCGGCTGTTCGAGGAACAGGCGGAA 11781
                            ******* ********** ****************  ******* ********** *** 

pmxA_Polymyxin-P            CGCACGCCGGAAGAGACGGCCGTCGTGTATGAGAACGACCGGCTGACGTATGCGGAGCTG 11877
pmxA_Polymyxin-A            CGCACGCCGGAAGCGGTAGCCGTCGTGTACGAGAATGACCGGCTGACGTATGCGGAGCTG 11787
pmxA_1G_Polymyxin           cgcacgccggaagcggaggccgtcgtgtacgagaacgaccggcttacgtatgcggagctg 11778
```

```
pmxA-D-DaB3-Polymyxin-B      CGCACGCCGGAAGCGGCGGCCGTCGTGTACGAGAACGACCGGCTGACGTATGCGGAGCTG 11667
pmxA_B-LR_Polymyxin-E        CGAACGCCGGAAGAGGCGGCCGTCGTGTACGAGAACGACCAGCTGACGTATGCGGAGCTG 11841
pmxA_KP26_Polymyxin-E        CGAACGCCGGAAGAGGCGGCCGTCGTGTACGAGAACGACCAGCTGACGTATGCGGAGCTG 11841
                             ** ********** *   *********** ***** **** *** ***************

pmxA_Polymyxin-P             AACGAGCGGGCGAACCGCTTGGCGGCTACGCTGCGCGCAAGCGGCATCGGCCGGGAGACG 11937
pmxA_Polymyxin-A             AACGAGCGGGCGAACCGCTTGGCGGCTACGCTGCGCGCAAGCGGCATCGGCCGGGAGACG 11847
pmxA_1G_Polymyxin            aacgagcgggcgaaccgcttggcgggcacgctgcgcgcaagcggcatcggccgggagacg 11838
pmxA-D-DaB3-Polymyxin-B      AACGAGCGGGCGAACCGCTTGGCGGCTACGCTGCGTGCAAGCGGCATCGGCCGGGAGACG 11727
pmxA_B-LR_Polymyxin-E        AACGAGCGGGCGAACCGCTTAGCGGCCACGCTGCGCGCAAGCGACATCGGCCGGGAGACG 11901
pmxA_KP26_Polymyxin-E        AACGAGCGGGCGAACCGCTTAGCGGCCACGCTGCGCGCAAGCGACATCGGCCGGGAGACG 11901
                             ******************** ****  ******** ******* ****************

pmxA_Polymyxin-P             ATCGTCGGCATTCTCGCCGAGCGTGCGGTGGACTTGCTGGTGGCCGTGCTGGCCGTCTGG 11997
pmxA_Polymyxin-A             ATCGTCGGCATTCTGGCCGAGCGTTCGGTGGACTTGCTAGTGGCCGTGCTGGCCGTCTGG 11907
pmxA_1G_Polymyxin            atcgtcggcattctcgccgagcgttcggtggacttgctggtggccgtgctggccgtctgg 11898
pmxA-D-DaB3-Polymyxin-B      ATCGTCGGCATTCTCGCCGAGCGTTCGGTGGACTTGCTGGTGGCCGTGCTGGCCGTCTGG 11787
pmxA_B-LR_Polymyxin-E        ATCGTCGGCATTCTCGCCGAGCGTTCGGTGGATCTGCTGGTGTCCGTGCTGGCCGTCTGG 11961
pmxA_KP26_Polymyxin-E        ATCGTCGGCATTCTCGCCGAGCGTTCGGTGGATCTGCTGGTGTCCGTGCTGGCCGTCTGG 11961
                             ************** ********* *******  **** *** *****************

pmxA_Polymyxin-P             AAAGCGGGCGGGGCGTATGTGCCGCTGGACCCGGATTATCCGGCGGAGCGCGTGCGGTTC 12057
pmxA_Polymyxin-A             AAAGCGGGCGGGGCGTATGTGCCGCTCGACCCGGATTATCCGGCAGAGCGCGTGCGGTTC 11967
pmxA_1G_Polymyxin            aaagcgggcggtgcgtatgtgccgctcgacccggattatccggcggaccgcgtgcggttc 11958
pmxA-D-DaB3-Polymyxin-B      AAAGCGGGCGGGGCGTATGTGCCGCTCGACCCGGATTACCCGGCGGAGCGCGTGCGGTTC 11847
pmxA_B-LR_Polymyxin-E        AAAGCGGGCGGGGCATATGTGCCGCTCGACCCGGATTATCCGGCGGATCGCGTGCGGTTC 12021
pmxA_KP26_Polymyxin-E        AAAGCGGGCGGGGCATATGTGCCGCTCGACCCGGATTATCCGGCGGATCGCGTGCGGTTC 12021
                             *********** ** *********** *********** ***** ** ************

pmxA_Polymyxin-P             ATGCTCGAAGACAGCGGAGCGAAGGTACTGCTGACGCAAACGGCGCTGCGAGAGCGCGCC 12117
pmxA_Polymyxin-A             ATGCTCGAAGACAGCGGAGCGAAGGTGCTGCTGACGCAAACGCCGCTGCGAGAGCGTGCC 12027
pmxA_1G_Polymyxin            atgcttgaagacagcggagcgaaggtgctgctgacgcaaacgccgctgcgagagcgtgcc 12018
pmxA-D-DaB3-Polymyxin-B      ATGCTCGAAGACAGCGGAGCGAAGGTGCTGCTGACGCAAACGCCGCTGCGAGAGCGTGCC 11907
pmxA_B-LR_Polymyxin-E        ATGCTTGAAGACAGTGGAGCGAAGGTACTGTTGACGCAAATGCCGCTGCGAGAACGCGCC 12081
pmxA_KP26_Polymyxin-E        ATGCTTGAAGACAGTGGAGCGAAGGTACTGTTGACGCAAATGCCGCTGCGAGAACGCGCC 12081
                             ***** ******** *********** *** ********* * ********** ** ***

pmxA_Polymyxin-P             GAAGCCTGGCTCGGCGAAGAGGAGCTGACGCTGGCGGCGGTGCTGTACCTGGACAAAGAA 12177
pmxA_Polymyxin-A             AAAGCCTGGCTCGGCGAAGAGGAGCTGGCGCTGGCGGCGGTGCTGTACCTGGACGACGAA 12087
pmxA_1G_Polymyxin            gaagcctggctcggcgaagaggagctggcgctggcggcggtgctgtacctcgacgacgaa 12078
pmxA-D-DaB3-Polymyxin-B      GAAGCCTGGCTCGGCGAAGAGGAGCTGGCGCTGGCGGCGGTGCTGTACCTCGACGACGAA 11967
pmxA_B-LR_Polymyxin-E        GAAGCCTGGCTCGGCGAAGAGGAGCTGGCGCTGGCAGCGGTGCTGTACCTCGACGACGAA 12141
pmxA_KP26_Polymyxin-E        GAAGCCTGGCTCGGCGAAGAGGAGCTGGCGCTGGCAGCGGTGCTGTACCTCGACGACGAA 12141
                              ************************** ******* ************** *** * ***
```

```
pmxA_Polymyxin-P            ACTTCGTACAGCGAGGAGCGGGCGAATGCGCCGATTGGCTCGGGCATGGTCTCCGGCAAG 12237
pmxA_Polymyxin-A            GCGTCGTACAGCGAGGAGCGGGCGAATGC------------------------------- 12116
pmxA_1G_Polymyxin           gcgtcgtacagcgacgagcgggcgaatgcgccgctggaagcgg----------------- 12121
pmxA-D-DaB3-Polymyxin-B     GCGTCGTACAGCGAGGAGCGGACGAATGCGCCGCTGGAAGCGGAT--------------- 12012
pmxA_B-LR_Polymyxin-E       GCATCGTACAGCGAGGAGCGGGCGAATGCGCCGATTGGCTCCGGCATGGTCCCCGGCAAG 12201
pmxA_KP26_Polymyxin-E       GCATCGTACAGCGAGGAGCGGGCGAATGCGCCGATTGGCTCCGGCATGGTCCCCGGCAAG 12201
                             * *********** ****** *******

pmxA_Polymyxin-P            CTGAGGGATGCTGTGGACGACAGCGATGAGAGCCATCAGCATGTTGGCACCGACAGCTTC 12297
pmxA_Polymyxin-A            --------------------------------------------------CGACAGCTTC 12126
pmxA_1G_Polymyxin           ------------------------------------------------atgatcacttg 12132
pmxA-D-DaB3-Polymyxin-B     ---------------------------------------------------GATCACTTG 12021
pmxA_B-LR_Polymyxin-E       CTGACGGATGCTGTGGATGACGGCGATGAGACCCATCCGAATATTGGCATGGGCAGCTTC 12261
pmxA_KP26_Polymyxin-E       CTGACGGATGCTGTGGATGACGGCGATGAGACCCATCCGAATATTGGCATGGGCAGCTTC 12261
                                                                               *    ***

pmxA_Polymyxin-P            CATGAAGCCCGTCCGGAGGATTTGGCGTACGTGATCTATACGTCGGGAACGACGGGCAAG 12357
pmxA_Polymyxin-A            CATGAAGCCCGTCCAGAGGACCTGGCGTACGTGATCTATACGTCGGGAACGACGGGCAAG 12186
pmxA_1G_Polymyxin           cacgaatgtcgtccggaggacctggcgtacgtgatctacacgtcgggaacgacgggcaag 12192
pmxA-D-DaB3-Polymyxin-B     CACGAATGCCGTCCGGAGGACCTGGCGTACGTGATCTATACGTCGGGAACGACGGGCAAG 12081
pmxA_B-LR_Polymyxin-E       CATGAAGCCCGTCCGGATGATCTGGCGTATGTGATCTATACGTCGGGAACGACGGGCAAG 12321
pmxA_KP26_Polymyxin-E       CATGAAGCCCGTCCGGATGATCTGGCGTATGTGATCTATACGTCGGGAACGACGGGCAAG 12321
                            ** ***   ***** ** **  ******* ******** ********************

pmxA_Polymyxin-P            CCGAAGGGCGTGATGATCGAGCACCGTAGCCTGGTGAACACGGCGGCGGGCTACCGGCGG 12417
pmxA_Polymyxin-A            CCGAAGGGCGTGATGATCGAGCACCGCAGCCTGGTGAACACGGCGGCGAGCTACCGGCGG 12246
pmxA_1G_Polymyxin           ccgaagggcgtgatgatcgagcaccgcagcctggtgaacacggcggcgggctaccggcgg 12252
pmxA-D-DaB3-Polymyxin-B     CCGAAGGGCGTGATGATCGAGCACCGTAGCCTGGTGAACACGGCGGCGGGCTACCGGCGG 12141
pmxA_B-LR_Polymyxin-E       CCGAAAGGCGTGATGATCGAGCACCGCAGCCTGGTGAACACGGCGGCGGGCTACCGGCGG 12381
pmxA_KP26_Polymyxin-E       CCGAAAGGCGTGATGATCGAGCACCGCAGCCTGGTGAACACGGCGGCGGGCTACCGGCGG 12381
                            ***** ******************** ********************* ***********

pmxA_Polymyxin-P            GAATACCAGTTGGATCAGTTCCCGGTGCGGCTGCTGCAGCTCGCAAGCTTCTCGTTCGAC 12477
pmxA_Polymyxin-A            GAATACCGGTTGGATCAGTTCCCGGTGCGGCTGCTGCAACTCGCCAGCTTCTCGTTCGAC 12306
pmxA_1G_Polymyxin           gaataccggttggaccagttcccggtgcggctgctgcagctcgcaagcttctcgtttgac 12312
pmxA-D-DaB3-Polymyxin-B     GAATACCGGTTGGATCAGTTCCCGGTGCGGTTGCTGCAGCTCGCAAGCTTCTCGTTCGAC 12201
pmxA_B-LR_Polymyxin-E       GAATACCGGTTGGATCAGTTCCCGGTACGGCTGCTGCAGCTCGCCAGCTTCTCGTTTGAC 12441
pmxA_KP26_Polymyxin-E       GAATACCGGTTGGATCAGTTCCCGGTACGGCTGCTGCAGCTCGCCAGCTTCTCGTTTGAC 12441
                            ******* ****** *********** *** ******* ***** *********** ***

pmxA_Polymyxin-P            GTGTTCGTGGGAGATATCGCGCGGACGCTGTACAACGGAGGCACGATGGTGATTGTGCCG 12537
pmxA_Polymyxin-A            GTGTTCGTGGGAGATATCGCGCGGACGCTGTATAACGGAGGTACGATGGTGATCGTGCCG 12366
pmxA_1G_Polymyxin           gtgttcgtgggagatatcgcgcggacgctgtacaacggaggcacgatggtgattgtgccg 12372
```

```
pmxA-D-DaB3-Polymyxin-B       GTGTTCGTGGGAGATATCGCGCGGACGCTGTACAACGGAGGCACGATGGTGATTGTGCCG 12261

pmxA_B-LR_Polymyxin-E         GTGTTCGTGGGAGATATCGCGCGGACGCTGTACAACGGAGGCACGATGGTGATTGTGCCG 12501

pmxA_KP26_Polymyxin-E         GTGTTCGTGGGAGATATCGCGCGGACGCTGTACAACGGAGGCACGATGGTGATTGTGCCG 12501

                              ******************************** ******** *********** ******


pmxA_Polymyxin-P              AAGGACGACCGGATCGATCCGTCTCGTCTGCACTACTGGATGGAGCGGGAGCGGGTCACC 12597

pmxA_Polymyxin-A              AAGGACGACCGGATCGATCCGTCTCGTCTGCACTACTGGATGGAGCGGGAGCAAGTGACG 12426

pmxA_1G_Polymyxin             aaggacgatcggatcgatccgtctcgtctgcactactggattgagcgggagcgggtcacc 12432

pmxA-D-DaB3-Polymyxin-B       AAGGACGACCGGATCGATCCGTCTCGTCTGCACCACTGGATTGAGCGGGAGCGGGTCACC 12321

pmxA_B-LR_Polymyxin-E         AAGGACGACCGGATCGATCCGTCTCGTCTGCACCACTGGATGGAGCGAGAGCGGGTCACC 12561

pmxA_KP26_Polymyxin-E         AAGGACGACCGGATCGATCCGTCTCGTCTGCACCACTGGATGGAGCGAGAGCGGGTCACC 12561

                              ******** ************************ ******* ***** ****  ** **


pmxA_Polymyxin-P              ATCTTCGAATCGACACCGGCGCTGATCGTGCCGTTCCTAGAGTACGTGCACGATCAGGGG 12657

pmxA_Polymyxin-A              ATCTTTGAATCAACGCCGGCGCTGATCGTGCCGTTTATGGAATACGTGCACGAGCAAGGG 12486

pmxA_1G_Polymyxin             atcttcgaatcgacgccggcgctgatcgtgccgttcctggagtacgtgcacgagcagcaa 12492

pmxA-D-DaB3-Polymyxin-B       ATCTTCGAATCGACGCCGGCGCTGATCGTGCCGTTCCTGGAGTACGTGCACGAGCAGCAA 12381

pmxA_B-LR_Polymyxin-E         ATTTTCGAATCAACGCCGGCGCTGATCGTGCCGTTCTTAGAGTACGTGCACGAGCAGGGG 12621

pmxA_KP26_Polymyxin-E         ATTTTCGAATCAACGCCGGCGCTGATCGTGCCGTTCTTAGAGTACGTGCACGAGCAGGGG 12621

                              ** ** ***** ** ********************  * ** *********** **


pmxA_Polymyxin-P              CTGGATATGAGTCGGTTGGAGCTGTTAATCACGAGCTCGGACAGCTGCAGCGTGGCGGAT 12717

pmxA_Polymyxin-A              CTGGATATGAGCTGGATGGAACTGTTGATCACGAGCTCGGACAGCTGCAGCGTGGCGGAT 12546

pmxA_1G_Polymyxin             ctggacatgagccgggtggagctgttgatcacgagctcggacagctgcagcgtggcggat 12552

pmxA-D-DaB3-Polymyxin-B       CTGGATATGAGCCGGTTGGAGCTGTTGATCACGAGCTCGGACAGCTGCAGCGTGGCGGAT 12441

pmxA_B-LR_Polymyxin-E         CTGGATATCAGTTGGATGGAGCTGTTGATCACGAGTTCGGACAGCTGCAGCGTGGCGGAT 12681

pmxA_KP26_Polymyxin-E         CTGGATATCAGTTGGATGGAGCTGTTGATCACGAGTTCGGACAGCTGCAGCGTGGCGGAT 12681

                              ***** ** **  ** **** ***** ******** ************************


pmxA_Polymyxin-P              TACCGGACCTTGCAGGAACGCTTCGGCTCGTTGTTCCGGATCATCAACGCATACGGCGTG 12777

pmxA_Polymyxin-A              TACCGGACCTTGCAGGAACGCTTCGGCTCGTTGTTCCGGATCATCAACGCCTACGGCGTG 12606

pmxA_1G_Polymyxin             taccggaccttgcaggaacgcttcggctcgttgttccggatcatcaacgcatacggcgtg 12612

pmxA-D-DaB3-Polymyxin-B       TACCGGACCTTGCAGGAACGCTTCGGCTCGTTGTTCCGGATCATCAACGCATACGGCGTG 12501

pmxA_B-LR_Polymyxin-E         TACCGGATCTTGCAGGAACGCTTCGGCTCGTTATTCCGGATCATCAACGCATACGGCGTG 12741

pmxA_KP26_Polymyxin-E         TACCGGATCTTGCAGGAACGCTTCGGCTCGTTATTCCGGATCATCAACGCATACGGCGTG 12741

                              ******* ************************ ***************** *********


pmxA_Polymyxin-P              ACGGAAGCGGCGATCGACTCCAGCTTCTACGAAGAAGAGCTGGCGAAGCTGCCGCAGACA 12837

pmxA_Polymyxin-A              ACGGAAGCGGCGATCGACTCCAGCTTCTACGACGAGGAGCTGGCGAAGCTGCCGCAGACA 12666

pmxA_1G_Polymyxin             acggaagcggcgatcgactcaagcttctacgacgagccgctgacgaagctgccgcagaca 12672

pmxA-D-DaB3-Polymyxin-B       ACGGAAGCGGCGATCGACTCAAGCTTCTACGACGAGGAGCTGGCGAAGCTGCCGCAGACA 12561

pmxA_B-LR_Polymyxin-E         ACGGAAGCGGCGATCGACTCCAGCTTCTATGACGAGGAGCTGGCGAAGCTGCCGCAGACA 12801

pmxA_KP26_Polymyxin-E         ACGGAAGCGGCGATCGACTCCAGCTTCTATGACGAGGAGCTGGCGAAGCTGCCGCAGACA 12801

                              ******************** ******** ** **   **** *****************
```

```
pmxA_Polymyxin-P            GGCAATGTGCCGATCGGCAAAGCGTGGCTGAATGCGAAATTCTATATCGTGGACGCGCAC 12897
pmxA_Polymyxin-A            GGCCATGTGCCGATCGGCAAAGCGTGGCTGAATGCGAAATTCTACATCGTGGACGCGCAT 12726
pmxA_1G_Polymyxin           ggccatgtgccgatcggcaaagcgtggctgaatgcgaaattctacatcgtggacgcgtat 12732
pmxA-D-DaB3-Polymyxin-B     GGCAATGTGCCGATCGGCAAAGCGTGGCTGAATGCGAAATTCTACATCGTGGATGCGCAT 12621
pmxA_B-LR_Polymyxin-E       GGCCATGTACCGATTGGAAAAGCGTGGCTGAATGCGAAATTCTACATCGTGGATGCGCAT 12861
pmxA_KP26_Polymyxin-E       GGCCATGTACCGATTGGAAAAGCGTGGCTGAATGCGAAATTCTACATCGTGGATGCGCAT 12861
                            *** **** ***** ** ************************** ******** *** *

pmxA_Polymyxin-P            CTGAATCCGGTGCCGGTCGGGGTGCTGGGCGAGCTGGTCATCGGCGGAGTCGGAGTGGCG 12957
pmxA_Polymyxin-A            CTGAACCCGGTGCCGGTCGGGGTGCTGGGCGAGCTGGTCATCGGCGGAGTCGGAGTGGCG 12786
pmxA_1G_Polymyxin           ctgaacccggtgccggtcggggtgctgggcgagctggtcatcggcggagtcggggtggcg 12792
pmxA-D-DaB3-Polymyxin-B     CTGAACCCGGTGCCGGTCGGGGTGCTGGGCGAGCTGGTCATCGGCGGAGTCGGAGTGGCG 12681
pmxA_B-LR_Polymyxin-E       CTGAACCCGGTGCCGGTCGGGGTGCTGGGCGAGCTGGTAATCGGCGGAGTCGGTGTGGCG 12921
pmxA_KP26_Polymyxin-E       CTGAACCCGGTGCCGGTCGGGGTGCTGGGCGAGCTGGTAATCGGCGGAGTCGGTGTGGCG 12921
                            ***** ******************************** ************** ******

pmxA_Polymyxin-P            CGCGGGTACTTGAACCGTCCGGAGCTGACGGAAGAGAAGTTCGTAGACAGCCCGTTCGCC 13017
pmxA_Polymyxin-A            CGCGGGTACTTGAACCGTCCGGAACTGACGGGAGAGAAGTTCGTAGACAGCCCGTTCGCC 12846
pmxA_1G_Polymyxin           cgcgggtacttgaaccgtccggagttgacggaagagaagttcgtagacagcccgttcgcc 12852
pmxA-D-DaB3-Polymyxin-B     CGCGGGTACTTGAACCGTCCGGAGCTGACGGAAGAGAAGTTCGTAGACAGCCCGTTCGCC 12741
pmxA_B-LR_Polymyxin-E       CGAGGGTATTTGAACCGTCCGGAGCTGACGGAAGAGAAGTTCGTAGACAGTCCGTTCGCC 12981
pmxA_KP26_Polymyxin-E       CGAGGGTATTTGAACCGTCCGGAGCTGACGGAAGAGAAGTTCGTAGACAGTCCGTTCGCC 12981
                            ** ***** **************  ****** ****************** *********

pmxA_Polymyxin-P            GCGGGCGAGCGGTTGTACCGCACGGGAGACTTGGCGCGGTGGATGGAAGACGGCAACGTG 13077
pmxA_Polymyxin-A            GCGGGCGAGCGGCTGTACCGCACGGGAGACTTGGCGCGGTGGATGGAGGACGGGAACGTG 12906
pmxA_1G_Polymyxin           gcgggcgagcggctgtaccgcacaggagacttggcgcggtggatggaggacggcaacgtg 12912
pmxA-D-DaB3-Polymyxin-B     GCGGGCGAGCGGCTGTACCGCACGGGAGACTTGGCGCGGTGGATGGAGGACGGGAACGTG 12801
pmxA_B-LR_Polymyxin-E       GCAGGCGAGCGGCTGTACCGCACGGGAGACTTGGCGCGGTGGATGGAGGACGGGAACGTG 13041
pmxA_KP26_Polymyxin-E       GCAGGCGAGCGGCTGTACCGCACGGGAGACTTGGCGCGGTGGATGGAGGACGGGAACGTG 13041
                            ** ********* ********** *********************** ***** ******

pmxA_Polymyxin-P            GACTTCATCGGCCGGATCGACAACCAGGCGAAAATCCGGGGCTACCGGATCGAGACGGGC 13137
pmxA_Polymyxin-A            GACTTCATCGGCCGGATCGACAACCAGGCGAAAATCCGGGGCTACCGGATTGAGACGGGC 12966
pmxA_1G_Polymyxin           gacttcatcggccggatcgacaaccaggcgaaaatccggggctaccggattgagacgggc 12972
pmxA-D-DaB3-Polymyxin-B     GACTTCATCGGCCGGATCGACAACCAGGCGAAAATCCGGGGCTACCGGATCGAGACGGGC 12861
pmxA_B-LR_Polymyxin-E       GACTTCATCGGCCGGATCGACAACCAAGCGAAAATCCGGGGCTACCGGATCGAAACGGGT 13101
pmxA_KP26_Polymyxin-E       GACTTCATCGGCCGGATCGACAACCAAGCGAAAATCCGGGGCTACCGGATCGAAACGGGT 13101
                            ************************** *********************** ** *****

pmxA_Polymyxin-P            GAGATCGAGTCGCAGCTGCTGCGGGTGGAAGGCGTGCGCGAAGCGGTGGTGCTAGTTCGA 13197
pmxA_Polymyxin-A            GAGATCGAGTCGCAGCTACTGCAGGTGGAAGGCGTGCGCGAAGCGGTGGTGCTGGTTCGA 13026
pmxA_1G_Polymyxin           gagatcgagtcgcagctgctgcgggtggaaggcgtgcgcgaagcggtggtgctggttcga 13032
```

```
pmxA-D-DaB3-Polymyxin-B      GAGATCGAGTCGCAGCTGCTGCGGGTGGAAGGCGTGCGCGAAGCGGTGGTGCTGGTTCGA 12921
pmxA_B-LR_Polymyxin-E        GAGATCGAGTCGCAGCTGCTGCGGGTGGAAGGCGTGCGCGAAGCGGTGGTGCTGGTTCGA 13161
pmxA_KP26_Polymyxin-E        GAGATCGAGTCGCAGCTGCTGCGGGTGGAAGGCGTGCGCGAAGCGGTGGTGCTGGTTCGA 13161
                             ***************** **** ****************************** ******

pmxA_Polymyxin-P             AGTGACGCGAACGGGCAGAAGGCGCTATGCGCGTATTACACGCCGGATACCGGAGCGGAG 13257
pmxA_Polymyxin-A             AGTGACGCGAACGGGCAGAAGGCGCTATGCGCGTATTACACGCCGGATACCGGAGCGGAG 13086
pmxA_1G_Polymyxin            agtgacgcgaacgggcagaaggcgctatgcgcgtattacacgccggataccggagcggag 13092
pmxA-D-DaB3-Polymyxin-B      AGTGACGCGAACGGGCAGAAGGCGCTATGCGCGTATTACACGCCGGATACCGGAGCGGAG 12981
pmxA_B-LR_Polymyxin-E        AGTGACGCAAACGGGCAGAAGGCGTTATGCGCGTATTACACGCTGGATACCGGAGCGGAA 13221
pmxA_KP26_Polymyxin-E        AGTGACGCAAACGGGCAGAAGGCGTTATGCGCGTATTACACGCTGGATACCGGAGCGGAA 13221
                             ******** *************** ****************** ***************

pmxA_Polymyxin-P             CTGGCAGTGAACGATCTGCGCAGCGCGCTGGCGCAGGAGCTGCCGGGTTACATGATCCCG 13317
pmxA_Polymyxin-A             CTGGCGGTGAACGATTTGCGCGGCGCGCTGGCGCAGGAGCTGCCGGGCTACATGATCCCG 13146
pmxA_1G_Polymyxin            ctggcggtgaacgatttgcgcagcgcgctggcgcaggagctgccgggctacatgatcccg 13152
pmxA-D-DaB3-Polymyxin-B      CTGGCGGTGAACGATTTGCGCAGCGTGCTGGCGCAGGAGCTGCCGGGGTACATGATCCCG 13041
pmxA_B-LR_Polymyxin-E        CTGGCAGTGAACGATCTGCGCAGCACGCTGGCGCAGGAGCTGCCGGGCTACATGATCCCG 13281
pmxA_KP26_Polymyxin-E        CTGGCAGTGAACGATCTGCGCAGCACGCTGGCGCAGGAGCTGCCGGGCTACATGATCCCG 13281
                             ***** ********* ***** **  ********************* ************

pmxA_Polymyxin-P             TCGTACTTCGTGGAGATGGAGCGCCTGCCTCTGACGCCGAACGGAAAGATTGACCGGAAG 13377
pmxA_Polymyxin-A             TCGTACTTCGTGGAGATGGAGCGCCTGCCTCTGACGCCGAACGGAAAGATTGACCGGAAG 13206
pmxA_1G_Polymyxin            tcgtacttcgtggagatggagcgcctgcctctgacgccgaacggaaagattaaccggaag 13212
pmxA-D-DaB3-Polymyxin-B      TCGTATTTCGTGGAGCTGGAGCGCCTGCCTCTGACGCCGAACGGAAAGATTGACCGGAAG 13101
pmxA_B-LR_Polymyxin-E        TCGTACTTCGTGGAGCTGGAGGGCCTGCCTCTGACGCCGAACGGAAAGATTGACCGGAAG 13341
pmxA_KP26_Polymyxin-E        TCGTACTTCGTGGAGCTGGAGGGCCTGCCTCTGACGCCGAACGGAAAGATTGACCGGAAG 13341
                             ***** ********* ***** ***************************** ********

pmxA_Polymyxin-P             GCGCTGCCAGCGCCGGAAGGGGAAGCGGGAAGCGGAACGGAGTACGTCGCACCGCGCAAT 13437
pmxA_Polymyxin-A             GCGCTGCCGGCGCCGGAAGGGGAAGCGGGAAGCGGAACGGAGTACGTCGCACCGCGCAAT 13266
pmxA_1G_Polymyxin            gcgctgccggcgccggaaggggaagcgggaagcggaacggagtacgtcgcaccgcgcaat 13272
pmxA-D-DaB3-Polymyxin-B      GCGCTGCCAGCGCCGGAAGGGGAAGCGGGAAGTGGAACGGAGTACGTCGCACCGCGCAAT 13161
pmxA_B-LR_Polymyxin-E        GCGCTGCCGGCGCCGGAAGGAGAAGCGGGAAGCGGAACGGAGTACGTCGCACCGCGCAAT 13401
pmxA_KP26_Polymyxin-E        GCGCTGCCGGCGCCGGAAGGAGAAGCGGGAAGCGGAACGGAGTACGTCGCACCGCGCAAT 13401
                             ******** *********** *********** ***************************

pmxA_Polymyxin-P             GAGTTGGAAACGAAGCTGGCGGCGATTTGGCAGGAGGTGCTGGGGCTTGCGAAGGAGATT 13497
pmxA_Polymyxin-A             GAGCTGGAAACGAAGCTGACGGCGATTTGGCAGGAGGTGCTGGGGCTTGCGAAGGAGATT 13326
pmxA_1G_Polymyxin            gagctggaaacgaagctggcggcgatttggcaggaggtgctggggcttgcgaaggagatt 13332
pmxA-D-DaB3-Polymyxin-B      GAGCTGGAAACGAAGCTGGCGGCGATTTGGCAGGAGGTGCTGGGGCTTGCGAAGGAGGTT 13221
pmxA_B-LR_Polymyxin-E        GAGCTGGAAACAAAGCTGGCGGCGATTTGGCAGGAGGTGCTGGGGCTTGCGAAGGAGATT 13461
pmxA_KP26_Polymyxin-E        GAGCTGGAAACAAAGCTGGCGGCGATTTGGCAGGAGGTGCTGGGGCTTGCGAAGGAGATT 13461
                             *** ******* ****** ************************************** **
```

```
pmxA_Polymyxin-P            GGCGTTCACGACAACTTCTTCGACATCGGCGGCCACTCCCTGCGAGCGACGACGCTGGCG 13557
pmxA_Polymyxin-A            GGCGTTCACGACAACTTCTTCGACATCGGCGGCCACTCCCTGCGAGCGACGACGCTGGCG 13386
pmxA_1G_Polymyxin           ggcgttcacgacaacttcttcgacatcggcggccactccctgcgagcgacaacgctggcg 13392
pmxA-D-DaB3-Polymyxin-B     GGCGTTCACGACAACTTCTTCGACATCGGCGGCCACTCCCTGCGAGCGACGACGCTGGCG 13281
pmxA_B-LR_Polymyxin-E       GGCGTTTACGACAACTTCTTCGACATCGGTGGTCACTCCCTGCGGGCAACGACGCTGGCG 13521
pmxA_KP26_Polymyxin-E       GGCGTTTACGACAACTTCTTCGACATCGGTGGTCACTCCCTGCGGGCAACGACGCTGGCG 13521
                            ****** ********************** ** *********** ** ** *********

pmxA_Polymyxin-P            GGCAAGGTATTTAAGGAATTAAACGTCAACCTGCCGCTGCGCGACGTATTCCGTCACTCG 13617
pmxA_Polymyxin-A            GGCAAGGTATTTAAGGAATTAAACGTCAACCTGCCGCTGCGCGACGTATTCCGTCACTCG 13446
pmxA_1G_Polymyxin           ggcaaggtatttaaggaattaaacgtcaacctgccgctgcgcgacgtattccgtcactcg 13452
pmxA-D-DaB3-Polymyxin-B     GGCAAGGTATTTAAGGAATTAAACGTCAACCTGCCGTTGCGCGACGTATTCCGTCACTCG 13341
pmxA_B-LR_Polymyxin-E       GGCAAAGTATTTAAGGAATTAAACGTCAACCTGCCGCTGCGTGACGTATTTCGTCACTCG 13581
pmxA_KP26_Polymyxin-E       GGCAAAGTATTTAAGGAATTAAACGTCAACCTGCCGCTGCGTGACGTATTTCGTCACTCG 13581
                            ***** ****************************** **** ******** *********

pmxA_Polymyxin-P            ACGATTGCGGCGATGGCCGAGGCGATCGCCCGGATGGAACGGCGGGAGCATGAGGCCATT 13677
pmxA_Polymyxin-A            ACGATTGCGGCGATGGCCGAGGCGATCGCCCGGATGGAACGGCGGGAGCATGAGGCCATT 13506
pmxA_1G_Polymyxin           acgattgcggcgatggccgaggcgatcgcacggatggaacggcgggagcatgaggccatt 13512
pmxA-D-DaB3-Polymyxin-B     ACGATTGCGGCGATGGCCGAGGCGATCGCCCGGATGGAACGGCGGGAGCATGAGGCCATT 13401
pmxA_B-LR_Polymyxin-E       ACGATTGCAGCGATGGCCGAGGCGATCGCCCGAATGGAACGGCTGGAGCATGAGGACATT 13641
pmxA_KP26_Polymyxin-E       ACGATTGCAGCGATGGCCGAGGCGATCGCCCGAATGGAACGGCTGGAGCATGAGGACATT 13641
                            ******** ******************** ** ********** *********** ****

pmxA_Polymyxin-P            CCTCAAGCGGAGGAGAGAGAGTACTACCCTCTGTCCTCCGCGCAGAAACGGCTGTTCATT 13737
pmxA_Polymyxin-A            CCTCAAGCGGAGGAGAGAGAGTACTACCCTCTGTCCTCCGCGCAGAAACGGCTGTTCATT 13566
pmxA_1G_Polymyxin           cctcaagctgaggagagagagtactaccctctgtcctccgcccagaaacggctgttcatt 13572
pmxA-D-DaB3-Polymyxin-B     CCTCAAGCGGAGGAGAGAGAGTACTACCCTCTGTCCTCCGCGCAGAAACGGCTGTTCATT 13461
pmxA_B-LR_Polymyxin-E       CCTCAAGCGGAGGAGAGAGAGTATTACCCTCTGTCCTCTGCGCAGAAACGGCTGTTCATT 13701
pmxA_KP26_Polymyxin-E       CCTCAAGCGGAGGAGAGAGAGTATTACCCTCTGTCCTCTGCGCAGAAACGGCTGTTCATT 13701
                            ******** ************** ************** ** ******************

pmxA_Polymyxin-P            CAGCACACGCTGGATGGAGCGGATCAGCTTTACAACATGCCGGAGTTGGTGCAGGTGGAA 13797
pmxA_Polymyxin-A            CAGCACACGCTGGATGGAGCGGATCAGCTTTACAACATGCCGGAGCTGGTGCAGGTGGAA 13626
pmxA_1G_Polymyxin           cagcacacgctggatggagcggatcagctttacaacatgccggagctggtgcaggtggaa 13632
pmxA-D-DaB3-Polymyxin-B     CAGCACACGCTGGATGGAGCGGATCAGCTTTACAACATGCCGGAGCTGGTGCAGGTGGAA 13521
pmxA_B-LR_Polymyxin-E       CAGCACACGCTGGATGGAGCGGATCAGCTTTACAACATGCCGGAACTGGTGCAGGTGGAA 13761
pmxA_KP26_Polymyxin-E       CAGCACACGCTGGATGGAGCGGATCAGCTTTACAACATGCCGGAACTGGTGCAGGTGGAA 13761
                            ********************************************  **************

pmxA_Polymyxin-P            GGCGAGTTTGATTTAGACCGGTTGGAAGCCGCCTTGCGGAAATTGATAACACGGCATGAA 13857
pmxA_Polymyxin-A            GGCGAGTTTGATTTAGAACGGTTGGAAGCCGCCTTGCGGAAATTGATAACACGGCATGAA 13686
pmxA_1G_Polymyxin           ggcgagtttgatttagaccgcttggaagccgccttgcggaaattgataacacggcatgaa 13692
```

```
pmxA-D-DaB3-Polymyxin-B      GGCGAGTTTGATATAGACCGGTTGGAAGCGGCCTTGCGGAAATTGATAACACGGCATGAA 13581
pmxA_B-LR_Polymyxin-E        GGCGAGTTTGATTTAGACCGGTTGGAAGCCGCCTTGCGGAAATTGATAACACGGCATGAA 13821
pmxA_KP26_Polymyxin-E        GGCGAGTTTGATTTAGACCGGTTGGAAGCCGCCTTGCGGAAATTGATAACACGGCATGAA 13821
                             ************ **** ** ******** ******************************

pmxA_Polymyxin-P             TCGCTGCGCACCGGTTTTGAAATCGTGAAGGGCGAAGCGGTTCAGCGGATTTACCCGCAG 13917
pmxA_Polymyxin-A             TCGCTGCGCACCGGTTTTGAAATCGTGAAGGGCGAAGCGGTTCAGCGGATTTACCCGCAG 13746
pmxA_1G_Polymyxin            tcgctgcgcaccggttttgaaatcgtgaagggcgaagcggttcagcggatttacccgcag 13752
pmxA-D-DaB3-Polymyxin-B      TCGCTGCGCACCGGTTTTGAAATCGTGAAGGCCGAAGCGGTTCAGCGGATTTACCCGCAG 13641
pmxA_B-LR_Polymyxin-E        TCGCTGCGTACCGGTTTTGAACTCGTGAAGGGCAAAGCGGTTCAGCGGATTTACCCGCAG 13881
pmxA_KP26_Polymyxin-E        TCGCTGCGTACCGGTTTTGAACTCGTGAAGGGCAAAGCGGTTCAGCGGATTTACCCGCAG 13881
                             ******** ************ ********* * **************************

pmxA_Polymyxin-P             GTCGATTTTGCTATCGAGCATCATCAAGCGGATAAAGAGGATGCGGCTCAAATCGAGCAG 13977
pmxA_Polymyxin-A             GTCGATTTTGCTATCGAGCATTATCAAGCGGATAAAGAGGATGCGGCTCAAATCGAGCAG 13806
pmxA_1G_Polymyxin            gtcgattttactatcgagcatcatcaagcggataaagaggatgcggctcaaatcgagcag 13812
pmxA-D-DaB3-Polymyxin-B      GTCGATTTTGCTATCGAGCATCATCAAGCGGATAAAGAGGATGCGGCTCAAATCGAGCAG 13701
pmxA_B-LR_Polymyxin-E        GTCGATTTTGCTGTCGAGCATCATCAAGCGGATAAAGAGGATGCGGCTCAAATCGAGCAG 13941
pmxA_KP26_Polymyxin-E        GTCGATTTTGCTGTCGAGCATCATCAAGCGGATAAAGAGGATGCGGCTCAAATCGAGCAG 13941
                             ********* ** ******** **************************************

pmxA_Polymyxin-P             ATCGTCCGCAGCTTCGTTCGTCCATTTGATCTCGGCAAGCCGCCGCTGCTGCGCGCCGGG 14037
pmxA_Polymyxin-A             ATCGTCCGCAGCTTCGTTCGTCCGTTTGATCTCGGCAAGCCGCCGCTGCTGCGCGCCGGG 13866
pmxA_1G_Polymyxin            atcgtccgtagcttcgttcgtccgtttgatctcggcaagccgccgctgctgcgcgccggg 13872
pmxA-D-DaB3-Polymyxin-B      ATCGTCCGCAGCTTCGTTCGTCCGTTTGATCTCGGCAAGCCGCCGCTGCTGCGCGCCGGG 13761
pmxA_B-LR_Polymyxin-E        ATCGTCCGCAGCTTCGTTCGTCCATTTGATCTCGGCAAGCCGCCGCTGCTGCGCGCCGGG 14001
pmxA_KP26_Polymyxin-E        ATCGTCCGCAGCTTCGTTCGTCCATTTGATCTCGGCAAGCCGCCGCTGCTGCGCGCCGGG 14001
                             ******** ************** ************************************

pmxA_Polymyxin-P             GTCATCGAACTGGAGCCGAACCTGCATATTCTCCTTTTCGACATGCACCATATGGTGTCC 14097
pmxA_Polymyxin-A             GTCATCGAGCTGGAGCCGAACCTGCATATTCTCCTTTTCGACATGCACCATATGGTGTCC 13926
pmxA_1G_Polymyxin            gccatcgagctagagccgaacctgcatattctccttttcgacatgcaccatatggtgtcc 13932
pmxA-D-DaB3-Polymyxin-B      GTCATCGAGCTGGAGCCGAACCTGCATATTCTCCTTTTCGACATGCACCATATGGTGTCC 13821
pmxA_B-LR_Polymyxin-E        GTCATCGAGCTGGAGCCGAACCTGTATATTCTCATTTTCGACATGCACCATATGGTGTCC 14061
pmxA_KP26_Polymyxin-E        GTCATCGAGCTGGAGCCGAACCTGTATATTCTCATTTTCGACATGCACCATATGGTGTCC 14061
                             * ****** ** ************ ******** **************************

pmxA_Polymyxin-P             GACGGCGTATCAATGGCGATTGTGATCGATGAGTTCTCGAGTTTCTACGCCGGGGAAGAA 14157
pmxA_Polymyxin-A             GACGGCGTATCGATGGCGATTGTGATCGATGAGTTCTCGAGTTTCTACGCCGGGGAAGAA 13986
pmxA_1G_Polymyxin            gacggcgtatcgatggcgattgtgatcgatgagttctcgagtttctacgccgggggagaa 13992
pmxA-D-DaB3-Polymyxin-B      GACGGCGTATCGATGGCGATTGTGATCGATGAGTTCTCGAGTTTCTACGCCGGGGAAGAA 13881
pmxA_B-LR_Polymyxin-E        GACGGCGTATCAATGGCGATTGTGATCGATGAGTTCTCGAGTTTCTACGCCGGGGAAGAG 14121
pmxA_KP26_Polymyxin-E        GACGGCGTATCAATGGCGATTGTGATCGATGAGTTCTCGAGTTTCTACGCCGGGGAAGAG 14121
                             *********** ******************************************* ***
```

```
pmxA_Polymyxin-P            CTGCCGCCACTGCGCATTCAATACAAGGATTATGCCGTTTGGCAGCAGTCGAAGGCCCAC 14217
pmxA_Polymyxin-A            CTGCCGCCACTGCGCATTCAATACAAGGATTATGCCGTTTGGCAGCAGTCGAAGGCCCAC 14046
pmxA_1G_Polymyxin           ttgccgccactgcgcattcaatacaaggattatgtcgtttggcagcagtcaaaggcccac 14052
pmxA-D-DaB3-Polymyxin-B     CTGCCGCCACTGCGCATTCAATACAAGGATTATGCCGTTTGGCAGCAGTCGAAGGCCCAC 13941
pmxA_B-LR_Polymyxin-E       CTGCCGTCACTGCGCATTCAATACAAGGATTATGTCGTTTGGCAGCAGTCGAAGGCCTAC 14181
pmxA_KP26_Polymyxin-E       CTGCCGTCACTGCGCATTCAATACAAGGATTATGTCGTTTGGCAGCAGTCGAAGGCCTAC 14181
                             ***** *************************** *************** ****** **

pmxA_Polymyxin-P            CGAGAGCGGATCGGGCGGCAGGAAGCTTACTGGCTGCAAACCTTCGAAGGCGAGCTGCCG 14277
pmxA_Polymyxin-A            CGAGAGCGGATCGGGCGGCAGGAAGCGTACTGGCTGCAAACCTTCGAAGGCGAGCTGCCG 14106
pmxA_1G_Polymyxin           cgagagcggatcgggcggcaggaagcgtactggctgcaaaccttcgaaggcgagctgccg 14112
pmxA-D-DaB3-Polymyxin-B     CGAGAGCGGATCGGGCGGCAGGAAGTGTACTGGCTGCAAACCTTCGAAGGCGAGCTGCCG 14001
pmxA_B-LR_Polymyxin-E       CGAGAACGGATCGGGCGGCAGGAAGCGTACTGGCTGCAAACCTTCAAAGGCGAGCTGCCG 14241
pmxA_KP26_Polymyxin-E       CGAGAACGGATCGGGCGGCAGGAAGCGTACTGGCTGCAAACCTTCAAAGGCGAGCTGCCG 14241
                            ***** *******************  ****************** **************

pmxA_Polymyxin-P            ACGGCGGACCTGCCGATGGACTACGAACGGTCTGCGGCTCGCAGCTACGAAGGTGCGCAT 14337
pmxA_Polymyxin-A            ACGGCAGACCTGCCGATGGACTACGAACGGTCTGCGGCTCGCAGCTACGAAGGCGCGCAT 14166
pmxA_1G_Polymyxin           acggcggacctgccgatggactacgaacggtctgcggctcgcagctacgaaggcgcgcat 14172
pmxA-D-DaB3-Polymyxin-B     ACGGCGGACCTGCCGATGGACTACGAACGGTCTGCGGCTCGCAGCTACGAAGGCGCGCAT 14061
pmxA_B-LR_Polymyxin-E       ACGGCGAACCTGCCGATGGACTACAAACGGTCTGCAGCTCGCAGCTACGAAGGTGCACAT 14301
pmxA_KP26_Polymyxin-E       ACGGCGAACCTGCCGATGGACTACAAACGGTCTGCAGCTCGCAGCTACGAAGGTGCACAT 14301
                            *****  ***************** ********** ***************** ** ***

pmxA_Polymyxin-P            CTGGAGTTCGACGTCGAAGCTTCTCTCTCTACGCGGCTGCGCGAATTGGCGGCCGAGCGT 14397
pmxA_Polymyxin-A            CTGGAGTTCGACGTCGAAGCTTCTCTCTCTGCGCAGCTGCGCGAATTGGCGGCCGAGCGT 14226
pmxA_1G_Polymyxin           ctggagttcgacgtcgaagcttctctctctgcgcggctgcgcgaattggcagccgagcgt 14232
pmxA-D-DaB3-Polymyxin-B     CTGGAGTTCGACGTCGAAGCTTCGCTCTCTGCGCGGCTGCGCGAATTGGCGGCCGAGCGT 14121
pmxA_B-LR_Polymyxin-E       CTGGAGTTTGACGTCGAAGCCTCTCTCTCTATGCGGCTGCACGAATTGGCGGCAGAGCGT 14361
pmxA_KP26_Polymyxin-E       CTGGAGTTTGACGTCGAAGCCTCTCTCTCTATGCGGCTGCACGAATTGGCGGCAGAGCGT 14361
                            ******** *********** ** ******  ** ***** ********* ** ******

pmxA_Polymyxin-P            GAAAGCACGCTGTTCATGGTGCTGCTTGCTGCTTATACCGTGCTGCTGTCCAAGTACAGC 14457
pmxA_Polymyxin-A            GAAAGCACGCTGTTCATGGTGCTGCTTGCGGCTTATACCGTGCTGCTGTCCAAGTACAGC 14286
pmxA_1G_Polymyxin           gaaagcacgctgttcatggtgctgcttgcggcttataccgtgctgctgtccaagtacagc 14292
pmxA-D-DaB3-Polymyxin-B     GAAAGCACACTGTTCATGGTGCTGCTTGCGGCTTATACCGTGCTGCTGTCCAAGTACAGC 14181
pmxA_B-LR_Polymyxin-E       AAAAGCACGCTGTTCATGGTGCTGCTTGCGGCTTATACCGTGCTGCTGTCCAAATACAGC 14421
pmxA_KP26_Polymyxin-E       AAAAGCACGCTGTTCATGGTGCTGCTTGCGGCTTATACCGTGCTGCTGTCCAAATACAGC 14421
                             ******* ******************** *********************** ******

pmxA_Polymyxin-P            GGGCAGGAGGACTTGGTCGTGGGCACCCCGGTGGCGGGAAGAACGAACGCCGATTTGGAA 14517
pmxA_Polymyxin-A            GGGCAGGAGGACTTGGTCGTGGGCACCCCGGTGGCGGGAAGAACGAACGCCGATTTGGAA 14346
pmxA_1G_Polymyxin           gggcaggaggacttaatcgtgggcaccccggtggcgggaagaacgaacgccgatttggaa 14352
```

```
pmxA-D-DaB3-Polymyxin-B      GGGCAGGAGGACTTGGTCGTGGGCACCCCGGTGGCGGGAAGAACGAACGCCGATTTGGAA 14241
pmxA_B-LR_Polymyxin-E        GGGCAGGAGGACTTGATCGTGGGCACCCCGGTGGCGGGAAGAACGAACGCCGATTTGGAA 14481
pmxA_KP26_Polymyxin-E        GGGCAGGAGGACTTGATCGTGGGCACCCCGGTGGCGGGAAGAACGAACGCCGATTTGGAA 14481
                             ************** *********************************************

pmxA_Polymyxin-P             CCGGTCATCGGGATGTTTGTCAATACGCTGGCGATCCGCAATCGCCCGTCGGGCGACAAA 14577
pmxA_Polymyxin-A             CCGATCATCGGGATGTTTGTCAATACGCTGGCGATCCGCAATCGTCCGTCGGGCGACAAA 14406
pmxA_1G_Polymyxin            gcggtcatcgggatgtttgtcaatacgctggcgatccgcaatcgtccgtcgggcgacaaa 14412
pmxA-D-DaB3-Polymyxin-B      CCGGTCATAGGGATGTTTGTCAATACGCTGGCGATCCGCAATCGTCCGTCGGGCGACAAA 14301
pmxA_B-LR_Polymyxin-E        CCGGTCATCGGAATGTTTGTCAATACACTGGCGATCCGCAATCGTCCGTCGGGCAACAAA 14541
pmxA_KP26_Polymyxin-E        CCGGTCATCGGAATGTTTGTCAATACACTGGCGATCCGCAATCGTCCGTCGGGCAACAAA 14541
                              ** **** ** ************** ***************** ********* *****

pmxA_Polymyxin-P             ACGTTCTTGTCCTACCTGGAAGAAGTGAAGGAAACGGCTTTGGGTGCTTTCGAGAACCAG 14637
pmxA_Polymyxin-A             ACGTTCTTGTCCTACCTGGAAGAAGTGAAGGAAACGGCTTTGGGTGCTTTCGAGAACCAG 14466
pmxA_1G_Polymyxin            acgttcttgtcctacctggaagaagtgaaggaaacggctttgggtgcttttgagaaccag 14472
pmxA-D-DaB3-Polymyxin-B      ACGTTCTTGTCCTACCTGGAAGAAGTGAAGGAAACGGCTTTGGGTGCTTTCGAGAACCAA 14361
pmxA_B-LR_Polymyxin-E        ACGTTTTTGTCCTACCTGGAAGAAGTGAAGGAAACGGCTTTGGGTGCTTTCGAGAACCAG 14601
pmxA_KP26_Polymyxin-E        ACGTTTTTGTCCTACCTGGAAGAAGTGAAGGAAACGGCTTTGGGTGCTTTCGAGAACCAG 14601
                             ***** ******************************************** ********

pmxA_Polymyxin-P             GATTATCCATTCGAGGAGCTGGTGGAGCGTTTGAATGTGAAGCGGGAGCCGGGCCGCTTC 14697
pmxA_Polymyxin-A             GATTATCCATTCGAGGAGCTTGTGGAGCGTTTGAATGTGAAGCGGGAACCGGGCCGCTTC 14526
pmxA_1G_Polymyxin            gattatccattcgacgagcttgtggagcgcttgaatgtgaagcgggagccgggccgcttc 14532
pmxA-D-DaB3-Polymyxin-B      GATTATCCATTCGAGGAGCTCGTGGAGCGTTTGAATGTGAAGCGGGAGCCGGGCCGCTTC 14421
pmxA_B-LR_Polymyxin-E        GATTATCCATTTGAGGAGCTCGTGGAACGTTTGAATGTGAAGCGGGAGCCGGGCCGCTTC 14661
pmxA_KP26_Polymyxin-E        GATTATCCATTTGAGGAGCTCGTGGAACGTTTGAATGTGAAGCGGGAGCCGGGCCGCTTC 14661
                             *********** ** ***** ***** ** ***************** ************

pmxA_Polymyxin-P             CCGCTGTTCGATGCCGTTTTCGACTTGCAAAATATCGAAGAACGAGACGCCGAGCTGGAA 14757
pmxA_Polymyxin-A             CCGCTGTTCGATGCCGTTTTCGACTTGCAAAATATCGAAGAACGAGACGCCGAGCTGGAA 14586
pmxA_1G_Polymyxin            ccgctgttcgatgccgttttcgacttgcaaaatatcgaagaacgagacgccgagctggaa 14592
pmxA-D-DaB3-Polymyxin-B      CCGCTGTTCGATGCCGTTTTCGACTTGCAAAATATCGAAGAACGAGACGCCGAGCTGGAA 14481
pmxA_B-LR_Polymyxin-E        CCGCTGTTCGATGCCGTTTTCGACTTGCAAAATATCGAAGAACGAGACGTCGAGCTGGAA 14721
pmxA_KP26_Polymyxin-E        CCGCTGTTCGATGCCGTTTTCGACTTGCAAAATATCGAAGAACGAGACGTCGAGCTGGAA 14721
                             ************************************************* **********

pmxA_Polymyxin-P             GGGGTCAGCCTGAAGACTTACGAGCTTGACCATTTGGAAGAAGCGAAGTTCGATCTGACG 14817
pmxA_Polymyxin-A             GGGGTCAGCCTGAAGACTTACGAGCTTGACCATTTGGAAGAAGCGAAGTTCGATCTGACG 14646
pmxA_1G_Polymyxin            ggggtcagcctgaagacttacgagcttgaccatttggaagaagcgaagttcgatctgacg 14652
pmxA-D-DaB3-Polymyxin-B      GGGGTCAGCCTGAAGACTTACGAGCTTGACCATTTGGAAGAAGCGAAGTTCGATCTGACG 14541
pmxA_B-LR_Polymyxin-E        GGGGTCAGCCTGAAGAATTACGAGCTTGACCATTTGGAAGAAGCGAAGTTCGATCTGACG 14781
pmxA_KP26_Polymyxin-E        GGGGTCAGCCTGAAGAATTACGAGCTTGACCATTTGGAAGAAGCGAAGTTCGATCTGACG 14781
                             **************** *******************************************
```

```
pmxA_Polymyxin-P            CTGTTTATGTATGAAAACAACGGGGCGCTGAGCGGGGGCTTCTTCTACGCCACCAAGCTG 14877
pmxA_Polymyxin-A            CTGTTTATGTATGAAAACAATGGGGCGCTGAGCGGGGGCTTCTTCTACGCCACCAAGCTG 14706
pmxA_1G_Polymyxin           ctgtttatgtatgaaaacaacggggcgctgagcgggggcttcttctacgccaccaagctg 14712
pmxA-D-DaB3-Polymyxin-B     CTGTTTATGTATGAAAACAACGGGGCGCTGAGCGGGGGCTTCTTCTACGCCACCAAGCTG 14601
pmxA_B-LR_Polymyxin-E       CTGTTTATGTATGAAAACAACGGGGCGCTGAGCGGGGGCTTCTTCTACGCCACCAAGCTG 14841
pmxA_KP26_Polymyxin-E       CTGTTTATGTATGAAAACAACGGGGCGCTGAGCGGGGGCTTCTTCTACGCCACCAAGCTG 14841
                            ******************** ***************************************

pmxA_Polymyxin-P            TTCAAAAAAGCGATGATCCGCACCTTGACCGAGGATTACCTGCGGGTACTGTCTCAAATT 14937
pmxA_Polymyxin-A            TTCAAAGAAGCGATGATCCGCACCTTGACCGAGGATTACCTGCGGGTACTGTCTCAAATT 14766
pmxA_1G_Polymyxin           ttcaaagaagcgatgatccgcaccttgaccgaggattacctgcgggtactgtctcaaatt 14772
pmxA-D-DaB3-Polymyxin-B     TTCAAAGAAGCGATGATCCGCACCTTGACCGAGGATTACCTGCGGGTACTGTCTCAAATT 14661
pmxA_B-LR_Polymyxin-E       TTCAAAGAAGCCATGATCCGCACCTTGACCGAGGATTACCTGAGGGTACTGTCTCAAATT 14901
pmxA_KP26_Polymyxin-E       TTCAAAGAAGCCATGATCCGCACCTTGACCGAGGATTACCTGAGGGTACTGTCTCAAATT 14901
                            ****** **** ****************************** *****************

pmxA_Polymyxin-P            GCGGAAAATCCACAACTCGAGCTAAGCCGGATTGAATGTCATAAACCGGCGGCAGGCGCA 14997
pmxA_Polymyxin-A            GCGAAAAATCCACAACTCGAGCTAAGCCGGATAGAATGTCATAAACCGGCGGCAGGCGCA 14826
pmxA_1G_Polymyxin           gcggaaaatccacaacttgagctaagccggattgaatgtcataaaccggcggcaggcgca 14832
pmxA-D-DaB3-Polymyxin-B     GCGGAAAATCCACAACTCGAGCTAAGCCGGATTGAATGTCATAAACCGACGGCAGGCGCA 14721
pmxA_B-LR_Polymyxin-E       GCGGAAAATCCGCAACTTGAGCTAAGTCGGATTGAATGTCATAAACCGGCGGCTGGCGCA 14961
pmxA_KP26_Polymyxin-E       GCGGAAAATCCGCAACTTGAGCTAAGTCGGATTGAATGTCATAAACCGGCGGCTGGCGCA 14961
                            *** ******* ***** ******** ***** *************** **** ******

pmxA_Polymyxin-P            AAAAGTGCCGTCGATACGATCGAATTCGCGTTCTAA     15033
pmxA_Polymyxin-A            AAGAGTGCCGTCGATACGATCGAATTCGCGTTCTAA     14862
pmxA_1G_Polymyxin           aagagtgccgtcgatacgatcgaattcgcgttctaa     14868
pmxA-D-DaB3-Polymyxin-B     AAGAGTGCCGTCGATACGATCGAATTCGCGTTCTAA     14757
pmxA_B-LR_Polymyxin-E       AAGAGTGCCGTCGATACGATCGAATTTGCGTTCTAA     14997
pmxA_KP26_Polymyxin-E       AAGAGTGCCGTCGATACGATCGAATTTGCGTTCTAA     14997
                            ** *********************** *********
```

CLUSTAL O(1.2.4) multiple sequence alignment of pmxB

```
pmxB_B-LR_Polymyxin-E       ATGAAATCTTTATTTGAAAAGGAAGAAAGGTACTGGAGCGGCAAGTTTGACGCCGATGAC 60
pmxB_KP26_Polymyxin-E       ATGAAATCTTTATTTGAAAAGGAAGAAAGGTACTGGAGCGGCAAGTTTGACGCCGATGAC 60
pmxB_Polymyxin-A            ATGAAATCTTTGTTTGAAAAGGAAGAACAGTACTGGAGCGGTAAGTTTGACGCCGATGAC 60
pmxB_1G_Polymyxin           atgaaatctttgtttgaaaaggaagaacggtactggagcggcaagtttgatgccgatgac 60
pmxB-D-DaB3-Polymyxin-B     ATGAAATCTTTGTTTGAAAAGGAAGAACAGTACTGGAGCGGCAAGTTTGACGCCGATGAC 60
pmxB_Polymyxin-P            ATGAAATCTTTGTTTGAAAAGGAAGAACGATACTGGAGCGGCAAGTTTGACGCCGATGAC 60
                            *********** ***************   *********** ******** *********
```

```
pmxB_B-LR_Polymyxin-E        AACCTGAGCTTCCTTCCCTACAGTCAATCCTCCAAATTATCCGCCGACGGGGAAGCTGCG 120
pmxB_KP26_Polymyxin-E        AACCTGAGCTTCCTTCCCTACAGTCAATCCTCCAAATTATCCGCCGACGGGGAAGCTGCG 120
pmxB_Polymyxin-A             AGCCTGAGCTTCCTTCCCTACAGTCAATCCTCCAAATTATCCGCCCACGGGGAAGCTGCG 120
pmxB_1G_Polymyxin            agcctgagcttccttccctacagtcaatcctccaaattatccgccgacggggaagctgcg 120
pmxB-D-DaB3-Polymyxin-B      AGCCTGAGCTTCCTTCCCTACAGTCAATCCTCCAAATTATCCGCCGACGGGGAAGCTGCG 120
pmxB_Polymyxin-P             AGCCTGAGCTTCCTTCCCTACAGTCAATCCTCCAAATTATCCGCCGACGGGGAAGCTGCG 120
                             * ******************************************* **************

pmxB_B-LR_Polymyxin-E        GCCGAGCCGGGCTTGCTTCACCGTACCCTGCCGAGCGAACTCTCGGAGAGAATCATTCGC 180
pmxB_KP26_Polymyxin-E        GCCGAGCCGGGCTTGCTTCACCGTACCCTGCCGAGCGAACTCTCGGAGAGAATCATTCGC 180
pmxB_Polymyxin-A             GCCGAGCCGGGCTTGCTTCACCGTACTCTGCCGAGTCAACTCTCGGAGAGAATCATTAGC 180
pmxB_1G_Polymyxin            gccgagccgggcttgcttcaccgtactctgccgagcgaactctcagagagaatcatttgc 180
pmxB-D-DaB3-Polymyxin-B      GCCGAGCCGGGCTTGCTTCACCGTACTCTGCCGAGCGAACTCTCGGAGAGAATCATTTGC 180
pmxB_Polymyxin-P             GCCGAGCCGGGCTTGCTTCACCGTACTCTGCCGAGTGAACTCTCGGAGAGAATCATTTGC 180
                             ************************** ********  ******* ************ **

pmxB_B-LR_Polymyxin-E        CTCGCCAACGGTTCGGATTTGGCTATGTACATGATTGTTTTGGCAGGAGTAAAAAGCCTG 240
pmxB_KP26_Polymyxin-E        CTCGCCAACGGTTCGGATTTGGCTATGTACATGATTGTTTTGGCAGGAGTAAAAAGCCTG 240
pmxB_Polymyxin-A             CTCGCCAACGGTTCGGATTTGGCTTTGTACATGATTGTTTTGGCAGGAGTAAAAAGCCTG 240
pmxB_1G_Polymyxin            ctcgccaacggttcggatttggctttgtacatgattgttttggcaggagtaaaaagcctg 240
pmxB-D-DaB3-Polymyxin-B      CTCGCCAACGGTTCGGATTTGGCTTTGTACATGATTGTTTTGGCAGGAGTAAAAAGCCTG 240
pmxB_Polymyxin-P             CTCGCCAACGGTTCGGATTTGGCTTTGTACATGATTGTTTTGGCAGGAGTAAAAAGCCTG 240
                             ************************ ***********************************

pmxB_B-LR_Polymyxin-E        CTGTTCAAATATACCGGGCAGGACCAAGTGCTGGTCGGCATGCCTTCTTATAGCGCAGAC 300
pmxB_KP26_Polymyxin-E        CTGTTCAAATATACCGGGCAGGACCAAGTGCTGGTCGGCATGCCTTCTTATAGCGCAGAC 300
pmxB_Polymyxin-A             CTGTTCAAATATACCGGGCGGGACCAAGTGCTGGTCGGCATGCCTTCTTATAGCGCAGAC 300
pmxB_1G_Polymyxin            ctgttcagatataccgggcgggaccaagtgctggtgggcatgccttcttatagcgcagac 300
pmxB-D-DaB3-Polymyxin-B      CTGTTCAAGTATACCGGGCGGGACCAAGTGCTGGTCGGCATGCCTTCTTATAGCGCAGAC 300
pmxB_Polymyxin-P             CTGTTCAAATATACCGGGCGGGACCAAGTGCTGATCGGTATGCCTTCTTATAGCGCAGAC 300
                             *******  ********** ************* * ** *********************

pmxB_B-LR_Polymyxin-E        CCCGACGGGACTCCGCCGCCGCATGACATCTTGGTGATCAAGACGGCCGTAAGCCATCAG 360
pmxB_KP26_Polymyxin-E        CCCGACGGGACTCCGCCGCCGCATGACATCTTGGTGATCAAGACGGCCGTAAGCCATCAG 360
pmxB_Polymyxin-A             CCCGACGGGACTCCGCCGCCGCATGACATCTTGGTGATCAAGACGTCCGTAAGCCGCCAG 360
pmxB_1G_Polymyxin            cctgacgggactccgccgccgcaggacattttggtaatcaagacgtccgtaagccaccag 360
pmxB-D-DaB3-Polymyxin-B      CCCGACGGGACTCCGCCGCCGCATGACATCTTGGTGATCAAGACGTCCGTAAGCCGCCAG 360
pmxB_Polymyxin-P             CCCGACGGGACTCCGCCGCCGCATGACATCTTGGTGATCAAGACGTCCGTAAGCCGCCAG 360
                             ** ******************** ***** ***** ********* *********  ***

pmxB_B-LR_Polymyxin-E        ACTACGCTGAAAACGCTGCTCGGGGGCATCAAAGCCTCCATTGGCGAGGCGCTGGAGCAT 420
pmxB_KP26_Polymyxin-E        ACTACGCTGAAAACGCTGCTCGGGGGCATCAAAGCCTCCATTGGCGAGGCGCTGGAGCAT 420
pmxB_Polymyxin-A             ACTACGCTGAAAACGCTGCTCGGGGGCATCAAAGCCTCCATCGGCGAGGCGCTGGAGCAT 420
```

```
pmxB_1G_Polymyxin            actacgctgaaaacgctgctcgggggcatcaaagcctccatcggcgaggcgctggagcat 420

pmxB-D-DaB3-Polymyxin-B      ACTACGCTGAAAACGCTGCTCGGGGGCATCAAAGCCTCCATCGGCGAGGCGCTGGAGCAT 420

pmxB_Polymyxin-P             ACTACGCTGAAAACGCTGCTCGGGGGCATCAAAGCCTCCATCGGCGAGGCGCTGGAGCAT 420

                             ***************************************** ******************


pmxB_B-LR_Polymyxin-E        CAGCACCTGCCTTTTCAGAAAATGGTGGAGCCGCTCCATCTGGACTATACGGGGGATGGC 480

pmxB_KP26_Polymyxin-E        CAGCACCTGCCTTTTCAGAAAATGGTGGAGCCGCTCCATCTGGACTATACGGGGGATGGC 480

pmxB_Polymyxin-A             CAGCACCTGCCTTTTCGGAAAATGGTGGAGCCACTCCATCTGGACTATACGGGGGACGGC 480

pmxB_1G_Polymyxin            cagcacctgccttttcggaaaatggtggagccgctccatctggactatacgggggacggc 480

pmxB-D-DaB3-Polymyxin-B      CAGCACCTGCCTTTTCGGAAAATGGTGGAGCCGCTCCATCTGGACTATACGGGGGACGGC 480

pmxB_Polymyxin-P             CAGCACCTGCCTTTTCGGAAAATGGTGGAGCCGCTCCATCTGGACTATACGGGGGACGGC 480

                             **************** *************** *********************** ***


pmxB_B-LR_Polymyxin-E        CTCCCGGTCGTTAACACCGTTGCATCCTTCGCCCCGATTCATCCCGAACCGCTGGGTAAC 540

pmxB_KP26_Polymyxin-E        CTCCCGGTCGTTAACACCGTTGCATCCTTCGCCCCGATTCATCCCGAACCGCTGGGTAAC 540

pmxB_Polymyxin-A             CTCCCGGTCGTTAACACCGTCGTATCCTTCGCCCCGATTCATCCGGAACCGCAAGGTAAT 540

pmxB_1G_Polymyxin            ctaccaatcatcaacaccgtcgcatcctttgcaccgattcatccggaaccgctgggcaac 540

pmxB-D-DaB3-Polymyxin-B      CTCCCGGTCGTCAACACCGTCGTATCCTTCGCTCCGATTCATCCGGAACCGCTGGGTAAC 540

pmxB_Polymyxin-P             CTCCCGGTCGTCAACACCGTCGCATCCTTCGCCTCGATTCATCCGGAACCGCTGGTTAAC 540

                             ** **  ** * ******** * ****** **  ********** *******  *  ** 


pmxB_B-LR_Polymyxin-E        CGGGTGGCGGCCGATACGGTTTTTCGCTTCGATCGCCAAAACCACTCCATCGAGCTGGAA 600

pmxB_KP26_Polymyxin-E        CGGGTGGCGGCCGATACGGTTTTTCGCTTCGATCGCCAAAACCACTCCATCGAGCTGGAA 600

pmxB_Polymyxin-A             CGGGTGGCGGCCGATACGGTTTTTCGCTTCGACCGCCAAAACCACTCCATCGAGCTGGAA 600

pmxB_1G_Polymyxin            cgggtggcggccgatacggtttttcgcttcgaccgccaaaaccattccatcgagctggaa 600

pmxB-D-DaB3-Polymyxin-B      CGGGTGGCGGCCGATACGTTTTTTCGCTTCGACCGCCAAAACCACTCCATCGAGCTGGAA 600

pmxB_Polymyxin-P             CGGGTGGCGGCCGATACGGTTTTTCGCTTCGACCGCCAAAACCACTCCATCGAGCTGGAA 600

                             ****************** ************* *********** ***************


pmxB_B-LR_Polymyxin-E        ATAAGCTTTGACGGGCAGCGGTACGAGCGGGCATTTGTGGAACAGGCGGCCGACCATTTT 660

pmxB_KP26_Polymyxin-E        ATAAGCTTTGACGGGCAGCGGTACGAGCGGGCATTTGTGGAACAGGCGGCCGACCATTTT 660

pmxB_Polymyxin-A             ATAAGCTTTGACGGGCAGCGGTACGAGCGGGCATTTGTGGAACAGGCGGCCGACCATCTT 660

pmxB_1G_Polymyxin            ataagctttgacgggcagtggtacgagcgggcatttgtggaacaggcggccgaccatctt 660

pmxB-D-DaB3-Polymyxin-B      ATAAGCTTTGACGGGCAGCGGTACGAGCGCGCATTTGTGGAACAGGCGGCCGACCATCTT 660

pmxB_Polymyxin-P             ATAAGCTTTGATGGGCAGCGGTACGAGCGGGCATTTGTGGAACAGGCGGCCGACCATCTT 660

                             *********** ****** ********** *************************** **


pmxB_B-LR_Polymyxin-E        GTTCGGCTACTGTCCTTGCTTTTATTTCAGCCTGATCTGGAGCTTGGACAAGCCGATGTG 720

pmxB_KP26_Polymyxin-E        GTTCGGCTACTGTCCTTGCTTTTATTTCAGCCTGATCTGGAGCTTGGACAAGCCGATGTG 720

pmxB_Polymyxin-A             GTTCGGCTGCTGTCCGTGCTTTTATTTCAGCCGGATCTGAAGCTGGGACAAGCCGATGTG 720

pmxB_1G_Polymyxin            gttcggctgctgtccgtgcttttatttcagccggatctggagctcggacaagccgatgtg 720

pmxB-D-DaB3-Polymyxin-B      GTTAGGCTGCTGTCCGTGCTTTTATTTCAGCCGGATCTGGAGCTCGGACAAGCCGATGTG 720

pmxB_Polymyxin-P             GTTCGGCTGCTGTCCGTGCTTTTATTTCAGCCGGATCTGGAGCTCGGACAAGCCGATGTG 720

                             *** **** ****** **************** ****** **** ***************
```

```
pmxB_B-LR_Polymyxin-E        CTGTCCCCAGACGAGAGGGAGACGCTGCTGAAGCGATTTAATGACACCGAAACCGGGTTC 780
pmxB_KP26_Polymyxin-E        CTGTCCCCAGACGAGAGGGAGACGCTGCTGAAGCGATTTAATGACACCGAAACCGGGTTC 780
pmxB_Polymyxin-A             CTGTCACCAGACGAGAGGGAGACGCTGCTGAAACGATTTAATGACACCGAAACCGAGTTC 780
pmxB_1G_Polymyxin            ctgtccccagacgagagggagacgctgctgaagcgatttaatgacaccgaaaccgggttc 780
pmxB-D-DaB3-Polymyxin-B      CTGTCCCCAGACGAGAGGGAGACGCTGCTGAAGCGATTTAATGACACCGAAAGCGGGTTC 780
pmxB_Polymyxin-P             CTGTCCCCAGACGAGAGGGAGACGCTGCTGAAGCGATTTAATGACACCGAAACCGAGTTC 780
                             ***** ************************** ******************* ** ****

pmxB_B-LR_Polymyxin-E        GAGCGGGGGAAAACGATTCATGGCTTGTTCGAAGAGCAGGCGGAGCTTTACCCGGACAAC 840
pmxB_KP26_Polymyxin-E        GAGCGGGGGAAAACGATTCATGGCTTGTTCGAAGAGCAGGCGGAGCTTTACCCGGACAAC 840
pmxB_Polymyxin-A             GAGCGGGGGAAAACGATTTACGGCTTGTTCGAAGAGCAGGCGGAGCTTTACCCGGACAAC 840
pmxB_1G_Polymyxin            gagcgggggaaaacgatttacggcttgttcgaagaacaggcggagctttacccggacaac 840
pmxB-D-DaB3-Polymyxin-B      GAGCGGGGGAAAACGATTTACGGCTTGTTCGAAGAACAGGCGGAGCTTTACCCGGACAAC 840
pmxB_Polymyxin-P             GAGCGGGGGAAAACGATTTACGGCTTGTTCGAAGAACAGGCGGAGCTTTACCCGGACAAC 840
                             ****************** * ************** ************************

pmxB_B-LR_Polymyxin-E        GTGGCCGCCGTCATGAATGAGCGGCAGCTGACTTACCGCGAACTGAACGAGCGATCCAAC 900
pmxB_KP26_Polymyxin-E        GTGGCCGCCGTCATGAATGAGCGGCAGCTGACTTACCGCGAACTGAACGAGCGATCCAAC 900
pmxB_Polymyxin-A             GTGGCCGCCGTCATGAACGAGCGGCAGCTGACCTACCGCGAGCTGAACGAGCGATCCAAC 900
pmxB_1G_Polymyxin            gtggccgccgtcatgaacgaggggcagttgacctaccgcgagctgaacgagcgatccaac 900
pmxB-D-DaB3-Polymyxin-B      GTGGCCGCCGTCATGAACGAGCGGCAGCTGACCTACCGCGAGCTGAACGAGCGATCCAAC 900
pmxB_Polymyxin-P             GTGGCCGCCGTCATGAACGAGCGGCAGCTGACCTACCGCGAGCTGAACGAGCGATCCAAC 900
                             ***************** *** ***** **** ******** ******************

pmxB_B-LR_Polymyxin-E        CGCCTTGCGCGGAAGCTGCGGGAGACGGGAGTAGAAGCGGATCAGCTGGTAGCGATTCTG 960
pmxB_KP26_Polymyxin-E        CGCCTTGCGCGGAAGCTGCGGGAGACGGGAGTAGAAGCGGATCAGCTGGTAGCGATTCTG 960
pmxB_Polymyxin-A             CGCCTTGCGCGGAAGCTGCGTGAGGCGGGAGTAGAAGCGGACCAGCTGGTAGCGATTCTG 960
pmxB_1G_Polymyxin            cgccttgcgcggaagctgcgggagacgggagtagaagcggaccagctggtagcgattctg 960
pmxB-D-DaB3-Polymyxin-B      CGCCTTGCGCGGAAGCTGCGGAAGACGGGAGTAGAAGCGGACCAGCTGGTAGCGATTCTG 960
pmxB_Polymyxin-P             CGCCTTGCGCGGAAGCTGCGGGAGACGGGAGTAGAAGCGGACCAGTTGGTAGCGATTCTG 960
                             ********************  ** **************** *** **************

pmxB_B-LR_Polymyxin-E        GCTGAACGCTCGCTCGATATGGTCGTCGGCATTCTGGCGATTCTTAAAGCGGGCGGAGCC 1020
pmxB_KP26_Polymyxin-E        GCTGAACGCTCGCTCGATATGGTCGTCGGCATTCTGGCGATTCTTAAAGCGGGCGGAGCC 1020
pmxB_Polymyxin-A             GCCGAACGCTCGCTCGATATGGTCGTCGGCATTCTGGCGATTCTCAAAGCGGGCGGAGCA 1020
pmxB_1G_Polymyxin            gccgaacgctcgctcgatatggttgtgggcattctggcgattctcaaagcgggcggagcc 1020
pmxB-D-DaB3-Polymyxin-B      GCCGAACGCTCGCTCGATATGGTTGTCGGCATTCTGGCGATTCTCAAAGCGGGCGGAGCA 1020
pmxB_Polymyxin-P             GCCGAACGCTCGCTCGATATGGTTGTCGGCATTCTGGCGATTCTCAAAGCGGGCGGAGCA 1020
                             ** ******************** ** ***************** **************

pmxB_B-LR_Polymyxin-E        TACGTGCCTGTTGATCCCGACTACCCGGAGGAGCGCATCCGCTTCATGATCGAGGATTCG 1080
pmxB_KP26_Polymyxin-E        TACGTGCCTGTTGATCCCGACTACCCGGAGGAGCGCATCCGCTTCATGATCGAGGATTCG 1080
pmxB_Polymyxin-A             TACGTGCCTGTCGATCCCGACTACCCGGAGGAGCGTATCCGCTTCATGATCGAGGATTCG 1080
```

```
pmxB_1G_Polymyxin            tacgtgcctgtcgatcccgactacccggaggagcgcatccgcttcatgatcgaggattcg 1080
pmxB-D-DaB3-Polymyxin-B      TACGTGCCTGTCGATCCCGACTACCCGGAGGAGCGCATCCGCTTCATGATCGAGGATTCG 1080
pmxB_Polymyxin-P             TACGTGCCTGTCGATCCCGACTACCCGGAGGAGCGCATCCGCTTCATGATCGAGGATTCG 1080
                             *********** *********************** ************************

pmxB_B-LR_Polymyxin-E        GGCGCGCCGTTATTGTTGATTCAAAAGCATCTGCACGAGAAGACCGACTTCGCAGGAACG 1140
pmxB_KP26_Polymyxin-E        GGCGCGCCGTTATTGTTGATTCAAAAGCATCTGCACGAGAAGACCGACTTCGCAGGAACG 1140
pmxB_Polymyxin-A             GGCGCGCCGTTATTGCTGATTCAAAAGCATCTGCACGAGAAGACCGACTTCGCAGGAACG 1140
pmxB_1G_Polymyxin            ggcgcgccgttattgctgattcaaaagcatctgcacgagaagaccgacttcgcaggaacg 1140
pmxB-D-DaB3-Polymyxin-B      GGCGCGCCGTTATTGCTGATTCAAAAGCATCTGCACGAGAAGACCGACTTCGCAGGAACG 1140
pmxB_Polymyxin-P             GGCGCGCCGTTATTGCTGATTCAAAAGCATCTGCACGAGAAGACCAACTTCGCAGGAACG 1140
                             *************** ***************************** **************

pmxB_B-LR_Polymyxin-E        CGCCTCGAGTTGGATGATTTCGTGTGGAACGACAGAGGGGTGGACTCCGAAGGTAGGCTG 1200
pmxB_KP26_Polymyxin-E        CGCCTCGAGTTGGATGATTTCGTGTGGAACGACAGAGGGGTGGACTCCGAAGGTAGGCTG 1200
pmxB_Polymyxin-A             CGCCTCGAATTGGACGATTTCGTTTGGGGCGACAGAGGGGCGAACTCCGCCGAAGCGCTG 1200
pmxB_1G_Polymyxin            cgcctcgaattggacgatttcgtttggggcgacagaggggcggacgccgccgatgcgctg 1200
pmxB-D-DaB3-Polymyxin-B      CGCCTCGAATTGGACGATTTCGTTTGGGGCGACAGAGGGGCGGACTCCGCCGATGCGCTG 1200
pmxB_Polymyxin-P             CGCCTCGAATTGGACGATTTCGTTTGGGGCGACAGAGGGGCGGACTCCGCCGATGCGCTG 1200
                             ******** ***** ******** ***  *********** * ** ***  *    ****

pmxB_B-LR_Polymyxin-E        GATGCTTCGAACCTGGAGCCGATTTCCGGGCCGGGCAATCTGGCTTATGTTATCTACACG 1260
pmxB_KP26_Polymyxin-E        GATGCTTCGAACCTGGAGCCGATTTCCGGGCCGGGCAATCTGGCTTATGTTATCTACACG 1260
pmxB_Polymyxin-A             GACGCTTCGAACCTGGAGCCGATTTCCGGGCCGGGCAACCTGGCATATGTCATCTACACG 1260
pmxB_1G_Polymyxin            gacgcttcgaacctggagccgatttccgggccgggcaacctggcatatgtcatctacacg 1260
pmxB-D-DaB3-Polymyxin-B      GACGCTTCGAACCTGGAGCCGATTTCCGGGCCGGGCAACCTGGCTTATGTCATCTACACG 1260
pmxB_Polymyxin-P             GACGCTTCGAACCTGGAGCCGATTTCCGGGCCGGGCAACCTGGCATATGTCATCTACACG 1260
                             ** *********************************** ***** ***** *********

pmxB_B-LR_Polymyxin-E        TCGGGAACGACCGGCAGACCGAAAGGAACATTAATCGAGCATAAGAACGTCGTGCGGCTC 1320
pmxB_KP26_Polymyxin-E        TCGGGAACGACCGGCAGACCGAAAGGAACATTAATCGAGCATAAGAACGTCGTGCGGCTC 1320
pmxB_Polymyxin-A             TCGGGAACGACCGGCAGACCGAAAGGAACGCTGATCGAGCATAAGAACGTCGTGCGGCTC 1320
pmxB_1G_Polymyxin            tcgggaacgaccggcagaccgaaaggaacgctgatcgagcataagaacgtcgtgcgcctc 1320
pmxB-D-DaB3-Polymyxin-B      TCGGGAACGACCGGCAGACCGAAAGGAACGCTGATCGAGCATAAGAACGTCGTGCGCCTC 1320
pmxB_Polymyxin-P             TCGGGAACGACCGGCAGACCGAAAGGAACACTGATCGAGCATAAGAACGTCGTGCGGCTC 1320
                             *****************************  * *********************** ***

pmxB_B-LR_Polymyxin-E        CTGTTCAACGACAAGAACCTGTTCGACTTCGGGCCATCCGACACGTGGACGCTGTTCCAC 1380
pmxB_KP26_Polymyxin-E        CTGTTCAACGACAAGAACCTGTTCGACTTCGGGCCATCCGACACGTGGACGCTGTTCCAC 1380
pmxB_Polymyxin-A             CTGTTCAACGACAAGAATCTGTTTGACTTCGGGCCGTCCGACACGTGGACGCTGTTCCAC 1380
pmxB_1G_Polymyxin            ctgttcaacgacaagaacctgttcgacttcgggccgtccgacacgtggacgctgttccac 1380
pmxB-D-DaB3-Polymyxin-B      CTGTTCAACGACAAGAACCTGTTCGACTTCGGGCCGTCCGACACGTGGACGCTGTTCCAC 1380
pmxB_Polymyxin-P             CTGTTCAACGACAAGAACCTGTTCGACTTCGGGCCGTCCGACACGTGGACGCTGTTCCAC 1380
                             ***************** ***** *********** ************************
```

```
pmxB_B-LR_Polymyxin-E        TCGTTCTGCTTCGATTTCTCCGTCTGGGAAATGTACGGAGCGCTGCTGTACGGAGGCAAG 1440
pmxB_KP26_Polymyxin-E        TCGTTCTGCTTCGATTTCTCCGTCTGGGAAATGTACGGAGCGCTGCTGTACGGAGGCAAG 1440
pmxB_Polymyxin-A             TCGTTCTGCTTCGATTTCTCCGTCTGGGAAATGTACGGAGCGCTGCTGTACGGAGGCAAG 1440
pmxB_1G_Polymyxin            tcgttctgcttcgatttctccgtctgggaaatgtacggagcgctgctgaacggaggcaag 1440
pmxB-D-DaB3-Polymyxin-B      TCGTTCTGCTTCGATTTCTCCGTCTGGGAAATGTACGGAGCGCTGCTGTACGGAGGCAAG 1440
pmxB_Polymyxin-P             TCGTTCTGCTTCGATTTCTCCGTCTGGGAAATGTACGGAGCGCTGCTGAACGGAGGCAAG 1440
                             ************************************************ ***********

pmxB_B-LR_Polymyxin-E        CTGGTCATCGTACCGCCGCTCACGGCGAAAAATCCGGCTGATTTCCTGGTGCTGCTGGGC 1500
pmxB_KP26_Polymyxin-E        CTGGTCATCGTACCGCCGCTCACGGCGAAAAATCCGGCTGATTTCCTGGTGCTGCTGGGC 1500
pmxB_Polymyxin-A             CTGGTCATCGTACCGCCGCTCACGGCGAAAAATCCGGCCGATTTCCTGGCGCTGCTGGGC 1500
pmxB_1G_Polymyxin            ctggtcatcgtaccgccgctcacggcgaaaaatccggtcgatttcctggcgctgctgggc 1500
pmxB-D-DaB3-Polymyxin-B      CTGGTCATCGTACCGCCGCTCACGGCGAAAAATCCAGCCGATTTCCTGGCGCTGCTGGGC 1500
pmxB_Polymyxin-P             CTGGTCATCGTACCGCCGCTCACGGCGAAAAATCCGGCCGATTTCCTGGCGCTGCTGGGC 1500
                             *********************************** *  ********** **********

pmxB_B-LR_Polymyxin-E        CACGAACAGGTCACGATTTTGAACCAGACGCCAACGTACTTCTACCAGCTGCTGCGTAAG 1560
pmxB_KP26_Polymyxin-E        CACGAACAGGTCACGATTTTGAACCAGACGCCAACGTACTTCTACCAGCTGCTGCGTAAG 1560
pmxB_Polymyxin-A             CGCGAACAGGTTACCATTTTGAACCAGACGCCAACGTACTTCTACCAGCTGCTGCGTAAG 1560
pmxB_1G_Polymyxin            cgcgaacaggtcacgattttgaaccagacgccaacgtacttctaccagctgctgcgtaag 1560
pmxB-D-DaB3-Polymyxin-B      CGCGAACAGGTCACGATTTTGAACCAGACGCCAACGTACTTCTACCAGCTGCTGCGTAAG 1560
pmxB_Polymyxin-P             CGCGAACAGGTCACGATTTTGAACCAGACACCAACGTACTTCTATCAGCTGCTGCGTAAG 1560
                             * ********* ** ************** ************** ***************

pmxB_B-LR_Polymyxin-E        GTCTTGGCGGACCATCCGTACGATCTGCGGATTCGCAACGTCATCTTCGGGGGCGAAGCG 1620
pmxB_KP26_Polymyxin-E        GTCTTGGCGGACCATCCGTACGATCTGCGGATTCGCAACGTCATCTTCGGGGGCGAAGCG 1620
pmxB_Polymyxin-A             GTCTTGGCGGACCATCCGTACGATCTGCGGATTCGCAACGTCATCTTCGGGGGCGAAGCG 1620
pmxB_1G_Polymyxin            gtcttggcggaccatccgtacgatctgcggattcgcaacgtcatcttcgggggcgaagcg 1620
pmxB-D-DaB3-Polymyxin-B      GTCTTGGCGGACCATCCGTACGATCTGCGGATTCGCAACGTCATCTTCGGGGGCGAAGCG 1620
pmxB_Polymyxin-P             GTCTTGGCGGACCATCCGTACGATCTGCGGATTCGCAACGTCATCTTCGGGGGCGAAGCG 1620
                             ************************************************************

pmxB_B-LR_Polymyxin-E        CTGAGTCCGCTGCTGCTCAAGGGCTTCAAGACGAAGTACCCGGAGACGAAGCTGATCAAT 1680
pmxB_KP26_Polymyxin-E        CTGAGTCCGCTGCTGCTCAAGGGCTTCAAGACGAAGTACCCGGAGACGAAGCTGATCAAT 1680
pmxB_Polymyxin-A             CTGAGTCCGCTGTTGCTCAAGGGCTTCAAGACGAAGTACCCGGAGACGAAGCTGATTAAT 1680
pmxB_1G_Polymyxin            ctgagtccgctgctgctcaagggcttcaagacgaagtacccggagacgaagctgatcaat 1680
pmxB-D-DaB3-Polymyxin-B      CTGAGTCCGCTGCTGCTCAAGGGCTTCAAGACGAAGTACCCGGAGACGAAGCTGATCAAT 1680
pmxB_Polymyxin-P             CTGAGTCCGCTGCTGCTCAAGGGCTTCAAGACGAAGTACCCAGAGACGAAGCTGATCAAT 1680
                             ************ **************************** ************** ***

pmxB_B-LR_Polymyxin-E        ATGTACGGCATTACCGAGACGACAGTTCACGTTACGTATAAGGAAATCACCTGGGTCGAA 1740
pmxB_KP26_Polymyxin-E        ATGTACGGCATTACCGAGACGACAGTTCACGTTACGTATAAGGAAATCACCTGGGTCGAA 1740
pmxB_Polymyxin-A             ATGTACGGCATTACCGAGACGACGGTTCACGTGACGTATAAGGAAATCACGTGGGTCGAA 1740
```

```
pmxB_1G_Polymyxin            atgtacggcattaccgagacgacggttcacgtgacgtataaggaaataacgtgggtcgaa 1740
pmxB-D-DaB3-Polymyxin-B      ATGTACGGCATTACCGAGACGACGGTTCACGTTACGTATAAGGAAATCACGTGGGTCGAA 1740
pmxB_Polymyxin-P             ATGTATGGCATTACCGAGACGACGGTTCACGTGACGTATAAGGAAATAACGTGGGTCGAA 1740
                             ***** ***************** ******** ************** ** *********

pmxB_B-LR_Polymyxin-E        ATGGAGGCGGCGAAGAGCAATATCGGCAAGCCGATCCCGACGCTGAGGGTGTACGTCCTT 1800
pmxB_KP26_Polymyxin-E        ATGGAGGCGGCGAAGAGCAATATCGGCAAGCCGATCCCGACGCTGAGGGTGTACGTCCTT 1800
pmxB_Polymyxin-A             ATGGAGGCGGCGAAGAGCAATATCGGCAAGCCGATCCCGACGTTGAGGGTGTACGTCCTG 1800
pmxB_1G_Polymyxin            atggaggcggcgaagagcaatatcggcaagccgatcccgacgctgagggtgtacgtcctg 1800
pmxB-D-DaB3-Polymyxin-B      ATGGAGGCGGCGAAGAGCAATATCGGCAAGCCGATCCCGACGCTGAGCGTGTACGTCCTT 1800
pmxB_Polymyxin-P             ATGGAGGCGGCGAAGAGCAATATCGGCAAGCCGATCCCGACGCTGAGGGTGTACGTCCTG 1800
                             ****************************************** **** ***********

pmxB_B-LR_Polymyxin-E        GATGAAAACCGCCGCCCAGTGCCGATCGGCGTAGCAGGCGAAATGTACGTGGCCGGGGAA 1860
pmxB_KP26_Polymyxin-E        GATGAAAACCGCCGCCCAGTGCCGATCGGCGTAGCAGGCGAAATGTACGTGGCCGGGGAA 1860
pmxB_Polymyxin-A             GATGAAAACCGCCGCCTTGTGCCGATCGGCGTAGCGGGCGAAATGTACGTGGCCGGGGAA 1860
pmxB_1G_Polymyxin            gatgaaaaccaccgccctgtgccgatcggcgtagcgggcgaaatgtacgtggccggggaa 1860
pmxB-D-DaB3-Polymyxin-B      GATGAAAACCGCCGCCCTGTGCCGATCGGCGTAGCGGGCGAAATGTACGTGGCCGGGGAA 1860
pmxB_Polymyxin-P             GATGAAAACCGCCGCCCTGTGCCGATTGGCGTAGCGGGCGAAATGTACGTGGCCGGGGAA 1860
                             ********** *****  ******** ******** ************************

pmxB_B-LR_Polymyxin-E        GGCCTGGCGAGAGGATACCTGAACCGTCCGGATCTGACGGCGGAGAAGTTCGTCGATTCC 1920
pmxB_KP26_Polymyxin-E        GGCCTGGCGAGAGGATACCTGAACCGTCCGGATCTGACGGCGGAGAAGTTCGTCGATTCC 1920
pmxB_Polymyxin-A             GGCCTTGCGAGAGGATACCTGAACCGTCCGGATCTGACGGCGGAGAAGTTCGTCGATTCC 1920
pmxB_1G_Polymyxin            gggcttgcgagaggatacctgaaccgtccagatctgacggcggagaagttcgtcgattcc 1920
pmxB-D-DaB3-Polymyxin-B      GGCCTTGCGAGAGGATACCTGAACCGCCCGGATCTGACGGCGGAGAAGTTCGTCGATTCC 1920
pmxB_Polymyxin-P             GGCCTTGCGAGAGGATACCTGAACCGTCCGGATCTGACGGCGGAGAAGTTCGTCGATTCC 1920
                             ** ** ******************** ** ******************************

pmxB_B-LR_Polymyxin-E        CCGTTTGCGGAGGGGGAGAAACTATACCGCTCGGGCGACTTGGCGGTTTGGCTGCCGGAC 1980
pmxB_KP26_Polymyxin-E        CCGTTTGCGGAGGGGGAGAAACTATACCGCTCGGGCGACTTGGCGGTTTGGCTGCCGGAC 1980
pmxB_Polymyxin-A             CCGTTTGCGGAGGGGGAGAGACTATACCGCTCGGGCGACTTGGCGGCTTGGCTGCCGGAC 1980
pmxB_1G_Polymyxin            ccgtttgcggagggggagaaactgtaccgctcgggcgacttggcgacttggctgccggac 1980
pmxB-D-DaB3-Polymyxin-B      CCGTTTGCGGAGGGGGAGAAACTGTACCGCTCGGGCGACTTGGCGGCTTGGCTGCCGGAC 1980
pmxB_Polymyxin-P             CCGTTTGCGGAGGGGGAGAAACTGTACCGCTCGGGTGACTTGGCGGCTTGGCTGCCGGAC 1980
                             ******************* *** *********** *********  *************

pmxB_B-LR_Polymyxin-E        GGCAACATCGAATATCTGGGCCGAATCGACCACCAAGTGAAAATCCGCGGGTACCGGATC 2040
pmxB_KP26_Polymyxin-E        GGCAACATCGAATATCTGGGCCGAATCGACCACCAAGTGAAAATCCGCGGGTACCGGATC 2040
pmxB_Polymyxin-A             GGCAACATCGAATACCTGGGCCGGATCGACCACCAGGTCAAAATCCGCGGGTACCGGATC 2040
pmxB_1G_Polymyxin            ggcaacatcgaatacctgggacggatcgaccaccaggtcaaaatccgcgggtaccggatc 2040
pmxB-D-DaB3-Polymyxin-B      GGCAACATCGAATACCTGGGCCGGATCGATCATCAGGTGAAAATCCGTGGGTACCGGATC 2040
pmxB_Polymyxin-P             GGCAACATCGAATACCTGGGCCGGATCGACCACCAGGTGAAAATCCGCGGGTACCGGATC 2040
                             ************** ***** ** ***** ** ** ** ******** ************
```

```
pmxB_B-LR_Polymyxin-E        GAGCTGGACGAAATCGAGACGCAGTTGCTGAAGATTGCCGCCGTGCAAGAAGCCAAGGTG 2100
pmxB_KP26_Polymyxin-E        GAGCTGGACGAAATCGAGACGCAGTTGCTGAAGATTGCCGCCGTGCAAGAAGCCAAGGTG 2100
pmxB_Polymyxin-A             GAGCTGGACGAAATCGAGACGCAGCTTCTGAACGCCCGGGGCGTGGAAGAAGCGGTGGTG 2100
pmxB_1G_Polymyxin            gagctggacgaaatcgagacgcaactgctgaagatcgccgccgtgcaagaagccaaggtg 2100
pmxB-D-DaB3-Polymyxin-B      GAGCTGGACGAAATCGAGACGCAGCTGCTGAAGATCGCCGCCGTGCAAGAAGCCAAGGTG 2100
pmxB_Polymyxin-P             GAGTTGGACGAAATCGAGACGCAGCTACTGAAGATCGCCGCCGTGCAAGAAGCCAAGGTG 2100
                             *** *******************  * *****       * **** *******   ****

pmxB_B-LR_Polymyxin-E        CTCGACCGCGACGACGCGAACGGCCAAAAGCAGCTTGTCGCTTACTACGTCGCGGAAATG 2160
pmxB_KP26_Polymyxin-E        CTCGACCGCGACGACGCGAACGGCCAAAAGCAGCTTGTCGCTTACTACGTCGCGGAAATG 2160
pmxB_Polymyxin-A             CTCGCCCGCGACGACGCGCACGGCCATAAGCAGCTTGTCGCTTATTACGTCGCGGAAACG 2160
pmxB_1G_Polymyxin            ctcgactgcgacgacgcgaacggccaaaagcagcttgtcgcttactatgtcgcggaaacg 2160
pmxB-D-DaB3-Polymyxin-B      CTCGACCGCGACGACGCGAACGGCCATAAGCAGCTTGTCGCTTATTACGTCGCGGAAACG 2160
pmxB_Polymyxin-P             CTCGACCGCGACGACGCGAACGGCCATAAGCAGCTTGTCGCTTATTACGTCGCGGAAACG 2160
                             **** * *********** ******* ***************** ** ********** *

pmxB_B-LR_Polymyxin-E        AGGCTGGCGGCGCATGAACTCAAGGAGGAGCTCGCCAAACAGCTTCCAGGGTATATGATT 2220
pmxB_KP26_Polymyxin-E        AGGCTGGCGGCGCATGAACTCAAGGAGGAGCTCGCCAAACAGCTTCCAGGGTATATGATT 2220
pmxB_Polymyxin-A             AGGCTGGCGGCGAATGAACTCAAGGAGGAGCTCGCCAAGCAGCTTCCAGGGTATATGATT 2220
pmxB_1G_Polymyxin            aggctggcggcgcatgaactcaaggaggagctcgccaagcagcttccggggtatatgatt 2220
pmxB-D-DaB3-Polymyxin-B      AGGCTGGCGGCGCATGAACTCAAGGAGGAGCTCGCCAAGCAGCTTCCGGGGTATATGATT 2220
pmxB_Polymyxin-P             AGGCTGGCGGCGAATGAACTCAAGGAGGAGCTCGCCAAGCAGCTTCCGGGGTATATGATT 2220
                             ************ ************************* ******** ************

pmxB_B-LR_Polymyxin-E        CCTTCGCACTTCGTGCAGCTTTCGCGGATGCCTCTAACCCCGAACGGAAAAATCGACCGC 2280
pmxB_KP26_Polymyxin-E        CCTTCGCACTTCGTGCAGCTTTCGCGGATGCCTCTAACCCCGAACGGAAAAATCGACCGC 2280
pmxB_Polymyxin-A             CCTTCGCACCTCGTGCAGCTTTCGCGGATGCCGCTGACCCCGAACGGGAAAATCGACCGC 2280
pmxB_1G_Polymyxin            ccttcgtacctcgtgcagctttcggggatgccgctgaccccgaacgggaaaatcgaccgc 2280
pmxB-D-DaB3-Polymyxin-B      CCTTCGCACCTCGTGCAGCTTTCGCGGATGCCGCTGACCCCGAACGGGAAAATCGACCGC 2280
pmxB_Polymyxin-P             CCTTCGCACCTCGTGCAGCTTTCGGGGATGCCGCTGACCCCGAACGGGAAAATCGACCGC 2280
                             ****** ** ************** ******* ** *********** ************

pmxB_B-LR_Polymyxin-E        AAAGCGCTGCCAGCGCCGGAGGAAGTCGCGGCCTTAGGAGCGGAATATGTCGCGCCGAGA 2340
pmxB_KP26_Polymyxin-E        AAAGCGCTGCCAGCGCCGGAGGAAGTCGCGGCCTTAGGAGCGGAATATGTCGCGCCGAGA 2340
pmxB_Polymyxin-A             AAAGCGCTGCCCGCGCCGGAGGAAGCCGCGGCCGGAGGAGCGGAATATGTCGCGCCGAGA 2340
pmxB_1G_Polymyxin            aaagcgctgcccgcgccggaggaagccgcggtcggaggagcggaatatgtcgcgccgaga 2340
pmxB-D-DaB3-Polymyxin-B      AAAGCGCTGCCCGCGCCGGAGGAAGCCGCGGCCGGAGGAGCGGAATATGTCGCGCCGAGA 2340
pmxB_Polymyxin-P             AAAGCACTGCCCGCGCCGGAGGAAGCCGCGGCTGGAGGAGCGGAATATGTCGCACCGAGA 2340
                             ***** ***** ************* *****    ****************** ******

pmxB_B-LR_Polymyxin-E        ACGCTGCTCGAAATGAAGATTGCCCGCGTCTGGCAGGATACGCTTGGCGTTCCGCAGGTC 2400
pmxB_KP26_Polymyxin-E        ACGCTGCTCGAAATGAAGATTGCCCGCGTCTGGCAGGATACGCTTGGCGTTCCGCAGGTC 2400
pmxB_Polymyxin-A             ACGCTGCTCGAAATGAAGATCGTCCGCGTCTGGCAGGATACGCTTGGCGTTCCGCAGGTC 2400
```

```
pmxB_1G_Polymyxin            acgctgcttgaaatgaagatcgcccgcgtctggcaggatacgcttggcgttccgcaggtc 2400
pmxB-D-DaB3-Polymyxin-B      ACGCTGCTCGAAATGAAGATCGCCCGCGTTTGGCAGGATACGCTTGGCGTTCCGCAGGTC 2400
pmxB_Polymyxin-P             ACGCTGCTCGAAATGAAGATCGCACGCGTCTGGCAGGATACGCTTGGCGTTCCGCAGGTC 2400
                             ******** *********** *  ***** ******************************

pmxB_B-LR_Polymyxin-E        GGCGTAAAGGATAACTTTTTTGATTTGGGTGGCAATTCGTTAAGTCTGATGAGGCTCGTT 2460
pmxB_KP26_Polymyxin-E        GGCGTAAAGGATAACTTTTTTGATTTGGGTGGCAATTCGTTAAGTCTGATGAGGCTCGTT 2460
pmxB_Polymyxin-A             GGCGTAAAGGATAACTTTTTTGAGTTGGGTGGCAATTCGTTAAGTCTGATGAGGCTCGTT 2460
pmxB_1G_Polymyxin            ggcgtaaaggataacttttttgagttgggtggcaattcgttaagtctgatgaggctcgtt 2460
pmxB-D-DaB3-Polymyxin-B      GGCGTAAAGGATAACTTTTTTGAGTTGGGTGGCAATTCGTTAAGTCTGATGAGGCTCGTT 2460
pmxB_Polymyxin-P             GGCGTAAAGGATAACTTTTTTGAGTTGGGTGGCAATTCGTTAAGTCTGATGAGGCTCGTT 2460
                             *********************** ************************************

pmxB_B-LR_Polymyxin-E        CAAGCCGTTTACGATGAAACGGGCATTGAGATTCCGCTGAATCGCCAATTTCATCATGTA 2520
pmxB_KP26_Polymyxin-E        CAAGCCGTTTACGATGAAACGGGCATTGAGATTCCGCTGAATCGCCAATTTCATCATGTA 2520
pmxB_Polymyxin-A             CAAGCCGTTTACGATGAAACGGGCATTGAGATACCACTGAACCGCCAATTCCATAATGTA 2520
pmxB_1G_Polymyxin            caagccgtttatgatgaaacgggatttgagataccgctgaaccgccaattccataatgta 2520
pmxB-D-DaB3-Polymyxin-B      CAAGCCGTTTACGATGAAACGGACATCGAGATACCGCTGAACCGCCAATTCCACAATCTA 2520
pmxB_Polymyxin-P             CAAGCCGTTTACGATGAAACGGGCATCGAGATACCTTTGAACCGCCAATTCCACAATGTA 2520
                             *********** **********   * ***** **  **** ******** **  ** **

pmxB_B-LR_Polymyxin-E        ACCGTTGAAGCCATGGCTTTCGAAGAGGGGGATCTGGGCCTGGATAAAGGGGGAGACTCC 2580
pmxB_KP26_Polymyxin-E        ACCGTTGAAGCCATGGCTTTCGAAGAGGGGGATCTGGGCCTGGATAAAGGGGGAGACTCC 2580
pmxB_Polymyxin-A             ACAGTTGAAGCCATGGCTTTCGGAGAGGGGGATCTGGGCCTGGATAAAGGGGGAGACTCC 2580
pmxB_1G_Polymyxin            acagttgaagccatggctttcggagagggggatctcggcctggataaaggaggagactcc 2580
pmxB-D-DaB3-Polymyxin-B      ACAGTTGAAGCTATGGCTTTCGGCAAGGGGGATCTGGGCCTGGATAAAGGGGGAGACTCC 2580
pmxB_Polymyxin-P             ACAGTTGAAGCCATGGCTTTCGGCGAGTCGGATCTCGGCCTGGATAAAGGGGGAAACTCC 2580
                             ** ******** **********   **  ****** ************** *** *****

pmxB_B-LR_Polymyxin-E        TTCATAAAGCTGAATAAAGCAGGAGATCTGAACGTGTTCTGCTTCCCTCCGGGCAGCGGC 2640
pmxB_KP26_Polymyxin-E        TTCATAAAGCTGAATAAAGCAGGAGATCTGAACGTGTTCTGCTTCCCTCCGGGCAGCGGC 2640
pmxB_Polymyxin-A             TTCATTAAGCTGAATAAAGCAGGAGATCTGAACGTGTTCTGCTTCCCTCCGGGCAGCGGC 2640
pmxB_1G_Polymyxin            ttcattaagctgaataaagcaggagatctgaacgtgttctgcttccctccgggcagcggc 2640
pmxB-D-DaB3-Polymyxin-B      TTCATTAAGCTGAATAAAGCAGGAGATCTGAACGTGTTCTGTTTCCCTCCGGGCAGCGGC 2640
pmxB_Polymyxin-P             TTCATTAAGCTGAATAAAGCAGGAGATCTGAACGTGTTCTGCTTCCCTCCGGGTAGCGGT 2640
                             ***** ********************************** ************ *****

pmxB_B-LR_Polymyxin-E        TTCGGCATCGGTTACCGAGAGCTCGCAAGCAGGCTCGACGGCCAGTTCGTGCTCTACGGC 2700
pmxB_KP26_Polymyxin-E        TTCGGCATCGGTTACCGAGAGCTCGCAAGCAGGCTCGACGGCCAGTTCGTGCTCTACGGC 2700
pmxB_Polymyxin-A             TTCGGCATCGGTTACCGGGAGCTCGCAAGCAGGCTCGACGGCCGGTTCGTGCTCTACGGC 2700
pmxB_1G_Polymyxin            ttcggcatcggttatcgagagctcgcaagcaggctcgacggccggttcgtgctctacggc 2700
pmxB-D-DaB3-Polymyxin-B      TTCGGCATCGGTTACCGAGAGCTCGCAAGCAGGCTCGACGGCCGGTTCGTGCTCTACGGC 2700
pmxB_Polymyxin-P             TTCGGCATCGGTTACCGAGAGCTCGCAAGCAGGCTCGACGGCCGGTTCGTGCTCTACGGC 2700
                             ************** ** ************************* ****************
```

```
pmxB_B-LR_Polymyxin-E        ATTGATTTTATCGACGATACCGCCGATTACGAGGCCATGCTGAACCGTTATGTTGACGAG 2760
pmxB_KP26_Polymyxin-E        ATTGATTTTATCGACGATACCGCCGATTACGAGGCCATGCTGAACCGTTATGTTGACGAG 2760
pmxB_Polymyxin-A             ATTGATTTTATCGACGATGCCGCCGATTACGAAGCCATGCTGAACCGTTATGTGGACGAG 2760
pmxB_1G_Polymyxin            attgattttatcgacgatgccgtcgattacaaggccatgctgaaccgttatgtggacgag 2760
pmxB-D-DaB3-Polymyxin-B      ATTGATTTTATCGACGATGCCGTCGATTACGAGGCCATGCTGAACCGTTATGTGGACGAG 2760
pmxB_Polymyxin-P             ATTGATTTTATCGACGATGCCGTCGATTACGAGGCCATGCTGAACCGTTATGTGGACGAG 2760
                             ****************** *** ******* * ******************** ******

pmxB_B-LR_Polymyxin-E        ATTGTCCGCATCCAGCCGGAAGGACCTTACGTGCTGCTCGGCTACTGCTTCGGAGGCAAC 2820
pmxB_KP26_Polymyxin-E        ATTGTCCGCATCCAGCCGGAAGGACCTTACGTGCTGCTCGGCTACTGCTTCGGAGGCAAC 2820
pmxB_Polymyxin-A             ATCGTCCGCATCCAGCCGGAAGGACCTTACGTGCTGCTCGGCTACTGCTTTGGAGGCAAC 2820
pmxB_1G_Polymyxin            atcgtccgcatccagccggaaggaccttacgtgctgctcggctactgcttcggaggcaac 2820
pmxB-D-DaB3-Polymyxin-B      ATCGTCCGCATCCAGTCGGAAGGACCTTACGTGCTGCTCGGCTACTGCTTCGGAGGCAAC 2820
pmxB_Polymyxin-P             ATCGTCCGCATCCAGTCGGAAGGACCTTACGTGCTGCTCGGCTACTGCTTCGGAGGCAAC 2820
                             ** ************ ********************************** *********

pmxB_B-LR_Polymyxin-E        CTGACGTTCGAGGTAGCCAAAACGATGGAGAAAAGAGGGTATCCCGTAACGGACGTGCTC 2880
pmxB_KP26_Polymyxin-E        CTGACGTTCGAGGTAGCCAAAACGATGGAGAAAAGAGGGTATCCCGTAACGGACGTGCTC 2880
pmxB_Polymyxin-A             CTGATGTTCGAGGTAGCCAAAACGATGGAGAAAAGAGAGTATTCCGTAACGGACGTGCTC 2880
pmxB_1G_Polymyxin            ctgacgttcgaggtagccaaaacgatggagaaaagagggtattccgtaacggacgtgctc 2880
pmxB-D-DaB3-Polymyxin-B      CTGACGTTCGAGGTAGCCAAAACGATGGAGAAAAGAGGGTATTCCGTAACGGACGTGCTC 2880
pmxB_Polymyxin-P             CTGACGTTCGAGGTAGCCAAAACGATGGAGAAAAGAGGGTATTCCGTAACGGACGTGCTC 2880
                             **** ******************************** **** *****************

pmxB_B-LR_Polymyxin-E        ATGGTGGACTCGTGGATTAAGGAGACGCTGACGCCTTCCGAAACGTCAGAGAAAGAGCTT 2940
pmxB_KP26_Polymyxin-E        ATGGTGGACTCGTGGATTAAGGAGACGCTGACGCCTTCCGAAACGTCAGAGAAAGAGCTT 2940
pmxB_Polymyxin-A             ATGGTGGACTCGTGGATTAAGGACACGCTGACGCCTTCCGAAACGTCGGAGAAAGAGCTT 2940
pmxB_1G_Polymyxin            atggtggactcgtggattaaggacgcgctgacgccttccgaaacgtcggagaaagagctt 2940
pmxB-D-DaB3-Polymyxin-B      ATGGTGGACTCGTGGATTAAGGACACGCTGACGCCTTCCGAAACGTCGGAGAAAGAGCTT 2940
pmxB_Polymyxin-P             ATGGTGGACTCGTGGATTAAGGACGCGCTGACGCCTTCTGAAACGTCGGAGAAAGAGCTT 2940
                             ***********************  ************* ******** ************

pmxB_B-LR_Polymyxin-E        GAAGAAATGCTTGCCGATTTCGACGAAGAAGAGAAGGAATTAATGAGCAATCCGCTCGTG 3000
pmxB_KP26_Polymyxin-E        GAAGAAATGCTTGCCGATTTCGACGAAGAAGAGAAGGAATTAATGAGCAATCCGCTCGTG 3000
pmxB_Polymyxin-A             GAAGAAACGCTTGCCGATTTCGACGAAGAAGAGAAGGAATTAATGAGCAACCCGCTCGTG 3000
pmxB_1G_Polymyxin            gaagaaatgcttgctgatttcgacgaagaagagaaggaattaatgagcaacccgctcgtg 3000
pmxB-D-DaB3-Polymyxin-B      GAAGAAACGCTTGCCGATTTCGACGAAGAAGAGAATGAATTAATGAGCAACCCGCTCGTG 3000
pmxB_Polymyxin-P             GAAGAAATGCTTGCGGATTTCGACGAAGAAGAGAAGGAATTAATGAGCAACCCGCTCGTG 3000
                             ******* ****** ******************** ************** *********

pmxB_B-LR_Polymyxin-E        CGGGAGCGGGTTCATCAGAAGGTCAAAGCGACTTTGGCGTACGAAGCGCAGCTTATTAAC 3060
pmxB_KP26_Polymyxin-E        CGGGAGCGGGTTCATCAGAAGGTCAAAGCGACTTTGGCGTACGAAGCGCAGCTTATTAAC 3060
pmxB_Polymyxin-A             CGGGAGCGTGTTCATCGGAAGGTCAAAGCGACCTTGGCGTACGAAGCGCAGCTTATGAAT 3060
```

```
pmxB_1G_Polymyxin            cgggagcgggttcatcggaaggtcaaagcgaccttggcgtacgaagcgcagcttattaat 3060
pmxB-D-DaB3-Polymyxin-B      CGGGAGCGGGTTCATCGGAAGGTCAAAGCGACCTTGGCGTACGAAGCGCAGCTTATTAAT 3060
pmxB_Polymyxin-P             CGGGAGCGGGTTCATCGGAAGGTCAAAGCGACCTTGGCGTACGAAGCGCAGCTTATTAAT 3060
                             ******** ******* *************** ********************** **

pmxB_B-LR_Polymyxin-E        TCCGGCACGATCCCGGCCCGGATTTACGAACTGATTGCGAAGGACAGCGAAGCGTTCCGC 3120
pmxB_KP26_Polymyxin-E        TCCGGCACGATCCCGGCCCGGATTTACGAACTGATTGCGAAGGACAGCGAAGCGTTCCGC 3120
pmxB_Polymyxin-A             TCCGGCACGATCCCGGCCAGGATTTACGAACTGATTGCGAAGGACAGCGAAGCGTTCCGC 3120
pmxB_1G_Polymyxin            tccggcacgatcccggcccggatttacgaactgattgcgaaggacagcgaagcgttccgc 3120
pmxB-D-DaB3-Polymyxin-B      TCCGGCACGATCCCGGCCCGGATTTACGAACTGATTGCGAAGGACAGCGAAGCGTTCCGC 3120
pmxB_Polymyxin-P             TCCGGCACGATCCCGGCCAGGATTTATGAACTGATTGCGAAGGACAGCGAAGCTTTCCGC 3120
                             ****************** ******* ************************* ******

pmxB_B-LR_Polymyxin-E        TTGGAGCACCAATTGCCGTCCTGGCGGGGGGCAACGACGCAAGCTTACACCGATTACCGG 3180
pmxB_KP26_Polymyxin-E        TTGGAGCACCAATTGCCGTCCTGGCGGGGGGCAACGACGCAAGCTTACACCGATTACCGG 3180
pmxB_Polymyxin-A             CTGGAGCACCAATTGCCGTCCTGGCGGGGGGCAACGACGCAAGCTTACGCCGATTACCGG 3180
pmxB_1G_Polymyxin            ttgaagtaccaattgccttcctggcggggggcaacgacgcaagcttatgccgattaccgg 3180
pmxB-D-DaB3-Polymyxin-B      CTGGAGCACCAATCGCCTTCCTGGCGGGGGGCAACGACGCAAGCTTACGCCGATTACCGG 3180
pmxB_Polymyxin-P             CTGGAGCACCAATTGCCTTCCTGGCGGGGGGCAACGACGCAAGCTTACTCCGATTACCGG 3180
                              ** ** ****** *** *****************************  ***********

pmxB_B-LR_Polymyxin-E        CTGGAGGGCGCGCACGAGGAATTGCTGGAACTCGCGCGCGTGGACGAAACGGCCGTTGTC 3240
pmxB_KP26_Polymyxin-E        CTGGAGGGCGCGCACGAGGAATTGCTGGAACTCGCGCGCGTGGACGAAACGGCCGTTGTC 3240
pmxB_Polymyxin-A             CTGGAGGGCGCGCACGAGGAATTGCTGGAACTCGCGCGCGTGGACGAAACGGCCGTTGTC 3240
pmxB_1G_Polymyxin            ctggagggcgcgcacgaggaattgctggaactcgcgcgcgtggaagaaacggccgtcgtc 3240
pmxB-D-DaB3-Polymyxin-B      CTGGAGGGCGCGCACGAGGAATTGCTGGAACTCGCGCGCGTGGAAGAAACGGCCGTCGTC 3240
pmxB_Polymyxin-P             CTGGAGGGTGCGCACGAGGAATTGCTGGAACTCGCGCGCGTGGAAGAAACGGCCGTCGTC 3240
                             ******** *********************************** *********** ***

pmxB_B-LR_Polymyxin-E        ATCCGGGATATCTTAGAGCAAGTCAAGCGGCAGATCGAAGTGGAGGCCGGGGTACTGCAT 3300
pmxB_KP26_Polymyxin-E        ATCCGGGATATCTTAGAGCAAGTCAAGCGGCAGATCGAAGTGGAGGCCGGGGTACTGCAT 3300
pmxB_Polymyxin-A             ATCCGGGACATCTTAGAACAAGTCAAGCGGCAGATCGAAGCGGAGGCCGGGGTACTGCAT 3300
pmxB_1G_Polymyxin            atccgggacatcttagagcaagtcaagcggcaaatcgaagcggaggccggggtactgcat 3300
pmxB-D-DaB3-Polymyxin-B      ATCCGGGACATCTTAGAGCAAGTCAAGCGGCAGATCGAAGCGGAGGCCGGGGTACTGCAT 3300
pmxB_Polymyxin-P             ATTCGAGACATCTTAGAGCAAGTCAAGCGGCAGATCGAAGCGGAGGCCGGGGTACTGCAT 3300
                             ** ** ** ******** *************** ******* *******************

pmxB_B-LR_Polymyxin-E        GGAAGCTGA  3309
pmxB_KP26_Polymyxin-E        GGAAGCTGA  3309
pmxB_Polymyxin-A             GGAAGCTGA  3309
pmxB_1G_Polymyxin            ggaagctga  3309
pmxB-D-DaB3-Polymyxin-B      GGAAGCTGA  3309
pmxB_Polymyxin-P             GGAAGCTGA  3309
                             *********
```

CLUSTAL O(1.2.4) multiple sequence alignment of pmxE

```
pmxE-D-DaB3-Polymyxin-B      GTGAGAGAGAATACCAACGAGCAATATGGATTAACGCAAGCCCAGCGCCGAATATGGTTC 60
pmxE_1G_Polymyxin            gtgagagagaataccaacgagcaatatggattaacgcaagcccagcgccgaatatggttc 60
pmxE_B-LR_Polymyxin-E        GTGAGAGATAATACCAACGGGCAATATGAATTAACGCAAGCCCAGCGCCGAATATGGTTC 60
pmxE_KP26_Polymyxin-E        ------------------------------------------------------------ 0
pmxE_Polymyxin-P             GTGAGAGAGAATACCAACGAGAAATATGGATTAACGCAAGCCCAGCGCCGAATATGGTTC 60
pmxE_Polymyxin-A             GTGAGAGAGAATACCAAAGAGCAATATGGATTAACGCAAGCCCAGCGCCGAATATGGTTC 60


pmxE-D-DaB3-Polymyxin-B      ATGGAAATTATGAATCCGGGAACGTCCATCACGATGCTTTCCGCGACCTACCAGATTACG 120
pmxE_1G_Polymyxin            atggaaattatgaatccgggaacgtccatcacgatgctttccgcgacctaccagattacg 120
pmxE_B-LR_Polymyxin-E        ATGGAAATTATGAATCCGGGAACGTCCATCACGATGCTTTCCGCGACCTACCAGATTACG 120
pmxE_KP26_Polymyxin-E        ATGGAAATTATGAATCCGGGAACGTCCATCACGATGCTTTCCGCGACCTACCAGATTACG 60
pmxE_Polymyxin-P             ATGGAAATTATGAATCCGGGAACGTCCATCACGATGCTTTCCGCGACCTACCAGATTACG 120
pmxE_Polymyxin-A             ATGGAAATTATGAATCCGGGAACGTCCATCACGATGCTTTCCGCGACCTACCAGATTACG 120
                             ************************************************************

pmxE-D-DaB3-Polymyxin-B      GGCGAGATCAACACACAGCTTCTGGAGCAAGCGGTGGCAGAGATCGTCAAAACCTATGAC 180
pmxE_1G_Polymyxin            ggcgagatcaacacacagcttctggagcaagcggcggcagagatcgtcaaaacctatgac 180
pmxE_B-LR_Polymyxin-E        GGCCAGATCGACACACAGCTTCTGGAGCAAGCTGCGGCGGAGATCGTCAAAACCTATGAC 180
pmxE_KP26_Polymyxin-E        GGCCAGATCGACACACAGCTTCTGGAGCAAGCTGCGGCGGAGATCGTCAAAACCTATGAC 120
pmxE_Polymyxin-P             GGCGAGATCAACGCACAGCTTCTGGAGCAAGCGGCGGCAGAGATCGTCAAAACCTATGAC 180
pmxE_Polymyxin-A             GGCGAGATCAACACACAGCTTCTGGAGCAAGCGGCAGCAGAGATCGTCAAAACCTATGAC 180
                             *** ***** ** ******************* *  ** *********************

pmxE-D-DaB3-Polymyxin-B      GTTTTCCGAATCCGCATTAGCGGGGATTTGCAAAATCCAACGCAGTGGTTCGAAGAGCCG 240
pmxE_1G_Polymyxin            gttttccgaatccgcattagcggggatttgcaaaatccaacgcagtggttcgaagagccg 240
pmxE_B-LR_Polymyxin-E        GCTTTCCGAATACGCATTAGCGGGGATTTGCAGAATCCAACGCAGTGGTTCGAAGAGCCG 240
pmxE_KP26_Polymyxin-E        GCTTTCCGAATACGCATTAGCGGGGATTTGCAGAATCCAACGCAGTGGTTCGAAGAGCCG 180
pmxE_Polymyxin-P             GTTTTCCGAATCCGCATTAGCGGGGATTTGCAAAATCCAACGCAGTGGTTCGAAGAGCCG 240
pmxE_Polymyxin-A             GTTTTCCGAATCCGCATTAGCGGGGATTTGCAAAATCCAACGCAGTGGTTCGAAGAGCCG 240
                             * ********* ******************* ****************************

pmxE-D-DaB3-Polymyxin-B      GAGAATGTCCAAGCTAGGATAAGCCGCCTTGAAATAGGCACAACCGAACAATTCTATGCT 300
pmxE_1G_Polymyxin            gagaatgtccaagctaggataagccgcctcgaaataggcacaaccgaacaattctatgct 300
pmxE_B-LR_Polymyxin-E        GAGAATGTCCAAGCCAGGATAAGCCGCCTCGAAATAGGCACAACCGAACAATTCTATGCT 300
pmxE_KP26_Polymyxin-E        GAGAATGTCCAAGCCAGGATAAGCCGCCTCGAAATAGGCACAACCGAACAATTCTATGCT 240
pmxE_Polymyxin-P             GAGAATGTCCAAGCTAGGATAAGCCGCCTCGAAATAGGCACAACCGAACAATTCTATGCT 300
pmxE_Polymyxin-A             GAGAATGTCCAGGCTAGGATAAGCCGCCTCGAAATAGGCACAACCGAACAATTCTATGCT 300
                             *********** ** ************** ******************************
```

```
pmxE-D-DaB3-Polymyxin-B      TGGGTGAAAGAAGTAAGCGAAAAACCGGCCAGCGTGTTCGACGAACACCTCTACCAATTT 360
pmxE_1G_Polymyxin            tgggtgaaagaagtaagcgaaaaaccggccagcgtgttcgacgaacacctctaccaattt 360
pmxE_B-LR_Polymyxin-E        TGGGTGAAAGAAGTAAGCGAAAAACCGGCCAGCGTGTTCGACGAACACCTCTACCAATTT 360
pmxE_KP26_Polymyxin-E        TGGGTGAAAGAAGTAAGCGAAAAACCGGCCAGCGTGTTCGACGAACACCTCTACCAATTT 300
pmxE_Polymyxin-P             TGGGTGAAAGAAGTAAGCGAAAAACCGGCCAGCGTGTTCGACGAACACCTCCACCAATTT 360
pmxE_Polymyxin-A             TGGGTGAAAGAAGTAAGCGAAAAACCGGCCAGCGTGTTCGACGAACACCTCCACCAATTT 360
                             *************************************************** ********

pmxE-D-DaB3-Polymyxin-B      ACGATTATCCATTTTGCGAACGGCCAAGTATGGCTCAATTTGACGGTAAATCATATTATC 420
pmxE_1G_Polymyxin            acgattatccattttgcgaacggccaagtatggctcaatttgacggtaaatcatattatc 420
pmxE_B-LR_Polymyxin-E        ACGATTATCCATTTTGCGAACGGCCAAGTATGGCTCAATTTGACGGTAAATCATATTATC 420
pmxE_KP26_Polymyxin-E        ACGATTATCCATTTTGCGAACGGCCAAGTATGGCTCAATTTGACGGTAAATCATATTATC 360
pmxE_Polymyxin-P             ACGATTATCCATTTTGCGAACGGCCAAGTATGGCTCAATTTGACGGTAAATCATATTATC 420
pmxE_Polymyxin-A             ACGATTATCCATTTTGTGAACGGCCAAGTATGGCTCAATTTGACGGTAAATCATATTATC 420
                             **************** *******************************************

pmxE-D-DaB3-Polymyxin-B      GCCGACGGCTTGTCCGTCAATGCTTTGCTGCATGCGGTGATGGAAAAATACCTGGAACTG 480
pmxE_1G_Polymyxin            gccgacggcttgtccgtcaatgctttgctgcatgcggtgatggaaaaatacctggaactg 480
pmxE_B-LR_Polymyxin-E        GCCGACGGCTTGTCCGTCACTGCTTTGCTGCATGCGGTGATGGAAAAATACATGGAACTG 480
pmxE_KP26_Polymyxin-E        GCCGACGGCTTGTCCGTCACTGCTTTGCTGCATGCGGTGATGGAAAAATACATGGAACTG 420
pmxE_Polymyxin-P             GCCGACGGCTTGTCCGTCAATGCTTTGCTGCATGCGGTGATGGAAAAATACCTGGAACTG 480
pmxE_Polymyxin-A             GCCGACGGCTTGTCCGTCAATGCTTTGCTGCATGCGGTGATGGAAAAATACCTGGAACTG 480
                             ******************* ******************************* ********

pmxE-D-DaB3-Polymyxin-B      CGCAAAGGCATCTCCAGCAGTTACCAGGCCCCTTCCTATCTGGATTATATTTCCGCGGAG 540
pmxE_1G_Polymyxin            cgcaaaggcatctccagcagttaccaggccccttcctatctggattatatttccgcggag 540
pmxE_B-LR_Polymyxin-E        CGCAAAGGCATCTCCAGCAGTTACCAGGCCCCTTCTTATCTGGATTATATTTCCGCGGAG 540
pmxE_KP26_Polymyxin-E        CGCAAAGGCATCTCCAGCAGTTACCAGGCCCCTTCTTATCTGGATTATATTTCCGCGGAG 480
pmxE_Polymyxin-P             CGCAAAGGCACCTACAGCAGTTACCAGGCCCCTTCCTATCTGGATTATATTTCTGCGGAG 540
pmxE_Polymyxin-A             CGCAAAGGCATCTCCAGCAGTTACCAGGCCCCTTCCTATCTGGATTATATTTCCGCGGAG 540
                             ********** ** ********************* ***************** ******

pmxE-D-DaB3-Polymyxin-B      CGTGAATATGAGCAATCGCAGCGTTATCAAAAAGGCAAGGAATACTGGCTGACGAAGTAC 600
pmxE_1G_Polymyxin            cgtgaatatgagcaatcgcagcgttatcaaaaaggcaaggaatactggctgacgaagtac 600
pmxE_B-LR_Polymyxin-E        CGTGAATATGAGCAATCGCAGCGTTATCAAAAAGGCAAGGAATACTGGCTGACGAAGTAC 600
pmxE_KP26_Polymyxin-E        CGTGAATATGAGCAATCGCAGCGTTATCAAAAAGGCAAGGAATACTGGCTGACGAAGTAC 540
pmxE_Polymyxin-P             CGTGAATATGAGCAATCGCAGCGTTATCAAAAAGGCAAGGAATACTGGCTGACGAAGTAC 600
pmxE_Polymyxin-A             CGTGAATATGAGCAATCGCAGCGTTATCAAAAAGGCAAGGAATACTGGCTGACGAAGTAC 600
                             ************************************************************

pmxE-D-DaB3-Polymyxin-B      AACACTTTGCCTGAAACGACCGGCATTAAATCGTATCCGCCATTCTCGATCGGCAGCGAA 660
pmxE_1G_Polymyxin            aacactttgcctgaaacgaccggcattaaatcgtatccgccattctcgatcggcagcgaa 660
pmxE_B-LR_Polymyxin-E        AGCACTTTGCCTGAAACGACCGGCTTTAAATCGTATCCGCCATTCTCGATCGGCAGCGAA 660
```

```
pmxE_KP26_Polymyxin-E        AGCACTTTGCCTGAAACGACCGGCTTTAAATCGTATCCGCCATTCTCGATCGGCAGCGAA 600
pmxE_Polymyxin-P             AACACTTTGCCTGAAACGACCGGCATTAAATCGTATCCGCCATTCTCGATCGGCAGCGAA 660
pmxE_Polymyxin-A             AACACTTTGCCTGAAACGACCGGCATTAAATCGTATCCGCCATTCTCGATCGGCAGCGAA 660
                             * ********************** ***********************************

pmxE-D-DaB3-Polymyxin-B      TCCAATAAACTGGCCATCACTTTGGACGGTTCCCGGTATGAACGCATTCTGGCCTTCAGC 720
pmxE_1G_Polymyxin            tccaataaactggccatcactttggacggttcccggtatgaacgcattctggcctttagc 720
pmxE_B-LR_Polymyxin-E        TCCAATAAACGGGCCACCACTTTGGACGGTTCCCGGTATGAACGCATTCTGGCCTTTAGC 720
pmxE_KP26_Polymyxin-E        TCCAATAAACGGGCCACCACTTTGGACGGTTCCCGGTATGAACGCATTCTGGCCTTTAGC 660
pmxE_Polymyxin-P             TCCAATAAACTGGCCATCACTTTGGACGGTTCCCGGTATGAACGCATTCTGGCCTTCAGC 720
pmxE_Polymyxin-A             TCCAATAAACTGTCCATCACTTTGGACGGTTCCCGGTATGAACGCATTCTGACCTTCAGC 720
                             ********** * *** ********************************** **** ***

pmxE-D-DaB3-Polymyxin-B      GAACAATATCAGGTCAGCTTATATACGTTATTTCTGTCCGCCATGTACGCCTTATTGTAC 780
pmxE_1G_Polymyxin            gaacaatatcaggtcagcttatatacgttatttctgtccgccatgtacgccttattgtac 780
pmxE_B-LR_Polymyxin-E        GAACAATATCAGGTCAGCTTATATACGTTATTTCTGTCCGCGATGTATGCCTTGCTATAC 780
pmxE_KP26_Polymyxin-E        GAACAATATCAGGTCAGCTTATATACGTTATTTCTGTCCGCGATGTATGCCTTGCTATAC 720
pmxE_Polymyxin-P             GAACAATATCAGGTCAGCTTATATACGTTATTTCTGTCCGCTATGTACGTCTTATTGTAC 780
pmxE_Polymyxin-A             GAACAATATCAGGTCAGCTTATATACGTTATTTCTGTCCGCCATGTACGCCTTATTGTAC 780
                             ***************************************** ***** * ***  * ***

pmxE-D-DaB3-Polymyxin-B      AAGCTGACCGACAGCACCGATGTTCCGGTCGGCACGGTTTTCGCCAATCGCACCAGCAAG 840
pmxE_1G_Polymyxin            aagctgaccgacagcaccgatgttccggtcggcacggttttcgccaatcgcaccagcaag 840
pmxE_B-LR_Polymyxin-E        AAGCTGACCGACAGCACCGATGTTCCGGTCGGCACGGTGTTCGCCAATCGCACCAGCAAG 840
pmxE_KP26_Polymyxin-E        AAGCTGACCGACAGCACCGATGTTCCGGTCGGCACGGTGTTCGCCAATCGCACCAGCAAG 780
pmxE_Polymyxin-P             AAGCTGACCGACAGCACCGATGTTCCGGTCGGCACGGTTTTCGCCAATCGCACCAGCAAG 840
pmxE_Polymyxin-A             AAGCTGACCGACAGCATCGATGTTCCGGTCGGCACGGTTTTCGCCAATCGCACCAGCAAG 840
                             **************** ********************* *********************

pmxE-D-DaB3-Polymyxin-B      AAGGAAAAAGAAACGATCGGCATGTTCGTCAGCACCGTGGCTACGCGGATTCGTCTGAAT 900
pmxE_1G_Polymyxin            aaggaaaaagaaacgatcggcatgttcgtcagcaccgtggctacgcggattcgtctgaat 900
pmxE_B-LR_Polymyxin-E        AAGGAAAAAGAAACGATCGGCATGTTCGTCAGCACCGTGGCTACACGGATTCATCTGAAT 900
pmxE_KP26_Polymyxin-E        AAGGAAAAAGAAACGATCGGCATGTTCGTCAGCACCGTGGCTACACGGATTCATCTGAAT 840
pmxE_Polymyxin-P             AAGGAAAAAGAAACGATCGGCATGTTCGTCAGCACCGTGGCTACGCGGATTCGTCTGAAT 900
pmxE_Polymyxin-A             AAGGAAAAAGAAACGATTGGCATGTTCGTCAGCACCGTGGCTACGCGGATTCGTCTGAAT 900
                             ***************** ************************** ******* *******

pmxE-D-DaB3-Polymyxin-B      CCAGACGGGCACGTGCTTTCCTTGATCCAAACGGTTTCCAAGGAAAATACGGCGGATCTG 960
pmxE_1G_Polymyxin            ccagacaggcacgtgctttccttgatccaaacggtttccaaggagaatacggcggatctg 960
pmxE_B-LR_Polymyxin-E        CCAGACGGGGACGTGCTTTCCTTGATCCAAGCGGTTTCCAAGGAGAATACGGCGGATCTG 960
pmxE_KP26_Polymyxin-E        CCAGACGGGGACGTGCTTTCCTTGATCCAAGCGGTTTCCAAGGAGAATACGGCGGATCTG 900
pmxE_Polymyxin-P             CCAGACAAGCACGTGCTTTCCTTGATCCAAACGGTTTCCAAGGAAAATACGGCGGATCTG 960
pmxE_Polymyxin-A             CCAGACAGGAACGTGCTTTCCTTGATCCAAACGGTTTCCAAGGAAAATACGGCGGATCTG 960
                             ******  * ******************** ************* ***************
```

```
pmxE-D-DaB3-Polymyxin-B      CGGTATCAAAAATACCCTTATAACCAATTGATCCAGGATTTACGCGAACAACACGGCCGC 1020
pmxE_1G_Polymyxin            aggtatcagaaatacccttataaccaattgatccaggatttacgcgaacaacacggccgc 1020
pmxE_B-LR_Polymyxin-E        CGGTATCAAAAATACCCTTATAACCAATTGATCCAGGATTTACGCGAACAACACGGCCGC 1020
pmxE_KP26_Polymyxin-E        CGGTATCAAAAATACCCTTATAACCAATTGATCCAGGATTTACGCGAACAACACGGCCGC 960
pmxE_Polymyxin-P             CGGTATCAGAAATACCCTTATAACCAATTGATCCAGGATTTACGCGAACAACACGGCCGC 1020
pmxE_Polymyxin-A             CGGTATCAGAAATACCCTTATAACCAATTGATCCAGGATTTACGTGAACAACACGGCCGC 1020
                              ******* *********************************** ***************

pmxE-D-DaB3-Polymyxin-B      AACGATCTTTCGGGGCTGTTCCGCACGTCTCTGGAATATCTGCCTTTGAAAATCGTGGAG 1080
pmxE_1G_Polymyxin            aacgatctttcggggctgttccgcacgtctctggaatatctgcctttgaaaatcgtggag 1080
pmxE_B-LR_Polymyxin-E        AACGATCTTTCGGGACTGTTCCGTACGTCTCTGGAATATCTGCCTTTGAAAATCGTGGAA 1080
pmxE_KP26_Polymyxin-E        AACGATCTTTCGGGACTGTTCCGTACGTCTCTGGAATATCTGCCTTTGAAAATCGTGGAA 1020
pmxE_Polymyxin-P             AACGATCTTTCGGGGCTGTTCCGTACGTCTCTAGAATATCTGCCTTTGAAAATCGTGGAG 1080
pmxE_Polymyxin-A             AACGATCTTTCGGGGCTGTTCCGCACGTCTCTGGAATATCTGCCTTTGAAAATCGTGGAG 1080
                             ************** ******** ******** ************************** 

pmxE-D-DaB3-Polymyxin-B      TACGAAGAAATCAAGGTACGCCTGGAGGCTCACTTCGCTAGGCATGAGATGGACGATTTG 1140
pmxE_1G_Polymyxin            tacgaagaaatcaaggtacgcctggaggctcacttcgctaggcacgagatggacgatttg 1140
pmxE_B-LR_Polymyxin-E        TACGAAGAAATCAAGGTACGCCTGGAGGCTCACTTCGCTAAGCACGAGATGGATGATTTG 1140
pmxE_KP26_Polymyxin-E        TACGAAGAAATCAAGGTACGCCTGGAGGCTCACTTCGCTAAGCACGAGATGGATGATTTG 1080
pmxE_Polymyxin-P             TACGAAGAAATCAAGGTACGCCTGGAGGCTCACTTCGCTAGGCACGAGATGGACGATTTG 1140
pmxE_Polymyxin-A             TACGAAGAAATCAAGGTACGCCTGGAGGCTCACTTTGCTAGGCACGAGATGGACGATTTG 1140
                             *********************************** **** *** ******** ******

pmxE-D-DaB3-Polymyxin-B      CTGCTGCGCTTCGACCATATGCTGAATGAAGGCCATGTAATTCTCCATGCTTCCTATCGT 1200
pmxE_1G_Polymyxin            ctgctgcgcttcgaccatatgctgaatgaaggccatgtaattctccatgcttcctatcgt 1200
pmxE_B-LR_Polymyxin-E        CTGCTGCGTTTCGACCATATGCTGAATGAAGGCCATGTCATTCTCCACGCTTCCTATCGC 1200
pmxE_KP26_Polymyxin-E        CTGCTGCGTTTCGACCATATGCTGAATGAAGGCCATGTCATTCTCCACGCTTCCTATCGC 1140
pmxE_Polymyxin-P             CTGCTGCGCTTCGATCATATGTTGAATGAAGGCCATGTAATTCTCCATGCTTCCTATCGT 1200
pmxE_Polymyxin-A             CTGCTGCGCTTCGACCATATGCTGAATGAAGGCCATGTCATTCTCCATGCTTCCTATCGT 1200
                             ******** ***** ****** **************** ******** *********** 

pmxE-D-DaB3-Polymyxin-B      ACCGGCCTGTTCGAGACGGCCGAGATTGATCGGATTATGGAACAGTATGTAACCGTTCTG 1260
pmxE_1G_Polymyxin            accggcctgttcgagacggccgagattgatcggattatggaacagtatgtaaccgttctg 1260
pmxE_B-LR_Polymyxin-E        ACTGGCTTGTTCGAGACAGCCGAGATTGATCGGATTATGGAACAGTATGTAACCGTTCTG 1260
pmxE_KP26_Polymyxin-E        ACTGGCTTGTTCGAGACAGCCGAGATTGATCGGATTATGGAACAGTATGTAACCGTTCTG 1200
pmxE_Polymyxin-P             ACCGGCCTGTTCGAGACGGCCGAGATTGATCGGATTATGGAACAGTATGTAACCGTTCTG 1260
pmxE_Polymyxin-A             ACCGGCTTGTTCGAGACGGCCGAGATTGATCGGATTATGGAACAGTATGTAACCGTTCTG 1260
                             ** *** ********** ******************************************

pmxE-D-DaB3-Polymyxin-B      GACCAGTTTCTTCAGACTCCCGAACTGCCGATACGCGAGATTTCTCTGCTGAGCGATGAG 1320
pmxE_1G_Polymyxin            gaccagtttcttcagactcccgaactgccggtacgcgagatttctctgctgagcgatgag 1320
pmxE_B-LR_Polymyxin-E        GACCAGTTTCTTCAGACTCCCGAACTGCCGGTACGCGAAATTTCTCTGCTGAGCGATGAG 1320
```

```
pmxE_KP26_Polymyxin-E        GACCAGTTTCTTCAGACTCCCGAACTGCCGGTACGCGAAATTTCTCTGCTGAGCGATGAG 1260
pmxE_Polymyxin-P             GACCAGTTTCTTCAGACTCCCGAACTGCCGGTACGCGAGATTTCTCTGCTGAGCGATGAG 1320
pmxE_Polymyxin-A             GACCAGTTTCTTCAGACTCCCGAACTGCCGATACGCGAGATTTCTCTGCTGAGCGATGAG 1320
                             ****************************** ******* *********************

pmxE-D-DaB3-Polymyxin-B      GAGAGACACCGCATTCTCAACGTTTTTAACCCGCCGGTGGCAGGGCTGAGCGAGGGAGAA 1380
pmxE_1G_Polymyxin            gagagacaccgcattctcaacgtttttaacccgccggtggcagggctgagcgagggagaa 1380
pmxE_B-LR_Polymyxin-E        GAGAGACAGTGCATTCTGGGCGTTTTTAACCCGCCGGTGGCAGGGCTGAGCGAGGGAGAG 1380
pmxE_KP26_Polymyxin-E        GAGAGACAGTGCATTCTGGGCGTTTTTAACCCGCCGGTGGCAGGGCTGAGCGAGGGAGAG 1320
pmxE_Polymyxin-P             GAGAGACACCGCATTCTCAACGTTTTTAACCCGCCGGTGGCAGGGCTTAGCGAGGGAGAA 1380
pmxE_Polymyxin-A             GAGAGACACCGCATTCTCAACGTTTTTAACCCGCCGGTGGCAGGGCTGAGCGAGGGAGAA 1380
                             ********  *******   *************************** ***********

pmxE-D-DaB3-Polymyxin-B      GCGTTTCATCGGTATGTTGAAAAGTTTGCCCGGGAAATTCCGGATCATCCGGCAGTCGTC 1440
pmxE_1G_Polymyxin            gcgtttcatcggtatgttgaaaagtttgcccgggaaattccggagcatccggcagtcgtc 1440
pmxE_B-LR_Polymyxin-E        GCGTTTCATCGGTACGTTGAAAAGTTTGCCCGCGAAATTCCAGATCATCCGGCAGTTGTC 1440
pmxE_KP26_Polymyxin-E        GCGTTTCATCGGTACGTTGAAAAGTTTGCCCGCGAAATTCCAGATCATCCGGCAGTTGTC 1380
pmxE_Polymyxin-P             GCGTTTCATCGGTATGTTGAAAAGTTTGCCCGGGAAATTCCAGATCATCCGGCAGTCGTC 1440
pmxE_Polymyxin-A             GCGTTTCATCGGTATGTTGAAAAGTTTGCTCGAGAAATTCCAGATCATCCGGCAGTCGTC 1440
                             ************** ************** ** ******** ** *********** ***

pmxE-D-DaB3-Polymyxin-B      TACATGGACAAAAAGCTGACCTACGGCGAATTGAACGAACGCGCCGAGCGGCTGGCTTCT 1500
pmxE_1G_Polymyxin            tacatggacaaacagctgacctacggcgaattgaacgaacgcgccgagcggctggcttct 1500
pmxE_B-LR_Polymyxin-E        TACATGGATACACAGCTGACCTACGGTGAATTGAACGAACGTGCCGAGCGGCTGGCTTCT 1500
pmxE_KP26_Polymyxin-E        TACATGGATACACAGCTGACCTACGGTGAATTGAACGAACGTGCCGAGCGGCTGGCTTCT 1440
pmxE_Polymyxin-P             TACATGGACAAACAGCTGACCTACGGCGAATTGAACGAACGCGCCGAGCGGCTGGCTTCT 1500
pmxE_Polymyxin-A             TACATGGACAAACAGCTGACCTACGGCGAATTGAACGAACGCGCCGAGCGGCTGGCTTCT 1500
                             ******** * * ************* ************** ******************

pmxE-D-DaB3-Polymyxin-B      CTCCTTCGCGAACAGGGCGTGGGAAGGGAGACGATTACGGGGATCTGGGCGGAGCGTTCG 1560
pmxE_1G_Polymyxin            ctccttcgcgaacagggcgtgggaagggagacgattacggggatctgggcggagcgttcg 1560
pmxE_B-LR_Polymyxin-E        CTCCTTCGCGAGCAGGGCGTGGGAAAGGAGACGATTACGGGGATCTGGGCGGAGCGTTCG 1560
pmxE_KP26_Polymyxin-E        CTCCTTCGCGAGCAGGGCGTGGGAAAGGAGACGATTACGGGGATCTGGGCGGAGCGTTCG 1500
pmxE_Polymyxin-P             CTCCTTCGCGAACAGGGCGTGGGAAGGGAGACGATTACGGGGATCTGGGCAGAGCGTTCG 1560
pmxE_Polymyxin-A             CTCCTTCGCGAACAGGGCGTGGGAAAGGAGACGATTACGGGGATCTGGGCGGAGCGTTCA 1560
                             *********** ************* ************************ ******** 

pmxE-D-DaB3-Polymyxin-B      GTGGAACTGCTGGTCGGGGTGCTCGCCGTTTGGAAAGCCGGCGGAGCCTATGTACCGCTG 1620
pmxE_1G_Polymyxin            gtggaactgctggtcggggtgctcgccgtttggaaagccggcggagcctatgtaccgctg 1620
pmxE_B-LR_Polymyxin-E        GTGGAACTGCTGATCGGGGTGCTCGCCGTTTGGAAAGCCGGCGGAGCCTATGTACCGCTT 1620
pmxE_KP26_Polymyxin-E        GTGGAACTGCTGATCGGGGTGCTCGCCGTTTGGAAAGCCGGCGGAGCCTATGTACCGCTT 1560
pmxE_Polymyxin-P             GTGGAACTGCTGGTCGGGGTGCTCGCCGTTTGGAAAGCCGGCGGAGCCTATGTACCGCTG 1620
pmxE_Polymyxin-A             GTGGAACTGCTGGTCGGGGTGCTCGCCGTTTGGAAAGCCGGCGGAGCCTATGTACCGCTG 1620
                             ************ ********************************************** 
```

```
pmxE-D-DaB3-Polymyxin-B      GACCCTGATTATCCGGCGGAGCGGATTGAGTACATGCTCAGCGATAGCGATGCATCGGTG 1680
pmxE_1G_Polymyxin            gaccccgattatccggcggagcggattgagtacatgctcagcgatagcgatgcatcggtg 1680
pmxE_B-LR_Polymyxin-E        GACCCCGATTATCCGGCGGAGCGGATTGAGTACATGCTCAGCGATAGCGGTGCGTCGGTG 1680
pmxE_KP26_Polymyxin-E        GACCCCGATTATCCGGCGGAGCGGATTGAGTACATGCTCAGCGATAGCGGTGCGTCGGTG 1620
pmxE_Polymyxin-P             GACCCCGATTATCCGGCGGAGCGGATTGAGTACATGCTCAGCGATAGCGATGCATCGGTG 1680
pmxE_Polymyxin-A             GACCCCGATTATCCGGCGGAGCGGATTGAGTACATGCTCAGCGATAGCGATGCATCGGTG 1680
                             ***** ******************************************* *** ******

pmxE-D-DaB3-Polymyxin-B      CTGCTTACGCAGCGTCATCTGTTGGAGCGGACCAGAGGTTGGTTGGCCGATGACCGGCTG 1740
pmxE_1G_Polymyxin            ctgcttacgcagcgtcatctgttggagcgggccggaggttggttggccgatgaccggctg 1740
pmxE_B-LR_Polymyxin-E        CTGCTTACGCAGCGTCATCTGTTGGAGCGGGCCGGAGGTTGGTTGGCCGATGACCGGCTG 1740
pmxE_KP26_Polymyxin-E        CTGCTTACGCAGCGTCATCTGTTGGAGCGGGCCGGAGGTTGGTTGGCCGATGACCGGCTG 1680
pmxE_Polymyxin-P             CTGCTTACGCAGCGTCATCTGTTGGAGCGGGCCAGAGGTTGGTTGGCCGATGACCGGCTG 1740
pmxE_Polymyxin-A             CTGCTTACGCAGCGTCATCTGTTGGAGCGGGCCGGAGGTTGGTTGGCCGATGACCGGCTG 1740
                             ****************************** ** **************************

pmxE-D-DaB3-Polymyxin-B      AAGCTTCAAGCTGTCTATGCCATGGACGATGAACAGATTTATAACGGGGATGCCTTAGCC 1800
pmxE_1G_Polymyxin            aagcttcaagctgtctatgccatggacgatgaacagatttataacggggatgccttagcc 1800
pmxE_B-LR_Polymyxin-E        AAACTACAAGCTGTCTATGCTATGGACGATGAACAGATTTATAACAGGGATGCCTTAGCC 1800
pmxE_KP26_Polymyxin-E        AAACTACAAGCTGTCTATGCTATGGACGATGAACAGATTTATAACAGGGATGCCTTAGCC 1740
pmxE_Polymyxin-P             AAGCTTCAAGCTGTCTATGCCATGGACGATGAACAGATTTATAACGGGGATGCCTTAGCC 1800
pmxE_Polymyxin-A             AAGCTTCAAGCTGTCTATGCCATGGACGATGAACAGATTTATAACGGGGATGCCTTAGCC 1800
                             ** ** ************** ************************ **************

pmxE-D-DaB3-Polymyxin-B      GTGGAATTTGAATCTGCCGGCAGCGCCCCGCAAGACTTGGCTTATGTGATTTACACCTCG 1860
pmxE_1G_Polymyxin            gtggaatttgaatctgccggcagcgccctgcaagacttggcttatgtgatttacacctcg 1860
pmxE_B-LR_Polymyxin-E        GTGGAATTTGAGTCTGCCGATAGTGCCCCGCAAGACTTGGCTTATGTGATTTACACCTCG 1860
pmxE_KP26_Polymyxin-E        GTGGAATTTGAGTCTGCCGATAGTGCCCCGCAAGACTTGGCTTATGTGATTTACACCTCG 1800
pmxE_Polymyxin-P             GTGGAATTTGAATCTGCCGGCAGCGCCCCGCAAGACTTGGCTTATGTGATTTACACCTCG 1860
pmxE_Polymyxin-A             GTGGAATTTGAATCTGCCGGCAGCGCCCCGCAAGACTTGGCTTATGTGATTTACACCTCG 1860
                             *********** *******  ** **** *******************************

pmxE-D-DaB3-Polymyxin-B      GGTACGACGGGACGCCCGAAAGGCGTCATGATCGAGCACGGTAGTCTCGTGAATACGGCG 1920
pmxE_1G_Polymyxin            ggtacgacgggacgcccgaaaggcgtcatgatcgagcacggtagtctcgtgaatacggcg 1920
pmxE_B-LR_Polymyxin-E        GGTACGACGGGACGCCCGAAAGGCGTCATGATCGAACATGGTAGTCTCTTGAATACGGCG 1920
pmxE_KP26_Polymyxin-E        GGTACGACGGGACGCCCGAAAGGCGTCATGATCGAACATGGTAGTCTCTTGAATACGGCG 1860
pmxE_Polymyxin-P             GGTACGACGGGACGCCCGAAAGGCGTCATGATCGAGCACGGTAGTCTCGTGAATACGGCG 1920
pmxE_Polymyxin-A             GGTACGACGGGACGCCCGAAAGGTGTCATGATCGAGCATGGTAGTCTCGTGAATACGGCG 1920
                             *********************** *********** ** ********* ***********

pmxE-D-DaB3-Polymyxin-B      GATGCGTACCGTCGCGAGTACCAGTTGGATCAGTTTCCGGTGCGGCTGCTGCAGCTGGCC 1980
pmxE_1G_Polymyxin            gatgcgtaccgtcgcgagtaccggttggatcagtttccggtgcggctgctgcagttggcc 1980
pmxE_B-LR_Polymyxin-E        GATGCGTACCGTCGCGAGTACCGGTTGGATCAGTTCCCGGTGCGGCTGCTGCAGTTGGCC 1980
```

```
pmxE_KP26_Polymyxin-E        GATGCGTACCGTCGCGAGTACCGGTTGGATCAGTTCCCGGTGCGGCTGCTGCAGTTGGCC 1920
pmxE_Polymyxin-P             GATGCGTACCGTCGCGAGTACCGGTTGGATCAGTTTCCGGTGCGGCTGCTGCAGCTGGCC 1980
pmxE_Polymyxin-A             GATGCGTACCGTCGCGAGTACCGGTTGGATCAGTTTCCGGTGCGGCTGCTGCAGCTGGCC 1980
                             ********************** ************ ****************** *****

pmxE-D-DaB3-Polymyxin-B      AGCTTCTCGTTTGACGTGTTCGTCGGAGACATCGCGCGGACGCTGTATAACGGAGGCACG 2040
pmxE_1G_Polymyxin            agcttctcgtttgacgtgttcgtcggagacatcgcgcggacgctgtataacggaggcacg 2040
pmxE_B-LR_Polymyxin-E        AGCTTTTCGTTTGACGTGTTTGTCGGAGACATCGCTCGGACGCTGTATAACGGAGGTACG 2040
pmxE_KP26_Polymyxin-E        AGCTTTTCGTTTGACGTGTTTGTCGGAGACATCGCTCGGACGCTGTATAACGGAGGTACG 1980
pmxE_Polymyxin-P             AGCTTCTCGTTTGACGTGTTCGTCGGAGACATCGCGCGGACGCTGTATAACGGAGGCACG 2040
pmxE_Polymyxin-A             AGCTTCTCGTTTGACGTGTTCGTCGGAGACATCGCGCGGACGCTGTATAACGGAGGCACG 2040
                             ***** ************** ************** ******************** ***

pmxE-D-DaB3-Polymyxin-B      ATGGTGATTGTGCCGAAGGATGACCGGATTGATCCGAACCTCTTATACGGCTGGATTCGG 2100
pmxE_1G_Polymyxin            atggtgattgtgccgaaggatgaccggattgatccgaacctcttgtacggctggattcgg 2100
pmxE_B-LR_Polymyxin-E        ATGGTGATTGTGCCAAAGGATGACCGGATTGATCCGAACCGCTTATACGGCTGGATTCGG 2100
pmxE_KP26_Polymyxin-E        ATGGTGATTGTGCCAAAGGATGACCGGATTGATCCGAACCGCTTATACGGCTGGATTCGG 2040
pmxE_Polymyxin-P             ATGGTGATTGTGCCGAAGGATGACCGGATTGATCCGAACCTCTTATACGGCTGGATTCGG 2100
pmxE_Polymyxin-A             ATGGTGATTGTGCCGAAGGATGACCGGATTGATCCGAACCTCTTATACGGCTGGATTCGG 2100
                             ************** ************************* *** ***************

pmxE-D-DaB3-Polymyxin-B      GACCAAAACATTACGGTATTCGAATCGACGCCTGCGCTCATCCTACCGTTCATGCAGCAT 2160
pmxE_1G_Polymyxin            gaccaaaacattacggtattcgaatcgacgcctgcgctcatcctgccgttcatgcagcat 2160
pmxE_B-LR_Polymyxin-E        GACCAAAACATTACGGTATTCGAATCGACGCCTGCGCTCATCCTGCCATTCATGCAGCAT 2160
pmxE_KP26_Polymyxin-E        GACCAAAACATTACGGTATTCGAATCGACGCCTGCGCTCATCCTGCCATTCATGCAGCAT 2100
pmxE_Polymyxin-P             GACCAAAACATTACGGTATTCGAATCGACGCCTGCGCTCATCCTGCCGTTCATGCAGCAT 2160
pmxE_Polymyxin-A             GACCAAAACATTACGGTATTCGAATCGACGCCTGCGCTCATCCTGCCGTTCATGCAGCAT 2160
                             ******************************************** ** ************

pmxE-D-DaB3-Polymyxin-B      ATTTATGAAGAAGGGCTGGACGTTAGCTCCATGCAGTTGCTGATTACCAGCTCGGATGCT 2220
pmxE_1G_Polymyxin            atttatgaagaagggctggacgttagctccatgcagttgctgattaccagctcggatgct 2220
pmxE_B-LR_Polymyxin-E        ATTTATGAAGAAGGGCTGGACGTTAGCTCCATGCAGTTGCTGATTACCAGCTCGGATGCT 2220
pmxE_KP26_Polymyxin-E        ATTTATGAAGAAGGGCTGGACGTTAGCTCCATGCAGTTGCTGATTACCAGCTCGGATGCT 2160
pmxE_Polymyxin-P             ATTTATGAAGAAGGGCTGGACGTTAGCTCCATGCAGTTGCTGATTACCAGCTCGGATGCT 2220
pmxE_Polymyxin-A             ATTTATGAAGAAGGGCTGGACGTTAGCTCCATGCAGTTGCTGATTACCAGCTCGGATGCT 2220
                             ************************************************************

pmxE-D-DaB3-Polymyxin-B      TGCAGTGTCACCGATTACCGATTGCTGCAGGAAAGATTCGGCGGACAATTCCGCATCATC 2280
pmxE_1G_Polymyxin            tgcagtgtcaccgattaccgattgctgcaggaaagattcggcggacaattccgcatcatc 2280
pmxE_B-LR_Polymyxin-E        TGCAGTGTCACCGATTACCGATTGCTGCAGGAAAGATTCGGCGGACAATTCCGCATCATC 2280
pmxE_KP26_Polymyxin-E        TGCAGTGTCACCGATTACCGATTGCTGCAGGAAAGATTCGGCGGACAATTCCGCATCATC 2220
pmxE_Polymyxin-P             TGCAGTGTCACCGATTACCGATTGCTGCAGGAAAGATTCGGCGGACAATTCCGCATCATC 2280
pmxE_Polymyxin-A             TGCAGTGTCACCGATTACCGATTGCTGCAGGAAAGATTCGGCGGACAATTCCGCATCATC 2280
                             ************************************************************
```

```
pmxE-D-DaB3-Polymyxin-B      AACAGCTATGGCGTTACCGAAGCGGCCATTGACAGCAGCTTTTACGGTGAGCCGCTGGAT 2340
pmxE_1G_Polymyxin            aacagctatggcgttaccgaagcggccattgacagcagcttttacggtgagccgctggat 2340
pmxE_B-LR_Polymyxin-E        AACAGCTATGGCGTTACCGAAGCGGCCATTGACAGCAGCTTTTACGATGAGACGCTGGAC 2340
pmxE_KP26_Polymyxin-E        AACAGCTATGGCGTTACCGAAGCGGCCATTGACAGCAGCTTTTACGATGAGACGCTGGAC 2280
pmxE_Polymyxin-P             AACAGCTATGGCGTTACCGAAGCGGCCATTGACAGCAGCTTTTACGGTGAGCCGCTGGAT 2340
pmxE_Polymyxin-A             AACAGCTATGGCGTTACCGAAGCGGCCATTGACAGCAGCTTTTACGGTGAGCCGCTGGAT 2340
                             ********************************************** **** ******* 

pmxE-D-DaB3-Polymyxin-B      AAGCTGCCGCCGTCGGGTCATGTGCCGATCGGCAAAGCTTGGCTGAACGCCCGGTTTTAC 2400
pmxE_1G_Polymyxin            aagctgccgccgtcgggtcatgtgccgatcggcaaagcttggctgaacgcccggttttac 2400
pmxE_B-LR_Polymyxin-E        AAGCTGCCGTCGTCGGGTCATGTGCCGATCGGCAAAGCTTGGCTGAACGCCCGGTTTTAC 2400
pmxE_KP26_Polymyxin-E        AAGCTGCCGTCGTCGGGTCATGTGCCGATCGGCAAAGCTTGGCTGAACGCCCGGTTTTAC 2340
pmxE_Polymyxin-P             AAGCTGCCGCCGTCAGGTCATGTGCCGATCGGCAAAGCTTGGCTAAACGCCCGGTTTTAC 2400
pmxE_Polymyxin-A             AAGCTGCCGCCGTCGGGTCATGTGCCGATCGGCAAAGCTTGGCTGAACGCCCGGTTTTAC 2400
                             ********* **** ***************************** ***************

pmxE-D-DaB3-Polymyxin-B      ATTGTCGATGCCGCGTTAAAGCCGGTTCCTGTAGGGGTTCCGGGCGAGCTTGTCATCGGC 2460
pmxE_1G_Polymyxin            attgtcgatgccgcgttaaagccggttcctgtaggggttccgggcgagcttgtcatcggc 2460
pmxE_B-LR_Polymyxin-E        ATTGTCGATGCCGCGTTAAAACCGGTTCCTGTAGGGGTTCCGGGCGAGCTTGTCATCGGA 2460
pmxE_KP26_Polymyxin-E        ATTGTCGATGCCGCGTTAAAACCGGTTCCTGTAGGGGTTCCGGGCGAGCTTGTCATCGGA 2400
pmxE_Polymyxin-P             ATTGTCGATGCCGCATTAAAGCCGGTTCCTGTAGGGGTTCCGGGCGAGCTTGTCATCGGC 2460
pmxE_Polymyxin-A             ATTGTCGATGCCGCGTTAAAGCCGGTTCCTGTAGGGGTTCCGGGCGAGCTTGTCATCGGC 2460
                             ************** ***** ************************************** 

pmxE-D-DaB3-Polymyxin-B      GGCGCCGGGGTGGCGCGCGGGTACTGGAACCGTCCGGACCTAACCGCCGAGAAGTTTGCG 2520
pmxE_1G_Polymyxin            ggcgccggggtggcgcgcgggtactggaaccgtccggacctaaccgccgagaagtttgca 2520
pmxE_B-LR_Polymyxin-E        GGCGCTGGGGTGGCGCGCGGATACTGGAACCGTCCGGACCTAACGGCCGAGAAGTTTGCG 2520
pmxE_KP26_Polymyxin-E        GGCGCTGGGGTGGCGCGCGGATACTGGAACCGTCCGGACCTAACGGCCGAGAAGTTTGCG 2460
pmxE_Polymyxin-P             GGCGCCGGGGTGGCGCGCGGGTACTGGAACCGTCCGGACCTAACCGCCGAGAAGTTTGTG 2520
pmxE_Polymyxin-A             GGCGCCGGGGTGGCGCGCGGGTACTGGAACCGTCCAGACCTAACCGCCGAGAAGTTTGCG 2520
                             ***** ************** ************** ******** *************  

pmxE-D-DaB3-Polymyxin-B      GACAGCCCGTTTGTACCGGGCGAACGTCTGTACCGGACAGGCGATTTGGCCCGCTGGCTG 2580
pmxE_1G_Polymyxin            gacagcccgtttgtgccgggcgaacgtctgtaccggacaggcgatttggcccgctggctg 2580
pmxE_B-LR_Polymyxin-E        GACAGCCCGTTTGTGCCTGGCGAACGTTTGTATCGGACAGGCGATTTGGCCCGCTGGCTG 2580
pmxE_KP26_Polymyxin-E        GACAGCCCGTTTGTGCCTGGCGAACGTTTGTATCGGACAGGCGATTTGGCCCGCTGGCTG 2520
pmxE_Polymyxin-P             GACAGCCCGTTTGTGCCGGGCGAACGTCTGTACCGGACAGGCGATTTGGCCCGCTGGCTG 2580
pmxE_Polymyxin-A             GACAGCCCGTTTGTGCCGGGCGAACGTCTGTACCGGACAGGCGATTTGGCCCGCTGGCTG 2580
                             ************** ** ********* **** ***************************

pmxE-D-DaB3-Polymyxin-B      GAAGACGGCAACGTCGACTTCATCGGCCGGATCGACTATCAGGTGAAAATTCGCGGGTTC 2640
pmxE_1G_Polymyxin            aaagacggcaacgtcgacttcatcggccggatcgactatcaggtgaaaattcgcgggttc 2640
pmxE_B-LR_Polymyxin-E        GAAGACGGCAACGTCGACTTCATCGGCCGAATTGACTATCAGGTGAAAATTCGCGGGTTC 2640
```

```
pmxE_KP26_Polymyxin-E        GAAGACGGCAACGTCGACTTCATCGGCCGAATTGACTATCAGGTGAAAATTCGCGGGTTC 2580
pmxE_Polymyxin-P             GAAGACGGCAACGTCGACTTCATCGGCCGGATCGACTATCAGGTGAAAATTCGCGGGTTC 2640
pmxE_Polymyxin-A             GAAGACGGCAACGTCGACTTCATCGGCCGGATCGACTATCAGGTGAAAATTCGCGGGTTC 2640
                              **************************** ** ***************************

pmxE-D-DaB3-Polymyxin-B      CGGATCGAACTCGGCGAAATTGAAACGGCCCTGCTGCGTTTCCCTGGCGTCAAGCAGGCT 2700
pmxE_1G_Polymyxin            cggatcgaactcggcgaaattgaaacgaccctgctgcgtttcccgggcgtcaagcaggct 2700
pmxE_B-LR_Polymyxin-E        CGGATCGAACTCGGCGAAATTGAAACGGCCTTGCTGCGTTTCCCGGGCGTCAAGCAGGCT 2700
pmxE_KP26_Polymyxin-E        CGGATCGAACTCGGCGAAATTGAAACGGCCTTGCTGCGTTTCCCGGGCGTCAAGCAGGCT 2640
pmxE_Polymyxin-P             CGGATCGAACTCGGCGAAATTGAAACGGCCCTGCTGCGTTTTCCGGGCGTCAAGCAGGCT 2700
pmxE_Polymyxin-A             CGGATCGAACTCGGCGAAATTGAAACGGCCCTGCTGCGTTTCCCGGGCGTCAAGCAGGCT 2700
                             *************************** ** ********** ** ***************

pmxE-D-DaB3-Polymyxin-B      GTGGTGACCGACCGTACGGATGAGCAGGGGCAAAAGTATTTGTGCGGCTACGTGGCGGCG 2760
pmxE_1G_Polymyxin            gtggtgaccgaccgtacggatgagcaggggcaaaagtatttgtgcggctacgtggcggcg 2760
pmxE_B-LR_Polymyxin-E        GTGGTGACAGACCGTACAGATAAGCAGGGGCAAAAGTATTTGTGTGGCTACGTGGCGGCA 2760
pmxE_KP26_Polymyxin-E        GTGGTGACAGACCGTACAGATAAGCAGGGGCAAAAGTATTTGTGTGGCTACGTGGCGGCA 2700
pmxE_Polymyxin-P             GTGGTGACCGACCGTACGGATGAGCAGGGGCAGAAGTATTTGTGCGGCTACGTGACGGCG 2760
pmxE_Polymyxin-A             GTGGTGACCGACCGTACGGATGAGCAGGGGGAAAAGTATTTGTGCGGCTACGTGGCGGCG 2760
                             ******** ******** *** ******** * *********** ********* **** 

pmxE-D-DaB3-Polymyxin-B      GATGCTTCCTTGCAGCTGAGCGATCTGCTGTCCCAATTGAAGCAAGAGCTGCCGGCCCAT 2820
pmxE_1G_Polymyxin            gatgcttccttgcagctgagcgatctgctgtcccaattgaagcaagagctgccggcccat 2820
pmxE_B-LR_Polymyxin-E        GATACTTCCTTGCAGCTGAGCGATCTGCTGTCCCAATTGAAGCAAGAGCTGCCGGCCCAT 2820
pmxE_KP26_Polymyxin-E        GATACTTCCTTGCAGCTGAGCGATCTGCTGTCCCAATTGAAGCAAGAGCTGCCGGCCCAT 2760
pmxE_Polymyxin-P             GATGCTTCCTTGCAGCTGAGCGATCTGCTGTCCCAATTGAAGCAAGAGCTGCCGGCCCAT 2820
pmxE_Polymyxin-A             GATGCTTCCTTGCAGCTGAGCGATTTGCTGTCCCAATTGAAGCAAGAGCTGCCGGCCCAT 2820
                             *** ******************** ***********************************

pmxE-D-DaB3-Polymyxin-B      ATGGTCCCGGCCCGGCTGGTGTCTCTTGATAAGCTTCCGCTCACGCCGAACGGCAAAATT 2880
pmxE_1G_Polymyxin            atggtcccggcccggctggtgtctcttgataagcttccgctcacgccgaacggcaaaatt 2880
pmxE_B-LR_Polymyxin-E        ATGGTTCCGGCCCGGCTGGTGTCTCTTGATAAGCTTCCACTTACTCCGAACGGCAAAATT 2880
pmxE_KP26_Polymyxin-E        ATGGTTCCGGCCCGGCTGGTGTCTCTTGATAAGCTTCCACTTACTCCGAACGGCAAAATT 2820
pmxE_Polymyxin-P             ATGGTCCCGGCCCGGTTGGTGTCTCTTGATAAGCTTCCGCTCACGCCGAACGGCAAAATT 2880
pmxE_Polymyxin-A             ATGGTCCCGGCCCGGCTGGTGTCTCTTGATAAGCTTCCGCTCACGCCGAACGGCAAAATT 2880
                             ***** ********* ********************** ** ** ***************

pmxE-D-DaB3-Polymyxin-B      GACCGTAAAGCGCTGCCTGAACCGACCGGAGAGGTAGAAGCAGGCCGTGAGTATGTGGCT 2940
pmxE_1G_Polymyxin            gaccgtaaagcgctgcctgaaccgaccggagaggtagaagcaggccgtgagtatgtggct 2940
pmxE_B-LR_Polymyxin-E        GACCGTAAAGCGCTGCCTGAACCGACCGGAGAGGTAGAAGCAGGCCGTGAGTATGTGGCT 2940
pmxE_KP26_Polymyxin-E        GACCGTAAAGCGCTGCCTGAACCGACCGGAGAGGTAGAAGCAGGCCGTGAGTATGTGGCT 2880
pmxE_Polymyxin-P             GACCGTAAAGCGCTGCCTGAACCGACCGGAGAGGTAGAAGCAGACCGTGAGTATGTGGCT 2940
pmxE_Polymyxin-A             GACCGTAAAGCGCTGCCTGAACCGACCGGAGAGGTAGAAGCAGGCCGTGAGTATGTGGCT 2940
                             ******************************************* ****************
```

```
pmxE-D-DaB3-Polymyxin-B      CCTCGCACAACGCTGGAAACAAGACTTGCTCTCATTTGGCAGCAGGTGCTGGGTATTGCG 3000
pmxE_1G_Polymyxin            cctcgcacaacgctggaaacaagacttgctctcatttggcagcaggtgctgggtattgcg 3000
pmxE_B-LR_Polymyxin-E        CCTCGCACAACGCTGGAAACAAGACTTGCTCTCATTTGGCAGCAGGTGCTGGGTATTGCG 3000
pmxE_KP26_Polymyxin-E        CCTCGCACAACGCTGGAAACAAGACTTGCTCTCATTTGGCAGCAGGTGCTGGGTATTGCG 2940
pmxE_Polymyxin-P             CCTCGCACAACGCTTGAAACAAGACTTGCTCTCATTTGGCAGCAGGTGCTGGGTATTGCG 3000
pmxE_Polymyxin-A             CCTCGCACAACGCTGGAAACAAGACTTGCTCTCATTTGGCAGCAGGTGCTGGGTATTGCG 3000
                             ************** *********************************************

pmxE-D-DaB3-Polymyxin-B      CGAGTTGGAGTCCAAGACGATTTCTTTGACTTGGGCGGTCATTCCTTGCGGGCCTCCACG 3060
pmxE_1G_Polymyxin            cgagttggagtccaagacgatttctttgacttgggtggtcattccttgcgggcctccacg 3060
pmxE_B-LR_Polymyxin-E        CGAGTTGGAGTCGAAGACGATTTCTTTGACCTGGGTGGTCATTCCTTGCGGGCCTCCACG 3060
pmxE_KP26_Polymyxin-E        CGAGTTGGAGTCGAAGACGATTTCTTTGACCTGGGTGGTCATTCCTTGCGGGCCTCCACG 3000
pmxE_Polymyxin-P             CGAGTTGGAGTCCAAGACGATTTCTTTGACTTGGGTGGTCATTCCTTGCGGGCCTCCACG 3060
pmxE_Polymyxin-A             CGAGTTGGAGTCCAAGACGATTTTTTTGACTTGGGTGGTCATTCCTTGCGGGCCTCCACG 3060
                             ************ ********** ****** **** ************************

pmxE-D-DaB3-Polymyxin-B      CTTGTTTCCAAGATTCGGAAAGAGCTGCAAGTCGAGGTTCCGCTGCGGGAAGTTTTCCGC 3120
pmxE_1G_Polymyxin            cttgtatccaagattcggaaagagctgcaagtcgaggttccgttgcgggaagttttccgc 3120
pmxE_B-LR_Polymyxin-E        CTGGTTTCCAAGATTCGGAAAGAGCTGCAAGTCGAGGTTCCGCTGCGGGACGTTTTCCGC 3120
pmxE_KP26_Polymyxin-E        CTGGTTTCCAAGATTCGGAAAGAGCTGCAAGTCGAGGTTCCGCTGCGGGACGTTTTCCGC 3060
pmxE_Polymyxin-P             CTGGTTTCCAAGATTCGGAAAGAGCTGCAAGTCGAGGTTCCGCTGCGGGAAGTTTTCCGC 3120
pmxE_Polymyxin-A             CTGGTTTCCAAGATTCGGAAAGAGCTGCAAGTCGAGGTTCCGCTGCGCGAAGTTTTCCGC 3120
                             ** ** ************************************ **** ** *********

pmxE-D-DaB3-Polymyxin-B      TACACCACGATCGAACAGCTGGCCCAAAGAATCGGCGGTTTAAGGCAGCAGGAGACGTAT 3180
pmxE_1G_Polymyxin            tacaccacgatcgaacagctggcccaaagaatcagcggtttaaggcagcaggagacgtat 3180
pmxE_B-LR_Polymyxin-E        TACACCACGATCAAACAGCTGGCCCAAAGAATCGGCGGTTTAAAGCAGCAGGAGACGTAT 3180
pmxE_KP26_Polymyxin-E        TACACCACGATCAAACAGCTGGCCCAAAGAATCGGCGGTTTAAAGCAGCAGGAGACGTAT 3120
pmxE_Polymyxin-P             TACACCACGATCGAACAGCTGGCCCAAAGAATCGGCGGTTTAAGGCAGCAGGAGACGTAT 3180
pmxE_Polymyxin-A             TACACCACGATCGAACAGCTGGCCCAAAGAATCGGCGGTTTAAGGCAGCAGGAGACGTAT 3180
                             ************ ******************** ********* ****************

pmxE-D-DaB3-Polymyxin-B      GAGATTACAAAGGCGGCTGAGGCCGAGTACTATCCGGTTTCATCCGAGCAAAAGCGTCTG 3240
pmxE_1G_Polymyxin            gagatcacaaaggcggctgaggccgagtactatccggtttcatccgagcaaaagcgtctg 3240
pmxE_B-LR_Polymyxin-E        GAAATTACAAAGGCGGCTGAGGCCGAGTACTATCCGGTTTCATCCGAGCAAAAGCGTCTG 3240
pmxE_KP26_Polymyxin-E        GAAATTACAAAGGCGGCTGAGGCCGAGTACTATCCGGTTTCATCCGAGCAAAAGCGTCTG 3180
pmxE_Polymyxin-P             GAGATTACAAAGGCGGCTGAGGCTGAGTACTATCCGGTTTCATCCGAGCAAAAGCGTCTG 3240
pmxE_Polymyxin-A             GAGATTACAAAGGCGGCTGAGGCCGAGTACTATCCGGTTTCATCCGAGCAAAAGCGTCTG 3240
                             ** ** ***************** ************************************

pmxE-D-DaB3-Polymyxin-B      TACGTCCTGCGCCAGCTTGACGGGGCCGAGCGCAGCTACAATATGTCGGCGGCGCTTCTT 3300
pmxE_1G_Polymyxin            tacgtcctgcgccagcttgacggggccgagcgcagctacaatatgtcggcggcgcttctt 3300
pmxE_B-LR_Polymyxin-E        TACGTCCTGCGCCAGCTTGACGGGGCCGAGCGCAGCTACAATATGTCGGCGGCGCTTCTT 3300
```

```
pmxE_KP26_Polymyxin-E        TACGTCCTGCGCCAGCTTGACGGGGCCGAGCGCAGCTACAATATGTCGGCGGCGCTTCTT 3240
pmxE_Polymyxin-P             TACATCCTACGCCAGCTTGACGGGGCCGAGCGCAGCTACAATATGTCGGCGGCGCTTCTT 3300
pmxE_Polymyxin-A             TACGTCCTGCGCCAGCTTGACGGGGCCGAGCGCAGCTACAATATGTCGGCGGCGCTTCTT 3300
                             *** **** ***************************************************

pmxE-D-DaB3-Polymyxin-B      CTCGAAGGCAAGCTGGACCGCACGCGCGTCGAGCACGCGTTCCGGGCGCTGATTCAGCGC 3360
pmxE_1G_Polymyxin            ctcgaaggcaagctggaccgcacgcgcgtcgagcacgcgttccgggcgctgattcagcgc 3360
pmxE_B-LR_Polymyxin-E        CTCGAAGGCAAGCTGGATCGGACGCGCGTAGAGTACGCGTTCCGGGCGCTGATTCAGCGT 3360
pmxE_KP26_Polymyxin-E        CTCGAAGGCAAGCTGGATCGGACGCGCGTAGAGTACGCGTTCCGGGCGCTGATTCAGCGT 3300
pmxE_Polymyxin-P             CTCGAAGGCAAGCTGGACCGCACGCGCGTCGAGCACGCGTTCCGGGCGCTGATTCAGCGC 3360
pmxE_Polymyxin-A             CTCGAAGGCAAGCTGGACCGCATGCGCGTCGAGCACGCGTTCCGGGCGCTGATTCAGCGC 3360
                             ***************** ** * ****** *** *************************

pmxE-D-DaB3-Polymyxin-B      CATGAGACGCTGCGTACCGGGATCGAGCAGGTTCAAGGCGAGCTTGTCCAGCGCATCTAT 3420
pmxE_1G_Polymyxin            catgagacgctgcgtaccgggatcgagcaggttcaaggcgagcttgtccagcgcatctat 3420
pmxE_B-LR_Polymyxin-E        CATGAGACGCTGCGTACCGGGATCGAGCAGGTTCAAGGCGAACTTGTCCAGCGCATCTAT 3420
pmxE_KP26_Polymyxin-E        CATGAGACGCTGCGTACCGGGATCGAGCAGGTTCAAGGCGAACTTGTCCAGCGCATCTAT 3360
pmxE_Polymyxin-P             CATGAGACGCTGCGTACCGGGATCGAGCAGGTTCAAGGCGAGCTTGTTCAGCGCATCTAT 3420
pmxE_Polymyxin-A             CATGAGACGCTGCGTACGGGAATCGAGCAGGTTCAAGGCGAGCTTGTCCAGCGCATCTAT 3420
                             ***************** ** ******************** ***** ************

pmxE-D-DaB3-Polymyxin-B      GACGAGGTGGAGTTTGCTGTCGATTATTTCCAAGCGAGCGAGCGGGAAGTGGAGCAAGTG 3480
pmxE_1G_Polymyxin            gacgaggtggagttttctgtcgattatttccaggcgagcgagcgggaagtggaccaagtg 3480
pmxE_B-LR_Polymyxin-E        GACGAGGTGGAGTTTGCTGTGGATTATTTCCAGGCGAGTGAGCGGGAAGTGGAGCAAGGG 3480
pmxE_KP26_Polymyxin-E        GACGAGGTGGAGTTTGCTGTGGATTATTTCCAGGCGAGTGAGCGGGAAGTGGAGCAAGGG 3420
pmxE_Polymyxin-P             GATGAGGTGGAGTTTGCTGTCGATTATTTCCAGGCGAGCGAGCGGGAAGTGGACCAAGTG 3480
pmxE_Polymyxin-A             GACGAGGTCGAGTTTGCTGTGGATTATTTCCAGGCGAGCGAGCGGGAAGTGGAGCAAGTG 3480
                             ** ***** ****** **** *********** ***** ************** **** *

pmxE-D-DaB3-Polymyxin-B      GTGGAAGCTTACTATCACCCGTTTGATCTGACCAAGCCGCCACTTCTCCGCATCGGCCTG 3540
pmxE_1G_Polymyxin            gtggaagcttactattatccgtttgatgtgaccaagccgccacttctccgcatcggcctg 3540
pmxE_B-LR_Polymyxin-E        GTGGAAGCTTACTATCGCCCGTTTGATCTGACCAAGCCGCCACTTCTCCGCATCGGCCTG 3540
pmxE_KP26_Polymyxin-E        GTGGAAGCTTACTATCGCCCGTTTGATCTGACCAAGCCGCCACTTCTCCGCATCGGCCTG 3480
pmxE_Polymyxin-P             GTGGAAGCTTACTATCACCCGTTTGATGTAACCAAGCCGCCACTTCTCCGCATCGGCCTG 3540
pmxE_Polymyxin-A             GTGGAAGCTTACTATCACCCGTTTGATCTGACCAAGCCGCCACTTCTCCGCATCGGCCTG 3540
                             ***************   ********* * ******************************

pmxE-D-DaB3-Polymyxin-B      ATCGAAGTCGCCGCGGATCGCCACATTCTGCTGTTCGATATGCACCATATCGTCTCGGAC 3600
pmxE_1G_Polymyxin            atcgaagtcgccgaggatcgccacattctgctgttcgatatgcaccatatcgtctcggac 3600
pmxE_B-LR_Polymyxin-E        ATCGAAGTCACCGAGGATCGCCACATTCTGCTGTTCGATATGCACCATATCGTCTCGGAC 3600
pmxE_KP26_Polymyxin-E        ATCGAAGTCACCGAGGATCGCCACATTCTGCTGTTCGATATGCACCATATCGTCTCGGAC 3540
pmxE_Polymyxin-P             ATCGAAGTCGCCGAGGATCGCCACATTCTGCTGTTCGATATGCACCATATTGTCTCGGAC 3600
pmxE_Polymyxin-A             ATCGAAGTCGCCGAGGATCGCCACATTCTGCTGTTCGATATGCACCATATCGTCTCGGAT 3600
                             ********* *** ************************************ ********
```

```
pmxE-D-DaB3-Polymyxin-B      GGCATTTCGACAGCGCTGCTCTTCGACGAGTTCAGCCGCCTGTATCGGGGCGAGGAGCTG 3660
pmxE_1G_Polymyxin            ggcatttcgacagcgctgctcttcgacgagttcagccgcctgtatcggggcgaggagctg 3660
pmxE_B-LR_Polymyxin-E        GGCATATCGACAGCGCTGCTCTTCGACGAGTTCAGCCGCCTGTATCGGGGCGAGGAGCTG 3660
pmxE_KP26_Polymyxin-E        GGCATATCGACAGCGCTGCTCTTCGACGAGTTCAGCCGCCTGTATCGGGGCGAGGAGCTG 3600
pmxE_Polymyxin-P             GGCATTTCGACAGCGCTGCTCTTCGACGAGTTCAGCCGCCTGTATCGGGGCGAGGAGCTG 3660
pmxE_Polymyxin-A             GGCATTTCGACAGCGCTGCTCTTCGACGAGTTCAGCCGCCTGTATCGGGGCGAGGAGCTG 3660
                             ***** ******************************************************

pmxE-D-DaB3-Polymyxin-B      GCCCCGCTGCGTATTCAATACAAAGATTATGCCGTTTGGCAGCATTCCGAAGCTTACGGG 3720
pmxE_1G_Polymyxin            accccgctgcgcattcaatacaaagattatgccgtttggcagcattccgaagcttacggg 3720
pmxE_B-LR_Polymyxin-E        GCTCCGCTGCGCATTCAATACAAAGATTATGCCGTTTGGCAGCATTCCGAAGCTTACGCG 3720
pmxE_KP26_Polymyxin-E        GCTCCGCTGCGCATTCAATACAAAGATTATGCCGTTTGGCAGCATTCCGAAGCTTACGCG 3660
pmxE_Polymyxin-P             GTCCCGCTGCGCATTCAATACAAAGATTATGCCGTTTGGCAGCATTCCGAAGCTTACGGG 3720
pmxE_Polymyxin-A             GGCCCGCTGCGCATTCAATACAAAGATTATGCCGTTTGGCAGCATTCCGAAGCTTACGGG 3720
                                ******** ********************************************** *

pmxE-D-DaB3-Polymyxin-B      CAGCTGCTCCAGCCGCAGAAGGAGTATTGGCTGGAACAGCTGTCGGGCGAGCTGCCGGTC 3780
pmxE_1G_Polymyxin            cagatgcttcagccgcagaaggagtattggctggaacagctgtcgggcgagttgccggtc 3780
pmxE_B-LR_Polymyxin-E        CAACTGCTCCAGCCGCAGAAGGAGTACTGGCTGGAGCAGCTGTCAGGCGAGCTGCCGGTC 3780
pmxE_KP26_Polymyxin-E        CAACTGCTCCAGCCGCAGAAGGAGTACTGGCTGGAGCAGCTGTCAGGCGAGCTGCCGGTC 3720
pmxE_Polymyxin-P             CAGATGCTCCAGCCGCAGAAGGAGTACTGGCTGGAACAGCTGTCGGGCGAGCTGCCGGTC 3780
pmxE_Polymyxin-A             CAGATGCTCCAGCCGCAGAAGGAGTATTGGCTGGAACAGCTGTCGGGCGAGCTGCCGGTC 3780
                             **  **** ***************** ******** ******** ****** ********

pmxE-D-DaB3-Polymyxin-B      TTGGAGCTACCGACGGACTTCCCGCGGCCTGCGGTGCAAAGCTTTGACGGCCGAACCGTG 3840
pmxE_1G_Polymyxin            ttggagctaccgacggacttcccgcggcctgcggtgcaaagctttgacggccgaaccgtg 3840
pmxE_B-LR_Polymyxin-E        TTGGAGCTGCCGACGGATTTCCCGCGGCCAGCGGTGCAAAGCTTTGACGGCCGGACCGTG 3840
pmxE_KP26_Polymyxin-E        TTGGAGCTGCCGACGGATTTCCCGCGGCCAGCGGTGCAAAGCTTTGACGGCCGGACCGTG 3780
pmxE_Polymyxin-P             TTGGAACTGCCGACGGACTTCCCGCGGCCTGCGGTGCAAAGCTTTGACGGCCGGACCGTG 3840
pmxE_Polymyxin-A             TTGGAGCTACCGACGGACTTCCCGCGGCCTGCGGTGCAAAGCTTTGACGGTCGAACCGTG 3840
                             ***** ** ******** *********** ******************** ** ******

pmxE-D-DaB3-Polymyxin-B      AAGTTTTATATCGAGAAAGATCGGACGGAGAAGCTGAAAGAGCTGGCGGGACGGACAGGG 3900
pmxE_1G_Polymyxin            aagttttatatcgagaaagatcggacggagaagctgaaagaactggcggcacggacgggg 3900
pmxE_B-LR_Polymyxin-E        AAGTTTTATATCGGGAAAGAGCGGACGGAGAAGCTGAAAGAGCTGGCGTCACGGACGGGA 3900
pmxE_KP26_Polymyxin-E        AAGTTTTATATCGGGAAAGAGCGGACGGAGAAGCTGAAAGAGCTGGCGTCACGGACGGGA 3840
pmxE_Polymyxin-P             AAGTTTTATATCGGGAAAGAGCGGACGGAGAAGCTGAAAGAGCTGGCGGCGCGGACGGGG 3900
pmxE_Polymyxin-A             AAGTTTTATATCGAGAAAGATCGGACGGAGAAGCTGAAAGAGCTGGCGGCACGGACAGGG 3900
                             ************* ****** ******************** ******   ***** **

pmxE-D-DaB3-Polymyxin-B      ACGACCCTGTACATGGTGCTGCTGTCGGCTTACACCATCCTTATGCATAAATATTCGGGT 3960
pmxE_1G_Polymyxin            acgaccctgtacatggtgctgctgtcggcttacaccatccttatgcataaatattcgggt 3960
pmxE_B-LR_Polymyxin-E        ACGACCCTGTACATGGTGCTGCTGTCGGCTTATACCATCCTTATGCATAAATATTCGGGT 3960
```

```
pmxE_KP26_Polymyxin-E        ACGACCCTGTACATGGTGCTGCTGTCGGCTTATACCATCCTTATGCATAAATATTCGGGT 3900
pmxE_Polymyxin-P             ACGACCCTGTACATGGTGCTGCTGTCGGCTTACTCCATCCTTATGCATAAATATTCGGGT 3960
pmxE_Polymyxin-A             ACGACCCTGTACATGGTGCTGCTGTCGGCTTACACCATCCTTATGCATAAATATTCGGGT 3960
                             ********************************  **************************

pmxE-D-DaB3-Polymyxin-B      CAGGAAGATCTGATCGTAGGCACGCCGATTGCCGGAAGAACGCAGGAAGAAGTGCAGCCG 4020
pmxE_1G_Polymyxin            caggaagatctgatcgtaggaacgccgattgccggaagaacgcaggaagaagtgcagccg 4020
pmxE_B-LR_Polymyxin-E        CAGGAAGATCTGATCGTCGGAACGCCAATTGCCGGAAGAACGCAGGATGAAGTGCAGCCG 4020
pmxE_KP26_Polymyxin-E        CAGGAAGATCTGATCGTCGGAACGCCAATTGCCGGAAGAACGCAGGATGAAGTGCAGCCG 3960
pmxE_Polymyxin-P             CAGGAAGATCTGATTGTCGGAACGCCGATTGCCGGAAGAACGCAGGAAGAAGTGCAGCCG 4020
pmxE_Polymyxin-A             CAGGAAGATCTGATCGTAGGAACGCCGATTGCCGGAAGAACGCAGGAAGAAGTGCAGCCG 4020
                             ************** ** ** ***** ******************** ************

pmxE-D-DaB3-Polymyxin-B      ATCGTAGGGATGTTTATCAACACGCTGGCCATTCGCAGCCGTCCGGAGCGTTCCAAGCCG 4080
pmxE_1G_Polymyxin            atcgtagggatgtttatcaacacgctggccattcgcagccgtccggagcgttccaagccg 4080
pmxE_B-LR_Polymyxin-E        ATCGTAGGGATGTTTATCAACACGCTGACCATTCGCAGCCGTCCGGAGCGTTCCAAGCCA 4080
pmxE_KP26_Polymyxin-E        ATCGTAGGGATGTTTATCAACACGCTGACCATTCGCAGCCGTCCGGAGCGTTCCAAGCCA 4020
pmxE_Polymyxin-P             ATCGTGGGGATGTTTGTCAACACGCTGGCCATTCGCAGCCGTCCGGAGCGTTCCAAGCCG 4080
pmxE_Polymyxin-A             ATCGTAGGGATGTTTATCAACACGCTGGCCATTCGCAGCCGTCCGGAGCGTTCCAAGCCG 4080
                             ***** ********* *********** ******************************* 

pmxE-D-DaB3-Polymyxin-B      TACCTTTCGTACCTGGAAGAAATCAAGGACATCACACTCGGGGCTTTCGAACACCAAAAT 4140
pmxE_1G_Polymyxin            tacctttcgtacctggaagaaatcaaggacatcacgctcggggctttcgaacaccaaaat 4140
pmxE_B-LR_Polymyxin-E        TACCTTTCGTACCTGGAAGAAATCAAGGACATCACGCTCGGGGCTTTCGAACACCAAAAT 4140
pmxE_KP26_Polymyxin-E        TACCTTTCGTACCTGGAAGAAATCAAGGACATCACGCTCGGGGCTTTCGAACACCAAAAT 4080
pmxE_Polymyxin-P             TACCTTTCGTACCTGGAAGAAATCAAGGACATCACGCTCGGGGCTTTCGAACACCCAAAT 4140
pmxE_Polymyxin-A             TACCTTTCGTACCTGGAAGAAATCAAGGACATCACGCTCGGGGCTTTCGAACACCAAAAT 4140
                             *********************************** ******************* ****

pmxE-D-DaB3-Polymyxin-B      TATTTGTTCGAAGACTTGGTGGAAAGTCTTCACATTCCGCGCGCGACCGGCCGGAATCCG 4200
pmxE_1G_Polymyxin            tatttgttcgaagacttggtagtaagtcttcacattccgcgcgcgaccggccggaatccg 4200
pmxE_B-LR_Polymyxin-E        TATTTGTTCGAAGACTTGGTGGAAAGTCTTCACATTCCGCGCGCGACCGGCCGGAATCCG 4200
pmxE_KP26_Polymyxin-E        TATTTGTTCGAAGACTTGGTGGAAAGTCTTCACATTCCGCGCGCGACCGGCCGGAATCCG 4140
pmxE_Polymyxin-P             TATTTGTTCGAAGACTTGGTGGAAAGTCTTCACATTCCACGCGCGACCGGCCGGAATCCG 4200
pmxE_Polymyxin-A             TATTTGTTCGAAGACTTGGTGGAAAGTCTTCATATTCCACGCGCGACCGGCCGGAATCCG 4200
                             ******************** * ********* ***** *********************

pmxE-D-DaB3-Polymyxin-B      CTCTTTGACACGTTCTTCTCCCTACAAAATACGGAGAACGAGCAAATTGTCATCGAGGGG 4260
pmxE_1G_Polymyxin            ctctttgacacgttcttctccctacaaaatacggagaacgagcaaattgtcatcgagggg 4260
pmxE_B-LR_Polymyxin-E        CTCTTTGATACGTTCTTCTCCCTGCAAAATACGGAGAACGAGCAAATTGTCATTGAGGGG 4260
pmxE_KP26_Polymyxin-E        CTCTTTGATACGTTCTTCTCCCTGCAAAATACGGAGAACGAGCAAATTGTCATTGAGGGG 4200
pmxE_Polymyxin-P             CTCTTTGATACGTTCTTCGCCCTGCAAAATACGGAAAACGAGCAAATTGTCATCGAGGGG 4260
pmxE_Polymyxin-A             CTCTTTGACACGTTCTTCTCCCTACAAAATACGGAGAACGAGCAAATTGTCATCGAGGGG 4260
                             ******** ********* **** *********** ***************** ******
```

```
pmxE-D-DaB3-Polymyxin-B      CTGGAGCAATCGTTTTATCCACTGGAAAACCAAACATCCAAGTTCGAGCTGCTCCTGGAT 4320
pmxE_1G_Polymyxin            ctggagcaatcgttttatccactggaaaaccaaacatccaagttcgagctgctcctggat 4320
pmxE_B-LR_Polymyxin-E        CTGGAGCAATCGTTTTATCCGCTGGAAAACCGAACATCCAAGTTCGAGCTGCTCCTGGAC 4320
pmxE_KP26_Polymyxin-E        CTGGAGCAATCGTTTTATCCGCTGGAAAACCGAACATCCAAGTTCGAGCTGCTCCTGGAC 4260
pmxE_Polymyxin-P             CTGGAGCAATCGTTTTATCCGCTGGAAAACCGAACATCCAAGTTCGAGCTGCTCCTGGAC 4320
pmxE_Polymyxin-A             CTGGAGCAATCGTTTTATCCGCTGGAAAACCAAACATCCAAGTTCGAGCTGCTCCTGGAT 4320
                             ******************** ********** *************************** 

pmxE-D-DaB3-Polymyxin-B      ATTTCGGAGCTGGACGGTCAGCTCGAATGCCGGTTGGAGTACGCTACGGCTTTGTATAAA 4380
pmxE_1G_Polymyxin            atttcggagctggacggtcagctcgaatgccggttggagtacgctacggctttgtataaa 4380
pmxE_B-LR_Polymyxin-E        ATTTCTGAGCAGGACGGTCAGCTCGAATGCCGGTTGGAGTACGCAACGGCTTTGTATAAA 4380
pmxE_KP26_Polymyxin-E        ATTTCTGAGCAGGACGGTCAGCTCGAATGCCGGTTGGAGTACGCAACGGCTTTGTATAAA 4320
pmxE_Polymyxin-P             ATTTCGGAGCTGGACGGTCAGCTCGAATGCCGGTTGGATTACGCTACGGCTTTGTATAAA 4380
pmxE_Polymyxin-A             ATTTCGGAGCTGGACGGTCAGCTCGAATGCCGGTTAGAGTACGCTACGGCTTTGTATAAA 4380
                             ***** **** ************************ ** ***** ***************

pmxE-D-DaB3-Polymyxin-B      CAGGAGACCGCGGAGCGGTTCGCCAGACATTATGACAAGCTGCTCGAAACCATCGCAGCA 4440
pmxE_1G_Polymyxin            caggagaccgcggagcggttcgccagacattatgacaagctgctcgaaaccatcgcagca 4440
pmxE_B-LR_Polymyxin-E        CAGGAGACCGCGGAACGGTTCGCCAAACATTATGACAAGCTGTTAGAAACCATCGCAGCA 4440
pmxE_KP26_Polymyxin-E        CAGGAGACCGCGGAACGGTTCGCCAAACATTATGACAAGCTGTTAGAAACCATCGCAGCA 4380
pmxE_Polymyxin-P             CAGGAGACCGCGGAGCGGTTCGCCAGACATTATGACAAGCTGCTGGAAACCATCGCAGCA 4440
pmxE_Polymyxin-A             CAGGAGACCGCGGAGCGGTTCGCCAGACATTATGACAAGCTGCTTGAAACCATCGCAGCA 4440
                             ************** ********** **************** * ***************

pmxE-D-DaB3-Polymyxin-B      GGGCCGGACGGGGATATTGCCTCGCTGGAAATGCTCACGGAGGAGGAAATCCGCGAGCTG 4500
pmxE_1G_Polymyxin            gcgccggacggggatattgcctcgttggaaatgctcacggaggaggaaatccgcgagctg 4500
pmxE_B-LR_Polymyxin-E        GCGCCGGACGGGGATATTGCCTCGCTGGAAATGCTCACGGAGGAGGAAATCCGCGAACTA 4500
pmxE_KP26_Polymyxin-E        GCGCCGGACGGGGATATTGCCTCGCTGGAAATGCTCACGGAGGAGGAAATCCGCGAACTA 4440
pmxE_Polymyxin-P             GCGCCGGACGGGGATATTGCCTCGCTGGAAATGCTCACAGAGGAGGAAATCCGCGAGTTG 4500
pmxE_Polymyxin-A             GCGCCGGACGGGGATATTGCCTCGCTGGAAATGCTCAAGGAGGAGGAAATCCGCGAGCTG 4500
                             * ********************** ************  *****************  * 

pmxE-D-DaB3-Polymyxin-B      GTGCGCGGTTTCAACGATTCGGAGGCGGACTACCCGCGGCAGCAGACGATTCACGGCTTG 4560
pmxE_1G_Polymyxin            gtgcgcggtttcaacgattcggaggcggactacccgcggcagcagacgattcacggcttg 4560
pmxE_B-LR_Polymyxin-E        GTACGTGGTTTCAACGATTCGGAGGCGGACTACCCGCGGCAGCAGACGATTCACGGCTTG 4560
pmxE_KP26_Polymyxin-E        GTACGTGGTTTCAACGATTCGGAGGCGGACTACCCGCGGCAGCAGACGATTCACGGCTTG 4500
pmxE_Polymyxin-P             GTGGGCGGTTTCAACGATTCGGAGGCGGACTACCCGCGGCAGCAGACGATTCACGGCTTG 4560
pmxE_Polymyxin-A             GTGCGCGGTTTCAACGATTCGGAGGCGGACTACCCGCGGCAGCAGACGATTCACGGCTTG 4560
                             **  * ******************************************************

pmxE-D-DaB3-Polymyxin-B      TTCGAAGAGCAGGCGGAGCTTTACCCGGACAACGTGGCCGCCGTCATGAACGAGCGGCAG 4620
pmxE_1G_Polymyxin            ttcgaagagcaggcggagctttacccggacaacgtggccgccgtcatgaacgagcggcag 4620
pmxE_B-LR_Polymyxin-E        TTCGAAGAGCAGGCAGAGCTTTACCCGGACAACGTGGCCGCCGTCATGAACGAGCGGCAG 4620
```

```
pmxE_KP26_Polymyxin-E        TTCGAAGAGCAGGCAGAGCTTTACCCGGACAACGTGGCCGCCGTCATGAACGAGCGGCAG 4560
pmxE_Polymyxin-P             TTCGAAGAGCAGGCGGAGCTTTACCCGGACAACGTAGCCGCCGTCATGAACGAGCGGCAG 4620
pmxE_Polymyxin-A             TTCGAAGAACAGGCGGAGCTTTACCCGGACAACGTGGCCGCCGTCATGAACGAGCGGCAA 4620
                             ******** ***** ******************** *********************** 

pmxE-D-DaB3-Polymyxin-B      CTGACCTACCGCGAGCTGAACGAGCGATCCAACCGCCTTGCGCGGAAGCTGCGGAAGACG 4680
pmxE_1G_Polymyxin            ctgacctaccgcgagctgaacgagcgatccaaccgccttgcgcggaagctgcgggagacg 4680
pmxE_B-LR_Polymyxin-E        CTGACCTACCGCGAGCTGAACGAGCGATCCAACCGCCTTGCGCGGAAGCTGCGGGAGACG 4680
pmxE_KP26_Polymyxin-E        CTGACCTACCGCGAGCTGAACGAGCGATCCAACCGCCTTGCGCGGAAGCTGCGGGAGACG 4620
pmxE_Polymyxin-P             CTGACATACCGCGAGCTGAACGAGCGATCCAACCGCCTTGCGCGGAAGCTGCGGGAGACG 4680
pmxE_Polymyxin-A             CTGACCTACCGCGAGCTGAACGAGCGATCCAACCGCCTTGCGCGGAAGCTGCGAGAGACG 4680
                             ***** ***********************************************  *****

pmxE-D-DaB3-Polymyxin-B      GGAGTAGAAGCGGACCAGCTGGTAGCGATTCTGGCCGAACGCTCGCTCGATATGGTTGTC 4740
pmxE_1G_Polymyxin            ggagtagaagcggaccagctggtagcgattctggccgaacgctcgctcgatatggtcgtc 4740
pmxE_B-LR_Polymyxin-E        GGAGTAGAAGCGGATCAACTGGTAGCGATTCTGGCCGAACGCTCGCTCGATATGGTTGTC 4740
pmxE_KP26_Polymyxin-E        GGAGTAGAAGCGGATCAACTGGTAGCGATTCTGGCCGAACGCTCGCTCGATATGGTTGTC 4680
pmxE_Polymyxin-P             GGAGTAGAAGCGGACCAACTGGTAGCGATTCTGGCCGAACGCTCGCTCGATATGGTCGTC 4740
pmxE_Polymyxin-A             GGAGTAGAAGCGGACCAGCTGGTAGCGATTCTGGCCGAACGCTCGCTCGATATGGTCGTC 4740
                             ************** ** ************************************** ***

pmxE-D-DaB3-Polymyxin-B      GGCATTCTGGCGATTCTCAAAGCGGGCGGAGCATACGTGCCTGTCGATCCCGACTACCCG 4800
pmxE_1G_Polymyxin            ggcattctggcgattctcaaagcgggcggagcctacgtgcctgtcgatcccgactacccg 4800
pmxE_B-LR_Polymyxin-E        GGCATTCTGGCGATTCTTAAAGCGGGCGGAGCCTACGTGCCTGTCGATCCCGACTACCCG 4800
pmxE_KP26_Polymyxin-E        GGCATTCTGGCGATTCTTAAAGCGGGCGGAGCCTACGTGCCTGTCGATCCCGACTACCCG 4740
pmxE_Polymyxin-P             GGCATTCTGGCGATTCTCAAAGCGGGCGGAGCATACGTGCCTGTCGATCCCGACTACCCG 4800
pmxE_Polymyxin-A             GGCATTCTGGCGATTCTCAAAGCGGGCGGAGCCTACGTGCCTGTCGATCCCGACTACCCG 4800
                             ***************** ************** ***************************

pmxE-D-DaB3-Polymyxin-B      GAGGAGCGCATCCGCTTCATGATCGAGGATTCGGGCGCGCCGTTATTGCTGATTCAAAAG 4860
pmxE_1G_Polymyxin            gaggagcgcatccgcttcatgatcgaggattcgggcgcgccgttattgctgattcaaaag 4860
pmxE_B-LR_Polymyxin-E        GAGGAGCGTATCCGCTTCATGATCGAGGATTCGGGCGCGCCGTTATTGCTGATTCAAAAG 4860
pmxE_KP26_Polymyxin-E        GAGGAGCGTATCCGCTTCATGATCGAGGATTCGGGCGCGCCGTTATTGCTGATTCAAAAG 4800
pmxE_Polymyxin-P             GAGGAGCGTATCCGCTTCATGATCGAGGATTCGGGCGCGCCGTTATTGCTGATTCAAAAG 4860
pmxE_Polymyxin-A             GAGGAGCGCATCCGCTTCATGATCGAGGATTCGGGCGCGCCGTTATTGCTGATTCAAAAG 4860
                             ******** ***************************************************

pmxE-D-DaB3-Polymyxin-B      CATCTGCACGAGAAGACCGACTTCGCAGGAACGCGCCTCGAATTGGACGATTTCGTTTGG 4920
pmxE_1G_Polymyxin            catctgcacgagaagaccgacttcgcaggaacgcgcctcgaattggacgatttcgtttgg 4920
pmxE_B-LR_Polymyxin-E        CATTTGCACGAGAAGACCGACTTCGCAGGAACACGCCTCGAATTGGATGATTTCGTTTGG 4920
pmxE_KP26_Polymyxin-E        CATTTGCACGAGAAGACCGACTTCGCAGGAACACGCCTCGAATTGGATGATTTCGTTTGG 4860
pmxE_Polymyxin-P             CATCTGCACGAGAAGACCGACTTCGCAGGAACGCGCCTCGAATTGGACGATTACGTTTGG 4920
pmxE_Polymyxin-A             CATCTGCACGAGAAGACCGACTTCGCAGGAACGCGCCTCGAATTGGACGATTTCGTTTGG 4920
                             *** *************************** *************** **** *******
```

```
pmxE-D-DaB3-Polymyxin-B      GGCGACAGAGGGGCGGACTCCGCCGATGCGCTGGACGCTTCGAACTTGGAGCCGATTTCC 4980
pmxE_1G_Polymyxin            ggcgacagaggggcggactccgaaggtgcgctggacgcttcgaacctggagccgatttcc 4980
pmxE_B-LR_Polymyxin-E        GGCGACAGAGGTGCGGACTCCGAAGGTGCGCTGGATGCTTCGAATCTGGATCCGATTTCC 4980
pmxE_KP26_Polymyxin-E        GGCGACAGAGGTGCGGACTCCGAAGGTGCGCTGGATGCTTCGAATCTGGATCCGATTTCC 4920
pmxE_Polymyxin-P             GGTGACAGAGGGGCGGACTCCGCCGATGCACTGGACGCTTCGAACCTGGAGCCGATTTCC 4980
pmxE_Polymyxin-A             GGCGACAGAGGGGCGAACTCCGCCGAAGCGCTGGACGCTTCGAACCTGGAGCCAATTTCC 4980
                             ** ******** *** ******  *  ** ***** ********  **** ** ******

pmxE-D-DaB3-Polymyxin-B      GGGCCGGGCAACCTGGCTTATGTCATCTACACGTCGGGAACGACCGGCAGACCGAAAGGA 5040
pmxE_1G_Polymyxin            gggccgggcaacctggcatatgtcatctacacgtcgggaacgaccggcagaccgaaagga 5040
pmxE_B-LR_Polymyxin-E        GGGCCGGGCAACCTAGCCTATGTCATCTACACATCGGGAACGACCGGCAGACCGAAAGGA 5040
pmxE_KP26_Polymyxin-E        GGGCCGGGCAACCTAGCCTATGTCATCTACACATCGGGAACGACCGGCAGACCGAAAGGA 4980
pmxE_Polymyxin-P             GGACCGGGCAACCTGGCCTATGTCATCTACACGTCGGGAACGACCGGCAGACCGAAAGGA 5040
pmxE_Polymyxin-A             GGGCCGGGCAACCTGGCATATGTCATCTACACGTCGGGAACGACCGGCAGACCGAAAGGA 5040
                             ** *********** ** ************** ***************************

pmxE-D-DaB3-Polymyxin-B      ACGCTGATCGAGCATAAGAACGTCGTGCGCCTCCTGTTCAACGACAAGAACCTGTTCGAC 5100
pmxE_1G_Polymyxin            acgctgatcgagcataagaacgtcgtgcggctcctgttcaacgacaagaacctgttcgac 5100
pmxE_B-LR_Polymyxin-E        ACTTTGATCGAGCATAAGAACGTCGTGCGCCTCCTGTTCAACGACAAGAATCTGTTCGAC 5100
pmxE_KP26_Polymyxin-E        ACTTTGATCGAGCATAAGAACGTCGTGCGCCTCCTGTTCAACGACAAGAATCTGTTCGAC 5040
pmxE_Polymyxin-P             ACGCTGATCGAGCATAAGAACGTCGTGCGGCTCCTGTTCAACGACAAGAACCTGTTCGAC 5100
pmxE_Polymyxin-A             ACGCTGATCGAGCATAAGAACGTCGTGCGGCTCCTGTTCAACGACAAGAACCTGTTCGAC 5100
                             **  ************************* ******************** *********

pmxE-D-DaB3-Polymyxin-B      TTCGGGCCGTCCGACACGTGGACGCTGTTCCACTCGTTCTGCTTCGATTTCTCCGTCTGG 5160
pmxE_1G_Polymyxin            ttcgggccgtccgacacgtggacgctgttccactcgttctgcttcgatttctccgtctgg 5160
pmxE_B-LR_Polymyxin-E        TTCGGGCCGTCCGACACGTGGACGCTGTTCCACTCGTTCTGCTTCGATTTCTCCGTCTGG 5160
pmxE_KP26_Polymyxin-E        TTCGGGCCGTCCGACACGTGGACGCTGTTCCACTCGTTCTGCTTCGATTTCTCCGTCTGG 5100
pmxE_Polymyxin-P             TTCGGGCCGTCCGACACGTGGACGCTGTTCCACTCGTTCTGCTTCGATTTCTCCGTCTGG 5160
pmxE_Polymyxin-A             TTCGGGCCGTCCGACACGTGGACGCTGTTCCACTCGTTCTGCTTCGATTTCTCCGTCTGG 5160
                             ************************************************************

pmxE-D-DaB3-Polymyxin-B      GAAATGTACGGAGCGCTGCTGTATGGAGGCAAGCTGGTCATCGTACCGCCACTCACGGCG 5220
pmxE_1G_Polymyxin            gaaatgtacggagcgctgctgaacggaggcaagctggtcatcgtaccgccgctcacggcg 5220
pmxE_B-LR_Polymyxin-E        GAAATGTACGGAGCACTGCTGTACGGAGGCAAGCTGGTCATCGTACCGCCGCTCATGGCG 5220
pmxE_KP26_Polymyxin-E        GAAATGTACGGAGCACTGCTGTACGGAGGCAAGCTGGTCATCGTACCGCCGCTCATGGCG 5160
pmxE_Polymyxin-P             GAAATGTACGGAGCGCTGCTGTACGGAGGCAAGCTGGTCATCGTACCGCCGCTCACGGCG 5220
pmxE_Polymyxin-A             GAAATGTACGGAGCGCTGCTGTACGGAGGCAAGCTGGTCATCGTACCGCCGCTCACGGCG 5220
                             ************** ****** * ************************** **** ****

pmxE-D-DaB3-Polymyxin-B      AAAAATCCGGCCGATTTCCTGGCGCTGCTGGGCCGCGAACAGGTCACGATTTTGAACCAG 5280
pmxE_1G_Polymyxin            aaaaatccggtcgatttcctggcgctgctgggccgcgaacaggtcacgattttgaaccag 5280
pmxE_B-LR_Polymyxin-E        AAAAATCCGGCCGATTTCCTAGCGCTGCTGGGCCGCGAACAGATCACGATTTTGAACCAG 5280
```

```
pmxE_KP26_Polymyxin-E        AAAAATCCGGCCGATTTCCTAGCGCTGCTGGGCCGCGAACAGATCACGATTTTGAACCAG 5220

pmxE_Polymyxin-P             AAAAATCCGGCCGATTTCCTGGCGCTACTGGGCCGCGAACAGGTCACGATTTTGAACCAG 5280

pmxE_Polymyxin-A             AAAAATCCGGCCGATTTCCTGGCGCTGCTGGGCCGCGAACAGGTTACCATTTTGAACCAG 5280

                             ********** ********* ***** *************** * ** ************


pmxE-D-DaB3-Polymyxin-B      ACGCCAACGTACTTCTACCAGCTGCTACGTGAGGTCTTGGCGGACCATCCGTACGATCTG 5340

pmxE_1G_Polymyxin            acgccaacgtacttctaccagctgctgcgtaaggtcttggcggaccatccgtacgatctg 5340

pmxE_B-LR_Polymyxin-E        ACGCCAACGTACTTCTACCAGCTGCTGCGTGAGGTCTTGGCGGACCATCCGTACGATCTG 5340

pmxE_KP26_Polymyxin-E        ACGCCAACGTACTTCTACCAGCTGCTGCGTGAGGTCTTGGCGGACCATCCGTACGATCTG 5280

pmxE_Polymyxin-P             ACACCAACGTACTTCTACCAGCTGCTGCGTGAGGTCTTGGCGGACCATCCGTACGATCTG 5340

pmxE_Polymyxin-A             ACGCCAACATACTTCTACCAGCTGCTGCGTGAGGTCTTGGCGGACCATCCGTACGATCTG 5340

                             ** ***** ***************** *** *****************************


pmxE-D-DaB3-Polymyxin-B      CGGATTCGCAACGTCATCTTCGGGGGCGAAGCGCTCAGTCCGCTGCTGCTCAAGGGCTTC 5400

pmxE_1G_Polymyxin            cggattcgcaacgtcatcttcgggggcgaagcgctgagtccgctgctgctcaagggcttc 5400

pmxE_B-LR_Polymyxin-E        CGGATTCGCAACGTCATCTTCGGGGGCGAAGCACTGAGTCCGCTGCTGCTCAAGGGCTTC 5400

pmxE_KP26_Polymyxin-E        CGGATTCGCAACGTCATCTTCGGGGGCGAAGCACTGAGTCCGCTGCTGCTCAAGGGCTTC 5340

pmxE_Polymyxin-P             CGGATTCGCAACGTCATCTTCGGGGGCGAAGCGCTGAGTCCGCTGCTGCTCAAGGGCTTC 5400

pmxE_Polymyxin-A             CGGATTCGCAACGTCATCTTCGGGGGCGAAGCGCTGAGTCCGCTGCTGCTCAAGGGCTTC 5400

                             ******************************** ** ************************


pmxE-D-DaB3-Polymyxin-B      AAGACGAAGTACCCGGAGACGAAGCTGATTAATATGTACGGCATTACCGAGACGACGGTT 5460

pmxE_1G_Polymyxin            aagacgaagtacccggagacgaagctaatcaatatgtacggcattaccgagacgacggtt 5460

pmxE_B-LR_Polymyxin-E        AAGACGAAGTACCCGGAGACGAAGCTGATCAATATGTACGGCATTACCGAGACGACGGTT 5460

pmxE_KP26_Polymyxin-E        AAGACGAAGTACCCGGAGACGAAGCTGATCAATATGTACGGCATTACCGAGACGACGGTT 5400

pmxE_Polymyxin-P             AAGACGAAGTACCCGGAGACAAAGCTGATCAATATGTACGGCATTACCGAGACGACGGTT 5460

pmxE_Polymyxin-A             AAGACGAAGTACCCGGAGACGAAGCTGATCAATATGTACGGCATTACCGAGACGACGGTT 5460

                             ******************** ***** ** ******************************


pmxE-D-DaB3-Polymyxin-B      CACGTTACGTATAAGGAAATTACGTGGGTCGAGATTGAGGCGGCGAAGAGCAATATCGGC 5520

pmxE_1G_Polymyxin            cacgtgacatataaggaaatcacatgggtcgaaatagaggcggcgaagagcaatatcggc 5520

pmxE_B-LR_Polymyxin-E        CACGTTACGTATAAGGAAATCACGTGGGTCGAAATGGAGGCGGCGAAGAGCAATATCGGC 5520

pmxE_KP26_Polymyxin-E        CACGTTACGTATAAGGAAATCACGTGGGTCGAAATGGAGGCGGCGAAGAGCAATATCGGC 5460

pmxE_Polymyxin-P             CACGTGACGTATAAGGAAATCACGTGGGTCGAAATGGAGGCGGCGAAGAGCAATATCGGC 5520

pmxE_Polymyxin-A             CACGTGACGTATAAGGAAATCACGTGGGTCGAAATGGAGGCGGCGAAGAGCAATATCGGC 5520

                             ***** ** *********** ** ******** ** ************************


pmxE-D-DaB3-Polymyxin-B      AAGCCGATCCCGACGCTGAGCGTATACGTCCTTGATGAAAACCGCCGCCCTGTGCCGATC 5580

pmxE_1G_Polymyxin            aagccgatcccgacgctgagggtgtacgtcctggatgaaagctgccgccctgtgccgatc 5580

pmxE_B-LR_Polymyxin-E        AAGCCGATCCCGACGCTGAGGGTGTACGTCCTTGATGAAAACCGCCGCCCTGTGCCGATC 5580

pmxE_KP26_Polymyxin-E        AAGCCGATCCCGACGCTGAGGGTGTACGTCCTTGATGAAAACCGCCGCCCTGTGCCGATC 5520

pmxE_Polymyxin-P             AAGCCGATCCCGACGCTGAGGGTGTACGTCCTGGATGAAAACCGCCGCCCTGTGCCGATC 5580

pmxE_Polymyxin-A             AAGCCGATCCCGACGTTGAGGGTGTACGTCCTAGATGAAAACCGCCGCCCTGTGCCGATC 5580

                             *************** **** ** ******** ******* * *****************
```

```
pmxE-D-DaB3-Polymyxin-B      GGCGTAGCGGGCGAAATGTACGTGGCCGGGGAAGGCCTTGCGAGAGGATACCTGAACCGC 5640
pmxE_1G_Polymyxin            ggcgtagcgggcgaaatgtacgtggccggggaaggccttgcaagaggatatctgaaccgt 5640
pmxE_B-LR_Polymyxin-E        GGCGTAGCGGGCGAAATGTATGTGGCCGGGGAAGGCCTTGCGAGAGGATACCTGAACCGT 5640
pmxE_KP26_Polymyxin-E        GGCGTAGCGGGCGAAATGTATGTGGCCGGGGAAGGCCTTGCGAGAGGATACCTGAACCGT 5580
pmxE_Polymyxin-P             GGTGTAGCGGGCGAAATGTACGTGGCCGGGGAAGGCCTTGCGAGAGGATACCTGAACCGT 5640
pmxE_Polymyxin-A             GGCGTAGCGGGCGAAATGTACGTGGCCGGGGAAGGCCTTGCGAGAGGATACCTGAACCGT 5640
                             ** ***************** ******************** ******** ********

pmxE-D-DaB3-Polymyxin-B      CCGGATCTGACGGCGGAGAAGTTCGTCGATTCCCCGTTTGCGGAGGGGGAGAAACTGTAC 5700
pmxE_1G_Polymyxin            ccggatctgacggcggagaagttcgtcgattccccgtttgcggagggggagaaactgtac 5700
pmxE_B-LR_Polymyxin-E        CCGGATCTGACGGCGGAGAAGTTTGTCGATTCCCCGTTTGCGGAGGGGGAGAAACTGTAC 5700
pmxE_KP26_Polymyxin-E        CCGGATCTGACGGCGGAGAAGTTTGTCGATTCCCCGTTTGCGGAGGGGGAGAAACTGTAC 5640
pmxE_Polymyxin-P             CCGGATCTGACGGCGGAGAAGTTCGTCGATTCCCCGTTTGCGGAGGGGGAGAAGCTGTAC 5700
pmxE_Polymyxin-A             CCGGATCTGACGGCGGAGAAGTTCGTTGATTCCCCGTTTGCGGAGGGGGAGAAACTGTAC 5700
                             *********************** ** ************************** ******

pmxE-D-DaB3-Polymyxin-B      CGCTCGGGCGACTTGGCGGCTTGGCTGCCGGACGGCAACATCGAATACCTGGGCCGGATC 5760
pmxE_1G_Polymyxin            cgctcgggcgacttggcggcttggctgccggacggcaatatcgaatacctgggccgaatc 5760
pmxE_B-LR_Polymyxin-E        CGCTCGGGCGACTTGGCGGCTTGGCAGCCGGACGGCAACATCGAATACCTGGGCCGGATC 5760
pmxE_KP26_Polymyxin-E        CGCTCGGGCGACTTGGCGGCTTGGCAGCCGGACGGCAACATCGAATACCTGGGCCGGATC 5700
pmxE_Polymyxin-P             CGCTCGGGCGACTTGGCAGCTTGGCTGCCGGACGGCAACATCGAATACCTAGGCCGGATC 5760
pmxE_Polymyxin-A             CGTTCGGGCGACTTGGCGGCTTGGCTGCCGGACGGCAACATCGAATACCTGGGCCGGATC 5760
                             ** ************** ******* ************ *********** ***** ***

pmxE-D-DaB3-Polymyxin-B      GATCATCAGGTGAAAATCCGCGGCTACCGGATCGAGCTGGACGAAATCGAGACGCAGCTG 5820
pmxE_1G_Polymyxin            gatcaccaggtcaaaatccgcggttaccggatcgaactggacgaaatcgagacgcagctg 5820
pmxE_B-LR_Polymyxin-E        GACCACCAGGTAAAAATCCGCGGGTACCGGATCGAGCTGGATGAAATCGAGACGCAGCTT 5820
pmxE_KP26_Polymyxin-E        GACCACCAGGTAAAAATCCGCGGGTACCGGATCGAGCTGGATGAAATCGAGACGCAGCTT 5760
pmxE_Polymyxin-P             GATCATCAGGTGAAAATCCGCGGGTACCGGATCGAGTTGGACGAAATCGAGACGCAGCTT 5820
pmxE_Polymyxin-A             GACCACCAGGTCAAAATCCGTGGGTACCGGATCGAGCTGGACGAAATCGAGACGCAGCTT 5820
                             ** ** ***** ******** ** ***********  **** *****************

pmxE-D-DaB3-Polymyxin-B      CTGAACGTCGAGGGCGTGGAAGAAGCGGTGGTGCTCGCCCGTCAGGACGGCGGGGGCGAG 5880
pmxE_1G_Polymyxin            ctgaacgtcgagggcgtggaagaagcggtggtgcttgcccgtcaggacggcgggggcgag 5880
pmxE_B-LR_Polymyxin-E        CTGAACGTTGAGGGCGTGGAAGAAGCGGTGGTGCTTGCTCGTCAGGACGGTGGGGGCGAG 5880
pmxE_KP26_Polymyxin-E        CTGAACGTTGAGGGCGTGGAAGAAGCGGTGGTGCTTGCTCGTCAGGACGGTGGGGGCGAG 5820
pmxE_Polymyxin-P             CTGAACGTCGAGGGCGTGGAAGAAGCGGTGGTGCTCGCCCGTCAGGACGGCGGGGGAGAG 5880
pmxE_Polymyxin-A             CTGAACGTCCGGGGTGTGGAAGAAGCGGTGGTGCTCGCCCGTCAGGACGGCGGGGGCGAG 5880
                             ********   *** ******************** ** *********** ***** ***

pmxE-D-DaB3-Polymyxin-B      AAGGCGCTTGTCGCCTACTTTGTGGCGGACCGGACGCTGACGGCCAGCGAAATGAGAACC 5940
pmxE_1G_Polymyxin            aaggcacttgtcgcctactttgtggcggaccggacactgacggtcagcgaaatgagaacc 5940
pmxE_B-LR_Polymyxin-E        AAGGCGCTTGTCGCCTACTTTGTGGCGAACCGGACACTGACGGTCAGTGAAATGAGAACC 5940
```

```
pmxE_KP26_Polymyxin-E        AAGGCGCTTGTCGCCTACTTTGTGGCGAACCGGACACTGACGGTCAGTGAAATGAGAACC 5880
pmxE_Polymyxin-P             AAGGCGCTTGTCGCCTACTTTGTGGCGGACCGGACGCTGACGGTCAGTGAAATGAGAACC 5940
pmxE_Polymyxin-A             AAGGCGCTTGTCGCCTACTTTGTGGCGGACCGGACGCTGACGGTCAGCGAAATGAGAACC 5940
                             ***** ********************* ******* ******* *** ************

pmxE-D-DaB3-Polymyxin-B      TCGCTGGCCCAGGAAATGCCGGGGTACATGATCCCGTCGTACTTCGTGCAGCTGGAGCGC 6000
pmxE_1G_Polymyxin            tcactggcccagggaatgccggggtacatgatcccgtcgtacttcgtgcagctggagcgc 6000
pmxE_B-LR_Polymyxin-E        TCACTGGCCAAGGAAATGCCGGGGTACATGATCCCGTCGTACTTCGTGCAGCTGGAGCGT 6000
pmxE_KP26_Polymyxin-E        TCACTGGCCAAGGAAATGCCGGGGTACATGATCCCGTCGTACTTCGTGCAGCTGGAGCGT 5940
pmxE_Polymyxin-P             TCGCTGGCCCAGGGAATGCCGGGGTACATGATCCCTTCGTACTTCGTGCAGTTGGAGCGC 6000
pmxE_Polymyxin-A             TCGCTGGCCCAGGGAATGCCGGGGTACATGATCCCGTCGTACTTCGTGCAGTTGGAGCGC 6000
                             ** ****** *** ********************* *************** *******

pmxE-D-DaB3-Polymyxin-B      ATGCCGCTGACGTCCAACGGAAAAGTGGACCGCAAAGCGCTGCCGGAGCCGCAAGGCGGC 6060
pmxE_1G_Polymyxin            atgccgctgacggccaacggcaaagtagaccgcaaagcgctgccggagccccaaggcagc 6060
pmxE_B-LR_Polymyxin-E        ATGCCGCTGACGTCCAACGGCAAAGTGGATCGCAAAGCCCTGCCGGAGCCGCAAGGCGGC 6060
pmxE_KP26_Polymyxin-E        ATGCCGCTGACGTCCAACGGCAAAGTGGATCGCAAAGCCCTGCCGGAGCCGCAAGGCGGC 6000
pmxE_Polymyxin-P             ATGCCGCTGACGACCAACGGCAAAGTGGACCGCAAAGCGCTGCCGGAGCCGCAAGGTGGC 6060
pmxE_Polymyxin-A             ATGCCGCTGACGACCAACGGCAAAGTGGACCGCAAAGCGCTGCCGGAGCCGCAAGGCGGC 6060
                             ************ ******* ***** ** ******** *********** *****  **

pmxE-D-DaB3-Polymyxin-B      TTGCAAACGGGCGTCGAATATGTAGCGCCGCGTAACCGGACGGAGTCCCAGCTTGTGAAG 6120
pmxE_1G_Polymyxin            ttgcagacgggcgtcgaatatgtagcaccgcgtaactggacggagtccgagcttgtgaag 6120
pmxE_B-LR_Polymyxin-E        TTGCAAACGGGCGTCGAATATGTAGCGCCGCGTAACCGGACGGAGTCCCAGCTTGTGAAG 6120
pmxE_KP26_Polymyxin-E        TTGCAAACGGGCGTCGAATATGTAGCGCCGCGTAACCGGACGGAGTCCCAGCTTGTGAAG 6060
pmxE_Polymyxin-P             TTACAAACGGGCGTCGAATACGTAGCGCCGCGTAACCGGACGGAGTCCCAGCTTGTGAAG 6120
pmxE_Polymyxin-A             ATACAAACGGGTGTCGAATACGTAGCGCCGCGTAACTGGACGGAGTCCCAGCTTGTGAAG 6120
                              * ** ***** ******** ***** ********* *********** ***********

pmxE-D-DaB3-Polymyxin-B      ATCTGGGAGGAAGTGCTAGGCTACTCCGGCATTGGAGTCCTGGACAATTTCTTCGAGCTC 6180
pmxE_1G_Polymyxin            atctgggaggaagtgctgggctactccggcattggagtcctggacaatttcttcgagctc 6180
pmxE_B-LR_Polymyxin-E        ATCTGGGAGGAAGTGCTGGGTTACTCCGGCATTGGAGTCATGGACAATTTCTTCGAGCTT 6180
pmxE_KP26_Polymyxin-E        ATCTGGGAGGAAGTGCTGGGTTACTCCGGCATTGGAGTCATGGACAATTTCTTCGAGCTT 6120
pmxE_Polymyxin-P             ATCTGGGAGGAAGTGCTGGGCTACTCCGGCATTGGAGTCTTGGACAATTTCTTCGAGCTC 6180
pmxE_Polymyxin-A             ATCTGGGAGGAAGTGCTGGGTTACTCCGGCATTGGAGTCCTGGACAATTTCTTCGAGCTC 6180
                             ***************** ** ****************** *******************

pmxE-D-DaB3-Polymyxin-B      GGAGGCCACTCCTTGCGGGCGACGAACCTTGTCAGCAAGATTCAGAAGGAAATGAACGTC 6240
pmxE_1G_Polymyxin            ggaggccactccttgcgggcgacgaaccttgtcagcaagattcagaaggaaatgaacgtc 6240
pmxE_B-LR_Polymyxin-E        GGAGGCCACTCCTTGCGGGCGACGAACCTTGTCAGCAAGATTCGGAAGGAAATGAACGTC 6240
pmxE_KP26_Polymyxin-E        GGAGGCCACTCCTTGCGGGCGACGAACCTTGTCAGCAAGATTCGGAAGGAAATGAACGTC 6180
pmxE_Polymyxin-P             GGCGGCCACTCCTTGCGGGCGACGAACCTTGTCAGCAAGATTCGGAAGGAAATGAACGTC 6240
pmxE_Polymyxin-A             GGAGGCCACTCCTTGCGGGCGACGAACCTTGTCAGCAAGATTCGGAAGGAAATGAACGTC 6240
                             ** **************************************** ****************
```

```
pmxE-D-DaB3-Polymyxin-B       GAACTTCCGCTGCGCGATGTGTTCCGCTATACGACGATAGAGTCGATGGCCGGGGCTATT 6300
pmxE_1G_Polymyxin             gaacttccgctgcgcgatgtgttccgctatacgacggtagagtcgatggccggggctatt 6300
pmxE_B-LR_Polymyxin-E         GAATTTCCGCTGCGCGATGTGTTCCGCTATATGACGGTAGAGTCGATGGCCGGGGCTATT 6300
pmxE_KP26_Polymyxin-E         GAATTTCCGCTGCGCGATGTGTTCCGCTATATGACGGTAGAGTCGATGGCCGGGGCTATT 6240
pmxE_Polymyxin-P              GAACTTCCGCTGCGCGATGTGTTCCGCTGTATGACGGTAGAGTCGATGGCCGGGGCTATT 6300
pmxE_Polymyxin-A              GAACTTCCACTGCGTGATGTGTTCCGCTATACGACGGTAGAGTCGATGGCCGGGGCTATT 6300
                              *** **** ***** ************* ** **** ***********************

pmxE-D-DaB3-Polymyxin-B       GCCAGCTTGGAAGAAACGCGGCACAGCTCGATTCCGAAAGCGGAAGAGAAGGCGTACTAT 6360
pmxE_1G_Polymyxin             gccagcttggaagaaacgcggcacagctcgattccgaaagcggaagagaaagcgtactat 6360
pmxE_B-LR_Polymyxin-E         GCCAGCTTGGAGGAAACGCGGCATAGCTCGATTCCGAAAGCGGAAGAGAGAGCGTACTAT 6360
pmxE_KP26_Polymyxin-E         GCCAGCTTGGAGGAAACGCGGCATAGCTCGATTCCGAAAGCGGAAGAGAGAGCGTACTAT 6300
pmxE_Polymyxin-P              GCCAGCTTGGAAGAAACGCGGCACAGCTCGATTCCGAAAGCGGAAGAGAAAGCGTACTAT 6360
pmxE_Polymyxin-A              GCCAGCTTGGAAGAAACACAGCACAGCTCGATTCCGAAAGCGGAAGAGAAAGCGTACTAT 6360
                              *********** ***** * *** *************************  *********

pmxE-D-DaB3-Polymyxin-B       CCGGTTTCCTCCGCGCAAAAAAGGCTGTACGTCCTGCACCAGCTGGATGGCTCGGAGCTG 6420
pmxE_1G_Polymyxin             ccggtttcctccgcgcaaaaaaggctgtacgtcctgcaccagctggatagcttggagctg 6420
pmxE_B-LR_Polymyxin-E         CCGGTTTCCTCCGCACAAAAAAGGTTGTACGTCCTGAACCAGCTGGATGGCTCGGAGCTG 6420
pmxE_KP26_Polymyxin-E         CCGGTTTCCTCCGCACAAAAAAGGTTGTACGTCCTGAACCAGCTGGATGGCTCGGAGCTG 6360
pmxE_Polymyxin-P              CCGGTTTCCTCCGCGCAAAAAAGGCTGTACGTCCTGCACCAACTGGATGGCTCAGAGCTG 6420
pmxE_Polymyxin-A              CCGGTTTCCTCCGCGCAAAAAAGGCTGTACGTCCTGCACCAGCTGGATAGCTCGGAGCTG 6420
                              ************** ********* *********** **** ****** ***  ******

pmxE-D-DaB3-Polymyxin-B       AATTACAACCTCCCAAGTGCCTTGCAGTTGGAAGGGGCTTTGAACGAGGCCAAAGTGGAA 6480
pmxE_1G_Polymyxin             aattataatctcccaagcgccttgcaattgagaggagctttgaacgaggccaaagtggaa 6480
pmxE_B-LR_Polymyxin-E         AATTACAACCTCCCAAGCGCCTTGCAATTGAAAGGGGCTTTGAACGAGGCCAAAGTGGAA 6480
pmxE_KP26_Polymyxin-E         AATTACAACCTCCCAAGCGCCTTGCAATTGAAAGGGGCTTTGAACGAGGCCAAAGTGGAA 6420
pmxE_Polymyxin-P              AATTACAACCTCCCAAGCGCCTTGCAATTGGAAGGGGCTTTGAACGAGGCCAAAGTGGAA 6480
pmxE_Polymyxin-A              AATTACAACCTCCCAAGCGCCTTGCAATTGGAAGGGGCTTTGAACGAGGCCAAAGTGGAA 6480
                              ***** ** ******** ******** ***  *** ************************

pmxE-D-DaB3-Polymyxin-B       AAGGCGCTGACAACTTTGGTGGCCCGGCATGCTATGCTGCGCACCGGTTTTGAAATCGTG 6540
pmxE_1G_Polymyxin             aaggcgctgactactttggtggcccggcacgatatgctgcgcaccggatttgaaatcgtg 6540
pmxE_B-LR_Polymyxin-E         AAGGCGCTGACTACTTTGGTGGCCCGGCACGATATGCTGCGCACCGGTTTTGAAATCGTA 6540
pmxE_KP26_Polymyxin-E         AAGGCGCTGACTACTTTGGTGGCCCGGCACGATATGCTGCGCACCGGTTTTGAAATCGTA 6480
pmxE_Polymyxin-P              AAGGCGCTGACTACTTTGGTGGCCCGGCACGATATGCTGCGTACCGGTTTTGAAATCGTG 6540
pmxE_Polymyxin-A              AAGGCACTGACTACTTTGGTGGCCCGGCACGATATGCTGCGCACCGGTTTTGAAATCGTG 6540
                              ***** ***** ***************** * ********* ***** *********** 

pmxE-D-DaB3-Polymyxin-B       AATGGGGAGCCGATTCAGCGTATTCATCCGCTCGTAGCTTTCAAGGTCGAGAAGCTTCAA 6600
pmxE_1G_Polymyxin             aatggggagccggttcagcgtattcatccgctcgtagctttcaaggtcgagaagcttcaa 6600
pmxE_B-LR_Polymyxin-E         GATGGGGAGCCGGTACAGCGTATTCATCCGCTCGCAGCTTTCAAGGTCGAGAAGCTTCAA 6600
```

```
pmxE_KP26_Polymyxin-E        GATGGGGAGCCGGTACAGCGTATTCATCCGCTCGCAGCTTTCAAGGTCGAGAAGCTTCAA 6540
pmxE_Polymyxin-P             AATGGGGAGCCGGTTCAGCGTATTCATCCGCTCGTAGCTTTCAAAGTCGAGAAGCTTCAA 6600
pmxE_Polymyxin-A             AATGGGGAGCCGGTTCAGCGTATTCATCCGCTCGTAGCTTTCAAGGTCGAGAAGCTTCAA 6600
                              *********** * ******************* ********* ***************

pmxE-D-DaB3-Polymyxin-B      GCAAGCGAAGATCAGGTTGCGGCCATTCTTGAAGGCTTCATTCAGCCTTTTGACTTGACC 6660
pmxE_1G_Polymyxin            gcaagcgaagatcaggttgcggccattcttgaaggcttcattcagccttttgacttgacc 6660
pmxE_B-LR_Polymyxin-E        GCAAGTGAAGATCAGGTTGCGGCCATTCTTGAAGGCTTCATTCAGCCTTTTGACTTGACC 6660
pmxE_KP26_Polymyxin-E        GCAAGTGAAGATCAGGTTGCGGCCATTCTTGAAGGCTTCATTCAGCCTTTTGACTTGACC 6600
pmxE_Polymyxin-P             GCAAGCGAAGATCAGGTTGCGGCCATTCTTGAAGGCTTCATTCAGCCTTTTGACTTGACC 6660
pmxE_Polymyxin-A             GCAAGCGAAGATCAGGTTGCGGCCATTCTTGAAGGCTTCATTCAGCCTTTTGACTTGACC 6660
                             ***** ******************************************************

pmxE-D-DaB3-Polymyxin-B      CAGCCGCCTTTGCTGCGCGCCCTGCTGATCGAACTGGAGAAAGAGAAATTCCTGCTCGCG 6720
pmxE_1G_Polymyxin            cagccgcctttgctgcgcgccctgctgatcgaactggagaaagagaaattcctgctcgcg 6720
pmxE_B-LR_Polymyxin-E        CAGCCGCCTTTGCTGCGTGCCCTGCTGATCGAACTGGAGAAAGAGAAATTCCTGCTTGCG 6720
pmxE_KP26_Polymyxin-E        CAGCCGCCTTTGCTGCGTGCCCTGCTGATCGAACTGGAGAAAGAGAAATTCCTGCTTGCG 6660
pmxE_Polymyxin-P             CAGCCGCCTTTGCTGCGCGCCCTACTGATCGAACTGGAGAAAGAGAAATTTCTGCTCGCG 6720
pmxE_Polymyxin-A             CAGCCGCCTTTGCTGCGCGCCCTGCTGATCGAACTGGAGAAAGAGAAATTTCTGCTCGCG 6720
                             ***************** ***** ************************** ***** ***

pmxE-D-DaB3-Polymyxin-B      CTGGATATTCATCATATTGGTTCCGACGGCCTTTCCATGGACGTGCTGCTGCGCGAATTC 6780
pmxE_1G_Polymyxin            ctggatattcatcatattggttccgacggcctttccatggacgtgctgctgcgtgaattc 6780
pmxE_B-LR_Polymyxin-E        CTGGATATTCATCATATTGGTTCCGACGGCCTCTCCATGGACGTGCTGCTGCGCGAATTC 6780
pmxE_KP26_Polymyxin-E        CTGGATATTCATCATATTGGTTCCGACGGCCTCTCCATGGACGTGCTGCTGCGCGAATTC 6720
pmxE_Polymyxin-P             CTGGATATTCATCATATTGGTTCCGACGGCCTTTCCATGGACGTGCTGCTGCGCGAATTC 6780
pmxE_Polymyxin-A             CTGGATATTCATCATATTGGTTCCGACGGCCTTTCCATGGACGTGCTGCTGCGCGAATTC 6780
                             ******************************** ******************** ******

pmxE-D-DaB3-Polymyxin-B      GTGCGGCTTTACAATGGGGAAGAATTGCCGGAGCTGCGGATTCAATATAAGGATTACGCC 6840
pmxE_1G_Polymyxin            gtgcggctttacaatggggaagaattgccggagctgcggattcaatacaaggattacgcc 6840
pmxE_B-LR_Polymyxin-E        GTGCGGCTTTACAATGGGGAAGAATTGCCGGTGCTACGGATTCAATACAAGGATTACGCC 6840
pmxE_KP26_Polymyxin-E        GTGCGGCTTTACAATGGGGAAGAATTGCCGGTGCTACGGATTCAATACAAGGATTACGCC 6780
pmxE_Polymyxin-P             GTGCGGCTTTACAATGGGGAGGAATTGCCGGAGCTGCGGATTCAATACAAGGATTACGCC 6840
pmxE_Polymyxin-A             GTGCGGCTTTACAATGGGGAGGAATTGCCGGAGCTGCGGATTCAATACAAGGATTACGCC 6840
                             ******************** ********** *** *********** ************

pmxE-D-DaB3-Polymyxin-B      GTTTGGCAGCAATCCGAGGAGCAGCGCCAGCGCATCAAACGGCAGGAGGAATACTGGCGC 6900
pmxE_1G_Polymyxin            gtttggcagcaatccgaggagcagcggcagcgcatcaaacggcaggaggaatactggcgc 6900
pmxE_B-LR_Polymyxin-E        GTTTGGCAGCAATCCGAGGAACAGCGCCAGCGTATCAAACAGCAGGAGGAATACTGGCGT 6900
pmxE_KP26_Polymyxin-E        GTTTGGCAGCAATCCGAGGAACAGCGCCAGCGTATCAAACAGCAGGAGGAATACTGGCGT 6840
pmxE_Polymyxin-P             GTTTGGCAGCAATCCGAGGAGCAGCGCCAGCGCATCAAACGGCAGGAGGAATACTGGCGC 6900
pmxE_Polymyxin-A             GTTTGGCAGCAATCCGAGGAGCAGCGCCAGCGCATCAAACGGCAGGAGGAATACTGGCGC 6900
                             ******************** ***** ***** ******* ***************** 
```

```
pmxE-D-DaB3-Polymyxin-B      GGGGTATTCAGCTCCGAGCTTCCGGTTCTTGAGCTGCCTCTCGACTTCTCCCGCCCGGCC 6960
pmxE_1G_Polymyxin            ggggtattcaactccgagcttccggttcttgagctgcctctcgacttctcccgcccggct 6960
pmxE_B-LR_Polymyxin-E        GGGGTATTCAACTCTGAGCTTCCGGTTCTTGAGCTGCCTCTCGACTTCTCCCGTCCGGCC 6960
pmxE_KP26_Polymyxin-E        GGGGTATTCAACTCTGAGCTTCCGGTTCTTGAGCTGCCTCTCGACTTCTCCCGTCCGGCC 6900
pmxE_Polymyxin-P             GGGGTATTCAGCTCCGAGCTTCCGGTTCTTGAGCTGCCTCTCGACTTCTCCCGCCCGGCC 6960
pmxE_Polymyxin-A             GGGGTATTCAGCTCCGAGCTTCCGGTTCTTGAGCTGCCTCTCGACTTCTCCCGCCCGGCC 6960
                             ********** *** ************************************** *****

pmxE-D-DaB3-Polymyxin-B      GTCCAGCAGTTTGACGGTCAAACGCTCACGTTTACGCTGGATGCGGAGAAAAGCGAAGCT 7020
pmxE_1G_Polymyxin            gtccagcagtttgacggtcaaacgctcacgtttacgctggatgcggagaaaagcgaagct 7020
pmxE_B-LR_Polymyxin-E        GTCCAGCAGTTTGACGGTCGAACGCTCACGTTTACGCTGGATGCGGAGAAAAGCGAAGCT 7020
pmxE_KP26_Polymyxin-E        GTCCAGCAGTTTGACGGTCGAACGCTCACGTTTACGCTGGATGCGGAGAAAAGCGAAGCT 6960
pmxE_Polymyxin-P             GTTCAGCAGTTTGACGGTCAAACGCTCACGTTTACGCTGGATGCGGAGAAAAGCGAAGCT 7020
pmxE_Polymyxin-A             GTTCAGCAGTTTGACGGTCAAACGCTCACGTTTACGCTGGATGCGGAGAAAAGCGAAGCT 7020
                             ** **************** ****************************************

pmxE-D-DaB3-Polymyxin-B      CTCAAACGGCTTGCCGGCGATTCGGGGGCGACGCTGTATATGCTTCTGCTGGCTGCGTAC 7080
pmxE_1G_Polymyxin            ctcaaacggcttgccggcgattcgggagcgacgctgtacatgcttctgctggctgcgtac 7080
pmxE_B-LR_Polymyxin-E        CTCAAACGGCTTGCCGGCGATTCGGGAGCGACGCTTTACATGCTTTTGCTGGCTGCGTAC 7080
pmxE_KP26_Polymyxin-E        CTCAAACGGCTTGCCGGCGATTCGGGAGCGACGCTTTACATGCTTTTGCTGGCTGCGTAC 7020
pmxE_Polymyxin-P             CTCAAACGGCTTGCCGGCGATTCGGGGGCGACGCTGTACATGCTTCTGCTAGCTGCGTAC 7080
pmxE_Polymyxin-A             CTCAAACGGCTTGCCGGCGATTCGGGGGCGACGCTGTACATGCTTCTGCTGGCTGCGTAC 7080
                             ************************** ******** ** ****** **** *********

pmxE-D-DaB3-Polymyxin-B      TCCGTATTGCTTCATAAATACGCGGGACAAGAAGATATAGTGGTCGGCACTCCGATTGCC 7140
pmxE_1G_Polymyxin            tccgtattgcttcataaatacgcgggacaggaagatatagtggtcggcactccgattgcc 7140
pmxE_B-LR_Polymyxin-E        TCCGTATTGCTTCATAAATATGCGGGACAGGAAGATATCGTGGTCGGAACTCCGATTGCC 7140
pmxE_KP26_Polymyxin-E        TCCGTATTGCTTCATAAATATGCGGGACAGGAAGATATCGTGGTCGGAACTCCGATTGCC 7080
pmxE_Polymyxin-P             TCCGTATTGCTTCATAAATACGCGGGACAGGAAGATATAGTGGTTGGCACTCCGATTGCC 7140
pmxE_Polymyxin-A             TCCGTATTGCTTCATAAATACGCGGGACAGGAAGATATAGTGGTCGGCACTCCGATTGCC 7140
                             ******************** ******** ******** ***** ** ************

pmxE-D-DaB3-Polymyxin-B      GCCCGTTCTCACGCCGACCTGCAGCCGATTATCGGCATGTTCGTCAATACGCTGGCCCTT 7200
pmxE_1G_Polymyxin            gcccgttctcacgccgacctgcagccgattatcggcatgttcgtcaatacgctggccctt 7200
pmxE_B-LR_Polymyxin-E        GCCCGATCTCACACTGACTTACAGCCGATTATCGGCATGTTCGTCAATACGCTTGCCCTT 7200
pmxE_KP26_Polymyxin-E        GCCCGATCTCACACTGACTTACAGCCGATTATCGGCATGTTCGTCAATACGCTTGCCCTT 7140
pmxE_Polymyxin-P             GCCCGTTCTCACGCCGACCTGCAGCCGATTATCGGCATGTTCGTCAATACGCTGGCCCTT 7200
pmxE_Polymyxin-A             GCCCGTTCTCACGCCGACCTGCAGCCGATTATCGGCATGTTCGTCAATACGCTGGCCCTT 7200
                             ***** ****** * *** * ******************************** ******

pmxE-D-DaB3-Polymyxin-B      CGCTTGGGTCCGGCGGCGGAGCGGACGTTCCTGGATTACTTGCAGGAAGTGAAGGAAACG 7260
pmxE_1G_Polymyxin            cgcttgggtccggcggcggagcggacgttcctggattacttgcaggaagtgaaggaaacg 7260
pmxE_B-LR_Polymyxin-E        CGCTTGGGACCGGCGGCGGAGCGGACGTTCCTGGATTACTTACAGGAAGTGAAGGAAACG 7260
```

```
pmxE_KP26_Polymyxin-E        CGCTTGGGACCGGCGGCGGAGCGGACGTTCCTGGATTACTTACAGGAAGTGAAGGAAACG 7200
pmxE_Polymyxin-P             CGCTTGGCCCCGGTGGCGGAGCGGACGTTCCTGGATTACTTGCAGGAAGTGAAGGAAACG 7260
pmxE_Polymyxin-A             CGTTTGCGTCCGGCGGCGGAGCGGACGTTCCTGGATTACTTGCAGGAAGTGAAGGAAACG 7260
                             ** ***   **** *************************** ******************

pmxE-D-DaB3-Polymyxin-B      ACGCTTGGAGCCTACGAGCACCAGGACTATCCGTTCGAGGAGCTGGTGGAAGCTCTTCAG 7320
pmxE_1G_Polymyxin            acgcttggagcctacgagcaccaggactatccgttcgaggagctggtggaagctcttcag 7320
pmxE_B-LR_Polymyxin-E        ACGCTAGGAGCCTACGAGCACCAGGACTATCCGTTTGAGGAGCTGGTGGAAGCTCTTCAG 7320
pmxE_KP26_Polymyxin-E        ACGCTAGGAGCCTACGAGCACCAGGACTATCCGTTTGAGGAGCTGGTGGAAGCTCTTCAG 7260
pmxE_Polymyxin-P             ACGCTTGGAGCCTACGAGCACCAGGACTATCCGTTCGAGGAGCTGGTGGAAGCTCTTCAG 7320
pmxE_Polymyxin-A             ACGCTTGGAGCCTACGAGCACCAGGACTATCCGTTCGAGGAGTTGGTGGAAGCTCTTCAG 7320
                             ***** ***************************** ****** *****************

pmxE-D-DaB3-Polymyxin-B      GTGAGCCGGGATTTAAGCCGGAATCCTCTGTTTGACACCATGTTTTCTTTGCAAAAACAC 7380
pmxE_1G_Polymyxin            gtgagccgggatttaagccggaatcctctgtttgacaccatgttttctttgcaaaagcac 7380
pmxE_B-LR_Polymyxin-E        GTGAGCCGGGATTTAAGCCGGAATCCGCTGTTTGACACCATGTTTTCTTTGCAAAAGCAC 7380
pmxE_KP26_Polymyxin-E        GTGAGCCGGGATTTAAGCCGGAATCCGCTGTTTGACACCATGTTTTCTTTGCAAAAGCAC 7320
pmxE_Polymyxin-P             GTGAGCCGGGATTTAAGCCGGAATCCTCTGTTTGACACCATGTTTTCTTTGCAAAAGCAC 7380
pmxE_Polymyxin-A             GTGAGCCGGGATTTAAGCCGGAATCCTCTGTTTGACACCATGTTTTCTTTGCAAAAGCAC 7380
                             ************************** ***************************** ***

pmxE-D-DaB3-Polymyxin-B      GAAAGCTTGGATTTAACCCTGGAAGGCTTGCAATGGTCGCTGTTCGACATCGAGGAAAAG 7440
pmxE_1G_Polymyxin            gaaagcttggatttaaccctggaaggcttgcaatggtcgctgttcgacattgaggaaaag 7440
pmxE_B-LR_Polymyxin-E        GAAAGCTTGGATTTAACCCTGGAAGGCTTGCAATGGTCGCTGTTCGACATCGAGGAAAAG 7440
pmxE_KP26_Polymyxin-E        GAAAGCTTGGATTTAACCCTGGAAGGCTTGCAATGGTCGCTGTTCGACATCGAGGAAAAG 7380
pmxE_Polymyxin-P             GAAAGCTTGGATTTACCCCTGGAAGGTTTGAAATGGTCGCTGTTCGACATCGAGGAAAAG 7440
pmxE_Polymyxin-A             GAAAGCTTGGATTTAACCCTGGAAGGCTTGCAATGGTCGCTGTTCGACATCGAGGAAAAG 7440
                             *************** ********** *** ******************* *********

pmxE-D-DaB3-Polymyxin-B      ACGGCAAAGTTTGATCTTAGCCTTGATATCGTGGAAGCCGATAACGAGCTGGTTTGCAAG 7500
pmxE_1G_Polymyxin            acggcaaagtttgatcttagctttgatatcgtggaagccgataacgagctggtttgcaag 7500
pmxE_B-LR_Polymyxin-E        ACGGCAAAGTTTGATCTTAGCTTTGATATCGTGGAAGCCGATAACGAGTTGGTTTGCAAG 7500
pmxE_KP26_Polymyxin-E        ACGGCAAAGTTTGATCTTAGCTTTGATATCGTGGAAGCCGATAACGAGTTGGTTTGCAAG 7440
pmxE_Polymyxin-P             ACGGCAAAGTTTGATCTTAGCTTTGATATCGTGGAAGCTGATAACGAGCTGGTTTGCAAG 7500
pmxE_Polymyxin-A             ACGGCCAAGTTTGATCTTAGCTTTGATATCGTGGAAGCCGATAACGAGCTGGTTTGCAAG 7500
                             ***** *************** **************** ********* ***********

pmxE-D-DaB3-Polymyxin-B      ATCGAGTACGCTACCTCGTTGTTTAGACAGGAAACAATGGTACGGCTGGCGGGTCATTAC 7560
pmxE_1G_Polymyxin            atcgagtacgctacctcgttgtttagacaggaaacgatggtacggctggcgggtcattac 7560
pmxE_B-LR_Polymyxin-E        ATCGAGTACGCTACCTCGTTGTTTAGACAGGAAACGATGGTACGGCTGGCGGGTCATTAC 7560
pmxE_KP26_Polymyxin-E        ATCGAGTACGCTACCTCGTTGTTTAGACAGGAAACGATGGTACGGCTGGCGGGTCATTAC 7500
pmxE_Polymyxin-P             ATCGAGTACGCTACCTCGTTGTTTAGACAGGAAACGATGGTACGGCTGGCGGGTCATTAC 7560
pmxE_Polymyxin-A             ATTGAGTACGCTACCTCGTTGTTTAGACAGGAAACGATGGTACGGCTGGCGGGACATTAT 7560
                             ** ******************************** ***************** *****
```

```
pmxE-D-DaB3-Polymyxin-B      GAGCAGCTATTGGCGTCGATCCTGGCTCAGCCGGGTGCGCGGATTTCGGATTTGGATATC 7620
pmxE_1G_Polymyxin            gagcagcttttggcgtcgatcctggctcagccgggtgcgcggatttcggatttggatatc 7620
pmxE_B-LR_Polymyxin-E        GAGCAGCTTTTGGCGTCGATCCTGGCTCAGCCGGGTGCGCGGATTTCGGATTTGGACATA 7620
pmxE_KP26_Polymyxin-E        GAGCAGCTTTTGGCGTCGATCCTGGCTCAGCCGGGTGCGCGGATTTCGGATTTGGACATA 7560
pmxE_Polymyxin-P             GAGCAGCTTTTGGCGTCGATCCTGGCTCAGCCGGGTGCGCGGATTTCGGATTTGGATATC 7620
pmxE_Polymyxin-A             GAGCAGCTTTTGGCGTCCATCCTGGCTCAGCCGGGTGCGCGGATTTCGGATTTGGATATC 7620
                             ******** ******** ************************************** **

pmxE-D-DaB3-Polymyxin-B      TTGACGGACAGCGAAAAGCATGATTTGCTGATCGGGTTTGACGTGTCGTCTTCGGCTCTT 7680
pmxE_1G_Polymyxin            ttgacggacagcgaaaagcatgatttcctggtcgagtttgacgtgtcgtctccggctctt 7680
pmxE_B-LR_Polymyxin-E        TTGACGGACAGCGAAAAGCATGATTTGCTGGTCGGGTTTGACGTGTCGTCTTCGGCTCTT 7680
pmxE_KP26_Polymyxin-E        TTGACGGACAGCGAAAAGCATGATTTGCTGGTCGGGTTTGACGTGTCGTCTTCGGCTCTT 7620
pmxE_Polymyxin-P             TTGACGGACAGCGAAAAGCATGATTTGCTGGTCGGGTTTGACGTGTCGTCTTCGGCTCTT 7680
pmxE_Polymyxin-A             TTGACGGACAGCGAAAAGCATGATTTGCTGGTCGGGTTTGACGTGTCGTCTTCGGCTCTT 7680
                             ************************** *** *** **************** ********

pmxE-D-DaB3-Polymyxin-B      GCGAAGCAACACGCCGCAGAGGGTACAGGTTTGGAAGCGGATGAGTCGTGGAGAGAGAGA 7740
pmxE_1G_Polymyxin            gcgaagcaacccgccgcagagggtacaggtttggaggcggatgagtcgtggagagagaga 7740
pmxE_B-LR_Polymyxin-E        GCGAAGCAATCCGCCGCAGAGGGTACAGGTTTGGAAGCGGATGAATCGTGGAGAGAGAGG 7740
pmxE_KP26_Polymyxin-E        GCGAAGCAATCCGCCGCAGAGGGTACAGGTTTGGAAGCGGATGAATCGTGGAGAGAGAGG 7680
pmxE_Polymyxin-P             GCGAAGCAACCCGCCGCAGAGGGTACAGGTTTGGAAGCGGATGAGTCGTGGAGAGAGAGA 7740
pmxE_Polymyxin-A             GCGAAGCAACCCGCCGCAGAAGGTACAGGTTTGGAAGCGGATGAGTCGTGGAGAGAGAGA 7740
                             *********  ********* ************** ******** ************** 

pmxE-D-DaB3-Polymyxin-B      ACGTTCCACGAGCTGTTTGAGGAGCAGGCGGAACGCACTCCGGGAGCGCTGGCGGTTGTC 7800
pmxE_1G_Polymyxin            acgttccacgagctgttcgaggagcaggcggagcgcactccgggagcgttggcggttgtc 7800
pmxE_B-LR_Polymyxin-E        ACGTTCCACGAGCTGTTCGAGGAGCAGGCGGAGCGCACTCCTGGAGCGCTGGCTGTTATC 7800
pmxE_KP26_Polymyxin-E        ACGTTCCACGAGCTGTTCGAGGAGCAGGCGGAGCGCACTCCTGGAGCGCTGGCTGTTATC 7740
pmxE_Polymyxin-P             ACGTTCCACGAGCTGTTCGAGGAGCAGGCGGAGCGCACTCCGGGAGCGCTGGCGGTTGTC 7800
pmxE_Polymyxin-A             ACGTTCCACGAGCTGTTCGAGGAGCAGGCGGAGCGCACTCCGGGAGCGCTGGCGGTTGTC 7800
                             ***************** ************** ******** ****** **** *** **

pmxE-D-DaB3-Polymyxin-B      TACGAAGACAGCAAGCTGACGTATGCGGAGCTGAACGCCAAAGCGAATCGTCTGGCGTAT 7860
pmxE_1G_Polymyxin            tacgaagacagtaagctgacgtatgcggagctgaacgccaaagcgaatcgtctggcgtat 7860
pmxE_B-LR_Polymyxin-E        TACGAAGACAGCAAGCTGACGTATGCGGAGCTGAACGCCAAAGCGAATCGTCTGGCGTAT 7860
pmxE_KP26_Polymyxin-E        TACGAAGACAGCAAGCTGACGTATGCGGAGCTGAACGCCAAAGCGAATCGTCTGGCGTAT 7800
pmxE_Polymyxin-P             TACGAAGACAGCAAGCTGACGTATGCGGAGCTGAACGCCAAAGCGAATCGTCTGGCGTTT 7860
pmxE_Polymyxin-A             TACGAAGACAGCAAGCTGACGTATGCGGAGCTGAACGCCAAAGCGAATCGTCTGGCGTAT 7860
                             *********** ********************************************* *

pmxE-D-DaB3-Polymyxin-B      GCACTGCGGGCGCGCGGGGTGAAGCCGGAGCAGGTGGTCGGCATTCTGGCTGGCCGTTCG 7920
pmxE_1G_Polymyxin            gcactgcgggcgcgcggggtgaagccggaacaggtggtcggcattctggccgaccgttcg 7920
pmxE_B-LR_Polymyxin-E        GCACTGCGGGCGCGCGGGGTGAAGCCGGAGCAGGTGGTCGGCATTCTGGCCGGCCGTTCG 7920
```

```
pmxE_KP26_Polymyxin-E        GCACTGCGGGCGCGCGGGGTGAAGCCGGAGCAGGTGGTCGGCATTCTGGCCGGCCGTTCG 7860
pmxE_Polymyxin-P             GCACTGCGGGAGCGCGGGGTGAAGCCGGAGCAGGTGGTCGGCATTCTGGCCGGCCGTTCG 7920
pmxE_Polymyxin-A             GCACTGCGGGCGCGCGGTGTGAAGCCGGAGCAGGTGGTCGGCATTCTGGCCGGCCGTTCA 7920
                             ********** ****** *********** ******************* * ******

pmxE-D-DaB3-Polymyxin-B      GCGGAGCTGTTGATCGGGGTGCTCGCCGTATGGAAAGCGGGCGGCGCTTATGTGCCGCTC 7980
pmxE_1G_Polymyxin            gcggagctgttgatcggggtgctcgccgtatggaaagcgggcggcgcttatgtgccgctc 7980
pmxE_B-LR_Polymyxin-E        GCGGAGCTGTTGATCGGGGTGCTCGCCGTATGGAAAGCGGGTGGCGCTTATGTGCCGCTT 7980
pmxE_KP26_Polymyxin-E        GCGGAGCTGTTGATCGGGGTGCTCGCCGTATGGAAAGCGGGTGGCGCTTATGTGCCGCTT 7920
pmxE_Polymyxin-P             GCGGAGCTGTTGATCGGGGTGCTCGCCGTATGGAAAGCGGGTGGCGCTTATGTGCCGCTC 7980
pmxE_Polymyxin-A             GCAGAGCTGTTGGTTGGTGTGCTTGCCGTATGGAAAGCGGGCGGCGCTTATGTGCCGCTC 7980
                             ** ********* * ** ***** ***************** *****************

pmxE-D-DaB3-Polymyxin-B      GACCCGGACTATCCGGCGGAGCGGATCGAGTATATGCTCGCGGACAGCGGGGCGTCGGTG 8040
pmxE_1G_Polymyxin            gacccggactatccggcggagcggatcgagtatatgctcgcggacagcggggcgtcggtg 8040
pmxE_B-LR_Polymyxin-E        GATCCGGACTATCCGGCGGAGCGGATCGAGTATATGCTCACGGACAGCGGGGCGTCGGTT 8040
pmxE_KP26_Polymyxin-E        GATCCGGACTATCCGGCGGAGCGGATCGAGTATATGCTCACGGACAGCGGGGCGTCGGTT 7980
pmxE_Polymyxin-P             GACCCGGACTATCCGGCGGAGCGGATCGAGTATATGCTCGCGGACAGCGGGGCGTCGGTG 8040
pmxE_Polymyxin-A             GACCCGGATTATCCGGCGGAACGGATCGAATATATGCTCGCGGACAGCGGGGCGTCGGTG 8040
                             ** ***** *********** ******** ********* *******************

pmxE-D-DaB3-Polymyxin-B      CTGCTCACACAGACCCGCCTGCTGGAGCAGGCGGAAGCTTGGCGCAGCGACGGAGCTCTA 8100
pmxE_1G_Polymyxin            ctgctcacgcagacccgcctgctggagcaggcggaagcttggcgcagcgacggagctcta 8100
pmxE_B-LR_Polymyxin-E        CTGCTCACGCAGACCCGCCTGCTGGAGCAGGCGGAAGTTTGGCGCAGCGACGGAGCTCTA 8100
pmxE_KP26_Polymyxin-E        CTGCTCACGCAGACCCGCCTGCTGGAGCAGGCGGAAGTTTGGCGCAGCGACGGAGCTCTA 8040
pmxE_Polymyxin-P             CTGCTCACGCAGACCCGCCTGCTGGAGCAGGCGGAAGCTTGGTGCAGCGACGGAGCTCTA 8100
pmxE_Polymyxin-A             CTGCTCACGCAGACCTGCCTGCTGGAGCAGGCGGAAGCTTGGCGCAGCGACGGAGCTCTA 8100
                             ******** ****** ********************* **** *****************

pmxE-D-DaB3-Polymyxin-B      GTGCTGCAAACGGTGCTTGCGCTTGACGACGCCGCGACGTACAGCCTCGG---------A 8151
pmxE_1G_Polymyxin            gtgctgcaaacggtgcttgcgcttgacgacgccgcgacgtacagcctcggagcggcggaa 8160
pmxE_B-LR_Polymyxin-E        GCGTTGCAAACGGTGCTTGCACTTGACGACGCTGCGACGTACAGTCTCGGAGCGGCAGAA 8160
pmxE_KP26_Polymyxin-E        GCGTTGCAAACGGTGCTTGCACTTGACGACGCTGCGACGTACAGTCTCGGAGCGGCAGAA 8100
pmxE_Polymyxin-P             GTGCTGCAAACGGTGCTTGCGCTTGACGACGCCGCGACGTACAGCCTCGGCGCGTCGGAA 8160
pmxE_Polymyxin-A             GTGCTGCAAACGGTGCTTGCGCTTGACGACGCCGCGACGTACAGCCTCGGAGCGGCGGAA 8160
                             * * **************** *********** *********** *****         *

pmxE-D-DaB3-Polymyxin-B      GTGGCTG---------------TGGGCGAAGCAGGCGCAGAGGCGGAGGCTTTGGCGCAA 8196
pmxE_1G_Polymyxin            gcggctg---------------tgggcgaagcaggcgcagaggcggaggctttggcgcaa 8205
pmxE_B-LR_Polymyxin-E        GTGGCTGTGGGCGTACAAGCTTTGGGCGAAGCAGGCGCAGAGGCGGAGGCTTTGGCGCAA 8220
pmxE_KP26_Polymyxin-E        GTGGCTGTGGGCGTACAAGCTTTGGGCGAAGCAGGCGCAGAGGCGGAGGCTTTGGCGCAA 8160
pmxE_Polymyxin-P             GCGGCTGTGGGCGTACAAGCTTTGGGCGAAGCAGGCGCAGAGGCGGAGGCTTTGGCGCAA 8220
pmxE_Polymyxin-A             GCGGCTGTGGGCGTACAAGCTTTGGGTGAAGCAGGCGCAGAGGCGGAGGCTTTGGCGCAA 8220
                             * *****               **** *********************************
```

```
pmxE-D-DaB3-Polymyxin-B      GCGGAAACGGCTTCTGCCGAAACGTCCGCCACGGCAGAAGCCGAGAAGAACGTACTAGCG 8256
pmxE_1G_Polymyxin            gcggaaacggctgccgccgaaacgtccgccacggcagaagccgagaagaacgtactagcg 8265
pmxE_B-LR_Polymyxin-E        GCGCAAACGGCTGCTGCCGAGACGTCCGCCACGGCAGAAGCCGAGCAGAACGTACTGGCG 8280
pmxE_KP26_Polymyxin-E        GCGCAAACGGCTGCTGCCGAGACGTCCGCCACGGCAGAAGCCGAGCAGAACGTACTGGCG 8220
pmxE_Polymyxin-P             GCGGAAACGGCTGCTGCCGAAACGTCCGCCACGGCAGAAGCCGAGAAGAACGTACTAGCG 8280
pmxE_Polymyxin-A             GCGGAAACGGCTGCTGCCGAAAAATCCGCCACGGCAGAAGCCGAGAAGAACGTACTAGCG 8280
                             *** ******** * ***** *  ********************* ********** ***

pmxE-D-DaB3-Polymyxin-B      GCGGATCTCGCATCGAATCCGGCGAATGTGAACAAGCCGCGCGATTTGGCTTATGTCATC 8316
pmxE_1G_Polymyxin            gcggatctcgcatcgaatccggcgaatgtgaacaagccgcgcgatttggcttacgtcatc 8325
pmxE_B-LR_Polymyxin-E        GCGGATCTCGCATCGAATCCGGCGAATGTGAACAAGCCGCGCGATTTGGCTTACGTCATC 8340
pmxE_KP26_Polymyxin-E        GCGGATCTCGCATCGAATCCGGCGAATGTGAACAAGCCGCGCGATTTGGCTTACGTCATC 8280
pmxE_Polymyxin-P             GCGGATCTCGCATCGAATCCGGCGAATGTGAACAAGCCGCGCGATTTGGCTTACGTGATC 8340
pmxE_Polymyxin-A             GCGGATCTCGCATCGAATCCGGCGAATGTGAACAAGCCGAGCGATTTGGCTTACGTCATC 8340
                             *************************************** ************* ** ***

pmxE-D-DaB3-Polymyxin-B      TACACCTCCGGTACGACGGGCCGTCCGAAGGGCGTGGCGGTGGAAAACCGCAGCCTGGTG 8376
pmxE_1G_Polymyxin            tacacctccggtacgacgggccgtccgaagggcgtggcggtggaaaaccgcagcctggtg 8385
pmxE_B-LR_Polymyxin-E        TACACCTCCGGTACGACTGGCCGTCCGAAGGGTGTGGCGGTGGAACACCGCAGCCTGGTG 8400
pmxE_KP26_Polymyxin-E        TACACCTCCGGTACGACTGGCCGTCCGAAGGGTGTGGCGGTGGAACACCGCAGCCTGGTG 8340
pmxE_Polymyxin-P             TATACGTCGGGAACGACGGGCAAGCCGAAGGGCGTGATGATCGAGCACCGCAGCCTGGTG 8400
pmxE_Polymyxin-A             TACACCTCCGGTACGACGGGCCGTCCGAAGGGCGTGGCGGTGGAACACCGCAGCTTGGTG 8400
                             ** ** ** ** ***** ***   ******** ***  * * **  ******** *****

pmxE-D-DaB3-Polymyxin-B      AACACGGCGGCGGGCTACCGGCGGGACTACCGCCTGGATCAGTTCCCGATCCGGCTGCTG 8436
pmxE_1G_Polymyxin            aacacggcggcgggctaccggcgggactaccgcctggatcagttcccgatccggctgctg 8445
pmxE_B-LR_Polymyxin-E        AACACGGCGGCGGGCTATCGGCGGGACTACCGCCTGGATCAGTTCCCGATCCGGCTGCTG 8460
pmxE_KP26_Polymyxin-E        AACACGGCGGCGGGCTATCGGCGGGACTACCGCCTGGATCAGTTCCCGATCCGGCTGCTG 8400
pmxE_Polymyxin-P             AACACGGCGGCGGGCTACCGGCGGGAATACCGCCTGGATCAGTTCCCGATCCGGCTGCTG 8460
pmxE_Polymyxin-A             AACACGGCGGCGGGCTACCGGCGGGACTACCGCCTGGATCAGTTCCCGATCCGGCTGCTG 8460
                             ***************** ******** *********************************

pmxE-D-DaB3-Polymyxin-B      CAGCTCGCCAGCTTCTCGTTCGACGTGTTCGTCGGCGACATTGCGCGGACGCTGTACAAC 8496
pmxE_1G_Polymyxin            cagctcgccagcttctcgttcgacgtgttcgtcggcgacattgcgcggacgctgtacaac 8505
pmxE_B-LR_Polymyxin-E        CAACTCGCCAGCTTCTCGTTTGACGTGTTCGTCGGCGACATTGCGCGGACGCTGTACAAC 8520
pmxE_KP26_Polymyxin-E        CAACTCGCCAGCTTCTCGTTTGACGTGTTCGTCGGCGACATTGCGCGGACGCTGTACAAC 8460
pmxE_Polymyxin-P             CAGCTCGCCAGCTTCTCGTTCGACGTGTTCGTCGGCGACATTGCGCGGACGCTGTACAAC 8520
pmxE_Polymyxin-A             CAGCTCGCCAGCTTCTCGTTTGATGTGTTCGTCGGTGATATTGCGCGGACGCTGTACAAC 8520
                             ** ***************** ** *********** ** *********************

pmxE-D-DaB3-Polymyxin-B      GGCGGCACCATGGTCATCGTGCCGAAGGACGACCGGATTGATCCAACCCGCCTCTACGGC 8556
pmxE_1G_Polymyxin            ggcggcaccatggtcatcgtgccgaaggacgaccggattgatccaacccgcctctacggc 8565
pmxE_B-LR_Polymyxin-E        GGCGGCACCATGGTCATCGTGCCGAAGGACGACCGGATTGATCCAACCCGCCTATACGGC 8580
```

```
pmxE_KP26_Polymyxin-E        GGCGGCACCATGGTCATCGTGCCGAAGGACGACCGGATTGATCCAACCCGCCTATACGGC 8520
pmxE_Polymyxin-P             GGGGGCACCATGGTCATCGTGCCGAAGGACGACCGGATTGATCCAACCCGCCTCTACGGC 8580
pmxE_Polymyxin-A             GGCGGCACTATGGTCATCGTGCCGAAGGACGACCGGATTGATCCAACCCGCCTCTACGGC 8580
                             ** ***** ******************************************** ******

pmxE-D-DaB3-Polymyxin-B      TGGATTCGCGACTACGCTGTGACGGTGTTCGAATCGACTCCTGCGCTGATCGTGCCGTTC 8616
pmxE_1G_Polymyxin            tggattcgcgactacgccgtgacggtgttcgaatcgactccggcgctgatcgtgccgttc 8625
pmxE_B-LR_Polymyxin-E        TGGATTCGCGACTACGCCGTGACGGTGTTCGAATCGACTCCGGCGCTGATCGTGCCGTTC 8640
pmxE_KP26_Polymyxin-E        TGGATTCGCGACTACGCCGTGACGGTGTTCGAATCGACTCCGGCGCTGATCGTGCCGTTC 8580
pmxE_Polymyxin-P             TGGATTCGCGACTACGCCGTGACGGTGTTCGAATCGACTCCGGCGCTGATCGTGCCGTTC 8640
pmxE_Polymyxin-A             TGGATTCGCGACTACGCCGTGACGGTGTTCGAATCGACTCCGGCGCTGATCGTGCCGTTC 8640
                             ***************** *********************** ******************

pmxE-D-DaB3-Polymyxin-B      ATGGAGCATGTGCATGCCGAGGGTCTGGACCTCAGCTCGATGCAGCTGCTGATCACAAGC 8676
pmxE_1G_Polymyxin            atggagcatgtgcatgccgagggtctggacctcagctcgatgcagctgctgatcacaagc 8685
pmxE_B-LR_Polymyxin-E        ATGGAGCATGTGTATGCCGAGGGTCTGGATCTCAGCTCGATGCAGTTGCTGCTCACAAGC 8700
pmxE_KP26_Polymyxin-E        ATGGAGCATGTGTATGCCGAGGGTCTGGATCTCAGCTCGATGCAGTTGCTGCTCACAAGC 8640
pmxE_Polymyxin-P             ATGGAGCATGTGCATGCCGAGGGTCTGGACCTCAGCTCGATGCAGCTGCTGATCACAAGC 8700
pmxE_Polymyxin-A             ATGGAGCATGTGCATGCCGAGGGTCTGGATCTCAGCTCGATGCAGCTGCTGATCACAAGC 8700
                             ************ **************** *************** ***** ********

pmxE-D-DaB3-Polymyxin-B      TCGGATGCGTGCAGCGTAGCGGATTACCGCACCTTGCAGGAGCGCTTCGGCTCGCAGTTC 8736
pmxE_1G_Polymyxin            tcggatgcgtgcagcgtagcggattaccgcaccttgcaggagcgcttcggctcgcagttc 8745
pmxE_B-LR_Polymyxin-E        TCGGATGCGTGCAGCGTAGCGGATTACCGCACCTTGCAGGAGCGCTTCGGCTCGCAGTTC 8760
pmxE_KP26_Polymyxin-E        TCGGATGCGTGCAGCGTAGCGGATTACCGCACCTTGCAGGAGCGCTTCGGCTCGCAGTTC 8700
pmxE_Polymyxin-P             TCGGATGCGTGCAGCGTAGCGGATTACCGCACCTTGCAGGAGCGCTTCGGCTCGCAGTTC 8760
pmxE_Polymyxin-A             TCGGATGCGTGCAGCGTAGCGGATTACCGCACCTTGCAGGAGCGCTTCGGCTCGCAGTTC 8760
                             ************************************************************

pmxE-D-DaB3-Polymyxin-B      CGTATTATTAACAGCTACGGCGTAACGGAAGCGGCGATTGACTCCAGCTTCTATGACGAG 8796
pmxE_1G_Polymyxin            cgtattattaacagctacggcgtaacggaagcggcgattgactccagcttctatgacgag 8805
pmxE_B-LR_Polymyxin-E        CGTATTATTAACAGCTACGGCGTCACGGAAGCGGCGATTGACTCCAGCTTCTATGACGAG 8820
pmxE_KP26_Polymyxin-E        CGTATTATTAACAGCTACGGCGTCACGGAAGCGGCGATTGACTCCAGCTTCTATGACGAG 8760
pmxE_Polymyxin-P             CGTATTATTAACAGCTACGGCGTAACGGAAGCGGCGATTGACTCCAGCTTCTATGACGAG 8820
pmxE_Polymyxin-A             CGTATTATTAACAGCTACGGCGTAACGGAAGCGGCGATTGACTCCAGCTTCTATGACGAG 8820
                             *********************** ************************************

pmxE-D-DaB3-Polymyxin-B      CCGCTGGAGAAGCTGCCGAAGACGGGCAGCGTGCCGATCGGGAAAGCGTGGCTGAACGCC 8856
pmxE_1G_Polymyxin            ccgctggagaagctgccgaagacgggcagcgtgccgatcgggaaagcgtggctgaacgcc 8865
pmxE_B-LR_Polymyxin-E        CCGCTGGAGAAGCTGCCGAAGACGGGCAGCGTGCCGATCGGGAAAGCGTGGCTGAACGCA 8880
pmxE_KP26_Polymyxin-E        CCGCTGGAGAAGCTGCCGAAGACGGGCAGCGTGCCGATCGGGAAAGCGTGGCTGAACGCA 8820
pmxE_Polymyxin-P             CCGCTGAAGAAGCTGCCGAAGACGGGTAGCGTGCCGATCGGCAAAGCTTGGCTGAACGCC 8880
pmxE_Polymyxin-A             CCGCTGGAGAAGCTGCCGAAGACGGGCAGCGTGCCGATCGGGAAAGCGTGGCTGAACGCC 8880
                             ****** ******************* ************** ***** *********** 
```

```
pmxE-D-DaB3-Polymyxin-B      AAGTTCTACATCGTGGATGCGAATCTGAAGCCGGTGCCGATCGGGGTGTTGGGCGAGCTG 8916
pmxE_1G_Polymyxin            aagttctacatcgtggatgcgaatctgaagccggtgccgatcggggtgttgggcgagctg 8925
pmxE_B-LR_Polymyxin-E        AAGTTCTACATCGTGGATGCGAGTCTGAAGCCGGTGCCGATCGGGGTGTTGGGCGAGCTG 8940
pmxE_KP26_Polymyxin-E        AAGTTCTACATCGTGGATGCGAGTCTGAAGCCGGTGCCGATCGGGGTGTTGGGCGAGCTG 8880
pmxE_Polymyxin-P             AAGTTCTACATCGTGGATGCGAATCTGAAGCCGGTGCCGATCGGGGTGTTGGGCGAGCTG 8940
pmxE_Polymyxin-A             AAGTTCTACATCGTGGATGCGAATCTGAAGCCGGTGCCGATCGGGGTGTTGGGCGAGCTG 8940
                             ********************** *************************************

pmxE-D-DaB3-Polymyxin-B      GTTATCGGCGGAGCGGGTGTGGCCCGCGGTTACTTGAACCGCCCGGATTTGACGGCGGAG 8976
pmxE_1G_Polymyxin            gttatcggcggagcgggtgtggcccgcggttacttgaaccgcccggatttgacggcggag 8985
pmxE_B-LR_Polymyxin-E        GTTATCGGCGGAGCGGGTGTGGCCCGCGGTTACTTGAACCGCCCGGATTTGACGGCGGAG 9000
pmxE_KP26_Polymyxin-E        GTTATCGGCGGAGCGGGTGTGGCCCGCGGTTACTTGAACCGCCCGGATTTGACGGCGGAG 8940
pmxE_Polymyxin-P             GTTATCGGCGGAGCGGGTGTGGGCCGCGGTTACTTGAATCGCCCGGATTTGACGGCGGAG 9000
pmxE_Polymyxin-A             GTTATCGGCGGAGCGGGTGTGGCCCGCGGTTACTTGAACCGCCCGGATTTGACGGCGGAG 9000
                             ********************** *************** *********************

pmxE-D-DaB3-Polymyxin-B      AAATTCGTAGACAGCCCGTTCGCCGCTGGGGAGCGGCTGTACCGGACGGGCGACCTGGCG 9036
pmxE_1G_Polymyxin            aaattcgtagacagcccgttcgccgctggggagcgtctgtaccggacgggcgacctggcg 9045
pmxE_B-LR_Polymyxin-E        AAATTCGTAGACAGCCCGTTCACTGCAGGGGAGCGGCTGTACCGGACGGGCGACCTGGCG 9060
pmxE_KP26_Polymyxin-E        AAATTCGTAGACAGCCCGTTCACTGCAGGGGAGCGGCTGTACCGGACGGGCGACCTGGCG 9000
pmxE_Polymyxin-P             AAATTCGTAGACAGCCCGTTCGCCGCTGGGGAGCGGCTGTACCGGACGGGCGACCTGGCG 9060
pmxE_Polymyxin-A             AAATTCGTAGACAGCCCGTTCGCCGCTGGGGAGCGGCTGTACCGGACGGGCGACCTGGCT 9060
                             ********************* * ** ******** ***********************

pmxE-D-DaB3-Polymyxin-B      CGCTGGATGCCGGACGGCAACGTGGACTTCATCGGCCGGATCGACAACCAGGTGAAAATT 9096
pmxE_1G_Polymyxin            cgctggatgccggacggcaacgtggacttcatcggccggatcgacaaccaggtgaaaatt 9105
pmxE_B-LR_Polymyxin-E        CGCTGGATGCCGGACGGCAACGTTGACTTCATCGGCCGGATCGACAACCAGGTAAAAATT 9120
pmxE_KP26_Polymyxin-E        CGCTGGATGCCGGACGGCAACGTTGACTTCATCGGCCGGATCGACAACCAGGTAAAAATT 9060
pmxE_Polymyxin-P             CGCTGGATGCCGGACGGCAACGTGGACTTCATCGGCCGGATCGACAACCAGGTGAAAATT 9120
pmxE_Polymyxin-A             CGCTGGATGCCGGACGGCAACGTGGACTTCATCGGCCGGATCGACAACCAGGTGAAAATT 9120
                             *********************** ***************************** ******

pmxE-D-DaB3-Polymyxin-B      CGCGGCTATCGGATCGAGCTTGGTGAAATTGAAGCGGCCATGAAAAATTTTGCCGGTGTG 9156
pmxE_1G_Polymyxin            cgcggctatcggatcgagcttggtgaaattgaagcggctatgaaaaattttgccggcgtg 9165
pmxE_B-LR_Polymyxin-E        CGCGGCTATCGGATCGAGCTTGGTGAAATTGAAGCGGCTATGAAAAATTTTGCCGGCGTT 9180
pmxE_KP26_Polymyxin-E        CGCGGCTATCGGATCGAGCTTGGTGAAATTGAAGCGGCTATGAAAAATTTTGCCGGCGTT 9120
pmxE_Polymyxin-P             CGCGGCTATCGGATCGAGCTTGGTGAAATTGAAGCGGCCATGAAAAATTTTGCCGGCGTG 9180
pmxE_Polymyxin-A             CGCGGCTATCGGATCGAGCTTGGTGAAATTGAAGCGGCCATGAAAAATTTTGCCGGCGTG 9180
                             ************************************** ***************** **

pmxE-D-DaB3-Polymyxin-B      CGTCAAGCGCTTGTCATCGACCGGATGGACGAGCGGGGGCAGAAATATTTGTGCGGGTAT 9216
pmxE_1G_Polymyxin            cgtcaagcgcttgtcatcgaccggacggacgagcgggggcagaaatatttgtgtgggtat 9225
pmxE_B-LR_Polymyxin-E        CGTCAAGCGCTTGTCATCGACCGGACGGACGAGCGGGGGCAGAAATATTTGTGCGGGTAT 9240
```

```
pmxE_KP26_Polymyxin-E        CGTCAAGCGCTTGTCATCGACCGGACGGACGAGCGGGGGCAGAAATATTTGTGCGGGTAT 9180
pmxE_Polymyxin-P             CGTCAAGCGCTTGTCATCGACCGGACGGACGAGCGGGGGCAGAAATATTTGTGCGGGTAT 9240
pmxE_Polymyxin-A             CGTCAAGCGCTTGTCATCGACCGGACGGACGAGCGGGGGCAGAAATATTTGTGCGGGTAT 9240
                             ************************* *************************** ******

pmxE-D-DaB3-Polymyxin-B      GTCGTAGCGGATTCCAGCTTCGATCTGGAAGGGCTTGTGGCCCATCTGGACGCCGCGCTG 9276
pmxE_1G_Polymyxin            gtcgtagcggattccagcttcgatctggaagggcttgtggcccatctggacgccgcgctg 9285
pmxE_B-LR_Polymyxin-E        GTCGTAGCGGATTCCAGCTTCGATCTGGAAGGGCTTGTGGCCCATCTGGACGCTGCACTG 9300
pmxE_KP26_Polymyxin-E        GTCGTAGCGGATTCCAGCTTCGATCTGGAAGGGCTTGTGGCCCATCTGGACGCTGCACTG 9240
pmxE_Polymyxin-P             GTCGTAGCGGATTCCAGCTTCGATCTGGAAGGGCTTGTGGCCCATCTGGACGCCGCGCTG 9300
pmxE_Polymyxin-A             GTCGTAGCGGATTCCAGCTTCGATCTGGAAGGGCTTGTGGCCCATCTCGACGCCGCGCTG 9300
                             *********************************************** ***** ** ***

pmxE-D-DaB3-Polymyxin-B      CCTTCCCATATGGTGCCTTCGCGCATCATGCGCCTGGATCAAATGCCGCTTACGCCGAAC 9336
pmxE_1G_Polymyxin            ccttcccatatggtgccttcgcgcatcatgcgcctggatcaaatgccgcttacgccgaac 9345
pmxE_B-LR_Polymyxin-E        CCTTCCCATATGGTGCCTTCGCGTATCATGCGCCTGGATCAAATGCCGCTTACGCCGAAC 9360
pmxE_KP26_Polymyxin-E        CCTTCCCATATGGTGCCTTCGCGTATCATGCGCCTGGATCAAATGCCGCTTACGCCGAAC 9300
pmxE_Polymyxin-P             CCTTCCCATATGGTGCCTTCGCGCATCATGCGCCTGGATCAATTGCCGCTTACGCCGAAC 9360
pmxE_Polymyxin-A             CCTTCCCATATGGTGCCTTCGCGCATCATGCGCCTGGATCAAATGCCGCTTACGCCGAAC 9360
                             *********************** ****************** *****************

pmxE-D-DaB3-Polymyxin-B      GGGAAGATCGACCGTAAAGCGCTGCCTGTACCGGAAGGAAGCATTCGTGCCGAGGCTGCA 9396
pmxE_1G_Polymyxin            gggaagatcgaccgtaaagcgctgcctgtgccggaaggaagcattcgtgccgaggctgca 9405
pmxE_B-LR_Polymyxin-E        GGGAAGATCGACCGTAAAGGGCTGCCTGTGCCGGAAGGAAGCATTCGTGCCGAGGCTGCA 9420
pmxE_KP26_Polymyxin-E        GGGAAGATCGACCGTAAAGGGCTGCCTGTGCCGGAAGGAAGCATTCGTGCCGAGGCTGCA 9360
pmxE_Polymyxin-P             GGGAAGATCGACCGTAAAGCGCTGCCTGTACCGGAAGGAAGCATTCGTGCCGAGGCTGCA 9420
pmxE_Polymyxin-A             GGGAAGATCGACCGTAAAGCGCTGCCTGTACCGGAAGGAAGCATTCGTGCCGAGGCTGCA 9420
                             ******************* ********* ******************************

pmxE-D-DaB3-Polymyxin-B      TACACGGCGCCTCGTACTCCTGCCGAGCAAGCACTTGCCTCGGTCTGGCAGTCGGTGCTG 9456
pmxE_1G_Polymyxin            tacacggcgcctcgtactcctgtcgagcaagcacttgcatcggtctggcagtcggtgctg 9465
pmxE_B-LR_Polymyxin-E        TACACGGCGCCTCGTACTCCTGCTGAGCAAGCACTTGCGTTGGTCTGGCAGTCAGTGCTG 9480
pmxE_KP26_Polymyxin-E        TACACGGCGCCTCGTACTCCTGCTGAGCAAGCACTTGCGTTGGTCTGGCAGTCAGTGCTG 9420
pmxE_Polymyxin-P             TACACGGCACCTCGTACTCCTGCCGAGCAAGCACTTGCGTCGGTCTGGCAGTCGGTGCTG 9480
pmxE_Polymyxin-A             TACACGGCGCCTCGTACTCCTGCCGAGCAAGCACTTGCGTCGGTCTGGCAGTCGGTGCTG 9480
                             ******** *************  ************** * ************ ******

pmxE-D-DaB3-Polymyxin-B      GGCGTGGATCAGGTCGGCACGATGGACAATTTCTTTGCGCTCGGCGGCGATTCGATCAAG 9516
pmxE_1G_Polymyxin            ggcgtggatcaggtcggcacgatggacaatttctttgcgctcggcggcgattcgatcaag 9525
pmxE_B-LR_Polymyxin-E        GGCGTGGATCAGGTCGGCACGATGGACAATTTCTTTGCGCTCGGCGGCGATTCGATCAAG 9540
pmxE_KP26_Polymyxin-E        GGCGTGGATCAGGTCGGCACGATGGACAATTTCTTTGCGCTCGGCGGCGATTCGATCAAG 9480
pmxE_Polymyxin-P             GGCGTGGATCAGGTCGGCACGATGGACAATTTCTTTGCGCTCGGCGGCGATTCGATCAAG 9540
pmxE_Polymyxin-A             GGCGTGGACCAGGTCGGCACGATGGACAATTTCTTTGCGCTCGGCGGCGATTCGATCAAG 9540
                             ******** ***************************************************
```

```
pmxE-D-DaB3-Polymyxin-B      GCCTTGCAGGTATCGTCCCGTCTTTTGCAAACGGGGTACAAGCTGGTCATGAAAGATTTG 9576
pmxE_1G_Polymyxin            gccttgcaggtatcgtcccgtcttttgcaaacggggtacaagctggtcatgaaagatttg 9585
pmxE_B-LR_Polymyxin-E        GCCTTGCAGGTATCGTCCCGTCTTTTGCAAACGGGGTACAAGCTGATCATGAAAGATTTG 9600
pmxE_KP26_Polymyxin-E        GCCTTGCAGGTATCGTCCCGTCTTTTGCAAACGGGGTACAAGCTGATCATGAAAGATTTG 9540
pmxE_Polymyxin-P             GCCTTGCAGGTATCGTCCCGTCTTTTGCAAACGGGGTACAAGCTGGTCATGAAAGATTTG 9600
pmxE_Polymyxin-A             GCCTTGCAGGTATCGTCCCGTCTTTTGCAAACGGGGTACAAGCTGGTCATGAAAGATTTG 9600
                             ******************************************** ***************

pmxE-D-DaB3-Polymyxin-B      TTCCATTACCCGACGATTTCCGCCCTTAGTTTGCAGCTGCAAACGGCGGAGAGAACGGCA 9636
pmxE_1G_Polymyxin            ttccattacccgacgatttccgcccttagtttgcagctgcaaacggcggagagaacggca 9645
pmxE_B-LR_Polymyxin-E        TTCCATTACCCGACGATTTCCGCCCTTAGTTTGCAGCTGCAAACGGCGGAGAGAACGGCA 9660
pmxE_KP26_Polymyxin-E        TTCCATTACCCGACGATTTCCGCCCTTAGTTTGCAGCTGCAAACGGCGGAGAGAACGGCA 9600
pmxE_Polymyxin-P             TTCCATTACCCGACGATTTCCGCCCTTAGTTTGCAGCTGCAAACGGCGGAGAGAACGGCA 9660
pmxE_Polymyxin-A             TTCCATTACCCGACGATTTCCGCCCTTAGTTTGCAGCTGCAAACGGCGGAGAGAACGGCA 9660
                             ************************************************************

pmxE-D-DaB3-Polymyxin-B      AGCCAGGCCGAAGTGACGGGAGAGGTCATCTTGACCCCGATTCAGCGCTGGTTCTTTGAA 9696
pmxE_1G_Polymyxin            agccaggccgaagtgacgggagaggtcatcttgaccccgattcagcgctggttctttgaa 9705
pmxE_B-LR_Polymyxin-E        AGCCAGGCCGAAGTGACGGGGGAGGTCATCTTGACCCCGATTCAGCGCTGGTTCTTTGAA 9720
pmxE_KP26_Polymyxin-E        AGCCAGGCCGAAGTGACGGGGGAGGTCATCTTGACCCCGATTCAGCGCTGGTTCTTTGAA 9660
pmxE_Polymyxin-P             AGCCAGGCCGAAGTGACGGGAGAGGTCATCTTGACCCCGATTCAGCGCTGGTTCTTTGAA 9720
pmxE_Polymyxin-A             AGCCAGGCCGAAGTGACGGGAGAGGTCATCTTGACCCCGATTCAGCGCTGGTTCTTTGAA 9720
                             ******************** ***************************************

pmxE-D-DaB3-Polymyxin-B      CAAAATCCGGCCGACGTGCATCACAGCAACCAGGCGTTCATGCAGTTCTCCAAGCAAGGC 9756
pmxE_1G_Polymyxin            caaaatccggccgacgtgcatcacagcaaccaggcgttcatgcagttctccaagcaaggc 9765
pmxE_B-LR_Polymyxin-E        CAAAATCCGGCCGACGTGCATCACAGCAACCAGGCATTCATGCAGTTCTCCAAGCTAGGC 9780
pmxE_KP26_Polymyxin-E        CAAAATCCGGCCGACGTGCATCACAGCAACCAGGCATTCATGCAGTTCTCCAAGCTAGGC 9720
pmxE_Polymyxin-P             CAAAATCCGGCCGACGTGCATCACAGCAACCAGGCGTTCATGCAGTTCTCCAAGCAAGGC 9780
pmxE_Polymyxin-A             CAAAATCCGGCCGACGTGCATCACAGCAACCAGGCGTTCATGCAGTTCTCCAAGGAAGGC 9780
                             *********************************** ******************  ****

pmxE-D-DaB3-Polymyxin-B      TTTGACGAAGAAGCTTTACGCCAAGCGGTGCGTCAGCTTGTCGTGCATCACGATGCTCTC 9816
pmxE_1G_Polymyxin            tttgacgaagaagctttacgccaagcggtgcgtcagcttgtcgtgcatcacgatgctctc 9825
pmxE_B-LR_Polymyxin-E        TTCGACGAAGAAGCTTTACGCCAAGCGGTGCGTCAACTTGTCGTGCATCACGATGCTCTC 9840
pmxE_KP26_Polymyxin-E        TTCGACGAAGAAGCTTTACGCCAAGCGGTGCGTCAACTTGTCGTGCATCACGATGCTCTC 9780
pmxE_Polymyxin-P             TTTGACGAAGAAGCTTTACGTCAAGCGGTGCGTCAGCTTGTCGTGCATCACGATGCTCTC 9840
pmxE_Polymyxin-A             TTTGACGAAGAAGCTTTACGCCAAGCGGTGCGTCAGATTGTCGTGCATCACGATGCTCTC 9840
                             ** ***************** **************  ***********************

pmxE-D-DaB3-Polymyxin-B      CGTACGGTTTACCGCCAAACTGAGAACGGCTATACCGCCTGGAACCGCGGCGCCGGGGAG 9876
pmxE_1G_Polymyxin            cgtacggtttaccgccaaaccgagaacggctataccgcctggaactgcggcgccggggag 9885
pmxE_B-LR_Polymyxin-E        CGTACGGTTTACCGCCAAACCGAAAACAGCTATACCGCCTGGAACCGCGGCGCCGGGGAG 9900
```

```
pmxE_KP26_Polymyxin-E        CGTACGGTTTACCGCCAAACCGAAAACAGCTATACCGCCTGGAACCGCGGCGCCGGGGAG 9840
pmxE_Polymyxin-P             CGTACGGTTTACCGCCAAACCGAGAATGGCTATATCGCCTGGAACCGCGGCGCCGGGGAG 9900
pmxE_Polymyxin-A             CGTACGGTTTACCGCCAAGCCGACAACGGCTATACCGCCTGGAACCGCGGCGCCGGGGAG 9900
                             ****************** * ** **  ****** ********** **************

pmxE-D-DaB3-Polymyxin-B      AACGAAGCGCTGTTCGATCTGGAAGTTGTCGATTTCAAGGGAGTCGGCGACGTGAAAGAA 9936
pmxE_1G_Polymyxin            aacgaagcgctgttcgatctggaagttgtcgatttcaagggagtcggcgacgtgaaagaa 9945
pmxE_B-LR_Polymyxin-E        AACGAAGCACTGTTCGATCTGGAAGTTGTAGATTTCAGGGGAGTCTGCGACGTGAAAGGC 9960
pmxE_KP26_Polymyxin-E        AACGAAGCACTGTTCGATCTGGAAGTTGTAGATTTCAGGGGAGTCTGCGACGTGAAAGGC 9900
pmxE_Polymyxin-P             AACGAAGCGCTGTTCGATCTGGAATTTGTCGATTTCAAGGGAGTCGGCGACGTGAAAGAA 9960
pmxE_Polymyxin-A             AACGAAGCGCTGTTCGATCTGGAAGTTGTCGATTTCAAGGGAGTCGGCGACGTGAAAGAA 9960
                             ******** *************** **** ******* ******* ************

pmxE-D-DaB3-Polymyxin-B      GCGGTAGAGGCTAAGGCGAATGACATTCAAGCGAGCATCGATCTGGAAAACGGTCCGCTG 9996
pmxE_1G_Polymyxin            gcggtagaggctaaggcgaatgacattcaagcgagcatcgatctggaaaacggtccgctg 10005
pmxE_B-LR_Polymyxin-E        GCGGTAGAGGCTAAGGCGAATGATATTCAAGCGAGCATCGATCTGGAAAACGGCCCGCTG 10020
pmxE_KP26_Polymyxin-E        GCGGTAGAGGCTAAGGCGAATGATATTCAAGCGAGCATCGATCTGGAAAACGGCCCGCTG 9960
pmxE_Polymyxin-P             GCGGTAGAGGCTAAGGCAAATGACATTCAAGCGAGCATCGATCTGGAAAACGGTCCGCTG 10020
pmxE_Polymyxin-A             GCGGTAGAGGCTAAGGCGAATGACATTCAAGCGAGCATCGATCTGGAAAACGGTCCGCTG 10020
                             ***************** ***** ***************************** ******

pmxE-D-DaB3-Polymyxin-B      GTGAAGCTCGGCTTGTTCCGCTGCGACGACGGCGACCACCTGCTCATCGCGATCCATCAC 10056
pmxE_1G_Polymyxin            gtgaagctcggcttgttccgctgcgacgacggcgaccacctgctcatcgcgatccatcac 10065
pmxE_B-LR_Polymyxin-E        GTGAAGCTCGGCTTGTTCCGCTGCGACGACGGCGACCACCTGCTCATCGCGATCCATCAC 10080
pmxE_KP26_Polymyxin-E        GTGAAGCTCGGCTTGTTCCGCTGCGACGACGGCGACCACCTGCTCATCGCGATCCATCAC 10020
pmxE_Polymyxin-P             GTGAAGCTCGGCTTGTTCCGCTGCGACGACGGCGACCACCTGCTCATCGCGATCCATCAC 10080
pmxE_Polymyxin-A             GTGAAGCTCGGCTTGTTCCGCTGCGACGACGGCGACCACCTACTCATCGCGATCCATCAC 10080
                             ***************************************** ******************

pmxE-D-DaB3-Polymyxin-B      TTGGTCGTAGACGGCGTATCCTGGCGGATTCTGCTTGAGGATTTTGCTGCCGGCTATGAG 10116
pmxE_1G_Polymyxin            ttggtcgtagacggcgtatcctggcggattctgcttgaggattttgctgccggctatgag 10125
pmxE_B-LR_Polymyxin-E        TTGGTCGTAGACGGCGTATCATGGCGGATTCTGCTTGAAGATTTTGCTGCCGGATATGAG 10140
pmxE_KP26_Polymyxin-E        TTGGTCGTAGACGGCGTATCATGGCGGATTCTGCTTGAAGATTTTGCTGCCGGATATGAG 10080
pmxE_Polymyxin-P             TTGGTCGTAGACGGCGTATCCTGGCGGATTCTGCTTGAGGATTTTGCTGCCGGCTATGAG 10140
pmxE_Polymyxin-A             TTGGTCGTAGACGGCGTATCCTGGCGGATTCTGCTTGAGGATTTTGCTGCCGGCTATGAG 10140
                             ******************** ***************** ************** ******

pmxE-D-DaB3-Polymyxin-B      CAGGCGCTGCAAGGGCAGCCGATCCGTCTGCCGCTCAAAACGGATTCATTCCAAACGTGG 10176
pmxE_1G_Polymyxin            caggcgctgcaagggcagccgatccgtctgccgctcaaaacggattcattccaaacgtgg 10185
pmxE_B-LR_Polymyxin-E        CAGGTGCTGCAAGGGCAGCCGATCCGTCTGCCGCTCAAAACGGATTCATTCCAAACGTGG 10200
pmxE_KP26_Polymyxin-E        CAGGTGCTGCAAGGGCAGCCGATCCGTCTGCCGCTCAAAACGGATTCATTCCAAACGTGG 10140
pmxE_Polymyxin-P             CAGGCGCTGCAAGGGCAGCCGATCCGTCTGCCGCTCAAAACGGATTCATTCCAAACGTGG 10200
pmxE_Polymyxin-A             CAGGCGCTGCAAGGGCAGCCGATCCGTCTGCCGCTCAAAACGGATTCATTCCAAACGTGG 10200
                             **** *******************************************************
```

```
pmxE-D-DaB3-Polymyxin-B     GCGAAACAGCTCGCTGATTATGCGAACGGTCCGGCGATGGAAAGCGAGAGAGAGTATTGG 10236
pmxE_1G_Polymyxin           gcgaaacagctcgctgattatgcgaacggtccggcgatggaaagcgagagagagtattgg 10245
pmxE_B-LR_Polymyxin-E       GCGAAACAGCTCGCTGATTATGCGAACGATCCGGCGATGGAAAGCGAAAGAGAGTATTGG 10260
pmxE_KP26_Polymyxin-E       GCGAAACAGCTCGCTGATTATGCGAACGATCCGGCGATGGAAAGCGAAAGAGAGTATTGG 10200
pmxE_Polymyxin-P            GCGAAACAGCTCGCTGATTATGCGAACGGTCCGGCGATGGAAAGCGAGAGAGAGTACTGG 10260
pmxE_Polymyxin-A            GCGAAACAGCTCGCTGATTATGCGAACAGCCCGGCGATGGAAAGCGAGAGAGAGTATTGG 10260
                            ***************************   ***************** ******** ***

pmxE-D-DaB3-Polymyxin-B     CAGCATATCGAGCAATTGATCTATGAGCCGCTTCCGAAAGATTTTGAACAAGGCAGATCC 10296
pmxE_1G_Polymyxin           cagcatatcgagcaattgacctatgagccgcttccgaaagattttgaacaaggcagatcc 10305
pmxE_B-LR_Polymyxin-E       CAGCATATCGAGCAATTGAGCTATGAGCCGCTTCCAAAAGATTTTGAACAAGGCAGATCC 10320
pmxE_KP26_Polymyxin-E       CAGCATATCGAGCAATTGAGCTATGAGCCGCTTCCAAAAGATTTTGAACAAGGCAGATCC 10260
pmxE_Polymyxin-P            CAGCATATCGAGCAATTGACCTATGAGCCGCTTCCGAAAGATTTTGAACAAGGCAGATCC 10320
pmxE_Polymyxin-A            CAGCATATCGAGCAATTGACCTATGAGCCGCTTCCGAAAGATTTTGAACAAGGCAGATCC 10320
                            ******************* *************** ************************

pmxE-D-DaB3-Polymyxin-B     AAGCTGAAGGACAGCGGGCTCGTGACCGTTCGCTGGACGGCGGAGGAAACCGAACAGCTG 10356
pmxE_1G_Polymyxin           aagctgaaggacagcgggctcgtgaccgttcgctggacggcggaggaaaccgaacagctg 10365
pmxE_B-LR_Polymyxin-E       ACGCTGAAGGACAGCGGTCTCGTGACCGTTCGCTGGACAGCGGAGGAAACCGAACAGCTG 10380
pmxE_KP26_Polymyxin-E       ACGCTGAAGGACAGCGGTCTCGTGACCGTTCGCTGGACAGCGGAGGAAACCGAACAGCTG 10320
pmxE_Polymyxin-P            AAGCTGAAGGACAGCGGGCTCGTGACCGTTCGCTTGACGGCGGAGGAAACCGAACAGCTG 10380
pmxE_Polymyxin-A            AAGCTGAAGGACAGCGGGCTCGTGACCGTTCGCTGGACGGCGGAGGAAACCGAACAGCTG 10380
                            * *************** **************** *** *********************

pmxE-D-DaB3-Polymyxin-B     CTGAAGCAGGCACACCGTGCTTACCATACGGAAATGAACGATTTGCTGCTTGCCGCGCTT 10416
pmxE_1G_Polymyxin           ctgaagcaggcacaccgtgcttaccatacggaaatgaacgatttgctgcttgccgcgctt 10425
pmxE_B-LR_Polymyxin-E       CTGAAGCACGCACACCGTGCTTACCGTACGGAAATGAACGATTTGCTGCTTGCCGCGCTT 10440
pmxE_KP26_Polymyxin-E       CTGAAGCACGCACACCGTGCTTACCGTACGGAAATGAACGATTTGCTGCTTGCCGCGCTT 10380
pmxE_Polymyxin-P            CTGAAGCAGGCACACCGTGCTTACCATACGGAAATGAACGATTTGCTGCTTGCCGCGCTT 10440
pmxE_Polymyxin-A            CTGAAGCAGGCACACCGTGCTTACCATACGGAAATGAACGATTTGCTGCTTGCCGCGCTT 10440
                            ******** **************** **********************************

pmxE-D-DaB3-Polymyxin-B     GGCCTCGCGGTACAAGCTTGGAGCGGCCGGGAACGCGTGCTGGTGAATCTCGAAGGCCAC 10476
pmxE_1G_Polymyxin           ggcctcgcggtacaagcttggagcggccgggaacgcgtgctggtgaatctcgaaggccac 10485
pmxE_B-LR_Polymyxin-E       GGCCTCGCGGTACAAGCTTGGAGCGGCCGGGGACGCGTGCTGGTGAATCTCGAAGGCCAC 10500
pmxE_KP26_Polymyxin-E       GGCCTCGCGGTACAAGCTTGGAGCGGCCGGGGACGCGTGCTGGTGAATCTCGAAGGCCAC 10440
pmxE_Polymyxin-P            GGCCTCGCGATACAAGCTTGGAGCGGCCGGGAACGCGTGCTGGTGAATCTCGAAGGCCAC 10500
pmxE_Polymyxin-A            GGCCTCGCTTTACAAGCTTGGAGCGGCCGGGAACGCGTGCTGGTGAATCTCGAAGGCCAC 10500
                            ********  ********************* ****************************

pmxE-D-DaB3-Polymyxin-B     GGCCGGGAAGATATTTTGCCGAATGTGGACATTACGCGCACGGTAGGCTGGTTTACAAGC 10536
pmxE_1G_Polymyxin           ggccgggaagatattttgccggatgtggacattacgcgcacggtaggctggtttacaagc 10545
pmxE_B-LR_Polymyxin-E       GGCCGGGAAGATATTTTGCCGGATGTGGACATTACACGCACGGTAGGCTGGTTTACAAGC 10560
```

```
pmxE_KP26_Polymyxin-E        GGCCGGGAAGATATTTTGCCGGATGTGGACATTACACGCACGGTAGGCTGGTTTACAAGC 10500

pmxE_Polymyxin-P             GGCCGGGAAGATATTTTGCCGGATGTGGACATTACGCGCACGGTAGGCTGGTTTACAAGC 10560

pmxE_Polymyxin-A             GGCCGGGAAGATATTTTGCCGGATGTGGACATTACGCGCACGGTAGGCTGGTTTACAAGC 10560

                             *********************** ************** ***********************


pmxE-D-DaB3-Polymyxin-B      CAATTCCCTGTCGTTCTGGAGCCGGGTCACGCCCAGGCGCTCGGTCATCAGGTAAAACAG 10596

pmxE_1G_Polymyxin            caattccctgtcgttctggagccgggtcacgcccaggcgctcggtcatcaggtgaaacag 10605

pmxE_B-LR_Polymyxin-E        CAATTCCCTGTCGTTCTGGAGCCGGGTCACGCCCAGGAGCTCGGTCATCAGCTGAAACAG 10620

pmxE_KP26_Polymyxin-E        CAATTCCCTGTCGTTCTGGAGCCGGGTCACGCCCAGGAGCTCGGTCATCAGCTGAAACAG 10560

pmxE_Polymyxin-P             CAATTCCCTGTCGTTCTGGAGCCGGGTCACGCCCAGGCGCTCGGTCATCAGGTGAAACAG 10620

pmxE_Polymyxin-A             CAATTCCCTGTCGTTCTGGAGCCGGGTCACGCCCAGGCGCTCGGTCATCAGGTGAAACAG 10620

                             ************************************* ************* * ******


pmxE-D-DaB3-Polymyxin-B      GTTAAAGAAAGCTTGCGCCGCATTCCGAACAAAGGAATCGGCTACGGCATCCTGCGTTAT 10656

pmxE_1G_Polymyxin            gttaaagaaagcttgcgccgcattccgaacaaaggaatcggctacggcatcctgcgttat 10665

pmxE_B-LR_Polymyxin-E        GTTAAAGAAAGCTTGCGCCGCATTCCGAACAAAGGAATCAGCTATGGCATCCTGCGCTAT 10680

pmxE_KP26_Polymyxin-E        GTTAAAGAAAGCTTGCGCCGCATTCCGAACAAAGGAATCAGCTATGGCATCCTGCGCTAT 10620

pmxE_Polymyxin-P             GTTAAAGAAAGCTTGCGCCGCATTCCGAACAAAGGAATCGGCTACGGCATCCTGCGTTAT 10680

pmxE_Polymyxin-A             GTTAAAGAAAGCTTGCGCCGCATTCCGAACAAAGGAATCGGCTACGGCATCCTGCGTTAT 10680

                             *************************************** **** *********** ***


pmxE-D-DaB3-Polymyxin-B      TTGTCGGCGCCGCGTGACGGCGAGCGCTTCGCTTTGGAGCCGGAGATCAGCTTTAACTAT 10716

pmxE_1G_Polymyxin            ttgtcggcgccgcgtgacggcgagcgcttcgctttggagccggagatcagctttaactat 10725

pmxE_B-LR_Polymyxin-E        TTGTCGGCGCCGCGTGACGGCGAGTGCTTCGCTTTGGAGCCGGAGATCAGCTTTAACTAT 10740

pmxE_KP26_Polymyxin-E        TTGTCGGCGCCGCGTGACGGCGAGTGCTTCGCTTTGGAGCCGGAGATCAGCTTTAACTAT 10680

pmxE_Polymyxin-P             TTGTCGGCGCCGCGTGACGGCGAGCGCTTCGCTTTGGAGCCGGAGATCAGCTTTAACTAT 10740

pmxE_Polymyxin-A             TTGTCGGCGCCGCGTGAAGGCGAGTACTTCGTTTTAGAGCCGGAGATCAGCTTTAACTAC 10740

                             ***************** ******  ***** *** ***********************


pmxE-D-DaB3-Polymyxin-B      TTGGGTCAGTTCGACCAGGATTACGAAAGCAGCGGCTCGAGGCCGTCTCCGTTCAGCCCG 10776

pmxE_1G_Polymyxin            ttgggtcagttcgaccaggattacgaaagcagcggctcaaggccgtctccgttcagcccg 10785

pmxE_B-LR_Polymyxin-E        TTGGGTCAGTTCGACCAGGATTACGAAAGCAGCGGCTCGCAGCCGTCTCCGTTCAGCCCG 10800

pmxE_KP26_Polymyxin-E        TTGGGTCAGTTCGACCAGGATTACGAAAGCAGCGGCTCGCAGCCGTCTCCGTTCAGCCCG 10740

pmxE_Polymyxin-P             TTGGGTCAGTTCGACCAGGATTACGAAAGCAGCGGCTCGCGGCCGTCTCCGTTCAGCCCG 10800

pmxE_Polymyxin-A             CTGGGTCAGTTCGACCATGATTACGAAAGCAGCAGCTCGCAGCCGTCCCCGTTCAGCCCG 10800

                              **************** *************** ****   ****** ************


pmxE-D-DaB3-Polymyxin-B      GGCTCCGATTCAAGCCCGGATGCAGTGATGGATTATGTCCTCGATATCAACGGTATGGTG 10836

pmxE_1G_Polymyxin            ggctccgattcaagtccgaatgcagtgatggattatgtcctcgatatcaacggtatggtg 10845

pmxE_B-LR_Polymyxin-E        GGCTCCGACTCAAGCCCGAACGCAGTGATGGATTTTGTCCTAGATATCAACGGTATGGTG 10860

pmxE_KP26_Polymyxin-E        GGCTCCGACTCAAGCCCGAACGCAGTGATGGATTTTGTCCTAGATATCAACGGTATGGTG 10800

pmxE_Polymyxin-P             GGCTCCGATTCAAGTCCGAATGCAGTGATGGATTATGTCCTCGATATCAACGGTATGGTG 10860

pmxE_Polymyxin-A             GGCTCCGACTCAAGCCCGAATGCTGTGATGGATTATGTCCTTGATATCAACGGTATGGTG 10860

                             ******** ***** *** * ** ********** ****** ******************
```

```
pmxE-D-DaB3-Polymyxin-B      TCGGAAGGAGTGCAGGAACTCACGATCCGTTATGGGGAAACCCAGTATAAACGGGAAACG 10896
pmxE_1G_Polymyxin            tcggaaggagtgcaggaactcacgatccgttatggggaaacccagtataaacgggaaacg 10905
pmxE_B-LR_Polymyxin-E        TCGGAAGGAGTGCTGGAACTCACGATCCGTTATGGGGAAACCCAGTATAAACGGGAAACG 10920
pmxE_KP26_Polymyxin-E        TCGGAAGGAGTGCTGGAACTCACGATCCGTTATGGGGAAACCCAGTATAAACGGGAAACG 10860
pmxE_Polymyxin-P             TCGGAAGGAGTGCAGGAACTCACGATCCGTTATGGGGAAACCCAGTATAAACGGGAAACG 10920
pmxE_Polymyxin-A             TCGGAAGGAGCGTTGGAACTCACGATCCGTTATGGGGAAACCCAGTATAAACGGGAAACG 10920
                             ********** *  **********************************************

pmxE-D-DaB3-Polymyxin-B      GTAGAGCGCCTGGGCACCCTGCTTCATTCGAGCTTGCGTGAAGTCATCAGCCATTGCGTA 10956
pmxE_1G_Polymyxin            gtagagcgcctgggcaccctgcttcattcgagcttgcgtgaagtcatcagccattgcgta 10965
pmxE_B-LR_Polymyxin-E        GTAGAGCGCCTGGGCACCCTGCTTCAATTGAGCTTGCGTGAAGTCATCAACCATTGCGTA 10980
pmxE_KP26_Polymyxin-E        GTAGAGCGCCTGGGCACCCTGCTTCAATTGAGCTTGCGTGAAGTCATCAACCATTGCGTA 10920
pmxE_Polymyxin-P             ATAGAGCGCCTGGGTACCCTGCTTCATTCGAGCTTGCGTGAAGTCATCAGCCATTGCGTA 10980
pmxE_Polymyxin-A             GTGGAGCGTCTGGGCACCCTGCTTCAATCGAGCTTGCGTGAAGTCATCAGCCATTGCTTA 10980
                              * ***** ***** *********** * ******************** ******* **

pmxE-D-DaB3-Polymyxin-B      TCGAAAGAGCGGCCGGAGCTTACGCCTAGCGACGTACTGCTTCAAGATGTGACGTTGGAG 11016
pmxE_1G_Polymyxin            tcgaaagagcggccggagcttacgcctagcgacgtactgcttcaagatgtgacgttggag 11025
pmxE_B-LR_Polymyxin-E        TCGAAAGAGCGGCCGGAGCTTACGCCTAGCGACGTACTGCTTCAAGATGTGACGGTGGAG 11040
pmxE_KP26_Polymyxin-E        TCGAAAGAGCGGCCGGAGCTTACGCCTAGCGACGTACTGCTTCAAGATGTGACGGTGGAG 10980
pmxE_Polymyxin-P             TCGAAAGAGCGGCCGGAGCTTACGCCTAGCGACGTACTGCTTCAAGATGTGACGGTGGAG 11040
pmxE_Polymyxin-A             TCAAAAGAGCGGCCGGAGCTTACGCCTAGCGACGTACTGCTTCAAGATGTGACGGTGGAG 11040
                             ** *************************************************** *****

pmxE-D-DaB3-Polymyxin-B      GAACTGGAGCGGCTGGCCGAACATACGGCGGCGCTCGGCGAACTGGAGAATGTATACACC 11076
pmxE_1G_Polymyxin            gaactggagcggctgaccgaacatacggcggcgctcggcgaactggagaatgtatacacc 11085
pmxE_B-LR_Polymyxin-E        GAACTGGAGCGGTTGGCTGAACATACGGCGGCGCTCGGCGAACTGGAGAATGTATACACC 11100
pmxE_KP26_Polymyxin-E        GAACTGGAGCGGTTGGCTGAACATACGGCGGCGCTCGGCGAACTGGAGAATGTATACACC 11040
pmxE_Polymyxin-P             GAACTGGAGCGGCTGGCCGAACATACGGCGGCGCTCGGCGAACTGGAGAATGTATACACC 11100
pmxE_Polymyxin-A             GAACTGGAGCGGCTGTCCGAACATACGGTGGCGCTCGGCGAACTGGAGAATGTATACACC 11100
                             ************ ** * ********** *******************************

pmxE-D-DaB3-Polymyxin-B      CTGACTCCGCTGCAAAAAGGATGTTGTTCCACAGCCTGCTGGATGCCGATTCGGAAGCT 11136
pmxE_1G_Polymyxin            ctgactccgctgcaaaaaggatgttgttccacagcctgctggatgccgattcggaagct 11145
pmxE_B-LR_Polymyxin-E        CTGACTCCGCTGCAAAAAGGATGTTGTTCCACAGCCTGCTGGATGCCGATTCGGAAGCT 11160
pmxE_KP26_Polymyxin-E        CTGACTCCGCTGCAAAAAGGATGTTGTTCCACAGCCTGCTGGATGCCGATTCGGAAGCT 11100
pmxE_Polymyxin-P             CTGACTCCGCTGCAAAAAGGATGTTGTTCCACAGCCTGCTGGATGCCGATTCGGAAGCT 11160
pmxE_Polymyxin-A             CTGACTCCACTGCAAAAAGGATGTTGTTCCACAGCTTGCTGGATGCCGATTCGGAAGCT 11160
                             ******** ************************** ************************

pmxE-D-DaB3-Polymyxin-B      TACTTCGAACAGGTGACCTTCGATCTGTACGGAAGCCTGAATGTCGAAGCCTTCACCCAA 11196
pmxE_1G_Polymyxin            tacttcgaacaggtgaccttcgatctgtacggaagcctgaatgtcgaagccttcacccaa 11205
pmxE_B-LR_Polymyxin-E        TACTTCGAACAGGTGACCTTCGATCTGAACGGAAGCCTGAATGTCGAAGCCTTCACCCAA 11220
```

```
pmxE_KP26_Polymyxin-E        TACTTCGAACAGGTGACCTTCGATCTGAACGGAAGCCTGAATGTCGAAGCCTTCACCCAA 11160
pmxE_Polymyxin-P             TACTTCGAACAGGTGACCTTCGATCTGTGCGGAAGCCTGAATGTCGAAGCCTTCACCCAA 11220
pmxE_Polymyxin-A             TACTTCGAACAGGTGACCTTCGATCTGTACGGAAGCCTGAATGTGGAAGCCTTCACCCGA 11220
                             ***************************  *************** ************* *

pmxE-D-DaB3-Polymyxin-B      GGATTGGATACGCTGGTGCAGCGGAATGAGGCGCTGCGTACCAACTTTATTACCGGCTGG 11256
pmxE_1G_Polymyxin            ggattggatacgctggtgcagcggaatgaggcgctgcggaccaactttattaccggctgg 11265
pmxE_B-LR_Polymyxin-E        GGATTGGATACGCTGGTGCAGCGGAATGAGGCACTGCGGACCAACTTTATTACCGGCTGG 11280
pmxE_KP26_Polymyxin-E        GGATTGGATACGCTGGTGCAGCGGAATGAGGCACTGCGGACCAACTTTATTACCGGCTGG 11220
pmxE_Polymyxin-P             GGATTGGATACGCTGGTGCAGCGGAATGAGGCGCTGCGGACCAACTTTATTACCGGCTGG 11280
pmxE_Polymyxin-A             GGATTGGATACGCTGGTGCAGCGGAATGAGGCGCTGCGGACCAACTTTATTACCGGCTGG 11280
                             ******************************** ***** *********************

pmxE-D-DaB3-Polymyxin-B      AGAGACGAGCCGATTCAAGTGGTATTCCGCGAGCGGAAGTGTGAAGTGTACTTCGAAGAT 11316
pmxE_1G_Polymyxin            agagacgagccgattcaagtggtattccgcgagcggaagtgtgaagtgtacttcgaagat 11325
pmxE_B-LR_Polymyxin-E        AGGGACGAGCCGATTCAAGTGGTATTCCGCGAGCGGAAGTGTGAAGTGTACTTCGAAGAT 11340
pmxE_KP26_Polymyxin-E        AGGGACGAGCCGATTCAAGTGGTATTCCGCGAGCGGAAGTGTGAAGTGTACTTCGAAGAT 11280
pmxE_Polymyxin-P             AGAGACGAGCCGATTCAAGTGGTATTCCGCGAGCGGAAGTGTGAAGTGTACTTCGAAGAT 11340
pmxE_Polymyxin-A             AGAGACGAGCCGATTCAAGTGGTATTCCGCGAGCGGAAGTGTGAAGTGTACTTCGAAGAT 11340
                             ** *********************************************************

pmxE-D-DaB3-Polymyxin-B      ATTCGCTCGGTAAGCGATGAAGACCCGGAGAAGACGATAGCCGATTTCGTCAGCGCGGAT 11376
pmxE_1G_Polymyxin            attcgctcggtaagcgatgaagacccggagaagacgatagccgatttcgtcagcgcggat 11385
pmxE_B-LR_Polymyxin-E        ATTCGCTCGGCAAGCGATGAAGACCCGGAGAAGACGATAGCCGATTTCGTCAGCGCGGAT 11400
pmxE_KP26_Polymyxin-E        ATTCGCTCGGCAAGCGATGAAGACCCGGAGAAGACGATAGCCGATTTCGTCAGCGCGGAT 11340
pmxE_Polymyxin-P             ATTCGCTCGGTAAGCGATGAAGACCCGGAGAAGACGATAGCCGATTTCGTCAGCGCGGAT 11400
pmxE_Polymyxin-A             ATTCGTTCGGTAAGCGATGAACACCCGGAGAAGACGATAGCCGATTTTGTCAGCGCGGAT 11400
                             ***** **** ********** ************************* ************

pmxE-D-DaB3-Polymyxin-B      AAAGCGAATAAGTTCGATTTGGCCCGAGGCTCTCTTATGCGCGTGACAGTTTTGCGCACG 11436
pmxE_1G_Polymyxin            aaagcgaacaagttcgatttggcccgaggctctcttatgcgcgtgacagttttgcgcacg 11445
pmxE_B-LR_Polymyxin-E        AAAGCGAACAAGTTCGATTTGGCTCAAGGCTCTCTTATGCGCGTGACCGTTTTGCGCACG 11460
pmxE_KP26_Polymyxin-E        AAAGCGAACAAGTTCGATTTGGCTCAAGGCTCTCTTATGCGCGTGACCGTTTTGCGCACG 11400
pmxE_Polymyxin-P             AAAGCGAACAAGTTCGATTTGGCCCGAGGCTCTCTTATGCGCGTGACAGTTTTGCGCACG 11460
pmxE_Polymyxin-A             AAAGCGAACAAGTTCGATCTGGCCCGAGGTCCTCTTATGCGTGTGACCGTTGTGCGCACA 11460
                             ******** ********* **** * ***  ********** ***** *** ******* 

pmxE-D-DaB3-Polymyxin-B      GGCGACGAGTCCTACCATGTGATCTGGAGTCACCATCACATTTTGATGGACGGCTGGTGC 11496
pmxE_1G_Polymyxin            ggcgacgagtcctaccatgtgatctggagtcaccatcacattttgatggacggctggtgc 11505
pmxE_B-LR_Polymyxin-E        GGCGACGAGTCTTACCATGTGATCTGGAGTCACCATCACATTTTGATGGACGGCTGGTGC 11520
pmxE_KP26_Polymyxin-E        GGCGACGAGTCTTACCATGTGATCTGGAGTCACCATCACATTTTGATGGACGGCTGGTGC 11460
pmxE_Polymyxin-P             GGCGACGAGTCCTACCATGTGATCTGGAGTCACCATCACATTTTGATGGACGGCTGGTGC 11520
pmxE_Polymyxin-A             GGCGACGAGTCCTACCATGTGATCTGGAGTCATCATCACATTTTGATGGACGGCTGGTGT 11520
                             *********** ******************** ************************** 
```

```
pmxE-D-DaB3-Polymyxin-B      ATGTCCTTCATGATCAAGGAAGTGTTCGACACCTACTTCGCGTTCCAGGAGAAGCGGACG 11556
pmxE_1G_Polymyxin            atgtccttcatgatcaaggaagtgttcgacacctacttcgcgttccaggagaagcggacg 11565
pmxE_B-LR_Polymyxin-E        ATGTCCTTCATGATCAAGGAAGTGTTCGACACCTACTTCGCGTTCCAAGAGAAGCGGACG 11580
pmxE_KP26_Polymyxin-E        ATGTCCTTCATGATCAAGGAAGTGTTCGACACCTACTTCGCGTTCCAAGAGAAGCGGACG 11520
pmxE_Polymyxin-P             ATGTCCTTCATGATCAAGGAAGTGTTCGACACCTACTTCGCGTTCCAAGAGAAGCAGACG 11580
pmxE_Polymyxin-A             ATGTCCTTCATGATCAAGGAAGTGTTCGACACCTACTTCGCGTTCCAGGAGAAGCGGACG 11580
                             *********************************************** ******* ****

pmxE-D-DaB3-Polymyxin-B      CTGGAGCTTCCTCCGGTTACCTCGTACTCCCGGTATATCGAATGGCTGGAAGCTCAAGAT 11616
pmxE_1G_Polymyxin            ctggagcttcctccggttacctcgtactcccggtatatcgaatggctggaagctcaagat 11625
pmxE_B-LR_Polymyxin-E        CTGGAGCTTCCTCCGGTTACCTCGTACTCCCGGTATATCGAATGGCTGGAAGCTCAAGAT 11640
pmxE_KP26_Polymyxin-E        CTGGAGCTTCCTCCGGTTACCTCGTACTCCCGGTATATCGAATGGCTGGAAGCTCAAGAT 11580
pmxE_Polymyxin-P             CTGGAGCTTCCTCCGGTTACCTCGTACTCCCGGTATATCGAATGGCTGGAAGCTCAAGAT 11640
pmxE_Polymyxin-A             CTGGAGCTTCCTCCGGTTACCTCGTACTCCCGGTATATCGAATGGCTGGAAGCTCAAGAT 11640
                             ************************************************************

pmxE-D-DaB3-Polymyxin-B      GCCGCGAAAGCTTCGCGTTACTGGTCCGAGTATTTGGCGGGTTACGATCAGCAGACCAAG 11676
pmxE_1G_Polymyxin            gccgcgaaagcttcgcgttactggtccgagtatttggcgggttacgatcagcagaccaag 11685
pmxE_B-LR_Polymyxin-E        GCCGCGAAAGCTTCGCGTTACTGGTCCGAATATTTGGCGGGTTACGATCAGCAGACCAAG 11700
pmxE_KP26_Polymyxin-E        GCCGCGAAAGCTTCGCGTTACTGGTCCGAATATTTGGCGGGTTACGATCAGCAGACCAAG 11640
pmxE_Polymyxin-P             GCCGCGAAAGCTTCGCGTTACTGGTCCGAGTATTTGGCGGGTTACGATCAGCAGACCAAG 11700
pmxE_Polymyxin-A             GCCGCGAAAGCTTCGCGTTACTGGTCCGAGTATTTGGCGGGTTACGATCAGCAGACCAAG 11700
                             ***************************** ******************************

pmxE-D-DaB3-Polymyxin-B      CTGCCCCAGGAGAAAACGCAGCTGAAGCAGGGCGCTTTTGAAGCGGCTGAAATCGATGTG 11736
pmxE_1G_Polymyxin            ctgccccaggagaaaacgcagctgaagcagggcgcttttgaagcggctgaaatcgatgtg 11745
pmxE_B-LR_Polymyxin-E        CTGCCCCAGGAGAAAACGCAGCTGAAGCAGGGCGCTTTTGAAGCGGCTGAAATCGATGTG 11760
pmxE_KP26_Polymyxin-E        CTGCCCCAGGAGAAAACGCAGCTGAAGCAGGGCGCTTTTGAAGCGGCTGAAATCGATGTG 11700
pmxE_Polymyxin-P             CTGCCCCAGGAGAAAACGCAGCTGAAGCAGGGCCATTTTGAAGCGGCTGAAATCGATGTG 11760
pmxE_Polymyxin-A             CTGCCCCAGGAGAAAACGCAGCTGAAGCAGGGCGCTTTTGAAGCGGCTGAAATCGATGTG 11760
                             *********************************  *************************

pmxE-D-DaB3-Polymyxin-B      GAACTCAGCAAGGAACTGACCGGGCAAATCGAGCGGGTGGCGCGCCAGCAGCAGGTGACG 11796
pmxE_1G_Polymyxin            gaactcagcaaggaactgaccgggcaaatcgagcgggtggcgcgccagcagcaggtgacg 11805
pmxE_B-LR_Polymyxin-E        GAACTCAGCAAGGAACTGACCGGGCAAATCGAGCGGGTGGCGCGCCAGCAGCAGGTGACG 11820
pmxE_KP26_Polymyxin-E        GAACTCAGCAAGGAACTGACCGGGCAAATCGAGCGGGTGGCGCGCCAGCAGCAGGTGACG 11760
pmxE_Polymyxin-P             GAACTCAGCAAGGAACTGACCGGGCAAATCGAGCGGGTGGCGCGCCAGCAGCAGGTGACG 11820
pmxE_Polymyxin-A             GAACTCAGCAAGGAACTGACCGGGCAAATCGAGCGGGTGGCGCGCCAGCAGCAGGTGACA 11820
                             ***********************************************************

pmxE-D-DaB3-Polymyxin-B      CTCAATACGTTCATGCAGACCGTATGGGGACTGGTTCTGCAGGTATACAACAACAGCGAG 11856
pmxE_1G_Polymyxin            ctcaatacgttcatgcagaccgtatggggactggttctgcaggtatacaacaacagcgag 11865
pmxE_B-LR_Polymyxin-E        CTCAATACGTTCATGCAGACCGTATGGGGACTGGTTCTGCAGATATACAACAACAGCGAG 11880
```

```
pmxE_KP26_Polymyxin-E        CTCAATACGTTCATGCAGACCGTATGGGGACTGGTTCTGCAGATATACAACAACAGCGAG 11820
pmxE_Polymyxin-P             CTCAATACGTTCATGCAGACCGTATGGGGACTGGTTTTGCAGGTATACAACAACAGCGAG 11880
pmxE_Polymyxin-A             CTCAATACGTTTATGCAGACTGTATGGGGACTGGTTCTACAGATATACAACAACAGCGAG 11880
                             *********** ******** *************** * *** *****************

pmxE-D-DaB3-Polymyxin-B      GATGTCGTATTCGGCTCCGTCGTATCCGGGCGTCCGGCGGAAATTCCGGGCATCGAAAGC 11916
pmxE_1G_Polymyxin            gatgtcgtattcggctccgtcgtatccgggcgtccggcggaaattccgggcatcgaaagc 11925
pmxE_B-LR_Polymyxin-E        GATGTCGTATTCGGCTCCGTCGTATCCGGGCGTCCGGCGGAAATTCCGGGCATCGAAAGC 11940
pmxE_KP26_Polymyxin-E        GATGTCGTATTCGGCTCCGTCGTATCCGGGCGTCCGGCGGAAATTCCGGGCATCGAAAGC 11880
pmxE_Polymyxin-P             GATGTCGTATTCGGCTCCGTCGTATCCGGGCGTCCAGCGGAAATTCCGGGCATCGAAAGC 11940
pmxE_Polymyxin-A             GATGTCGTATTCGGCTCCGTTGTATCCGGGCGTCCGGCGGAAATTCCGGGCATCGAAAGC 11940
                             ******************** ************** ************************

pmxE-D-DaB3-Polymyxin-B      ATGATCGGCCTGTTTATTAACACGATCCCGGTCCGTATTCAAGGCAAAGCCGAGGAGACG 11976
pmxE_1G_Polymyxin            atgatcggcctgtttattaatacgatcccggtccgtattcaaggcaaagccgaggagacg 11985
pmxE_B-LR_Polymyxin-E        ATGATCGGCCTGTTTATTAATACGATCCCGGTTCGTATTCAAGGCAAAGCCGAGGAGACG 12000
pmxE_KP26_Polymyxin-E        ATGATCGGCCTGTTTATTAATACGATCCCGGTTCGTATTCAAGGCAAAGCCGAGGAGACG 11940
pmxE_Polymyxin-P             ATGATCGGCCTGTTTATTAATACGATCCCGGTCCGTATTCAAGGCAAAGCCGGGGAGACG 12000
pmxE_Polymyxin-A             ATGATTGGCCTGTTTATTAATACGATCCCGGTCCGTATTCAAGGTAAAGCCGAGGAGAGG 12000
                             ***** ************** *********** *********** ******* ***** *

pmxE-D-DaB3-Polymyxin-B      GTAGCCGATATCTTGAGAAAAACCCAGGATCAAGCGCTGGCATCGGGAGCTTACGAAACG 12036
pmxE_1G_Polymyxin            gtagccgatatcttgagaaaaacccaggatcaagcgctggcatcgggagcttacgaaacg 12045
pmxE_B-LR_Polymyxin-E        GTAGCCGATATCTTGAGAAAAACCCAGGATCAAGCACTGGCATCGGGAGCTTACGAAACG 12060
pmxE_KP26_Polymyxin-E        GTAGCCGATATCTTGAGAAAAACCCAGGATCAAGCACTGGCATCGGGAGCTTACGAAACG 12000
pmxE_Polymyxin-P             GTAGCCGATATCTTGAGAAAAACCCAGGATCAAGCGCTGGCATCGGGAGCTTACGAAACG 12060
pmxE_Polymyxin-A             GTAGCCGATATCTTGAGAAAAACCCAGGATCAAGCGCTGGCATCGGGAGCATATGAAACG 12060
                             *********************************** ************** ** ******

pmxE-D-DaB3-Polymyxin-B      TTCCCGCTGTTCGAAATTCAGTCGCTGAGCGAGCACAAGCGCGACTTGATCAACCATATC 12096
pmxE_1G_Polymyxin            ttcccgctgttcgaaattcagtcgctgagcgagcaaaagcgcgacttgatcaaccatatc 12105
pmxE_B-LR_Polymyxin-E        TTCCCGCTGTTCGAAATTCAGTCGCTGAGCGAGCAAAAGCGCGACTTGATCAACCATATT 12120
pmxE_KP26_Polymyxin-E        TTCCCGCTGTTCGAAATTCAGTCGCTGAGCGAGCAAAAGCGCGACTTGATCAACCATATT 12060
pmxE_Polymyxin-P             TTCCCGCTGTTCGAAATTCAGTCGCTGAGCGAGCAAAAGCGAGACTTGATCAACCATATC 12120
pmxE_Polymyxin-A             TTCCCACTGTTCGAAATTCAGTCCCTGAGCGAGCAAAAGCGCGACCTGATCAACCATATT 12120
                             ***** ***************** *********** ***** *** ************* 

pmxE-D-DaB3-Polymyxin-B      ATGGTCTTTGAAAATTATCCGATGGAAGAACAGATTGAGCAGGTCGTCGGTGGAGACAAA 12156
pmxE_1G_Polymyxin            atggtcttcgaaaattatccgatggaagaacagattgagcaggtcgtcggcggagacaaa 12165
pmxE_B-LR_Polymyxin-E        ATGGTTTTTGAAAATTATCCGATGGAAGAACAGATTGAGCAGGTCGTCGGCGGTGACAAA 12180
pmxE_KP26_Polymyxin-E        ATGGTTTTTGAAAATTATCCGATGGAAGAACAGATTGAGCAGGTCGTCGGCGGTGACAAA 12120
pmxE_Polymyxin-P             ATGGTCTTTGAAAATTATCCGATGGAAGAACAGATTGAGCAGGTCGTCGGCGGAGACCAA 12180
pmxE_Polymyxin-A             ATGGTCTTTGAAAATTATCCGATGGAAGAACAGATCGAGCAGGTCGTCGGTGGAGGCCGA 12180
                             ***** ** ************************** ************* ** * *  * 
```

```
pmxE-D-DaB3-Polymyxin-B      GAAGCGCTGAAAATCGCTAATATCCAGTCGCCGGAGCAAACGAACTACGACCTGGACATT 12216
pmxE_1G_Polymyxin            gaagcgctgaaaatcgctaatatccagtcgccggagcaaacgaactacgacctggacatt 12225
pmxE_B-LR_Polymyxin-E        GAAGCGCTGAAAATCGCTAATATCCAGTCGCCAGAGCAAACGAACTACGACCTGGACATT 12240
pmxE_KP26_Polymyxin-E        GAAGCGCTGAAAATCGCTAATATCCAGTCGCCAGAGCAAACGAACTACGACCTGGACATT 12180
pmxE_Polymyxin-P             GAAGCGCTGAAAATCGCTAATATCCAGTCGCCGGAGCAAACGAACTACGACCTAGACATT 12240
pmxE_Polymyxin-A             GAAGCGCTGAAAATCGCTAATATCCAGTCGCCGGAGCAAACGAACTACGACCTGGACATT 12240
                             ******************************** ******************** ******

pmxE-D-DaB3-Polymyxin-B      ACCGTCATTCCGGAAGAGCATATTTTGCTGCGGTTTACGTACAATGCGCTGACGTTCAGA 12276
pmxE_1G_Polymyxin            accgtcattccggaagagcatattttgctgcggtttacgtacaatgcgctgacgttcaga 12285
pmxE_B-LR_Polymyxin-E        ACCGTCATTCCGGAAGAGCCTATTTTGCTGCGGTTTACGTACAATGCGCTGACGTACAGA 12300
pmxE_KP26_Polymyxin-E        ACCGTCATTCCGGAAGAGCCTATTTTGCTGCGGTTTACGTACAATGCGCTGACGTACAGA 12240
pmxE_Polymyxin-P             ACCGTCATTCCGGAAGAGCATATTTTGCTGCGGTTTACGTACAATGCGCTGACGTTCAGA 12300
pmxE_Polymyxin-A             ACCGTCATTCCGGAAGAGCATATTTTGCTGCGGTTTACGTACAATGCGCTAACGTTCAGA 12300
                             ******************* ****************************** **** ****

pmxE-D-DaB3-Polymyxin-B      GAGGAAGACATCAGGCTGATCCACGGTCATTTTGCCCGGGCACTGGAGAAGGTTGCGGCT 12336
pmxE_1G_Polymyxin            gaggaagacatcaggctgatccacggtcattttgcccgggcactggagaaggttgcggct 12345
pmxE_B-LR_Polymyxin-E        GAGGAAGACATCAGGCTGATCCACGGTCATTTTGCCCAGGCACTGGAGAAGGTTGCGGCT 12360
pmxE_KP26_Polymyxin-E        GAGGAAGACATCAGGCTGATCCACGGTCATTTTGCCCAGGCACTGGAGAAGGTTGCGGCT 12300
pmxE_Polymyxin-P             GAGGAAGACATCAGGCTGATCCACGGACATTTTGCCCAAGCACTGGAGAAGGTTGCGGCT 12360
pmxE_Polymyxin-A             GAGGATGACATTAGGCAGATCCACAGTCATTTTGCCTGGGCACTGGAGAAGGTTGCGGCT 12360
                             ***** ***** **** ******* * *********   *********************

pmxE-D-DaB3-Polymyxin-B      AACCCGAGCATCCGCGTGAATCAGTTGGAGCTTTTGACGGCGGCGGAAAAAGAGCAAATT 12396
pmxE_1G_Polymyxin            aacccaaacatccgcgtgaatcagttggagcttttgacggcggcggaaaaagagcaaatt 12405
pmxE_B-LR_Polymyxin-E        AACCCGAATATCCGCGTGAATCAGTTGGAGCTTTTGACGGCGGCGGAAAAAGACCAAATT 12420
pmxE_KP26_Polymyxin-E        AACCCGAATATCCGCGTGAATCAGTTGGAGCTTTTGACGGCGGCGGAAAAAGACCAAATT 12360
pmxE_Polymyxin-P             AACCCGAACATCCGCGTGAATCAGTTGGAGCTTTTGACGGCGGCGGAAAAAGAGCAAATT 12420
pmxE_Polymyxin-A             AACCCGAACATCCTCGTGAATCAGTTGGAGCTTTTGACGGCGGCGGAAAAAGAGCAAATT 12420
                             ***** *  **** *************************************** ******

pmxE-D-DaB3-Polymyxin-B      CTTGGCGCGTTTAACCCGGCGCAGCCGGAAGCGGCTCCGGCGGTCGCGTTCCACCGGCTG 12456
pmxE_1G_Polymyxin            cttggcgcgtttaacccggcgcagccggaagcggctccggcggccgtgttccaccggctg 12465
pmxE_B-LR_Polymyxin-E        CTCGGTGCGTTTAACCCGGCGCAGCCGGAAGCGGCTCCTGCGGCCGCGTTCCACCGGCTG 12480
pmxE_KP26_Polymyxin-E        CTCGGTGCGTTTAACCCGGCGCAGCCGGAAGCGGCTCCTGCGGCCGCGTTCCACCGGCTG 12420
pmxE_Polymyxin-P             CTTGGCGCGTTTAATCCGGCGCAGCCGGAAGCGGCTCCTGCGGCCGCGTTCCACCGGCTG 12480
pmxE_Polymyxin-A             CTCGGCGCGTTTAACCCGGCGCAGCCGGAAGCGGCTCCTGCGGCCGCGTTCCACCGGCTG 12480
                             ** ** ******** *********************** **** ** *************

pmxE-D-DaB3-Polymyxin-B      TTCGAGGAACAGGCGGAGCGCACGCCGAAAGCGGCAGCTGTCGTGTACGAGAATGACCGG 12516
pmxE_1G_Polymyxin            ttcgaggaacaggcggaacgcacgccggaagcggcagctgtcgtgtacgagaatgaccgg 12525
pmxE_B-LR_Polymyxin-E        TTCGAGGAACAGGCGGAACGCACGCCGGAAGAGGCGGCCGTCGTGTATGAGAATGACCGG 12540
```

```
pmxE_KP26_Polymyxin-E        TTCGAGGAACAGGCGGAACGCACGCCGGAAGAGGCGGCCGTCGTGTATGAGAATGACCGG 12480
pmxE_Polymyxin-P             TTTGAGGAACAGGCGGAGCGCACGCCGGAAGAGGCGGCCGTCGTGTACGAGAACGACCGG 12540
pmxE_Polymyxin-A             TTCGAGGAACAGGTGGAGCGCACGCCGGAAGAGGCGGCCGTCGTGTACGAGAATGACCGG 12540
                             ** ********** *** ********* *** *** ** ******** ***** ******

pmxE-D-DaB3-Polymyxin-B      CTGACGTATGCGGAGCTGAACGAGCGGGCGAACCGCTTGGCGGCCACGCTGCGCGCAAGC 12576
pmxE_1G_Polymyxin            ctgacgtatgcggagctgaacgagcgggcgaatcgcttggcggctacgctgcgcgcaagc 12585
pmxE_B-LR_Polymyxin-E        CTGACGTATGCGGAGCTGAACGAGCGGGCGAACCGCTTGGCGGCCACGCTGCGCGCAAGC 12600
pmxE_KP26_Polymyxin-E        CTGACGTATGCGGAGCTGAACGAGCGGGCGAACCGCTTGGCGGCCACGCTGCGCGCAAGC 12540
pmxE_Polymyxin-P             CTGACGTATGCGGAGCTGAACGAGCGGGCGAACCGCTTGGCGGCTACGCTGCGCGCAAGT 12600
pmxE_Polymyxin-A             CTGACGTATGCGGAGCTGAACGAGCGGGCGAACCGCTTGGCGGCTACGCTGCGCGCAAGC 12600
                             ******************************** *********** **************

pmxE-D-DaB3-Polymyxin-B      GGCATCGGCCGGGAGACGATCGTCGGCATACTCGCCGAGCGTTCGGTGGACTTGCTGGTG 12636
pmxE_1G_Polymyxin            ggcatcggccgggagtcgatcgtcggcattctcgccgagcgttcggtggccgtgctagtg 12645
pmxE_B-LR_Polymyxin-E        GGCATCGGCCGGGAGACGATCGTCGGCATTCTCGCCGAGCGTTCGGTGGACTTGCTGGTG 12660
pmxE_KP26_Polymyxin-E        GGCATCGGCCGGGAGACGATCGTCGGCATTCTCGCCGAGCGTTCGGTGGACTTGCTGGTG 12600
pmxE_Polymyxin-P             GGCATCGGCCGGGAGACGATCGTCGGCATTCTCGCCGAGCGTTCGGTGGACTTGCTGGTG 12660
pmxE_Polymyxin-A             GGCATCGGCCGGGAGACGATCGTCGGCATTCTCGCCGAGCGTTCGGTGGACTTGCTGGTG 12660
                             *************** ************* ******************* * **** ***

pmxE-D-DaB3-Polymyxin-B      GCCGTGCTGGCCGTCTGGAAAGCGGGCGGGGCGTATGTGCCGCTCGACCCGGATTACCCG 12696
pmxE_1G_Polymyxin            gccgtgctggccgtctggaaagcgggcggggcgtatgtgccgctcgacccggattacccg 12705
pmxE_B-LR_Polymyxin-E        GCCGTGCTGGCCGTCTGGAAAGCGGGCGGGGCATATGTGCCGCTCGACCCGGATTATCCG 12720
pmxE_KP26_Polymyxin-E        GCCGTGCTGGCCGTCTGGAAAGCGGGCGGGGCATATGTGCCGCTCGACCCGGATTATCCG 12660
pmxE_Polymyxin-P             GCCGTGCTGGCCGTCTGGAAAGCGGGCGGGGCGTATGTGCCGCTGGACCCGGATTATCCG 12720
pmxE_Polymyxin-A             GCCGTGCTGGCCGTCTGGAAAGCGGGCGGGGCGTATGTGCCGCTCGACCCGGATTATCCG 12720
                             ******************************** *********** *********** ***

pmxE-D-DaB3-Polymyxin-B      GCGGAGCGCGTGCGGTTCATGCTCGAAGACAGCGGAGCGAAGGTACTGCTGACGCAAACG 12756
pmxE_1G_Polymyxin            gcggagcgcgtgcggttcatgcttgaagacagcggagcgaaggtgctgctgacgcaaacg 12765
pmxE_B-LR_Polymyxin-E        GCAGACCGCGTGCGGTTCATGCTTGAAGACAGTGGAGCGAAGGTACTGTTGACGCAAATA 12780
pmxE_KP26_Polymyxin-E        GCAGACCGCGTGCGGTTCATGCTTGAAGACAGTGGAGCGAAGGTACTGTTGACGCAAATA 12720
pmxE_Polymyxin-P             GCGGAGCGCGTGCGGTTCATGCTTGAAGACAGCGGAGCGAAGGTACTGCTGACGCAAACG 12780
pmxE_Polymyxin-A             GCAGACCGCGTGCGGTTCATGCTTGAAGACAGCGGAGCGAAGGTGCTGCTGACGCAAACG 12780
                             ** ** ***************** ******** *********** *** *********

pmxE-D-DaB3-Polymyxin-B      CCGCTGCGAGAGCGTGCCGAAGCCTGGCTCGGCGAAGAGGAGCTGGCGCTGGCGGCGGTG 12816
pmxE_1G_Polymyxin            ccgctgcgagagcgcgccgaagcctggctcggcgaagaggagctggcgctggcggcggtg 12825
pmxE_B-LR_Polymyxin-E        CCGCTGCGAGAACGCGCCGAAGCCTGGCTCGGCGAAGAGGAGCTGGCGCTGGCAGCGGTG 12840
pmxE_KP26_Polymyxin-E        CCGCTGCGAGAACGCGCCGAAGCCTGGCTCGGCGAAGAGGAGCTGGCGCTGGCAGCGGTG 12780
pmxE_Polymyxin-P             CCGCTGCGAGAGCGTGCTGAAGCCTGGCTTGGCGAAGAGGAGCTGGCGCTGGCGGCGGTG 12840
pmxE_Polymyxin-A             GCGCTGCGAGAGCGTGCCGAAGCCTGGCTCGGCGAAGAGGAGCTGGCGCTGGCGGCGGTG 12840
                              ********** ** ** *********** *********************** ******
```

```
pmxE-D-DaB3-Polymyxin-B      CTGTACCTGGACGACGAAGCGTCGTACAGCGAGGAGCGGGCGAATGCGCCGATT------ 12870
pmxE_1G_Polymyxin            ctgtacctcgacgacgaagcgtcgtacagcgacgagcgggcgaatgcgccgctg------ 12879
pmxE_B-LR_Polymyxin-E        CTGTACCTCGACGACGAAGCGTCGTACAGCGAGGAGCGGGCGAATGCGCCGATTGGTTCC 12900
pmxE_KP26_Polymyxin-E        CTGTACCTCGACGACGAAGCGTCGTACAGCGAGGAGCGGGCGAATGCGCCGATTGGTTCC 12840
pmxE_Polymyxin-P             CTGTACCTGGACGACGAAGCGTCGTACAGCGAGGAGCGGGCGAATGCGCCGATTGGCTCC 12900
pmxE_Polymyxin-A             CTGTACCTGGACGACGAAGCGTCGTACAACGAGGAGCGGGCGAATGCGCCGGTTGGCTCC 12900
                             ******** ****************** *** ******************* *

pmxE-D-DaB3-Polymyxin-B      ------GTCTCCGGCAAGCTGACGGATGCTGTGGACGACGGCGATGAGAGCCATCAGAAT 12924
pmxE_1G_Polymyxin            ------gaagcggatgatca---------------------------------------- 12893
pmxE_B-LR_Polymyxin-E        GGCATGGTCTCCGGCCAGCTGACGGATGCTGTGGATGACGGCGATGAGACCCATCCGAAT 12960
pmxE_KP26_Polymyxin-E        GGCATGGTCTCCGGCCAGCTGACGGATGCTGTGGATGACGGCGATGAGACCCATCCGAAT 12900
pmxE_Polymyxin-P             GGCATAGTCTCCGGCAAGCTGACGGATGCTGTGGACGACGGCGATGAGAGCCATCAGAAT 12960
pmxE_Polymyxin-A             GGCATGGTCTCCGGCAAGCTGACGGATGCTGTGGACGACGGCGATGAGAGCCATCAGAAT 12960
                                   *   * *   * *

pmxE-D-DaB3-Polymyxin-B      GTTGGCACCGACAGCTTCCATGAATGCCGTCCGGAGGACCTGGCGTACGTGATCTACACG 12984
pmxE_1G_Polymyxin            --------------cttgcacgaataccgtccggaggacctggcgtacgtgatctatacg 12939
pmxE_B-LR_Polymyxin-E        ATTGGCATGGGCAGCTTCCATGAAGCTCGTCCGGAGGATCTGGCGTATGTGATCTATACG 13020
pmxE_KP26_Polymyxin-E        ATTGGCATGGGCAGCTTCCATGAAGCTCGTCCGGAGGATCTGGCGTATGTGATCTATACG 12960
pmxE_Polymyxin-P             ATTGGCACCGACAGCTTCCATGAAGCCCGTCCGGAGGAACTGGCGTACGTGATCTATACG 13020
pmxE_Polymyxin-A             GTTGGCACCGACGGCTTCCATGAAGCCCGTCCGGAGGACCTGGCGTACGTGATCTATACG 13020
                                           *** ** ***   *********** ******** ******** ***

pmxE-D-DaB3-Polymyxin-B      TCGGGAACGACGGGCAAGCCGAAGGGCGTGATGATCGAGCACCGCAGCCTGGTGAATACG 13044
pmxE_1G_Polymyxin            tcgggaacgacgggcaagccgaagggcgtgatgatcgagcaccgcagcctggtgaacacg 12999
pmxE_B-LR_Polymyxin-E        TCGGGAACGACGGGCAAGCCGAAAGGCGTGATGATCGAGCACCGCAGCCTGGTGAACACG 13080
pmxE_KP26_Polymyxin-E        TCGGGAACGACGGGCAAGCCGAAAGGCGTGATGATCGAGCACCGCAGCCTGGTGAACACG 13020
pmxE_Polymyxin-P             TCGGGAACGACGGGCAAGCCGAAGGGCGTGATGATCGAGCACCGCAGCCTGGTGAACACG 13080
pmxE_Polymyxin-A             TCGGGAACGACGGGCAAGCCGAAGGGCGTGATGATCGAGCACCGCAGCCTGGTGAACACG 13080
                             *********************** ******************************** ***

pmxE-D-DaB3-Polymyxin-B      GCGGCGGGCTACCGGCGGGAATACCGGTTGGATCAGTTCCCGGTGCGGCTGCTGCAGCTC 13104
pmxE_1G_Polymyxin            gcggcgggctaccggcgggaataccggttggatcagttcccggtgcggctgctgcagctc 13059
pmxE_B-LR_Polymyxin-E        GCAGCGGGCTACCGGCGGGAATACCGGTTGGATCAGTTCCCGGTACGGCTGCTGCAGCTC 13140
pmxE_KP26_Polymyxin-E        GCAGCGGGCTACCGGCGGGAATACCGGTTGGATCAGTTCCCGGTACGGCTGCTGCAGCTC 13080
pmxE_Polymyxin-P             GCGGCGGGCTACCGGCGGGAATACCGGTTGAACCAGTTCCCGGTGCGGCTGCTGCAGCTC 13140
pmxE_Polymyxin-A             GCGGCGGGCTACCGGCGGGAATACCGGTTGGATCAGTTCCCGGTGCGGCTGCTGCAGCTC 13140
                             ** *************************** * *********** ***************

pmxE-D-DaB3-Polymyxin-B      GCCAGCTTCTCGTTCGACGTGTTCGTGGGAGATATCGCGCGGACGCTGTACAACGGAGGC 13164
pmxE_1G_Polymyxin            gcaagcttctcgttcgacgtgttcgtgggagatatcgcgcggacgctgtacaacggaggc 13119
pmxE_B-LR_Polymyxin-E        GCAAGCTTCTCGTTCGACGTGTTCGTGGGAGATATCGCGCGGACGCTGTATAACGGAGGC 13200
```

```
pmxE_KP26_Polymyxin-E        GCAAGCTTCTCGTTCGACGTGTTCGTGGGAGATATCGCGCGGACGCTGTATAACGGAGGC 13140
pmxE_Polymyxin-P             GCAAGCTTCTCGTTCGACGTGTTCGTGGGAGATATCGCGCGGACGCTGTACAACGGAGGC 13200
pmxE_Polymyxin-A             GCAAGCTTCTCGTTCGACGTGTTCGTGGGAGATATCGCGCGGACGCTGTACAACGGAGGT 13200
                             ** *********************************************** ********

pmxE-D-DaB3-Polymyxin-B      ACGATGGTGATTGTGCCGAAGGACGACCGGATCGATCCGTCTCGTCTGCACCACTGGATT 13224
pmxE_1G_Polymyxin            acgatggtgattgtgccgaaggacgaccggatcgatccgtctcgtctgcaccactggatt 13179
pmxE_B-LR_Polymyxin-E        ACGATGGTGATTGTGCCGAAGGACGATCGGATCGATCCGTCTCGTCTGCACCACTGGATG 13260
pmxE_KP26_Polymyxin-E        ACGATGGTGATTGTGCCGAAGGACGATCGGATCGATCCGTCTCGTCTGCACCACTGGATG 13200
pmxE_Polymyxin-P             ACGATGGTGATCGTGCCGAAGGACGACCGGATCGATCCGTCTCGTCTGCACTACTGGATT 13260
pmxE_Polymyxin-A             ACGATGGTGATTGTGCCGAAGGACGATCGGATTGATCCGTCTCGTCTGCACCACTGGATG 13260
                             *********** ************** ***** ****************** *******

pmxE-D-DaB3-Polymyxin-B      GAGCGGGAGCGGGTCACCATCTTCGAATCGACGCCGGCGCTGATCGTGCCGTTCCTGGAG 13284
pmxE_1G_Polymyxin            gagcgggagcggatcacaatcttcgaatcgacgccggcgctgatcgtgccgttcctggag 13239
pmxE_B-LR_Polymyxin-E        GAGCGGGAGCGGGTCACCATTTTCGAATCAACGCCGGCGCTGATCGTGCCGTTCTTAGAG 13320
pmxE_KP26_Polymyxin-E        GAGCGGGAGCGGGTCACCATTTTCGAATCAACGCCGGCGCTGATCGTGCCGTTCTTAGAG 13260
pmxE_Polymyxin-P             GAGCGGGAGCAGGTCACCATCTTCGAATCGACGCCGGCGCTGATCGTGCCGTTCCTGGAG 13320
pmxE_Polymyxin-A             GAGCGGGAGCGGGTCACCATCTTCGAATCGACGCCGGCGCTGATCGTGCCGTTCCTGGAG 13320
                             ********** * **** ** ******** ************************ * ***

pmxE-D-DaB3-Polymyxin-B      TACGTGCACGAGCAGCAACTGGATATGAGCTGGATGGAGCTGTTGATCACGAGCTCGGAC 13344
pmxE_1G_Polymyxin            tacgtgcacgagcagcaactggatatgagccggttggagctgttgatcacgagctcggac 13299
pmxE_B-LR_Polymyxin-E        TACGTGCACGAGCAGGGGCTGGATATGAGCTGGATGGAGCTGTTGATCACGAGTTCGGAC 13380
pmxE_KP26_Polymyxin-E        TACGTGCACGAGCAGGGGCTGGATATGAGCTGGATGGAGCTGTTGATCACGAGTTCGGAC 13320
pmxE_Polymyxin-P             TACGTGCACGAGCAGCGGCTGGATATGAGCTGGATGGAGCTGTTGATCACGAGCTCGGAC 13380
pmxE_Polymyxin-A             TACGTGCACGATCAGCAGCTGGATATGAGCTGGATGGAGCTGTTGATCACGAGCTCGGAC 13380
                             *********** ***   ************ ** ******************* ******

pmxE-D-DaB3-Polymyxin-B      AGCTGCAGCGTGGCGGATTACCGGACCTTGCAGGAACGCTTCGGCTCGTTGTTCCGGATC 13404
pmxE_1G_Polymyxin            agctgcagcgtggcggattaccggaccttgcaggaacgcttcggctcgttgttccggatc 13359
pmxE_B-LR_Polymyxin-E        AGCTGCAGCGTGGCGGATTACCGGACCTTGCAGGAACGCTTCGGCTCGTTATTCCGGATC 13440
pmxE_KP26_Polymyxin-E        AGCTGCAGCGTGGCGGATTACCGGACCTTGCAGGAACGCTTCGGCTCGTTATTCCGGATC 13380
pmxE_Polymyxin-P             AGCTGCAGCGTGGCGGATTACCGGACCTTGCAGGAACGCTTCGACTCGTTGTTCCGGATC 13440
pmxE_Polymyxin-A             AGCTGCAGCGTGGCGGATTACCGGACCTTGCAGGAACGCTTCGGCTCGTTGTTCCGAATC 13440
                             ******************************************* ****** ***** ***

pmxE-D-DaB3-Polymyxin-B      ATCAACGCATACGGCGTGACGGAAGCGGCGATCGACTCAAGCTTCTACGACGAGGAGCTG 13464
pmxE_1G_Polymyxin            atcaacgcttacggcgtgacggaagcggcgatcgactcaagtttctacgacgagccgctg 13419
pmxE_B-LR_Polymyxin-E        ATCAACGCATACGGCGTGACGGAAGCGGCGATCGACTCCAGCTTCTACGACGAGGAGCTG 13500
pmxE_KP26_Polymyxin-E        ATCAACGCATACGGCGTGACGGAAGCGGCGATCGACTCCAGCTTCTACGACGAGGAGCTG 13440
pmxE_Polymyxin-P             ATCAACGCATACGGCGTGACGGAAGCGGCGATCGACTCCAGCTTCTACGACGAGGAGCTG 13500
pmxE_Polymyxin-A             ATCAACGCCTACGGCGTGACGGAAGCGGCGATCGACTCCAGCTTCTACGACGAGGAGCTG 13500
                             ******** ***************************** ** ************  ****
```

```
pmxE-D-DaB3-Polymyxin-B      GCGAAGCTGCCGCAGACAGGCAATGTGCCGATCGGCAAAGCGTGGCTGAATGCGAAATTC 13524
pmxE_1G_Polymyxin            acgaagctgccgcagacaggccatgtgccgatcggcaaagcgtggctgaatgcgaaattc 13479
pmxE_B-LR_Polymyxin-E        ACGAAGCTGCCGCAGACAGGACATGTGCCGATCGGTAAAGCGTGGCTGAATGCGAAATTC 13560
pmxE_KP26_Polymyxin-E        ACGAAGCTGCCGCAGACAGGACATGTGCCGATCGGTAAAGCGTGGCTGAATGCGAAATTC 13500
pmxE_Polymyxin-P             ACGAAGCTACCGCAGACAGGTAATGTGCCGATCGGTAAAGCGTGGCTGAATGCGAAATTC 13560
pmxE_Polymyxin-A             GCGAAGCTGCCGCAGACAGGCCATGTGCCGATCGGCAAAGCGTGGCTGAATGCGAAATTC 13560
                              ******* ***********  ************* ************************

pmxE-D-DaB3-Polymyxin-B      TACATCGTGGACGCGCATCTGAACCCGGTGCCGGTCGGGGTGCTGGGCGAGCTGGTCATC 13584
pmxE_1G_Polymyxin            tacatcgtggacgcgcatctgaacccggtgccggtcggggtgctgggcgagctggtcatc 13539
pmxE_B-LR_Polymyxin-E        TACATCGTGGACGCGCATCTGAACCCGGTGCCGGTCGGGGTGCTGGGCGAGCTGGTCATT 13620
pmxE_KP26_Polymyxin-E        TACATCGTGGACGCGCATCTGAACCCGGTGCCGGTCGGGGTGCTGGGCGAGCTGGTCATT 13560
pmxE_Polymyxin-P             TACATCGTGGATGCGCATCTGAATCCGGTGCCGGTCGGGGTGCTGGGCGAGCTGGTCATC 13620
pmxE_Polymyxin-A             TACATCGTGGACGCGCATCTGAACCCGGTGCCAGTCGGGGTGCTGGGCGAGCTGGTCATC 13620
                             *********** *********** ******** **************************

pmxE-D-DaB3-Polymyxin-B      GGCGGAGTCGGAGTGGCGCGCGGGTACTTGAACCGTCCGGAGCTGACAGAAGAGAAGTTC 13644
pmxE_1G_Polymyxin            ggcggagtcggggtagcgcgcgggtacttgaaccgtccggagctgacagaagagaagttc 13599
pmxE_B-LR_Polymyxin-E        GGCGGAGTCGGGGTAGCACGCGGGTACTTGAACCGTCCGGAGCTGACGGAAGAGAAGTTC 13680
pmxE_KP26_Polymyxin-E        GGCGGAGTCGGGGTAGCACGCGGGTACTTGAACCGTCCGGAGCTGACGGAAGAGAAGTTC 13620
pmxE_Polymyxin-P             GGCGGAGTCGGAGTGGCGCGCGGGTACTTGAACCGTCCGGAGCTGACGGAAGAAAAGTTC 13680
pmxE_Polymyxin-A             GGCGGAGTCGGAGTGGCGCGCGGGTACTTGAACCGTCCGGAACTGACGGGAGAGAAGTTC 13680
                             *********** ** ** *********************** ***** * *** ******

pmxE-D-DaB3-Polymyxin-B      GTAGACAGCCCGTTCGCCGCGGGCGAGCGGCTGTACCGCACGGGAGACTTGGCGCGGTGG 13704
pmxE_1G_Polymyxin            gtagacagcccgttcgccgcgggcgagcggctgtaccgcacgggagacttggcgcggtgg 13659
pmxE_B-LR_Polymyxin-E        GTAGACAGTCCGTTCGCCGCGGGCGAGCGGCTGTACCGCACGGGAGACTTGGCGCGGTGG 13740
pmxE_KP26_Polymyxin-E        GTAGACAGTCCGTTCGCCGCGGGCGAGCGGCTGTACCGCACGGGAGACTTGGCGCGGTGG 13680
pmxE_Polymyxin-P             GTAGACAGTCCGTTCGCCGCGGGCGAGCGGCTGTACCGCACGGGAGACTTGGCGCGATGG 13740
pmxE_Polymyxin-A             GTAGACAGCCCGTTCGCCGCGGGCGAGCGGCTGTACCGCACGGGAGACTTGGCGCGGTGG 13740
                             ******** *********************************************** ***

pmxE-D-DaB3-Polymyxin-B      ATGGAAGACGGCAACGTGGACTTCATCGGCCGGATCGACAACCAGGCGAAAATCCGGGGG 13764
pmxE_1G_Polymyxin            atggaggacgggaacgtggacttcatcggccggatcgacaaccaggcgaaaatccggggg 13719
pmxE_B-LR_Polymyxin-E        ATGGAGGACGGGAACGTGGACTTCATCGGCCGGATCGACAACCAGGCGAAAATCCGGGGC 13800
pmxE_KP26_Polymyxin-E        ATGGAGGACGGGAACGTGGACTTCATCGGCCGGATCGACAACCAGGCGAAAATCCGGGGC 13740
pmxE_Polymyxin-P             ATGGAGGACGGCAACGTGGACTTCATCGGCCGGATCGACAACCAGGCGAAAATCCGGGGG 13800
pmxE_Polymyxin-A             ATGGAGGACGGGAACGTGGACTTCATCGGCCGGATCGACAACCAGGCGAAAATCCGGGGG 13800
                             ***** ***** ***********************************************

pmxE-D-DaB3-Polymyxin-B      TACCGGATTGAGACGGGCGAAGTAGAAGCGAAGCTGCTGAGTGTAGGTGGCGTGAAGGAA 13824
pmxE_1G_Polymyxin            taccggattgagacgggcgaagtcgaagcgaagctgctgagtgtaggtggcgtgaaggaa 13779
pmxE_B-LR_Polymyxin-E        TACCGGATTGAGACGGGCGAAGTCGAAGCGAAGATGCTGAGTGTAGGTGGTGTGAAGGAA 13860
```

```
pmxE_KP26_Polymyxin-E       TACCGGATTGAGACGGGCGAAGTCGAAGCGAAGATGCTGAGTGTAGGTGGTGTGAAGGAA 13800
pmxE_Polymyxin-P            TACCGGATTGAGACGGGCGAAGTCGAAGCGAAGCTGCTGAGTGTAGGTGGCGTGAAGGAA 13860
pmxE_Polymyxin-A            TACCGGATTGAGACGGGCGAAGTCGAAGCAAAGCTGCTGAGTGTAGGTGGCGTGAAGGAA 13860
                            *********************** ***** *** **************** *********

pmxE-D-DaB3-Polymyxin-B     GCGGTCGTCGTCGTCAGGGAAGATCAAGAAGGTCAGAAAGCTTTGTGCGCTTATTATACA 13884
pmxE_1G_Polymyxin           gcggtcgtcgtcgtcagggaagatcaagaaggtcagaaagctttgtgcgcttattataca 13839
pmxE_B-LR_Polymyxin-E       GCGGTCGTTGTCGTCAGGGAAGATCAAGAAGGTCAGAAAGCTTTGTGCGCTTATTATACA 13920
pmxE_KP26_Polymyxin-E       GCGGTCGTTGTCGTCAGGGAAGATCAAGAAGGTCAGAAAGCTTTGTGCGCTTATTATACA 13860
pmxE_Polymyxin-P            GCGGTCGTCGTCGTCAGGGAAGATCAAGAAGGTCAGAAAGCTTTGTGCGCTTATTATACA 13920
pmxE_Polymyxin-A            GCGGTCGTCGTCGTCAGGGAAGATCAAGAAGGTCAGAAAGCTTTGTGCGCTTATTATACA 13920
                            ******** ***************************************************

pmxE-D-DaB3-Polymyxin-B     GCGGAAGAAGGCTTGACGGCGGCGGACCTGAAGCGGGCGATTGCCAGCGAGCTGCCGGGG 13944
pmxE_1G_Polymyxin           gcggaagaaggcttgacggcggcggacctgaagcgggcgattgccagcgagctgccgggt 13899
pmxE_B-LR_Polymyxin-E       GTGGAAGAAGGCATGACGGCGGCAGACCTGAAGCGTGCGATTTCCAGCGAGCTGCCGGGG 13980
pmxE_KP26_Polymyxin-E       GTGGAAGAAGGCATGACGGCGGCAGACCTGAAGCGTGCGATTTCCAGCGAGCTGCCGGGG 13920
pmxE_Polymyxin-P            GCGGAAGAAGGCTTGACGGCGTTGGACCTGAAGCGGGCGATTGCCAGCGAGCTGCCGGGG 13980
pmxE_Polymyxin-A            GCGGAAGAAGGCTTGACGGCGGCGGATCTGAAGCGGGCGATTGCCAGCGAGCTGCCGGGG 13980
                            * ********** ********   ** ******** ****** ****************

pmxE-D-DaB3-Polymyxin-B     TACATGATCCCGTCGTACTTCGTGGAGCTGGAGCGCCTGCCTCTGACGCCGAACGGGAAA 14004
pmxE_1G_Polymyxin           tacatgatcccgtcgtacttcgtggagctggagcgcctgcctctgacgccgaacggaaag 13959
pmxE_B-LR_Polymyxin-E       TACATGATCCCGTCGTATTTCGTGGAGCTGGAGCGTCTGCCTTTGACGCCGAACGGAAAG 14040
pmxE_KP26_Polymyxin-E       TACATGATCCCGTCGTATTTCGTGGAGCTGGAGCGTCTGCCTTTGACGCCGAACGGAAAG 13980
pmxE_Polymyxin-P            TACATGATCCCGTCGTACTTCGTGGAGATGGAGCGCCTGCCTCTGACGCCGAACGGAAAG 14040
pmxE_Polymyxin-A            TACATGATCCCGTCGTACTTCGTGGAGCTGGAGCGCCTGCCTCTGACGCCGAACGGAAAG 14040
                            ***************** ********* ******* ****** ************* **

pmxE-D-DaB3-Polymyxin-B     ATCGACCGGAAGGCGCTGCCGTCACCGGAAGGGGGAGCAGGCGGAGGCCGCGAATACGTG 14064
pmxE_1G_Polymyxin           atcgaccgtaaggcgctgccggcaccggaagggggagcaggcggaggccgcgaatacgtg 14019
pmxE_B-LR_Polymyxin-E       ATCGACCGGAAGGCGCTGCCGGCACCGGAAGGGGCAGCAGGCGGAGGCCGCGAATACGTG 14100
pmxE_KP26_Polymyxin-E       ATCGACCGGAAGGCGCTGCCGGCACCGGAAGGGGCAGCAGGCGGAGGCCGCGAATACGTG 14040
pmxE_Polymyxin-P            ATCGACCGGAAGGCGCTGCCAGCACCGGAAGGGGGAGCAAGCGCAGGCCGCGAATACGTG 14100
pmxE_Polymyxin-A            ATCGACCGGAAGGCGCTGCCGGCACCGGAAGGGGGAGCAGGCGGAGGCCGCGAATACGTG 14100
                            ******** ***********  ************ **** *** ****************

pmxE-D-DaB3-Polymyxin-B     GCGCCGCGCACCGAACTGGAGGCGAAGCTGGCCGCCATTTGGCAGGATGTGCTTGTTCGG 14124
pmxE_1G_Polymyxin           gagccgcgcaccgaacttgaggcgaagctggccgccatttggcaggatgtgcttgttcgg 14079
pmxE_B-LR_Polymyxin-E       GCGCCACGCACCGAACTGGAGGCGAAGCTGGCCGCCATTTGGCAGGAGGTGCTTGTTAGG 14160
pmxE_KP26_Polymyxin-E       GCGCCACGCACCGAACTGGAGGCGAAGCTGGCCGCCATTTGGCAGGAGGTGCTTGTTAGG 14100
pmxE_Polymyxin-P            GCGCCGCGCACCGAACTTGAGGCGAAGCTGGCCGCCATTTGGCAGGATGTACTTGTTCGG 14160
pmxE_Polymyxin-A            GCGCCGCGCACCGAACTTGAGGCGAAGCTGGCCGCCATTTGGCAGGATGTGCTTGTTCGG 14160
                            * *** *********** ***************************** ** ****** **
```

```
pmxE-D-DaB3-Polymyxin-B      GAGAAGGCGGTAGGCGTAACGGACAACTTCTTCGACCTCGGCGGACACTCCCTGCGGGCG 14184
pmxE_1G_Polymyxin            gagaaggcggtaggcgtaacggacaacttcttcgacctcggcggacactccctgcgggcg 14139
pmxE_B-LR_Polymyxin-E        GAGAAGGCAGTAGGTGTAACGGACAACTTCTTTGACCTCGGCGGACACTCCCTGCGGGCT 14220
pmxE_KP26_Polymyxin-E        GAGAAGGCAGTAGGTGTAACGGACAACTTCTTTGACCTCGGCGGACACTCCCTGCGGGCT 14160
pmxE_Polymyxin-P             GAGAAGGCGGTAGGCGTAACGGACAACTTCTTCGACCTCGGCGGACACTCCCTGCGGGCG 14220
pmxE_Polymyxin-A             GAGAAGGCGGTAGGCGTAACGGACAACTTCTTCGACCTCGGCGGACACTCCCTGCGGGCA 14220
                             ******** ***** ***************** **************************

pmxE-D-DaB3-Polymyxin-B      ACGACGCTTGTCAGCAAAATGCATAAGGAGCTAGGCG-TGAATTCCCGCTTCGCGACGTA 14243
pmxE_1G_Polymyxin            acgacgcttgtcagcaaaatgcataaggagctaggcgttgaattcccgcttcgcgacgta 14199
pmxE_B-LR_Polymyxin-E        ACGACGCTTGTCAGCAAAATGCATAAGGAGCTAGGCATTGAATTCCCGCTACGCGACGTA 14280
pmxE_KP26_Polymyxin-E        ACGACGCTTGTCAGCAAAATGCATAAGGAGCTAGGCATTGAATTCCCGCTACGCGACGTA 14220
pmxE_Polymyxin-P             ACGACGCTTGTCAGCAAAATGCATAAGGAGCTAGGCGTTGAATTCCCGCTTCGCGACGTA 14280
pmxE_Polymyxin-A             ACGACGCTTGTCAGCAAAATGCATAAGGAGCTAGGCGTTGAATTCCCGCTTCGCGACGTA 14280
                             ************************************  ************ *********

pmxE-D-DaB3-Polymyxin-B      TTCCGCTACTCGACGGTTGAGGAAATGGCCGCCGGCTATGGAGCGGCTGGAGATCGGCTC 14303
pmxE_1G_Polymyxin            ttccgctactcgacggttgaggaaatggccg-cggctatggagcggctggagatcggctc 14258
pmxE_B-LR_Polymyxin-E        TTCCGCTACTCGACGGTTGAGGAAATGGCCG-CGGCTATGGAGTGGCTGGAGATCGGCTC 14339
pmxE_KP26_Polymyxin-E        TTCCGCTACTCGACGGTTGAGGAAATGGCCG-CGGCTATGGAGTGGCTGGAGATCGGCTC 14279
pmxE_Polymyxin-P             TTCCGCTACTCGACGGTTGAGGAAATGGCCG-CGGCTATGGAGCGGCTGGAGATCGGCTC 14339
pmxE_Polymyxin-A             TTCCGCTACCCGACGGTTGAGGAAATGGCCG-CGGCTATGGAGCGGCTGGAGATCGGCTC 14339
                             ********* ********************* *********** ****************

pmxE-D-DaB3-Polymyxin-B      GTTCACGGCAATTCCCGTTGCGGAACCTGGCGAGTATTATCCGCTCTCTTCCGCTCAGAA 14363
pmxE_1G_Polymyxin            gttcacggctattccggctgcggaacctagcgagtattatccgctctcttccgctcagaa 14318
pmxE_B-LR_Polymyxin-E        GTTCATAGCTATTCCGGCTGCGGAACCTAGCGAGTATTATCCGCTATCATCCGCTCAGAA 14399
pmxE_KP26_Polymyxin-E        GTTCATAGCTATTCCGGCTGCGGAACCTAGCGAGTATTATCCGCTATCATCCGCTCAGAA 14339
pmxE_Polymyxin-P             GTTCACGGCTATTCCGGCTGCGGAACCTAGCGAGTATTATCCGCTCTCTTCCGCTCAGAA 14399
pmxE_Polymyxin-A             GTTCATGGCTATTCCGGCTGCGGAACCTAGCGAGTATTATCCGCTCTCTTCCGCTCAGAA 14399
                             *****  ** ***** * ********** **************** ** ***********

pmxE-D-DaB3-Polymyxin-B      ACGTCTCTATATTTTGAACCAGCTGGAAGGAGCCGAGCTGAGCTACAACATACCGGGAGC 14423
pmxE_1G_Polymyxin            acgtctctatatcttgaaccagctggaaggagccgagctgagctacaacataccgggagc 14378
pmxE_B-LR_Polymyxin-E        ACGTCTCTATATCTTGAACCAGCTGGAAGGAGGCGAGCTGAGCTACAACATACCGGGAGC 14459
pmxE_KP26_Polymyxin-E        ACGTCTCTATATCTTGAACCAGCTGGAAGGAGGCGAGCTGAGCTACAACATACCGGGAGC 14399
pmxE_Polymyxin-P             ACGTCTCTATATCTTGAACCAGCTGGAAGGAGCCGAGCTGAGTTACAACATACCGGGAGC 14459
pmxE_Polymyxin-A             ACGTCTCTATATCTTGAACCAGCTGGAAGGAGCCGAGCTGAGCTACAACATACCGGGAGC 14459
                             ************ ******************* ********* *****************

pmxE-D-DaB3-Polymyxin-B      TATGCTGCTCGAAGGGGAGCTCGACCGGCAGCGGTTTGAAGAAGCGTTCCGCGGGCTCGT 14483
pmxE_1G_Polymyxin            tatgctgctcgaaggggagctcgaccggcagcggtttgaagaagcgttccgcgggctcgt 14438
pmxE_B-LR_Polymyxin-E        AATGCTGCTCGAAGGGGAGCTCGACCGGCAGCGGTTTGAAGAAGCGTTCCGCGGGCTCGT 14519
```

```
pmxE_KP26_Polymyxin-E         AATGCTGCTCGAAGGGGAGCTCGACCGGCAGCGGTTTGAAGAAGCGTTCCGCGGGCTCGT 14459
pmxE_Polymyxin-P              TATGCTACTCGAAGGAGGGCTCGACCGGCAGCGGTTTGAAGAAGCGTTCCGCGGGCTCGT 14519
pmxE_Polymyxin-A              TATGCTGCTCGAAGGGGAGCTCGACCGGCAGCGGTTTGAAGAAGCGTTCCGCGGGCTCGT 14519
                               ***** ******** * ******************************************

pmxE-D-DaB3-Polymyxin-B       AGCTCGTCATGAAACGCTGCGTACCGGCTTTGAGATGGTAAAAGGCGAAGCGGTTCAGCG 14543
pmxE_1G_Polymyxin             agctcgtcatgaaacgctgcgtaccggctttgagatggtaaaaggcgaagcggttcagcg 14498
pmxE_B-LR_Polymyxin-E         AGCTCGTCATGAAACGCTGCGTACCGGATTTGAGATGGTAAAAGGCGAAGCGGTTCAACG 14579
pmxE_KP26_Polymyxin-E         AGCTCGTCATGAAACGCTGCGTACCGGATTTGAGATGGTAAAAGGCGAAGCGGTTCAACG 14519
pmxE_Polymyxin-P              AGCTCGTCATGAAACGCTGCGTACCGGCTTTGAGATGGTAAAAGGCGAAGCGGTTCAGCG 14579
pmxE_Polymyxin-A              AGCTCGCCATGAAACGCTGCGTACCGGCTTTGAGATGGTAAAAGGCGAAGCGGTTCAGCG 14579
                              ****** ******************** ***************************** **

pmxE-D-DaB3-Polymyxin-B       GATTTACGAAGAAGCCGCCTTTCAGGTGGAATATGTGCAGATCAGCGGGGAGCAGGCGGA 14603
pmxE_1G_Polymyxin             gatttacgaagaagccgccttccaggtggaatatgtgcagatcagcggggagcaggcgga 14558
pmxE_B-LR_Polymyxin-E         GATTTATGAAGAAGCTGCTTTCCAGGTGGAATATGTGCAGATTAGCGGGGAACGGGTGGA 14639
pmxE_KP26_Polymyxin-E         GATTTATGAAGAAGCTGCTTTCCAGGTGGAATATGTGCAGATTAGCGGGGAACGGGTGGA 14579
pmxE_Polymyxin-P              GATTTACGAAGAAGTCGCCTTCCAGGTGGAATATGTGCAGATCAGCGGGGAGCAGGCGGA 14639
pmxE_Polymyxin-A              GATTTACGAAGAAGCCGCCTTCCAGGTGGAATATGTGCAGATCAGCGCGGAGCAGGCGGA 14639
                              ****** *******  ** ** ******************** **** *** * ** ***

pmxE-D-DaB3-Polymyxin-B       AGAAACGGTGCGCCAATTCGTCCGTCCGTTTGATCTAGCGAAGCCGCCACTTCTGCGGGT 14663
pmxE_1G_Polymyxin             agaaacggtgcgccaattcgttcgtccgtttgatctagcgaagccgccacttctgcgggt 14618
pmxE_B-LR_Polymyxin-E         AGAAACGGTGCGCCAATTCGTTCGTCCATTTGATCTGGCGAAGCCGCCACTTCTGCGTGT 14699
pmxE_KP26_Polymyxin-E         AGAAACGGTGCGCCAATTCGTTCGTCCATTTGATCTGGCGAAGCCGCCACTTCTGCGTGT 14639
pmxE_Polymyxin-P              AGAAACGGTGCGCCAATTCGTTCGTCCGTTTGATCTAGCGAAGCCGCCACTTCTGCGGGT 14699
pmxE_Polymyxin-A              AGAAACGGTGCGCCAATTCGTTCGTCCGTTTGATCTAGCGAAGCCGCCACTTCTGCGGGT 14699
                              ********************* ***** ******** ******************** **

pmxE-D-DaB3-Polymyxin-B       AGGCCTTGCCGAACTGGCGCCGGACCGGCACATTCTAATGTTCGATACGCATCATATCGT 14723
pmxE_1G_Polymyxin             aggccttgccgaactggcgccggaccggcacattctaatgttcgatacgcatcatatcgt 14678
pmxE_B-LR_Polymyxin-E         AGGCCTTGCCGAACTGGCGCCGGACCGGCACATTCTGATGTTCGATACGCATCATATCGT 14759
pmxE_KP26_Polymyxin-E         AGGCCTTGCCGAACTGGCGCCGGACCGGCACATTCTGATGTTCGATACGCATCATATCGT 14699
pmxE_Polymyxin-P              AGGCCTTGCCGAACTGGCGCCGGACCGGCACATTCTAATGTTCGATACGCATCATATCGT 14759
pmxE_Polymyxin-A              AGGCCTTGCCGAACTGGCGCCGGACCGGCACATTCTAATGTTCGATACGCATCATATCGT 14759
                              ************************************ ***********************

pmxE-D-DaB3-Polymyxin-B       ATCTGACGGCGTTTCGATTGACGTACTGATCGAAGAGTTCGTCCGCTTGTACAGCGGGGA 14783
pmxE_1G_Polymyxin             atctgacggcgtttcgattgacgtactgatcgaagagttcgtccgcttgtacagcgggga 14738
pmxE_B-LR_Polymyxin-E         ATCTGACGGCGTTTCGATGGACGTACTGATTGAAGAGTTCGTCCGCTTGTACAGCGGGGA 14819
pmxE_KP26_Polymyxin-E         ATCTGACGGCGTTTCGATGGACGTACTGATTGAAGAGTTCGTCCGCTTGTACAGCGGGGA 14759
pmxE_Polymyxin-P              ATCCGACGGCGTTTCGATTGACGTACTGATCGAAGAGTTCGTCCGCTTGTACAGCGGGGA 14819
pmxE_Polymyxin-A              ATCTGACGGCGTTTCGATTGATGTACTGATCGAAGAGTTGGTCCGCTTGTACAGCGGGGA 14819
                              *** ************** ** ******** ******** ********************
```

```
pmxE-D-DaB3-Polymyxin-B      GCAGTTGGAGCCGCTCCACATTCAGTACAAAGATTATGCGGTATGGCAGCAATCGGACGA 14843
pmxE_1G_Polymyxin            gcagttggagccgctccgcattcagtacaaagattatgcggtatggcagcaatcggacga 14798
pmxE_B-LR_Polymyxin-E        GCCGTTGGAGCCGCTACGCATTCAGTACAAAGATTATGCGGTATGGCAGCAATCGGACGA 14879
pmxE_KP26_Polymyxin-E        GCCGTTGGAGCCGCTACGCATTCAGTACAAAGATTATGCGGTATGGCAGCAATCGGACGA 14819
pmxE_Polymyxin-P             ACAGTTGGAGCCGCTCCGCATTCAATACAAAGATTATGCGGTATGGCAGCAATCGGACGA 14879
pmxE_Polymyxin-A             GCAGTTGGAGCCGCTCCGCATTCAGTACAAAGATTATGCGGTATGGCAGCAATCGGACGA 14879
                              * ************ * ****** ***********************************

pmxE-D-DaB3-Polymyxin-B      GCAGAAAGCTCAGCTTGCCAAGCAGGAAGCCTACTGGCTTGACATGTTCCGCGGAGAGCT 14903
pmxE_1G_Polymyxin            gcagaaagctcagcttgccaagcaggaagcctactggctcgacatgttccgcggagagct 14858
pmxE_B-LR_Polymyxin-E        GCAGAAAGCTCAGCTTGCCAAGCAGGAAGCCTACTGGCTCGACATGTTCCGCGGAGAACT 14939
pmxE_KP26_Polymyxin-E        GCAGAAAGCTCAGCTTGCCAAGCAGGAAGCCTACTGGCTCGACATGTTCCGCGGAGAACT 14879
pmxE_Polymyxin-P             GCAGAAAGCTCAGCTTGCCAAGCAGGAAGCCTACTGGCTCGACATGTTCCGCGGAGAGCT 14939
pmxE_Polymyxin-A             GCAGAAAGCTCAGCTTGCCAAGCAGGAAGCCTACTGGCTCGACATGTTCCGCGGAGAGCT 14939
                             *************************************** ***************** **

pmxE-D-DaB3-Polymyxin-B      GCCGGTTTTGGAATTGCCGACGGACTATCCGCGCCCGGCTATGCAGAGCTACGAGGGCCG 14963
pmxE_1G_Polymyxin            gccggttttggaattgccgacggactatccgcgcccggctatgcagagctacgagggccg 14918
pmxE_B-LR_Polymyxin-E        GCCGGTTTTGGAATTGCCAACGGACTACCCACGCCCAGCTATGCAGAGCTACGAGGGTCG 14999
pmxE_KP26_Polymyxin-E        GCCGGTTTTGGAATTGCCAACGGACTACCCACGCCCAGCTATGCAGAGCTACGAGGGTCG 14939
pmxE_Polymyxin-P             GCCGGTTTTGGAATTGCCGACGGACTATCCGCGCCCGTCTATGCAGAGCTACGAGGGCCG 14999
pmxE_Polymyxin-A             GCCGGTTTTGGAATTGCCGACGGACTATCCGCGCCCGGCTATGCAGAGCTACGAGGGCCG 14999
                             ****************** ******** ** *****  ******************* **

pmxE-D-DaB3-Polymyxin-B      CACGCTGCAATTGTTTATGGATAGCGAGAAAAGAGAGGGTCTGAAACGGCTTGCAGCCGA 15023
pmxE_1G_Polymyxin            cacgctgcaattgtttatggatagcgagaaaagcgagggtctgaaacggcttgcagccga 14978
pmxE_B-LR_Polymyxin-E        CACACTGCAATTGTTTATGAATAGGGAGAAAAGCGAGGGTCTGAAACGGCTTGCAGCCGA 15059
pmxE_KP26_Polymyxin-E        CACACTGCAATTGTTTATGAATAGGGAGAAAAGCGAGGGTCTGAAACGGCTTGCAGCCGA 14999
pmxE_Polymyxin-P             CACGCTGCAATTGTTTATGGATAGCAAGAAAAGCGAGGGTCTGAAACGGCTTGCAGCCGA 15059
pmxE_Polymyxin-A             CACGCTGCAATTGTTTATGCATAGCGAGAAAAGCGAGGGTCTGAAACGGCTTGCAGCCGA 15059
                             *** *************** ****  ******* **************************

pmxE-D-DaB3-Polymyxin-B      GAACGGCGCAACGCTTTACATGGTTCTGCTTGCTGGTTATACAATATTATTGCATAAATA 15083
pmxE_1G_Polymyxin            gaacggcgcaacgctttacatggttctacttgctagttatacaattttattgcataaata 15038
pmxE_B-LR_Polymyxin-E        GAACGGCGCAACGCTTTACATGGTTCTGCTTGCTGGTTATACAATATTATTGCATAAATA 15119
pmxE_KP26_Polymyxin-E        GAACGGCGCAACGCTTTACATGGTTCTGCTTGCTGGTTATACAATATTATTGCATAAATA 15059
pmxE_Polymyxin-P             GAACGGCGCAACGCTTTACATGGTTCTGCTTGCTGGTTATACAATATTATTGCATAAGTA 15119
pmxE_Polymyxin-A             GAACGGCGCAACGCTTTACATGGTTCTGCTTGCTGGTTATACAATATTATTGCATAAATA 15119
                             *************************** ****** ********** *********** **

pmxE-D-DaB3-Polymyxin-B      TACAGGGCAAGAAGACGTGGTGGTCGGTACGCCGATTGCGGGAAGAAATCACAGCGACGT 15143
pmxE_1G_Polymyxin            tacagggcaagaagacgtggtggtcggtacgccgattgcgggaagaaatcatagcgacgt 15098
pmxE_B-LR_Polymyxin-E        TACTAGTCAAGAAGACGTAGTGGTCGGTACGCCGATTGCGGGAAGAAATCACAGTGACGT 15179
```

```
pmxE_KP26_Polymyxin-E        TACTAGTCAAGAAGACGTAGTGGTCGGTACGCCGATTGCGGGAAGAAATCACAGTGACGT 15119
pmxE_Polymyxin-P             TACAGGGCAAGAAGACGTGGTGGTCGGTACGCCGATTGCGGGAAGAAATCACAGCGACGT 15179
pmxE_Polymyxin-A             TACAGGACAAGAAGACGTGGTGGTCGGTACGCCGATTGCGGGAAGAAATCACAGCGATGT 15179
                             ***  * *********** ******************************** ** ** **

pmxE-D-DaB3-Polymyxin-B      TCAGCCGCTGATCGGGATGTTCGTCAATACCCTGGCCATCCGCAGTTATCCGGCTGCCGG 15203
pmxE_1G_Polymyxin            tcagccgctgatcgggatgttcgtcaataccctggccatccgcagttatccggctgccgg 15158
pmxE_B-LR_Polymyxin-E        TCAGCCGCTGATCGGAATGTTCGTCAATACTCTGGCCATCCGCAGTTATCCGACTGCGGG 15239
pmxE_KP26_Polymyxin-E        TCAGCCGCTGATCGGAATGTTCGTCAATACTCTGGCCATCCGCAGTTATCCGACTGCGGG 15179
pmxE_Polymyxin-P             TCAGCCGCTGATCGGGATGTTCGTCAATACCCTGGCCATCCGCAGTTATCCGGCTGCCGG 15239
pmxE_Polymyxin-A             TCAGCCGCTGATCGGGATGTTCGTCAATACCCTGGCCATCCGCAGTTATCCGGCTGCCGA 15239
                             *************** ************** ********************* **** *

pmxE-D-DaB3-Polymyxin-B      TAAGACGTTCCTTGACTACTTGAAGGAAATCAAGGAGACGACGCTGGGCGCTTTTGAACA 15263
pmxE_1G_Polymyxin            taagacgttccttggctacttgaaggaaatcaaggagacgacgctgggcgcttttgaaca 15218
pmxE_B-LR_Polymyxin-E        TAAGACGTTCCTTGACTACTTGAAGGAAATCAAGGAGACGACGCTGGGTGCTTTTGAACA 15299
pmxE_KP26_Polymyxin-E        TAAGACGTTCCTTGACTACTTGAAGGAAATCAAGGAGACGACGCTGGGTGCTTTTGAACA 15239
pmxE_Polymyxin-P             TAAGACGTTCCTTGACTACTTGAAGGAAATCAAGGAGACGACGCTGGGCGCTTTTGAACA 15299
pmxE_Polymyxin-A             TAAGACGTTCCTTGAGTACTTGAAGGAAATCAAGGAGACGACGCTGGGCGCTTTTGAACA 15299
                             **************  ******************************** ***********

pmxE-D-DaB3-Polymyxin-B      TCAGAATTATCCGTTTGAGGAACTGGTGGATAAGGTGAACGTGGCTCGCGATTTAAGCCG 15323
pmxE_1G_Polymyxin            tcagaattatccgtttgaggaactggtggataaggtgaacgtggctcgtgatttacgccg 15278
pmxE_B-LR_Polymyxin-E        TCAGAATTATCCGTTTGAGGAACTGGTGGATAAGGTGAACGTAGCTCGTGATTTAAGCCG 15359
pmxE_KP26_Polymyxin-E        TCAGAATTATCCGTTTGAGGAACTGGTGGATAAGGTGAACGTAGCTCGTGATTTAAGCCG 15299
pmxE_Polymyxin-P             TCAGAATTATCCGTTTGAGGAACTGGTGGATAAAGTGAACGTGGCTCGCGATTTACGCCG 15359
pmxE_Polymyxin-A             TCAGAATTATCCGTTTGAGGAACTGGTGGATAAGGTGAACGTGGCTCGTGATTTACGCCG 15359
                             ********************************* ******** ***** ****** ****

pmxE-D-DaB3-Polymyxin-B      CAATCCGCTATTCGATACGATGTTTGCTTTGCAAAATACAGAGAATTTGGAAATCCAGCT 15383
pmxE_1G_Polymyxin            caatccgctgttcgatacgatgtttgctttgcagaatacagagaatttggaaatccagct 15338
pmxE_B-LR_Polymyxin-E        CAATCCGCTGTTCGATACGATGTTTGCTTTGCAGAATACAGAGAATTTGGAAATCCAGCT 15419
pmxE_KP26_Polymyxin-E        CAATCCGCTGTTCGATACGATGTTTGCTTTGCAGAATACAGAGAATTTGGAAATCCAGCT 15359
pmxE_Polymyxin-P             CAATCCGCTATTCGATACAATGTTTGCTTTGCAGAATACAGAGAATTTGGAAATCCAGCT 15419
pmxE_Polymyxin-A             CAATCCGCTGTTCGATACGATGTTTGCTTTGCAGAATACAGAGAATTTGGAAATCCAGCT 15419
                             ********* ******** ************** **************************

pmxE-D-DaB3-Polymyxin-B      TCCCGGACTCCATTTGTCGACGTATGCCAGCGAAGAAATTGTTTCTAAATTCGATCTCAG 15443
pmxE_1G_Polymyxin            tcccggactccatttgtcgacgtatgccagcgaagaaattgtttctaaattcgatctcag 15398
pmxE_B-LR_Polymyxin-E        TCCCGGACTCCATTTGTCGACGTATGCCAGCGAAGAAATTGTTTCTAAATTCGATCTCAG 15479
pmxE_KP26_Polymyxin-E        TCCCGGACTCCATTTGTCGACGTATGCCAGCGAAGAAATTGTTTCTAAATTCGATCTCAG 15419
pmxE_Polymyxin-P             TCCCGGACTCCATTTGTCGACGTATGCCAGCGAAGAAATTGTTTCTAAATTCGATCTCAG 15479
pmxE_Polymyxin-A             TCCCGGACTCCATTTGTCGACGTATGCCAGCGAAGAAATTGTTTCTAAATTCGATCTCAG 15479
                             ************************************************************
```

```
pmxE-D-DaB3-Polymyxin-B      CTTGGACGTCACGGAGATCGAGGAAGGCTTGGAATATCTGTTTGAATACGCCACTGCTCT 15503
pmxE_1G_Polymyxin            cttggacgtcacggagatcgaggaaggcttggaatatctgtttgaatacgccactgctct 15458
pmxE_B-LR_Polymyxin-E        CTTGGACGTCACGGAGATCGAGGAAGGCTTGGAATATCTGTTTGAATACGCCACTGCTCT 15539
pmxE_KP26_Polymyxin-E        CTTGGACGTCACGGAGATCGAGGAAGGCTTGGAATATCTGTTTGAATACGCCACTGCTCT 15479
pmxE_Polymyxin-P             CTTGGACGTCACGGAGATCGAGGAAGGCTTGGAATATCTGTTTGAATATGCTACTGCTCT 15539
pmxE_Polymyxin-A             CTTGGACGTCACGGAGATCGAGGAAGGCTTGGAATATCTGTTTGAATACGCCACTGCTCT 15539
                             ************************************************ ** ********

pmxE-D-DaB3-Polymyxin-B      TTATAAAGCCGAAACGGTGGAGAAATTGGCCGCTCACTACTTGCAGCTGCTTGAATCCAT 15563
pmxE_1G_Polymyxin            ttataaaaccgaaacggtggagaaattggccgctcactacttgcagctgcttgaatccat 15518
pmxE_B-LR_Polymyxin-E        TTATAAAACCGAAACGGTGGAGAAATTGGCCGCTCACTACTTGCAGCTGCTTGAATCTAT 15599
pmxE_KP26_Polymyxin-E        TTATAAAACCGAAACGGTGGAGAAATTGGCCGCTCACTACTTGCAGCTGCTTGAATCTAT 15539
pmxE_Polymyxin-P             TTATAAAACCGAAACGGTGGAGAAATTGGCCGCTCACTACTTGCAGCTGCTTGAATCCAT 15599
pmxE_Polymyxin-A             TTATAAAACCGAAACGGTGGAGAAATTGGCCGCTCACTACTTGCAGCTGCTTGAATCCAT 15599
                             ******* ************************************************* **

pmxE-D-DaB3-Polymyxin-B      TCTCCGCAACCCGTCGGCGACTATTGCCGAGCTGGGCATTTTGACACCGGCGGAAAAAGA 15623
pmxE_1G_Polymyxin            tctccgcaacccatcggcgactattgccgagctgggcattttgacatcggcggaaaaaga 15578
pmxE_B-LR_Polymyxin-E        TCTCTGCAACCCTTCTGCGACTATTGCCGAGCTGGGCATTTTGACACCAGCGGAAAAAGA 15659
pmxE_KP26_Polymyxin-E        TCTCTGCAACCCTTCTGCGACTATTGCCGAGCTGGGCATTTTGACACCAGCGGAAAAAGA 15599
pmxE_Polymyxin-P             TCTCCGCAACCCGACGGCGACTATTGCCGAGCTGGGCATTTTGACACCGGCGGAAAAAGA 15659
pmxE_Polymyxin-A             TCTCCGCAACCCATCGGCGACTATTGCCGAGCTGGGCATTTTGACACCGGCGGAAAAAGA 15659
                             **** *******  * ****************************** * ***********

pmxE-D-DaB3-Polymyxin-B      GCAAATTCTCGGTGCGTTTAACCCGGCGCAGCCGGAAGCGGCTCCTGCGTCGGCGTTCCA 15683
pmxE_1G_Polymyxin            gcaaattctcggcgcgtttaacccggcgcagccggaggcggctcctgcggccgcgttcca 15638
pmxE_B-LR_Polymyxin-E        ACAAATTCTCGGCGCGTTCAACCCGGCGCAGCCGGAAGCGGCTCCTGCGGCGGCGTTCCA 15719
pmxE_KP26_Polymyxin-E        ACAAATTCTCGGCGCGTTCAACCCGGCGCAGCCGGAAGCGGCTCCTGCGGCGGCGTTCCA 15659
pmxE_Polymyxin-P             GCAGATTCTCGGCGCGTTTAACCCGGCGCAGCCGGAAGCGACTCCTGCGGCCGCGTTCCA 15719
pmxE_Polymyxin-A             GCAAATTCTCGGCGCGTTTAACCCGGCGCAGCCGGAAGCGGCTCCTGCGACCGCGTTCCA 15719
                              ** ******** ***** ***************** *** ******** * ********

pmxE-D-DaB3-Polymyxin-B      CCGGCTGTTTGAGGAACAGGCGGAGCGCACGCCGGAAGCGACAGCTGTCGTGTACGAGAA 15743
pmxE_1G_Polymyxin            ccggctgtttgaggaacaggcggagcgcacgccggaagaggcggccgtcgtgtacgagaa 15698
pmxE_B-LR_Polymyxin-E        CCGGCTGTTCGAGGAACAGGCGGAGCGCACGCCGGAAGCGGAGGCTGTCGTGTACGAGAA 15779
pmxE_KP26_Polymyxin-E        CCGGCTGTTCGAGGAACAGGCGGAGCGCACGCCGGAAGCGGAGGCTGTCGTGTACGAGAA 15719
pmxE_Polymyxin-P             CCGGCTGTTCGAGGAACAGGTGGAGCGCACGCCGGAAGAGGCAGCCGTCGTGTACGAGAA 15779
pmxE_Polymyxin-A             CCGGCTGTTTGAGGAACAGGTGGAGCGCACGCCGGAAGAGGCGGCCGTCGTGTACGAGAA 15779
                             ********* ********** ***************** *   ** **************

pmxE-D-DaB3-Polymyxin-B      CGACCGGCTGACGTATGCGGAGCTGAACGAGCGGGCGAACCGCTTGGCGGCTACGCTGCG 15803
pmxE_1G_Polymyxin            cgaccggctgacgtatgcggagctgaacgagcgggcgaaccgcttggcggctacgctgcg 15758
pmxE_B-LR_Polymyxin-E        CGACCGGCTGATTTATGCGGAGCTGAACGAGCGGGCGAACCGCTTGGCGGCTACGCTGCG 15839
```

```
pmxE_KP26_Polymyxin-E        CGACCGGCTGATTTATGCGGAGCTGAACGAGCGGGCGAACCGCTTGGCGGCTACGCTGCG 15779
pmxE_Polymyxin-P             CGACCGGCTGACGTATGCGGAGCTGAACGAGCGGGCGAACCGCTTGGCGGCTACGCTGCG 15839
pmxE_Polymyxin-A             TGACCGGCTGACGTATGCGGAGCTGAACAAGCGGGCGAACCGCTTGGCGGCTACGCTGCG 15839
                              **********  *************** *******************************

pmxE-D-DaB3-Polymyxin-B      CGCAAGCGGCATCGGCCGGGAGACGATCGTCGGCATTCTCGCCGAGCGTTCGGTGGACTT 15863
pmxE_1G_Polymyxin            cgcaagcggcatcggccgggagtcgatcgtcggcattctcgccgagcgttcggtggattt 15818
pmxE_B-LR_Polymyxin-E        CGCAAGCGGCATCGGCCGGGAGTCGATCGTCGGCATTCTCGCCGAGCGTTCGGTGGACTT 15899
pmxE_KP26_Polymyxin-E        CGCAAGCGGCATCGGCCGGGAGTCGATCGTCGGCATTCTCGCCGAGCGTTCGGTGGACTT 15839
pmxE_Polymyxin-P             CGCAAGCGGCATCGGCCGGGAGACGATCGTCGGCATTCTCGCCGAGCGTTCGGTGGACTT 15899
pmxE_Polymyxin-A             CGCAGGCGGCATCGGCCGGGAGACGATCGTCGGCATTCTTGCCGAGCGTTCGGTGGACTT 15899
                             **** ***************** **************** ***************** **

pmxE-D-DaB3-Polymyxin-B      GCTGGTGGCCGTGCTGGCCGTCTGGAAAGCGGGCGGGGCGTATGTGCCGCTCGACCCGGA 15923
pmxE_1G_Polymyxin            gctggtggccgtgctggccgtctggaaagcgggcggggcgtatgtgccgctcgacccgga 15878
pmxE_B-LR_Polymyxin-E        GCTGGTGGCCGTGCTGGCCGTCTGGAAAGCGGGCGGGGCGTATGTGCCGCTCGACCCGGA 15959
pmxE_KP26_Polymyxin-E        GCTGGTGGCCGTGCTGGCCGTCTGGAAAGCGGGCGGGGCGTATGTGCCGCTCGACCCGGA 15899
pmxE_Polymyxin-P             GCTGGTGGCCGTGCTGGCCGTCTGGAAAGCGGGCGGGGCGTATGTGCCGCTGGACCCGGA 15959
pmxE_Polymyxin-A             GCTGGTGGCCGTGCTGGCTATCTGGAAAGCGGGCGGGGCGTATGTGCCGCTCGACCCGGA 15959
                             ******************  ******************************* ********

pmxE-D-DaB3-Polymyxin-B      TTACCCGGCGGAGCGCGTGCGGTTCATGCTCGAAGTCAGCGGAGCGAAGGTGCTGTTGAC 15983
pmxE_1G_Polymyxin            ttatccggcagaccgcgtgcggttcatgcttgaagacagcggagcgaaggtgctgctgac 15938
pmxE_B-LR_Polymyxin-E        TTATCCGGCGGACCGCGTGCGGTTCATGCTTGAAGACAGCGGAGCGAAGGTTCTGCTGAC 16019
pmxE_KP26_Polymyxin-E        TTATCCGGCGGACCGCGTGCGGTTCATGCTTGAAGACAGCGGAGCGAAGGTTCTGCTGAC 15959
pmxE_Polymyxin-P             TTATCCGGCGGAGCGCGTGCGGTTCATGCTTGAAGACAGCGGAGCGAAGGTGCTGCTGAC 16019
pmxE_Polymyxin-A             TTATCCGGCAGACCGCGTGCGGTTCATGCTTGAAGACAGCGGAGCGAAGGTGCTGCTGAC 16019
                             *** ***** ** ***************** **** *************** *** ****

pmxE-D-DaB3-Polymyxin-B      GCAAACGCCGCTGCGAGAGCGCGCCGAAGCCTGGCTCGGCGAAGAGGAGCTGGCGCTGGC 16043
pmxE_1G_Polymyxin            gcaaacgccgctgcgagagcgtgccgaagcctggctcggcgaagaggagctggcgctggc 15998
pmxE_B-LR_Polymyxin-E        GCAAAAGGTGCTGCGAGAGCGCGCCGAAGCCTGGCTCGGCGAAGAGGAGCTGACGCTGGC 16079
pmxE_KP26_Polymyxin-E        GCAAAAGGTGCTGCGAGAGCGCGCCGAAGCCTGGCTCGGCGAAGAGGAGCTGACGCTGGC 16019
pmxE_Polymyxin-P             GCAAACGCCGCTGCGAGAGCGCGCCGAAGCCTGGCTCGGCGAAGAGGAGCTGGCGCTGGC 16079
pmxE_Polymyxin-A             GCAAACGCCGCTGCGAGAGCGTGCCGAAGCCTGGCTCGGCGAAGAGGAGCTGGCGCTGGC 16079
                             ***** *  ************ ****************************** *******

pmxE-D-DaB3-Polymyxin-B      GGCGGTGCTGTATCTCGACGACGAAACTTCGTACAGCGAGGAGCGGACGAATGC------ 16097
pmxE_1G_Polymyxin            ggcggtgctgtacctcgacgacgaagcgtcgtacagcgaggagcgggcgaatgc------ 16052
pmxE_B-LR_Polymyxin-E        AGCGGTGCTGTACCTCGACGACGAAGCGTCGTACAGCGAGGTGCGGGCGAATGCGCCGAT 16139
pmxE_KP26_Polymyxin-E        AGCGGTGCTGTACCTCGACGACGAAGCGTCGTACAGCGAGGTGCGGGCGAATGCGCCGAT 16079
pmxE_Polymyxin-P             CACGGTGCTGTACCTGGATGACGAAGCGTCGTACAGCGAGGAGCGGGCGAATGCGCCGAT 16139
pmxE_Polymyxin-A             GGCGGTGCTGTACCTCGATGACGAAACGTCGTACAGCGAGGAGCGGGCGAATGCGCCGAT 16139
                               ********** ** ** ****** * ************* **** *******
```

```
pmxE-D-DaB3-Polymyxin-B      ------------------------------------------------------------ 16097
pmxE_1G_Polymyxin            ------------------------------------------------------------ 16052
pmxE_B-LR_Polymyxin-E        TGGCTCCGGCATGGTCTCCGGCAAGCTGATGGATGCTGTGAATGACGGCGATGGGACCCA 16199
pmxE_KP26_Polymyxin-E        TGGCTCCGGCATGGTCTCCGGCAAGCTGATGGATGCTGTGAATGACGGCGATGGGACCCA 16139
pmxE_Polymyxin-P             TGGCTCGGGCAGGGTCTCCGGCAAGCTGACGGATGCTGTGGACGACGGCGATGAGAGCCA 16199
pmxE_Polymyxin-A             TGGCTCCGGCATGGTCTCCGGCAAGCTGACGGATGCTGTGAACGACGGCGATGAGAGCCA 16199


pmxE-D-DaB3-Polymyxin-B      ---------------CGACAGCTTCCATGAAGCCCGTCCAGAGGACCTGGCGTACGTGAT 16142
pmxE_1G_Polymyxin            ---------------cgacagcttccatgaagcccgtccagaggatctggcgtacgtgat 16097
pmxE_B-LR_Polymyxin-E        TCCGAATGTTGACATGGGCAGCTTCCATGAAGCCCGTCCGGAGGATCTGGCGTACGTGAT 16259
pmxE_KP26_Polymyxin-E        TCCGAATGTTGACATGGGCAGCTTCCATGAAGCCCGTCCGGAGGATCTGGCGTACGTGAT 16199
pmxE_Polymyxin-P             TCAGAATATTGGCACCGACAGCTTCCATGAAGCCCGTCCGGAGGAACTGGCGTACGTGAT 16259
pmxE_Polymyxin-A             TCAGAATGTTGGCACCGACAGCTTCCATGAAGCCCGTCCGGAGAACCTGGCGTACGTGAT 16259
                                             * ********************* *** * **************

pmxE-D-DaB3-Polymyxin-B      CTACACGTCGGGAACGACGGGCAAGCCGAAGGGCGTGATGATCGAGCACCGCAGCCTGGT 16202
pmxE_1G_Polymyxin            ctatacgtcgggaacgacgggcaagccgaagggcgtgatgatcgagcaccgcagcctggt 16157
pmxE_B-LR_Polymyxin-E        CTATACGTCGGGAACGACGGGCAAGCCGAAGGGCGTGATGATCGAGCACCGCAGCCTGGT 16319
pmxE_KP26_Polymyxin-E        CTATACGTCGGGAACGACGGGCAAGCCGAAGGGCGTGATGATCGAGCACCGCAGCCTGGT 16259
pmxE_Polymyxin-P             CTATACGTCGGGAACGACGGGCAAGCCGAAGGGCGTGATGATCGAGCACCGCAGCCTGGT 16319
pmxE_Polymyxin-A             CTATACGTCGGGAACGACGGGCAAGCCGAAGGGCGTGATGATCGAGCACCGCAGCCTGGT 16319
                             *** ********************************************************

pmxE-D-DaB3-Polymyxin-B      GAATACGGCGGCGGGCTACCGGCGGGAATACCGGTTGGATCAGTTCCCGGTGCGGCTGCT 16262
pmxE_1G_Polymyxin            gaacacggcggcgggttaccggcgggaataccggttggatcagttcccggtgcggctgct 16217
pmxE_B-LR_Polymyxin-E        GAACACGGCAGCGGGCTACCGGCGGGAATACCGGTTGGATCAGTTCCCGGTGCGGCTGCT 16379
pmxE_KP26_Polymyxin-E        GAACACGGCAGCGGGCTACCGGCGGGAATACCGGTTGGATCAGTTCCCGGTGCGGCTGCT 16319
pmxE_Polymyxin-P             GAACACAGCGGCGGGCTACCGGCGGGAATACCGGTTGGATCAGTTCCCGGTGCGGCTGCT 16379
pmxE_Polymyxin-A             GAACACGGCGGTGGGCTACCGGCGGGAATACCGGTTGGATCAGTTCCCGGTGCGGTTGCT 16379
                             *** ** ** * *** *************************************** ****

pmxE-D-DaB3-Polymyxin-B      GCAGCTCGCCAGCTTCTCGTTCGACGTGTTCGTGGGAGATATCGCGCGGACGCTGTACAA 16322
pmxE_1G_Polymyxin            gcagctcgcaagcttctcgttcgacgtgttcgtgggagatatcgcgcggacgctgtacaa 16277
pmxE_B-LR_Polymyxin-E        GCAGCTCGCAAGCTTCTCGTTCGACGTATTCGTGGGCGATATCGCGCGGACGCTGTATAA 16439
pmxE_KP26_Polymyxin-E        GCAGCTCGCAAGCTTCTCGTTCGACGTATTCGTGGGCGATATCGCGCGGACGCTGTATAA 16379
pmxE_Polymyxin-P             GCAGCTCGCAAGCTTCTCATTCGACGTGTTCGTGGGAGATATCGCGCGGACGCTGTACAA 16439
pmxE_Polymyxin-A             GCAGCTCGCAAGCTTCTCGTTCGACGTGTTCGTGGGAGATATCGCGCGGACGCTGTACAA 16439
                             ********* ******** ******** ******** ******************** **

pmxE-D-DaB3-Polymyxin-B      CGGAGGCACGATGGTGATTGTGCCGAAGGACGACCGGATCGATCCGTCTCGTCTGCACCA 16382
pmxE_1G_Polymyxin            cggaggcacgatggtgattgtgccgaaggacgaccggatcgatccgtctcgtctgcacca 16337
pmxE_B-LR_Polymyxin-E        CGGAGGCACGATGGTGATTGTGACGAAGGACGACCGGATCGATCCGTCTCGTCTGCACCA 16499
```

```
pmxE_KP26_Polymyxin-E        CGGAGGCACGATGGTGATTGTGACGAAGGACGACCGGATCGATCCGTCTCGTCTGCACCA 16439
pmxE_Polymyxin-P             CGGAGGCACGATGGTGATCGTGCCGAAGGACGACCGGATTGATCCGTCTCGTCTGCACCA 16499
pmxE_Polymyxin-A             CGGAGGTACGATGGTGATTGTGCCGAAGGACGATCGGATTGATCCGTCTCGTCTGCACCA 16499
                             ****** *********** *** ********** ***** ********************

pmxE-D-DaB3-Polymyxin-B      CTGGATTGAGCGGGAGCGGGTCACCATCTTCGAATCGACGCCGGCGCTGATCGTGCCGTT 16442
pmxE_1G_Polymyxin            ctggatggagcgggagcggatcaccatcttcgaatcgacgccggcgctgatcgtgccgtt 16397
pmxE_B-LR_Polymyxin-E        CTGGATGGAGCGGGAGCGGGTCACCATCTTCGAATCGACGCCGGCGCTAATCGTGCCGTT 16559
pmxE_KP26_Polymyxin-E        CTGGATGGAGCGGGAGCGGGTCACCATCTTCGAATCGACGCCGGCGCTAATCGTGCCGTT 16499
pmxE_Polymyxin-P             CTGGATTGAGCGGGAGCAGGTCACCATCTTCGAATCGACGCCGGCGCTGATCGTGCCGTT 16559
pmxE_Polymyxin-A             CTGGATGGAGCGGGAGCGGGTCACCATCTTCGAATCGACGCCGGCGCTGATCGTGCCGTT 16559
                             ****** ********** * **************************** ***********

pmxE-D-DaB3-Polymyxin-B      CCTGGAGTACGTGCACGAGCAGCAACTGGATATGAGCTGGATGGAGCTGTTGATCACGAG 16502
pmxE_1G_Polymyxin            cctggagtacgtgcacgagcagcggctggatatgagctggatggagctgttgatcacgag 16457
pmxE_B-LR_Polymyxin-E        CCTGGAGTACGTGCACGAGCAGGGGCTGGATATGAGCTGGATGGAGCTGTTGATCACGAG 16619
pmxE_KP26_Polymyxin-E        CCTGGAGTACGTGCACGAGCAGGGGCTGGATATGAGCTGGATGGAGCTGTTGATCACGAG 16559
pmxE_Polymyxin-P             CCTGGAGTACGTGCACGAGCAGGGGCTGGATATGAGCTGGATGGAGCTGTTGATCACGAG 16619
pmxE_Polymyxin-A             CCTGGAGTACGTGCACGATCAGCAGCTGGATATGAGCTGGATGGAGCTGTTGATCACGAG 16619
                             ****************** ***   ***********************************

pmxE-D-DaB3-Polymyxin-B      CTCGGACAGCTGCAGCGTGGCGGATTACCGGACCTTGCAGGAACGCTTCGGCTCGTTGTT 16562
pmxE_1G_Polymyxin            ctcggacagctgcagcgtggcggattaccggaccttgcaggaacgcttcggctcgttgtt 16517
pmxE_B-LR_Polymyxin-E        TTCGGACAGCTGCAGCGTGGCGGATTACCGGACCTTGCAGGAACGCTTCGGCTCGTTGTT 16679
pmxE_KP26_Polymyxin-E        TTCGGACAGCTGCAGCGTGGCGGATTACCGGACCTTGCAGGAACGCTTCGGCTCGTTGTT 16619
pmxE_Polymyxin-P             CTCGGACAGCTGCAGCGTGGCGGATTACCGGACCTTGCAGGAACGCTTCGGCTCGTTGTT 16679
pmxE_Polymyxin-A             CTCGGACAGCTGCAGCGTGGCGGATTACCGGACCTTGCAGGAACGCTTCGGCTCGTTGTT 16679
                              ***********************************************************

pmxE-D-DaB3-Polymyxin-B      CCGGATCATCAACGCATACGGCGTGACGGAAGCGGCGATCGACTCAAGCTTCTACGACGA 16622
pmxE_1G_Polymyxin            ccggatcatcaacgcctacggcgtgacggaagcggcgatcgactcaagcttctacgacga 16577
pmxE_B-LR_Polymyxin-E        CCGGATCATCAATGCTTATGGCGTGACGGAAGCGGCGATCGACTCCAGCTTCTACGACGA 16739
pmxE_KP26_Polymyxin-E        CCGGATCATCAATGCTTATGGCGTGACGGAAGCGGCGATCGACTCCAGCTTCTACGACGA 16679
pmxE_Polymyxin-P             CCGGATCATCAACGCATACGGCGTGACGGAAGCGGCGATCGACTCCAGCTTCTACGACGA 16739
pmxE_Polymyxin-A             CCGAATCATCAACGCCTACGGCGTGACGGAAGCGGCGATCGACTCCAGCTTCTACGACGA 16739
                             *** ******** ** ** ************************** **************

pmxE-D-DaB3-Polymyxin-B      GGAGCTGGCGAAGCTGCCGCAGACAGGCAATGTGCCGATCGGCAAAGCGTGGCTGAATGC 16682
pmxE_1G_Polymyxin            gccgctgacgaagctgccgcagacaggccatgtgccgatcggcaaagcgtggctgaatgc 16637
pmxE_B-LR_Polymyxin-E        GGAGCTGACGAAGCTGCCACAGACAGGCCATGTGCCGATCGGCAAAGCGTGGCTGAATGC 16799
pmxE_KP26_Polymyxin-E        GGAGCTGACGAAGCTGCCACAGACAGGCCATGTGCCGATCGGCAAAGCGTGGCTGAATGC 16739
pmxE_Polymyxin-P             GGAGCTGACGAAGCTACCGCAGACAGGTAATGTGCCGATCGGTAAAGCGTGGCTGAATGC 16799
pmxE_Polymyxin-A             GGAGCTGGCGAAGCTGCCGCAGACAGGCCATGTGCCGATCGGCAAAGCGTGGCTGAATGC 16799
                             *  **** ******* ** ********  ************* *****************
```

```
pmxE-D-DaB3-Polymyxin-B      GAAATTCTACATCGTGGACGCGCATCTGAACCCGGTGCCGGTCGGGGTGCTGGGCGAGCT 16742
pmxE_1G_Polymyxin            gaaattttacatcgtggacgcgcatctgaacccggtgccggtcggggtgctgggcgagct 16697
pmxE_B-LR_Polymyxin-E        GAAATTCTACATCGTGGACGCGCATCTGAACCCGGTGCCGGTCGGGGTGCTGGGCGAGCT 16859
pmxE_KP26_Polymyxin-E        GAAATTCTACATCGTGGACGCGCATCTGAACCCGGTGCCGGTCGGGGTGCTGGGCGAGCT 16799
pmxE_Polymyxin-P             GAAATTCTACATCGTGGATGCGCATCTGAATCCGGTGCCGGTCGGGGTGCTGGGCGAGCT 16859
pmxE_Polymyxin-A             GAAATTCTACATCGTGGACGCGCATCTGAACCCGGTGCCAGTCGGGGTGATGGGCGAGCT 16859
                             ****** *********** *********** ******** ********* **********

pmxE-D-DaB3-Polymyxin-B      GGTCATCGGCGGAGTCGGAGTGGCGCGCGGGTACTTGAACCGTCCGGAGCTGACAGAAGA 16802
pmxE_1G_Polymyxin            ggttatcggcggagtcggggtagcgcgcgggtacttgaaccgtccggagctgacagaaga 16757
pmxE_B-LR_Polymyxin-E        GGTCATCGGCGGAGTCGGAGTGGCGCGAGGGTACTTGAACCGTCCGGAGCTGACGGAAGA 16919
pmxE_KP26_Polymyxin-E        GGTCATCGGCGGAGTCGGAGTGGCGCGAGGGTACTTGAACCGTCCGGAGCTGACGGAAGA 16859
pmxE_Polymyxin-P             GGTCATCGGCGGAGTCGGAGTGGCGCGCGGGTACTTGAACCGTCCGAAGCTGACGGAAGA 16919
pmxE_Polymyxin-A             GGTCATCGGCGGAGTCGGGGTAGCGCGCGGGTACTTGAACCGTCCGGAACTGACGGAAGA 16919
                             *** ************** ** ***** ***************** * ***** *****

pmxE-D-DaB3-Polymyxin-B      GAAGTTCGTAGACAGCCCGTTCGCCGCGGGCGAGCGGCTGTACCGCACGGGAGACTTGGC 16862
pmxE_1G_Polymyxin            gaagttcgtagacagcccgttcgccgcgggcgagcggctgtaccgcacgggagacttggc 16817
pmxE_B-LR_Polymyxin-E        GAAGTTCGTAGACAGTCCGTTCGCCGCGGGCGAGCGACTGTACCGCACGGGAGACTTGGC 16979
pmxE_KP26_Polymyxin-E        GAAGTTCGTAGACAGTCCGTTCGCCGCGGGCGAGCGACTGTACCGCACGGGAGACTTGGC 16919
pmxE_Polymyxin-P             AAAGTTCGTAGACAGTCCGTTCGCCGCGGGCGAGCGGCTGTACCGCACGGGAGACTTGGC 16979
pmxE_Polymyxin-A             GAAGTTCGTAGACAGTCCGTTCGCCGCGGGCGAGCGGCTGTACCGCACGGGAGACTTGGC 16979
                              ************** ******************** ***********************

pmxE-D-DaB3-Polymyxin-B      GCGGTGGATGGAAGACGGCAACGTGGACTTCATCGGCCGGATCGACAACCAGGCGAAAAT 16922
pmxE_1G_Polymyxin            gcggtggatggaggacggcaacgtggacttcatcggccggatcgacaaccaggcgaaaat 16877
pmxE_B-LR_Polymyxin-E        GCGGTGGATGGAGGACGGGAACGTGGACTTCATCGGCCGGATCGACAACCAGGCGAAAAT 17039
pmxE_KP26_Polymyxin-E        GCGGTGGATGGAGGACGGGAACGTGGACTTCATCGGCCGGATCGACAACCAGGCGAAAAT 16979
pmxE_Polymyxin-P             GCGGTGGATGGAGGACGGCAACGTGGACTTCATCGGCCGGATCGACAACCAGGCGAAAAT 17039
pmxE_Polymyxin-A             GCGGTGGATGGAGGACGGGAACGTGGACTTCATCGGCCGGATCGACAACCAGGCGAAAAT 17039
                             ************ ***** *****************************************

pmxE-D-DaB3-Polymyxin-B      CCGGGGCTACCGGATCGAGACGGGCGAGATCGAGTCGCAGTTGCTGCGGGTGGAAGGCGT 16982
pmxE_1G_Polymyxin            ccggggctaccggatcgagacgggcgaagtcgagtcgcagctgctgcgggtggaaggcgt 16937
pmxE_B-LR_Polymyxin-E        CCGGGGGTACCGGATTGAGACGGGCGAAGTCGAAGCGAAGCTGCTAAGTGTGGAAGGCGT 17099
pmxE_KP26_Polymyxin-E        CCGGGGGTACCGGATTGAGACGGGCGAAGTCGAAGCGAAGCTGCTAAGTGTGGAAGGCGT 17039
pmxE_Polymyxin-P             CCGGGGGTACCGGATTGAGACGGGCGAAGTCGAAGCGAAGCTGCTGAGTGTAGGTGGCGT 17099
pmxE_Polymyxin-A             CCGGGGCTACCGGATCGAGACGGGCGAGATCGAGTCGCAGCTGCTGCGGGTGGAAGGCGT 17099
                             ****** ******** ***********  ****  ** ** ****  * ** *  *****

pmxE-D-DaB3-Polymyxin-B      GCGCGAAGCGGTGGTGCTGGTTCAAAGTGACGCGAACGGGCAGAAAGCGCTGTGCGCGTA 17042
pmxE_1G_Polymyxin            gcgcgaagcggtggtgctggttcgaagtgacgcgaacgggcagaaagcgctgtgcgcgta 16997
pmxE_B-LR_Polymyxin-E        GCGCGAAGCGGTGGTGCTGGTTCGAAGTGACGCGAACGGGCAGAAAGCGCTGTGTGCGTA 17159
```

```
pmxE_KP26_Polymyxin-E        GCGCGAAGCGGTGGTGCTGGTTCGAAGTGACGCGAACGGGCAGAAAGCGCTGTGTGCGTA 17099
pmxE_Polymyxin-P             GAAGGAAGCGGTCGTCGTCGTCAGGGAAGATCAAGAGGGTCAGAAAGCTTTGTGCGCTTA 17159
pmxE_Polymyxin-A             GCGCGAAGCGGTGGTGCTGGTTCGAAGTGACTCGAACGGGCAGAAAGCGCTGAGCGCGTA 17159
                             *   ******** **  * **       **     * ** ********  ** * ** **

pmxE-D-DaB3-Polymyxin-B      CTACACAACGGATGGCGAGCTGACGGCGGCAGACCTGAAACGGGCGATCTCCAGGGAGCT 17102
pmxE_1G_Polymyxin            ctacacaacggatggcgaactgacggcggcagacctgaaacgggcgatctccagcgagct 17057
pmxE_B-LR_Polymyxin-E        CTACACAATTGATGGCGAATTTACAGCGGCAGACCTGAAACGGGCGATTGCCAGCGAGCT 17219
pmxE_KP26_Polymyxin-E        CTACACAATTGATGGCGAATTTACAGCGGCAGACCTGAAACGGGCGATTGCCAGCGAGCT 17159
pmxE_Polymyxin-P             TTATACAGCGGAAGAAGGCTTGACGGCGGCGGACCTGAAGCGGGCGATTGCCAGCGAGCT 17219
pmxE_Polymyxin-A             CTACACAATTGATGGCGAACTGACGGCGGCAGACCTGAAACGGGCGATCTCCAGCGAGCT 17219
                              ** ***   ** *  *   * ** ***** ******** ********  **** *****

pmxE-D-DaB3-Polymyxin-B      GCCGGGTTACATGATCCCGTCGTACTTCGTCGAGCTGGAGCGCCTGCCTCTGACGCCGAA 17162
pmxE_1G_Polymyxin            gccgggttacatgatcccgtcgtacttcgtcgagctggagcgcctgcctctgacgccgaa 17117
pmxE_B-LR_Polymyxin-E        GCCGGGGTACATGATCCCGTCGTACTTCGTGGAGCTGGAGCGCCTGCCTCTGACGCCGAA 17279
pmxE_KP26_Polymyxin-E        GCCGGGGTACATGATCCCGTCGTACTTCGTGGAGCTGGAGCGCCTGCCTCTGACGCCGAA 17219
pmxE_Polymyxin-P             GCCGGGGTACATGATCCCGTCGTACTTCGTGGAGATGGAGCGCCTGCCTCTGACGCCGAA 17279
pmxE_Polymyxin-A             GCCGGGTTACATGATCCCGTCGTACTTCGTGGAGCTGGAGCGCCTGCCTCTGACGCCGAA 17279
                             ****** *********************** *** *************************

pmxE-D-DaB3-Polymyxin-B      CGGAAAGATCGACCGGAAGGCGCTGCCGGCACCGGAAGGGGGAGCAGGCGCAGGCCGCGA 17222
pmxE_1G_Polymyxin            cgggaaaatcgaccggaaggcgctgccggcgccggaagggggagcaagcgcaggccgcga 17177
pmxE_B-LR_Polymyxin-E        CGGGAAAATCGACCGGAAGGCGCTGCCGGCGCCGGAAGGGGGAGCAAACGCAGGCCGCGA 17339
pmxE_KP26_Polymyxin-E        CGGGAAAATCGACCGGAAGGCGCTGCCGGCGCCGGAAGGGGGAGCAAACGCAGGCCGCGA 17279
pmxE_Polymyxin-P             CGGAAAGATCGACCGGAAGGCGCTGCCGGCGCCGGAAGGGGGAGCAAGCGCAGGCCGCGA 17339
pmxE_Polymyxin-A             CGGGAAAATCGACCGGAAGGAGCTGCCGGCGCCGGAAGGGGGAGCAAGCGCAGGCCGCGA 17339
                             *** ** ************* ********* ***************  ************

pmxE-D-DaB3-Polymyxin-B      ATACGTGGCGCCGCGCACCGAACTGGAGGCGAAACTGGTCGCCATCTGGCAGGACGTGCT 17282
pmxE_1G_Polymyxin            atacgtggcaccgcgcaccgaactggaggcgaaactggtctccatctggcaggacgtgct 17237
pmxE_B-LR_Polymyxin-E        ATACGTGGCGCCGCGCACCGAACTGGAGGCGAAACTGGTCGCCATCTGGCAGGACGTGCT 17399
pmxE_KP26_Polymyxin-E        ATACGTGGCGCCGCGCACCGAACTGGAGGCGAAACTGGTCGCCATCTGGCAGGACGTGCT 17339
pmxE_Polymyxin-P             ATATGTGGCGCCGCGCACCCAACTGGAGGCGAAACTGGTCGCCATCTGGCAGGACGTGCT 17399
pmxE_Polymyxin-A             ATACGTGGCGCCGCGCACCGAACTGGAGGCGAAACTGGTCGCCATCTGGCAGGACGTGCT 17399
                             *** ***** ********* ******************** *******************

pmxE-D-DaB3-Polymyxin-B      CGGGCCGGTCACGATTGGCGTGACGGACAACTTCTTCGACCTCGGCGGGCACTCCCTGCG 17342
pmxE_1G_Polymyxin            cgggccggtcacgattggcgtgacggacaacttcttcgacctcggcgggcactccctgcg 17297
pmxE_B-LR_Polymyxin-E        CGGGCCGGTCACGATTGGCGTAACGGACAACTTCTTCGACCTCGGTGGGCACTCCCTGCG 17459
pmxE_KP26_Polymyxin-E        CGGGCCGGTCACGATTGGCGTAACGGACAACTTCTTCGACCTCGGTGGGCACTCCCTGCG 17399
pmxE_Polymyxin-P             CGGGTCGGTCACGATTGGCGTGACGGACAACTTCTTCGACCTCGGCGGGCACTCCCTGCG 17459
pmxE_Polymyxin-A             CGGGCCGATCACGATTGGCGTGACGGACAACTTCTTCGACCTCGGCGGGCACTCCCTGCG 17459
                             **** ** ************* *********************** **************
```

```
pmxE-D-DaB3-Polymyxin-B      GGCGACGACGCTGGTCAGCAAGGTGCACAAGGAGCTGAGCGTGGACCTGCCGCTGCGCGA 17402

pmxE_1G_Polymyxin            ggcgacgacgctggtcagcaaggtgcacaaggagctgagcgtggacctgccgctgcgcga 17357

pmxE_B-LR_Polymyxin-E        GGCGACGACGCTGGTCAGCAAGGTGCACAAGGAGCTGAGCGTGGACCTGCCGTTGCGCGA 17519

pmxE_KP26_Polymyxin-E        GGCGACGACGCTGGTCAGCAAGGTGCACAAGGAGCTGAGCGTGGACCTGCCGTTGCGCGA 17459

pmxE_Polymyxin-P             GGCGACGACGCTGGTCAGCAAGGTGCACAAGGAGCTGAGCGTGGACCTGCCGCTGCGCGA 17519

pmxE_Polymyxin-A             GGCGACGACGCTGGTCAGCAAGGTGCACAAGGAGCTGAGCGTGGACCTGCCGCTGCGCGA 17519

                             **************************************************** *******


pmxE-D-DaB3-Polymyxin-B      TGTGTTCCGGCACTCGACCATCGAAGCGATGGCCGAAGCGATCAGCCAATTGGAGCGGCA 17462

pmxE_1G_Polymyxin            tgtgttccggcactcgaccatcgaagcgatggccgaagcgatcagccaattggagcggca 17417

pmxE_B-LR_Polymyxin-E        TGTGTTCCGGCACTCGACCATCGAAGCGATGGCCGAAGCGATAAGCCAATTGGAGCGGCA 17579

pmxE_KP26_Polymyxin-E        TGTGTTCCGGCACTCGACCATCGAAGCGATGGCCGAAGCGATAAGCCAATTGGAGCGGCA 17519

pmxE_Polymyxin-P             TGTGTTCCGGCACTCGACCATCGAAGCGATGGCCGAAGCGATCAGCCAATTGGAGCGGCA 17579

pmxE_Polymyxin-A             TGTGTTCCGGCACTCGACCATCGAAGCGATGGCCGAAGCGATCAGCCAATTGGAGCGGCA 17579

                             ****************************************** *****************


pmxE-D-DaB3-Polymyxin-B      GGAACACCTCTCCATTCCGGTTCTGGATAAGAGGGATTACTATCCGCTTTCCTCCGTGCA 17522

pmxE_1G_Polymyxin            ggaacacctctccattccagttctggataagagggattactatccgctttcctccgtgca 17477

pmxE_B-LR_Polymyxin-E        GGAACACCTCTCCATTCCGGTTCTGGATAAGAGGGATCACTATCCGCTTTCCTCCGTGCA 17639

pmxE_KP26_Polymyxin-E        GGAACACCTCTCCATTCCGGTTCTGGATAAGAGGGATTACTATCCGCTTTCCTCCGTGCA 17579

pmxE_Polymyxin-P             GGAACACCTCTCCATTCCGGTTCTGGATAAGAGGGATTACTATCCGCTTTCCTCCGTGCA 17639

pmxE_Polymyxin-A             GGAACACCTCTCCATTCCGGTTCTGGATAAGAGGGATTACTATCCGCTTTCCTCCGTGCA 17639

                             ****************** ****************** **********************


pmxE-D-DaB3-Polymyxin-B      AAAACGGCTGTACATCCAGCAGCAAATGGAAGGCGCTGAGCTTAGCTACAACATGTCCGG 17582

pmxE_1G_Polymyxin            aaaacggctgtacatccagcagcagatggaaggcgccgagcttagctacaacatgtccgg 17537

pmxE_B-LR_Polymyxin-E        AAAACGGCTGTACATCCAGCAGAAGATGGAAGGCGCCGAGCTTAGCTACAACATGTCCGG 17699

pmxE_KP26_Polymyxin-E        GAAACGGCTGTATATCCAGCAGCAGATGGAAGGCGCCGAGCTTAGCTACAATATGTCCGG 17639

pmxE_Polymyxin-P             AAAACGGCTGTACATCCAGCAGCAGATGGAAGGCGCCGAGCTTAGCTACAACATGTCCGG 17699

pmxE_Polymyxin-A             GAAACGGCTGTATATCCAGCAGCAGATGGAAGGCGCCGAGCTTAGCTACAATATGTCCGG 17699

                              *********** ********* * *********** ************** ********


pmxE-D-DaB3-Polymyxin-B      CATGACGGTTCTCGTCGGGCGCTTGGAACGGAATCAATTCGAGGCGGCGCTCAAAGGATT 17642

pmxE_1G_Polymyxin            catgacggttctcgtcgggcgtttggaacggaatcaattcgaggcggcgctcaaaggatt 17597

pmxE_B-LR_Polymyxin-E        CATGACGGTTCTCGTCGGGCGCTTGGAGCGGAATCAATTCGAGGCGGCGCTCAAAGGATT 17759

pmxE_KP26_Polymyxin-E        CATGACGGTTCTCGTCGGGCGTTTGGAACGGAATCAATTCGAGGCGGCGCTCAAAGGATT 17699

pmxE_Polymyxin-P             CATGACGGTTCTCGTCGGGCGCTTGGAACGGAATCAATTCGAGGCGGCGCTCAAAGGATT 17759

pmxE_Polymyxin-A             CATGACGGTTCTCGTCGGGCGTTTGGAACGGAATCAATTCGAGGCGGCGCTCAAAGGATT 17759

                             ********************* ***** ********************************


pmxE-D-DaB3-Polymyxin-B      GATAGCTCGTCACGAAATTTTGCGAACCGGCTTCGAAATGGTCGACGGCGAACCGGTACA 17702

pmxE_1G_Polymyxin            gatagctcgtcacgaaattttgcgaaccggcttcgaaatggtcgacggcgaaccggtaca 17657

pmxE_B-LR_Polymyxin-E        GATCGCTCGTCACGAAATTTTACGAACCGGCTTCGAAATGGTCGAAGGCATACCAGTACA 17819
```

```
pmxE_KP26_Polymyxin-E        GATAGCTCGTCACGAAATTTTGCGAACCGGCTTCGAAATGGTCGACGGCGAACCGGTACA 17759
pmxE_Polymyxin-P             GATAGCTCGTCACGAAATTTTGCGAACCGGCTTCGAAATGGTCGACGGCGAACCGGTACA 17819
pmxE_Polymyxin-A             GATAGCTCGTCACGAAATTTTGCGAACCGGCTTCGAAATGGTCGACGGCGAACCGGTACA 17819
                             *** ***************** *********************** ***  *** *****

pmxE-D-DaB3-Polymyxin-B      ACGAGTTTATCCGGACTTGAAGTTTGCCGTCGAGTATACGAAAGCGACGGAAAGTGAAAC 17762
pmxE_1G_Polymyxin            acggatttatccggacttgaagtttgccgtcgagtatacgaaagcgacggaaagtgaaac 17717
pmxE_B-LR_Polymyxin-E        ACGAATTTATCCGGAATTGAAGTTTGCTGTCGAGTATACGAAAGCGACGGAAAGTGAAAC 17879
pmxE_KP26_Polymyxin-E        ACGGATTTATCCGGACTTGAAGTTTGCCGTCGAGTATACGAAAGCGATGGAAAGTGAAAC 17819
pmxE_Polymyxin-P             ACGGATTTATCCGGACTTGAAGTTTGCCGTCGAGTATACGAAAGCGACGGAAAGTGAAAC 17879
pmxE_Polymyxin-A             ACGGATTTATCCGGACTTGAAGTTTGCCGTCGAGTATACGAAAGCGATGGAAAGTGAAAC 17879
                             ***  ********** *********** ******************* ************

pmxE-D-DaB3-Polymyxin-B      GAAGAGTATCGTAGACGGCTTTGTGCGCGTCTTTGATTTGGAGCGGCCGCCGCTGCTGCG 17822
pmxE_1G_Polymyxin            gaagagcatcgtagacggctttgtacgcgtctttgatttggagcggccgccgctgctgcg 17777
pmxE_B-LR_Polymyxin-E        GAAGAGCATCGCAGACGGCTTTGTGCGCGTCTTTGATTTGGAGCGGCCGCCGCTACTGCG 17939
pmxE_KP26_Polymyxin-E        GAAGAGCATCGTAGACGGCTTTGTACGCGTCTTTGATTTGGAGCGGCCGCCGCTGCTGCG 17879
pmxE_Polymyxin-P             GAAGAGCATCGTAGATGGCTTTGTGCGCGTCTTTGATTTGGAGCGGCCGCCGCTGCTACG 17939
pmxE_Polymyxin-A             GAAGAGCATCGTAGACGGCTTTGTACGCGTCTTTGATTTGGAGCGGCCGCCGCTGCTGCG 17939
                             ****** **** *** ******** ***************************** ** **

pmxE-D-DaB3-Polymyxin-B      TGTGGGCTTAGTCGAATGGGAAGCGGAACGGCATCTGCTCATGCTGGACATTCATCATAT 17882
pmxE_1G_Polymyxin            tgtgggcttagtcgaatgggaagcggaacggcatctgctcatgctggacattcatcatat 17837
pmxE_B-LR_Polymyxin-E        TGTGGGCTTAGTCGAATGGGAAGAGGAACGGCATTTGCTCATGCTGGACATTCATCATAT 17999
pmxE_KP26_Polymyxin-E        TGTGGGCTTAGTCGAAATGGAAGCGGAACGGCATTTGCTCATGCTGGACATTCATCATAT 17939
pmxE_Polymyxin-P             TGTGGGCTTAGTCGAATGGGAAGCGGAACGGCATCTGCTCATGCTGGACATTCATCATAT 17999
pmxE_Polymyxin-A             TGTGGGCTTAGTCGAAATGGAAGCGGAACGGCATCTGCTCATGCTGGACATTCATCATAT 17999
                             ****************  ***** ********** ************************

pmxE-D-DaB3-Polymyxin-B      CGTCACGGATGGCATGTCGATGGGCATCTTCGTCGAAGAGCTGCTGCGCCTGTATAACGG 17942
pmxE_1G_Polymyxin            cgtcacggatggcatgtcgatgggcatcttcgtcgaagagctgctgcgcctgtataacgg 17897
pmxE_B-LR_Polymyxin-E        CGTCACGGATGGCATGTCGATGGGTATCTTCGTCGAAGAGCTGCTGCGCCTGTATAACGG 18059
pmxE_KP26_Polymyxin-E        CGTCACGGATGGCATGTCGATGGGTATCTTCGTCGAAGAGCTGCTGCGCCTGTATAACGG 17999
pmxE_Polymyxin-P             CGTCACAGATGGCATGTCGATGGGCATCTTCGTCGAAGAGCTGCTGCGCCTGTATAACGG 18059
pmxE_Polymyxin-A             CGTCACGGATGGCATGTCGATGGGCATCTTCGTCGAAGAGCTACTGCGCCTGTATAACGG 18059
                             ****** ***************** ***************** *****************

pmxE-D-DaB3-Polymyxin-B      CGAGACTCTGGAACCGCTTCGGATTCAATACAAGGAATTTGCCGCTTGGCAGCAGTCCGA 18002
pmxE_1G_Polymyxin            cgagactctggaaccgcttcggattcaatacaaggaattcgccgcttggcagcagtccga 17957
pmxE_B-LR_Polymyxin-E        CGAGAATCTGGAACCACTTCGGATTCAATACAAGGAATTCGCCGCTTGGCAGCAGTCCGA 18119
pmxE_KP26_Polymyxin-E        CGAGAATCTGGAACCACTTCGGATTCAATACAAGGAATTCGCCGCTTGGCAGCAGTCCGA 18059
pmxE_Polymyxin-P             CGAGACTCTGGAACCGCTTCGGATTCAATACAAGGAATTCGCCGCTTGGCAGCAGTCCGA 18119
pmxE_Polymyxin-A             CGAGACTCTGGAACCGCTTCGGATTCAATACAAGGAATTCGCCGCTTGGCAGCAGTCCGA 18119
                             ***** ********* *********************** ********************
```

```
pmxE-D-DaB3-Polymyxin-B      ACCTGTAAAAGAGCGGCTGAAACGTCAGGAAGCCTACTGGCTGGACGTGCTGGAAGGCGA 18062
pmxE_1G_Polymyxin            acctgtaaaagagcggctgaaacgtcaggaagcctactggctggacgtgctggaaggcga 18017
pmxE_B-LR_Polymyxin-E        ACCTGTAAAAGAGCGGCTGAAACGTCAGGAAGCCTACTGGCTGGACGTGCTGGAAGGCGA 18179
pmxE_KP26_Polymyxin-E        ACCTGTAAAAGAGCGGCTGAAACGTCAGGAAGCCTACTGGCTGGACGTGCTGGAAGGCGA 18119
pmxE_Polymyxin-P             ACCTGTAAAAGAGCGGCTGAAACGTCAGGAAGCCTACTGGCTGGACGTGCTGGAAGGCGA 18179
pmxE_Polymyxin-A             ACCTGTAAAAGAGCGGCTGAAACGTCAGGAAGCCTACTGGCTGGACGTGCTGGAAGGCGA 18179
                             ************************************************************

pmxE-D-DaB3-Polymyxin-B      ACTGCCGACGCTTGAACTGCCTACGGACTTTGTCAGACCTGTCGCTCGCAGCTTTGAGGG 18122
pmxE_1G_Polymyxin            actgccgacgcttgaactgcctacggactttgtcagacctgccgctcgcagctttgaggg 18077
pmxE_B-LR_Polymyxin-E        ACTGCCGACGCTTGAACTGCCAACGGACTTTGTCAGACCTGCCGCTCGCAGCTTTGAGGG 18239
pmxE_KP26_Polymyxin-E        ACTGCCGACGCTTGAACTGCCAACGGACTTTGTCAGACCTGCCGCTCGCAGCTTTGAGGG 18179
pmxE_Polymyxin-P             ACTGCCGACGCTTGAACTGCCTACGGACTTTGTCAGACCTGCCGCTCGCAGCTTTGAGGG 18239
pmxE_Polymyxin-A             ATTGCCGACGCTTGAACTGCCTACGGACTTTGTCAGACCTGCCGCTCGCAGCTTTGAGGG 18239
                             * ******************* ******************* ******************

pmxE-D-DaB3-Polymyxin-B      AGATGTGCTGCCTTTCAGCATCGACAAGCAGATGACCGACAGCTTGCAGCGCATCGCCGA 18182
pmxE_1G_Polymyxin            agatgtgctgcctttcagcatcgacaagcagatgaccgacagcttgcagcgcatcgccga 18137
pmxE_B-LR_Polymyxin-E        AGATGTGCTGCCTTTCAGCATCGACAAGCAGATGACCGACAGCTTGCAGCGCATCGCCGA 18299
pmxE_KP26_Polymyxin-E        AGATGTGCTGCCTTTCAGCATCGACAAGCAGATGACCGACAGCTTGCAGCGCATCGCCGA 18239
pmxE_Polymyxin-P             AGATGTGCTGCCTTTCAGCATCGACAAGCAGATGACCGACAGCTTGCAGCGCATCGCCGA 18299
pmxE_Polymyxin-A             AGATGTGCTGCCTTTCAGCATCGACAAGCAGATGACCGACAGCTTGCAGCGCATCGCCGA 18299
                             ************************************************************

pmxE-D-DaB3-Polymyxin-B      TGAGAACGGTGCCACCCTTTATATGGTGTTATTGGCGGCCTATTCAATCCTGCTCAGCAA 18242
pmxE_1G_Polymyxin            tgagaacggtgccaccctttatatggtgttattggcggcctattcaatcctgctcagcaa 18197
pmxE_B-LR_Polymyxin-E        TGAGAACGGTGGCACCCTTTATATGGTGTTATCGGCGGTCTATTCAATCCTGCTCAGCAA 18359
pmxE_KP26_Polymyxin-E        TGAGAACGGTGGCACCCTTTATATGGTGTTATCGGCGGTCTATTCAATCCTGCTCAGCAA 18299
pmxE_Polymyxin-P             TGAGAACGGTGCCACCCTTTATATGGTGTTATTGGCGGCCTATTCAATCTTGCTCAGCAA 18359
pmxE_Polymyxin-A             TGAGAACGGTGCCACCCTTTATATGGTGTTATTGGCGGCCTATTCAATCCTGCTCAGCAA 18359
                             *********** ******************** ***** ********** **********

pmxE-D-DaB3-Polymyxin-B      GTACTCAGGACAAGAAGACTTCATTGTAGGGATACCGGTTTCGGGCCGCACACATGCCGA 18302
pmxE_1G_Polymyxin            gtactcgggacaagaagacttcattgtaggcacgccggtttcgggccgcacacatgccga 18257
pmxE_B-LR_Polymyxin-E        GTACTCGGGACAAGAAGATTTCATTGTAGGCACGCCGGTTTCAGGCCGCACACATGCAGA 18419
pmxE_KP26_Polymyxin-E        GTACTCGGGACAAGAAGATTTCATTGTAGGCACGCCGGTTTCAGGCCGCACACATGCAGA 18359
pmxE_Polymyxin-P             GTACTCGGGACAAGAAGACTTCATTGTAGGCACGCCGGCTTCGGGCCGCACACATGCCGA 18419
pmxE_Polymyxin-A             GTACTCGGGACAAGAAGACTTCATTGTAGGCACGCCGGTTTCGGGCCGCACACATGCCGA 18419
                             ****** *********** *********** *  **** *** ************** **

pmxE-D-DaB3-Polymyxin-B      CCTGGAGCCGCTGATCGGAATGTTTGTCAACACTTTGGCGATTCGCCATTATCCGTCCGG 18362
pmxE_1G_Polymyxin            cctggagccgctcatcggaatgtttgtcaacactttggcgattcgccattatccgtccgg 18317
pmxE_B-LR_Polymyxin-E        CCTGGAGCCGCTCATCGGAATGTTTGTCAACACTTTGGCGATTCGCCATTATCCGTCCGG 18479
```

```
pmxE_KP26_Polymyxin-E        CCTGGAGCCGCTCATCGGAATGTTTGTCAACACTTTGGCGATTCGCCATTATCCGTCCGG 18419
pmxE_Polymyxin-P             CCTGGAGCCGCTCATCGGAATGTTTGTCAACACTTTGGCGATTCGCCATTATCCGTCCGG 18479
pmxE_Polymyxin-A             CCTGGAGCCGCTCATAGGAATGTTTGTCAACACTTTGGCGATTCGCCATTATCCGTCCGG 18479
                             ************ ** ********************************************

pmxE-D-DaB3-Polymyxin-B      GGAGAAGATGTTCCTCGCTTACTTGAACGAAGTCAAAGAAACGATGCTGGGGGCCTACGA 18422
pmxE_1G_Polymyxin            ggagaagacgttcctcgcttacttgaacgaagtcaaagaaacgatgctgggggcctacga 18377
pmxE_B-LR_Polymyxin-E        GGAGAAGACGTTCCTCGCTTACTTGAACGAAGTCAAAGAAACGATGCTGGGGGCCTACGA 18539
pmxE_KP26_Polymyxin-E        GGAGAAGACGTTCCTCGCTTACTTGAACGAAGTCAAAGAAACGATGCTGGGGGCCTACGA 18479
pmxE_Polymyxin-P             GGAGAAGACGTTCCTCGCTTACTTGAACGAAGTCAAAGAAACGATGCTGGGGGCCTACGA 18539
pmxE_Polymyxin-A             GGAGAAGACGTTCCTCGCTTACTTGAACGAAGTCAAAGAAACAATGCTGGGGGCCTACGA 18539
                             ******** ********************************* *****************

pmxE-D-DaB3-Polymyxin-B      CCACCAGGATTATCCGTTCGAGGAGCTTGTGAAAAAGCTGCAGGCGCCGCGAGATCAAAG 18482
pmxE_1G_Polymyxin            ccaccaggattatccgttcgaggagcttgtgaaaaagctgcaggcgccgcgagatcaaag 18437
pmxE_B-LR_Polymyxin-E        TCACCAGGATTATCCGTTCGAGGAGCTTGTGAAAAAGCTGCAGGTTCCGCGAGATCTAAG 18599
pmxE_KP26_Polymyxin-E        TCACCAGGATTATCCGTTCGAGGAGCTTGTGAAAAAGCTGCAGGTTCCGCGAGATCTAAG 18539
pmxE_Polymyxin-P             CCACCAGGATTATCCGTTCGAGGAGCTTGTGAAAAAGCTGCAGGCGCCGCGAGATCAAAG 18599
pmxE_Polymyxin-A             GCACCAGGATTATCCGTTCGAGGAGCTTGTGAAAAAGCTGCAGGCACCGCGAGATCAAAG 18599
                              *******************************************  ********** ***

pmxE-D-DaB3-Polymyxin-B      CCGCAATCCTGTATTCTTTGTCATGTTTGTTCTGGAAACCAAGGAAGATAATGTTCAAAG 18542
pmxE_1G_Polymyxin            ccgcaatcctgtattcgatgtcatgtttgctttggaaaccaaggaagataacgttcaaag 18497
pmxE_B-LR_Polymyxin-E        CCGCAATCCTGTATTCGATGTCATGTTTGCTCTGGAAACCAAGGAAGATAACGTTCAAAA 18659
pmxE_KP26_Polymyxin-E        CCGCAATCCTGTATTCGATGTCATGTTTGCTCTGGAAACCAAGGAAGATAACGTTCAAAA 18599
pmxE_Polymyxin-P             CCGCAATCCTGTATTCGATGTCATGTTTGTTCTGGAAACCAAGGAAGATAACGTTCAAAG 18659
pmxE_Polymyxin-A             CCGCAATCCTGTATTCGATGTCATGTTTGCTCTGGAAACCAAGGAAGATAACGTTCAAAG 18659
                             ****************  *********** * ******************* ******* 

pmxE-D-DaB3-Polymyxin-B      CTTCGGGGATATCAAGATCGAATCTTATCCGGAAACTCATACGGTTTCCCAATTTGATCT 18602
pmxE_1G_Polymyxin            cttcggggatatcaagatcgaatcttatccggaaactcatacggtttcccaatttgacct 18557
pmxE_B-LR_Polymyxin-E        CTTCGGGGATATCAGGATCGAATCTTATCCGGAAACTCATACGGTTTCCCAATTTGATCT 18719
pmxE_KP26_Polymyxin-E        CTTCGGGGATATCAGGATCGAATCTTATCCGGAAACTCATACGGTTTCCCAATTTGATCT 18659
pmxE_Polymyxin-P             CTTCGGGGATATCAAGATCGAATCTTATCCGGAAACTCATACGGTTTCCCAATTTGATCT 18719
pmxE_Polymyxin-A             CTTCGGGGATATCAAGATCGAATCTTATCCGGAAACTCATACGGTTTCCCAATTTGATCT 18719
                             ************** ****************************************** **

pmxE-D-DaB3-Polymyxin-B      TACCTTGGTCATTTCGTTGCTGGATGAGGGAATGAACGGGCAGTTTGAATTTGCCACCAA 18662
pmxE_1G_Polymyxin            taccttggtcatttcgttgctggatgagggaatgaacgggcagtttgaatatgccaccaa 18617
pmxE_B-LR_Polymyxin-E        AACCTTGATCATTTCGTTGCTGGATGAGGGAATGAACGGGCAGTTTGAATATGCCACCAA 18779
pmxE_KP26_Polymyxin-E        AACCTTGATCATTTCGTTGCTGGATGAGGGAATGAACGGGCAGTTTGAATATGCCACCAA 18719
pmxE_Polymyxin-P             TACCTTGGTCATTTCGTTGCTGGATGAGGGAATGAACGGGCAGTTTGAATATGCAACCAA 18779
pmxE_Polymyxin-A             TACCTTGGTCATTTCGTTGCTGGATGAGGGAATGAACGGGCAGTTTGAATATGCCACCAA 18779
                              ******* ***************************************** *** *****
```

```
pmxE-D-DaB3-Polymyxin-B      GTTGTTTACGCGCAATCTGATCGACAATTTCGCTCAGGACCTGCTCGTGATCATCACCCA 18722

pmxE_1G_Polymyxin            gttgtttacgctcaatctgatcgacaatttcgctcaggacctgctcgtgatcatcaccca 18677

pmxE_B-LR_Polymyxin-E        GTTGTTCACACGCAATCTGATCGACAATTTCGCTCAGGACCTGCTCGTAATCATCTCTCA 18839

pmxE_KP26_Polymyxin-E        GTTGTTCACACGCAATCTGATCGACAATTTCGCTCAGGACCTGCTCGTAATCATCTCTCA 18779

pmxE_Polymyxin-P             GTTGTTTACGCGCAATCTGATCGACAATTTCGCTCAGGACCTGCTCGTGATCATCACCCA 18839

pmxE_Polymyxin-A             GTTGTTTACGCGCAATCTGATCGACAATTTCGCTCAGGACCTGCTCGTGATCATCACCCA 18839

                             ****** ** * ************************************ ****** * **


pmxE-D-DaB3-Polymyxin-B      AATTTGTGAACAGCCTTCGGTGCTGTTGAAGGATATTTCCCTGAACGGGCAATCCGAACA 18782

pmxE_1G_Polymyxin            aatttgcgaacagccttcggtgctgttgaaggatatttccctgaacgggcaatccgaaca 18737

pmxE_B-LR_Polymyxin-E        AATTTGCGAACAGCCTTCGGTGCTGTTGAAGGATATTTCCCTGAACGGGCAATCCGAACA 18899

pmxE_KP26_Polymyxin-E        AATTTGCGAACAGCCTTCGGTGCTGCTGAAGGATATTTCCCTGAACGGGCAATCCGAACA 18839

pmxE_Polymyxin-P             AATTTGCGAACAGCCTTCGGTGCTGTTGAAGGATATTTCCCTGAACGGGCAATCCGAACA 18899

pmxE_Polymyxin-A             AATTTGCGAACAGCCTTCGGCGCTGTTGAAGGATATTTCCCTGAACGGGCAATCCGAACA 18899

                             ****** ************* **** **********************************


pmxE-D-DaB3-Polymyxin-B      GGAGCAAGATGTGCTAGAGGCCATTGATATTATTTTCTAA 18822

pmxE_1G_Polymyxin            ggagcaagatgtgctagaggccattgatattattttctaa 18777

pmxE_B-LR_Polymyxin-E        GGAGCAAGATGTGCTAGAGGCCATTGATATTATTTTCTAA 18939

pmxE_KP26_Polymyxin-E        GGAGCAAGATGTGCTAGAGGCCATTGATATTATTTTCTAA 18879

pmxE_Polymyxin-P             GGAGCAAGATGTGCTAGAGGCCATTGATATTATTTTCTAA 18939

pmxE_Polymyxin-A             GGAGCAAGATGTGCTAGAGGCCATTGATATTATTTTCTAA 18939

                             ****************************************
```

Supplementary Figure S2: Alignment of *pmxA*, *pmxB* and *pmxE*

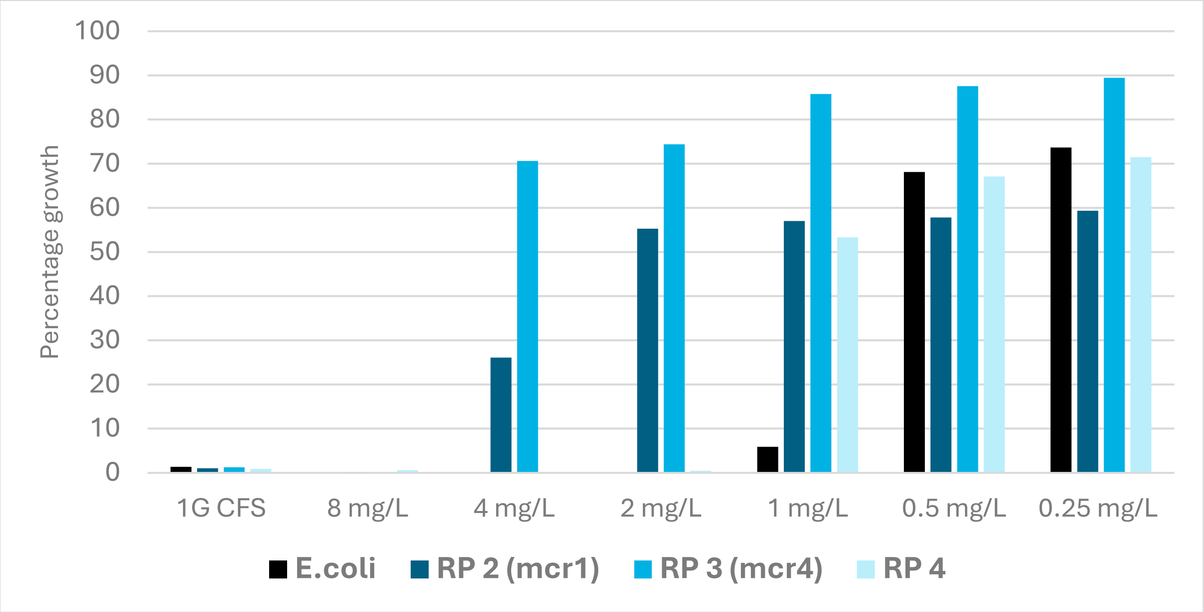

Supplementary Figure S3: Comparative percentage growth of resistance panel strains in the presence of colistin.

This shows that the isolates containing *mcr-1* (RP 2) and *mcr-4* (RP3) can grow in the presence of 4 mg/L colistin, above the EUCAST clinical breakpoint of 2 mg/L colistin. Isolate RP 4 could grow at 1 mg/L colistin, indicating a weak resistance mechanism may be present, but is not clinically resistant.
